# Urban sewage resistomes partially reflect clinical resistomes

**DOI:** 10.1128/msystems.00031-26

**Published:** 2026-03-13

**Authors:** Alix Vincent Thorn, Christian Brinch, Frank M. Aarestrup, Patrick Munk

**Affiliations:** 1 Research Group for Genomic Epidemiology, National Food Institute, Technical University of Denmark 114320 , Kgs. Lyngby, Denmark; University of Illinois Chicago, Chicago, Illinois, USA

**Keywords:** antibiotic resistance, clinical methods, microbial ecology, comparative studies, genomics, metagenomics, surveillance studies, computational biology, genetics, wastewater

## Abstract

Antimicrobial resistance (AMR) poses a major global public health threat, and ongoing surveillance of antimicrobial resistance genes (ARGs) is critical to mitigate current and future risks. Sewage-based ARG surveillance is gaining traction, but insight into how it compares to surveillance by clinical bacterial isolates is limited, especially when it comes to ARG mutational variants. We compared ARGs identified in clinical bacterial isolates (*n* = 2,989) with those detected in sewage metagenomes (*n* = 468) across 33 countries. ARG variant detection data from clinical isolates and sewage metagenomes shared some regional patterns in detection, but many ARG variants were detected exclusively in either sewage metagenomes or clinical isolates. We found that across all samples, only 69% of ARG clusters detected in clinical isolates were also detected via read mapping in sewage. Some ARGs highly prevalent in clinical isolates were not detected in sewage. Among clinically widespread ARGs, prevalence varied across bacterial species and clinical isolate types depending on whether the ARGs were also detected in sewage. This could indicate that sewage surveillance is better suited for detection of clinically relevant ARGs prevalent in certain bacterial species and infection sites than others. Spearman correlation between ARG abundance in sewage and the proportion of clinical isolates from the same country with detection was 0.28 overall, with stronger correlations for certain ARGs. The results demonstrate that sewage ARG profiles correlate, to some extent, to the clinical AMR landscape, but do not capture the full spectrum of clinically relevant ARGs at currently realistic sequencing depths.

**IMPORTANCE:**

Antimicrobial resistance (AMR) is a major public health threat. Surveillance of AMR is important and can be conducted via the detection of antimicrobial resistance genes (ARGs). Sewage can be used as a medium for surveillance as an alternative to analyzing individual bacterial isolates from health clinics. We compared detection in large global data collections of sewage metagenomes and clinical isolates. We found that while there were significant positive correlations between findings in sewage and clinical isolates, some widespread clinical ARGs were not detectable in sewage. This should be considered if establishing sewage surveillance systems.

## INTRODUCTION

Antimicrobial resistance (AMR) is a major threat to human health (1, 2). Infections caused by resistant bacteria are more difficult to treat, leading to longer illness and increased mortality and having major economic consequences (3). Antimicrobial resistance genes (ARGs) confer antimicrobial resistance to bacteria, and a range of different genes provide resistance to different classes of antibiotics. Resistant bacteria can vertically inherit ARGs or acquire them horizontally (4). Use of antibiotics, especially overuse and wrong use, is the main driver of AMR, but other factors contribute to its spread and prevalence (3, 5).

Active surveillance of antimicrobial-resistant bacteria can help limit the spread and emergence of AMR (6). The ARGs most prevalent in a given area, host, or bacterial taxa can change over time due to changes in antibiotic use, other anthropogenic factors (5), or clonal or plasmid replacement (7, 8). It is important to detect their introduction and dissemination early on. Since AMR is a global problem, it is pivotal to get sufficient data from all regions of the world (9), and surveillance is essential to both guide and evaluate the effect of interventions (3). Adequate AMR surveillance has not yet been implemented (10), and especially in low- and middle-income countries, the data are insufficient (9).

The antibiotics use data from an area can—if accurate—to some degree give an understanding of what is likely the most prevalent resistance, but studies have shown (5) that antimicrobial use data is not significantly associated with the global ARG abundance measured in sewage. In order to determine which AMR types are problematic in an area, the gold standard is to use pheno- and genotypic resistance tests on clinical microbial isolates.

Surveillance of ARGs using sewage has gained increased interest in recent years (9) since it is more effective in terms of labor and costs than surveillance via individual clinical isolates. In fact, wastewater-based AMR surveillance is being mandated in all EU member states from 2028 (11).

Sewage contains toilet waste from large populations, and a single sample therefore represents material from a broad, mixed subset of the population in a given area. In contrast to surveillance based solely on clinical isolates, sewage-based surveillance includes contributions from individuals without medical access as well as healthy individuals, thus increasing population coverage (9). This broad sampling has many benefits; however, clinical pathogens constitute a relatively small proportion of the bacteria sampled. Sewage also contains environmental bacteria and, in some cases, animal-derived material; thus, sewage surveillance also detects ARGs not associated with humans. However, the efficiency of wastewater surveillance makes it feasible to scale sampling across large geographical areas or to generate time-series data from one location over time, as done by Becsei et al. (12).

The Global Sewage Project has collected sewage samples from all over the world for several years, and sewage has proven useful for quantifying the occurrence and abundance of ARGs globally (13–15). The approach has been able to highlight systematic differences in abundance and diversity of ARGs from different regions of the world, as well as the large genomic diversity in areas flanking resistance determinants (13, 14).

There have been limited investigations into the association between observed AMR among human clinical isolates and that observed in sewage. Karkman et al. found a correlation between AMR from *Escherichia coli* isolates and in sewage metagenomes (16).

Njage et al. (5) observed significant positive correlations for some human pathogens belonging to taxa typically found in the intestinal tract, suggesting that sewage surveillance might only be predictive for some pathogens. However, these studies only compared clinical bacterial phenotypes with metagenomic class-level ARG abundances and did not investigate any correlations or regional differences in individual ARGs.

The clinical sensitivity and utility of wastewater surveillance of AMR remain poorly understood (6). Common sense suggests that ARGs that are mainly found in bacteria that cause infections in tissue with limited connection to the toilet waste-producing organs will be less detectable in sewage. As sewage surveillance of AMR becomes more widespread, it is crucial to understand both if it can detect the full spectrum of clinical ARGs and how well sewage data reflects the prevalence of clinical ARGs in an area.

In this study, we compared the ARGs detected in sewage and clinical samples across 33 countries. We focused on mobile/acquired ARGs so that the ARGs could be analyzed independently of bacterial species. We analyzed patterns in regional ARG variant detection across sewage metagenomes and clinical isolates. We analyzed the overlap of ARGs (90% homology clusters) detected in both sewage and clinical isolates and examined the diversity of clinical isolates harboring those genes. Finally, we assessed the association between country-wise ARG prevalence in the clinics and the sewage.

## RESULTS

### Data sets and detection of ARGs

We analyzed the ARGs in 468 sewage metagenomes from The Global Sewage Project (13–15) and assemblies of 2,989 clinical bacterial isolates from the Two Weeks in the World (TWIW) project (17) from the 33 countries that were included in both data sets (Fig. S1A through C). The clinical isolates derived from a range of sample types, with the following distribution: urine (*n* = 1,170), wound pus or biopsy (*n* = 524), blood (*n* = 503), respiratory system (*n* = 402), swabs (*n* = 225), and other sources (*n* = 220) (Fig. S1B). The isolate data set consisted of randomly selected clinical isolates from 59 diagnostic units, more details in Nag et al. (17) and were chosen because of the similar sampling protocol followed across all countries.

Sewage metagenome assemblies and clinical isolate assemblies were analyzed for the presence of gene sequences of mobile ARGs. Each unique sequence observed in the assemblies was defined as an ARG variant. Although the number of samples (Fig. S2A) varied between countries, the average number of different ARG variants detected per country was nearly identical between the sewage and clinical data sets (Fig. S2B). The average number of ARG variants detected per sample was 2.28 for clinical isolates and 16.83 for sewage metagenomes. Across data from all 33 countries, we detected ARGs 6,819 times in the clinical isolate assemblies and 7,898 times in the sewage metagenome assemblies.

### Sewage resistomes partially reflect regional patterns in clinical resistomes

We detected 1,870 ARG variants in total across 33 countries, of which 626 were detected more than once. Of those, 393 were detected in clinical isolates, and 335 were detected in sewage metagenomes, with 102 detected in both data sets.

**Fig 1:**
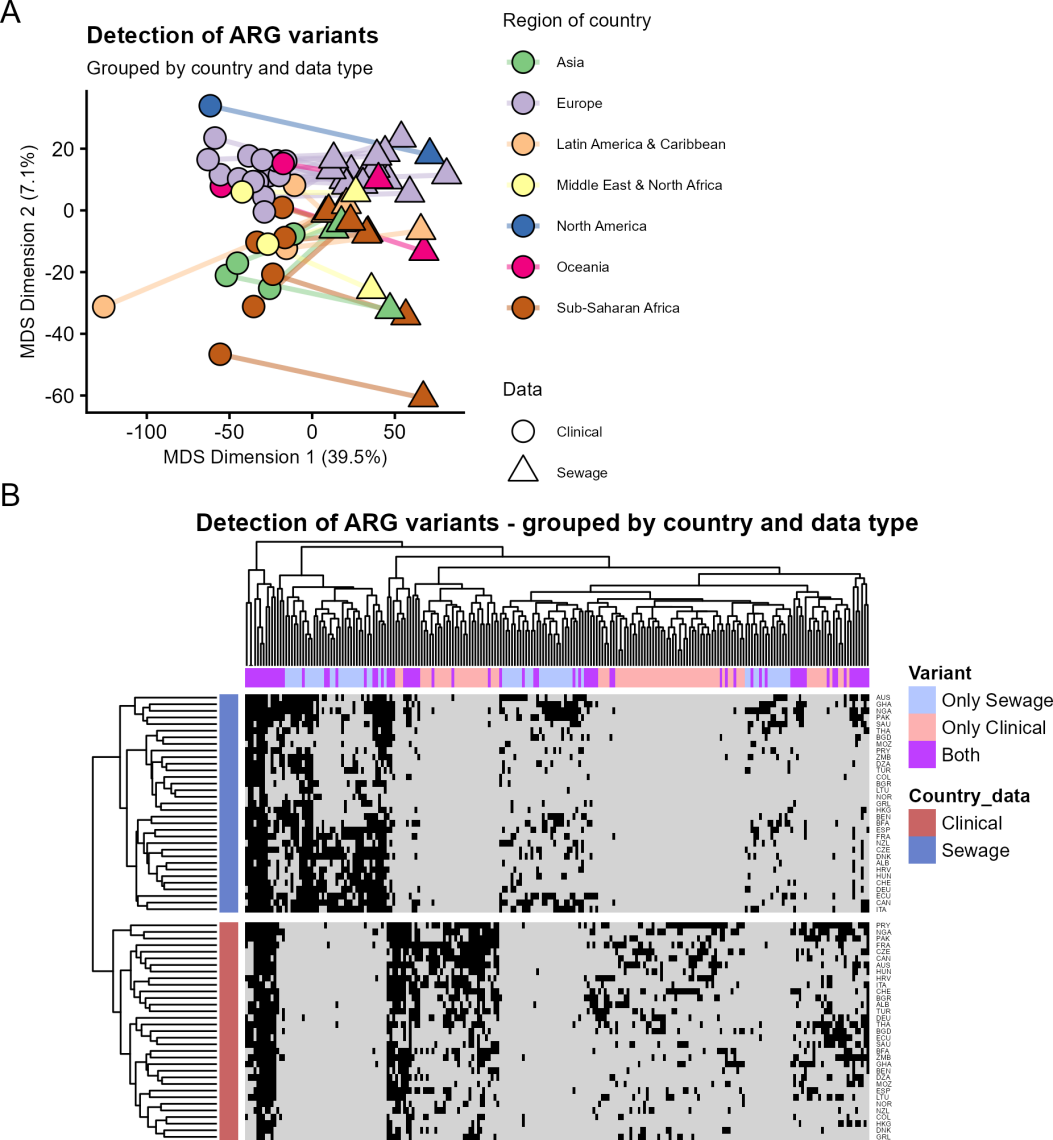
Presence/absence of detection of ARG variants in sewage metagenomes and clinical isolates. (**A**) Classical multidimensional scaling (MDS) of ARG variant detection data for each country. Every point represents either clinical (circle) or sewage (triangle) detection data. The color indicates the world region, and lines connect sewage and clinical data points for the same country. The first and second principal coordinates capture 46.6% of the variance. (**B**) Hierarchical clustering of ARG variant detection data grouped by country. Blue rows represent detection data from sewage, and red rows represent detection data from clinical isolates. ARG variants detected in at least five countries were included. Column color indicates whether the ARG variant was detected only in sewage (light blue), only in clinical isolates (pale pink) or in both (purple). Black color represents ARG detection in the heatmap.

Country-wise binary absence/presence data for detection of ARG variants in either sewage or clinical isolates was visualized with MDS (Multidimensional scaling) (Fig. 1A). There was a clear separation between clinical and sewage-derived resistomes, with the first and second principal coordinates capturing 46.6% of the variance. Data points representing countries from the same geographical regions tended to have more similar resistomes (Fig. 1A), regardless of whether the data represented sewage or clinical samples, suggesting some conserved geographical patterns between both sewage and clinical resistomes. Since MDS aims to preserve the pairwise distances and the global structure, we also applied UMAP, a method that favors the local structure (18), to the data (Fig. S3) with similar results.

When an ARG variant was detected in both sewage and clinical isolates from the same country, we defined this as a codetection. Codetection was more frequent than expected by chance (one-tailed permutation test, *P* = 0.001) (Fig. S4). This means that even though the country-specific sewage ARG detection data did not perfectly predict clinical detection, it did have some predictive value with regard to clinical detection.

### Many ARG variants are detected in either sewage or clinical isolates

Country-wise absence/presence detection data for common and widespread ARG variants in sewage and clinical isolates were analyzed with hierarchical clustering (Fig. 1B). All clinical resistomes were more similar to each other than to sewage resistomes, and vice versa. Many ARG variants were exclusively or mostly detected in either sewage metagenomes or clinical isolates across multiple countries.

The analysis from Fig. 1B was redone with the same ARG variants and sewage detection data, but only including clinical detections from either one of the four most common isolate types (Fig. S5A through D) or the four most common bacterial species (Fig. S6A through D) in the isolate data set. We observed that when comparing ARG variant detection data from sewage with detections in only clinical isolates of either type “Blood,” “Respiratory System,” “Wound Pus Biopsy,” or “Urine” (Fig. S5A through D), there were, similar to when all isolates were included, still many ARG variants that were detected clinically but not in sewage. Very few ARG variants were detected in *E. coli* isolates and not in sewage (Fig. S6A), despite the majority of the clinical isolates (28.1%) being from *E. coli*. More ARG variants that were not detected in sewage were found in *Staphylococcus aureus*, *Klebsiella pneumoniae*, and *Pseudomonas aeruginosa* isolates, which represented 12.6%, 12.5%, and 7.6% of the clinical isolate data set, respectively. Across all categories of clinical isolates (Fig. S5A thorugh D and S6A through D), we observed that the country-wise clinical isolate resistomes were, apart from a few exceptions, more similar to each other than to sewage resistomes.

### The overlap of ARGs detected in sewage and clinical samples within each country is limited

We also analyzed the ARGs at a higher level than exact variants. For the remainder of the study, we analyzed the ARGs in groups of genes with similar DNA sequences (90% homology clusters). For the detection of ARG in sewage, we used a read-based approach instead of assemblies, as we considered this the more likely choice for routine surveillance. A comparison of the lengths of ARGs detected in sewage via the read-based or assembly-based method (Fig. S7A) showed that they were similar. Fig. S7B through C shows the number of different ARGs detected per country in relation to the total number of bacterial reads or clinical isolates available for analysis. While there is a minor trend of detecting a higher number of different ARGs in countries with more data, the curve quickly flattened out, and the number of different ARGs detected within countries with similar amounts of input data varied.

**Fig 2:**
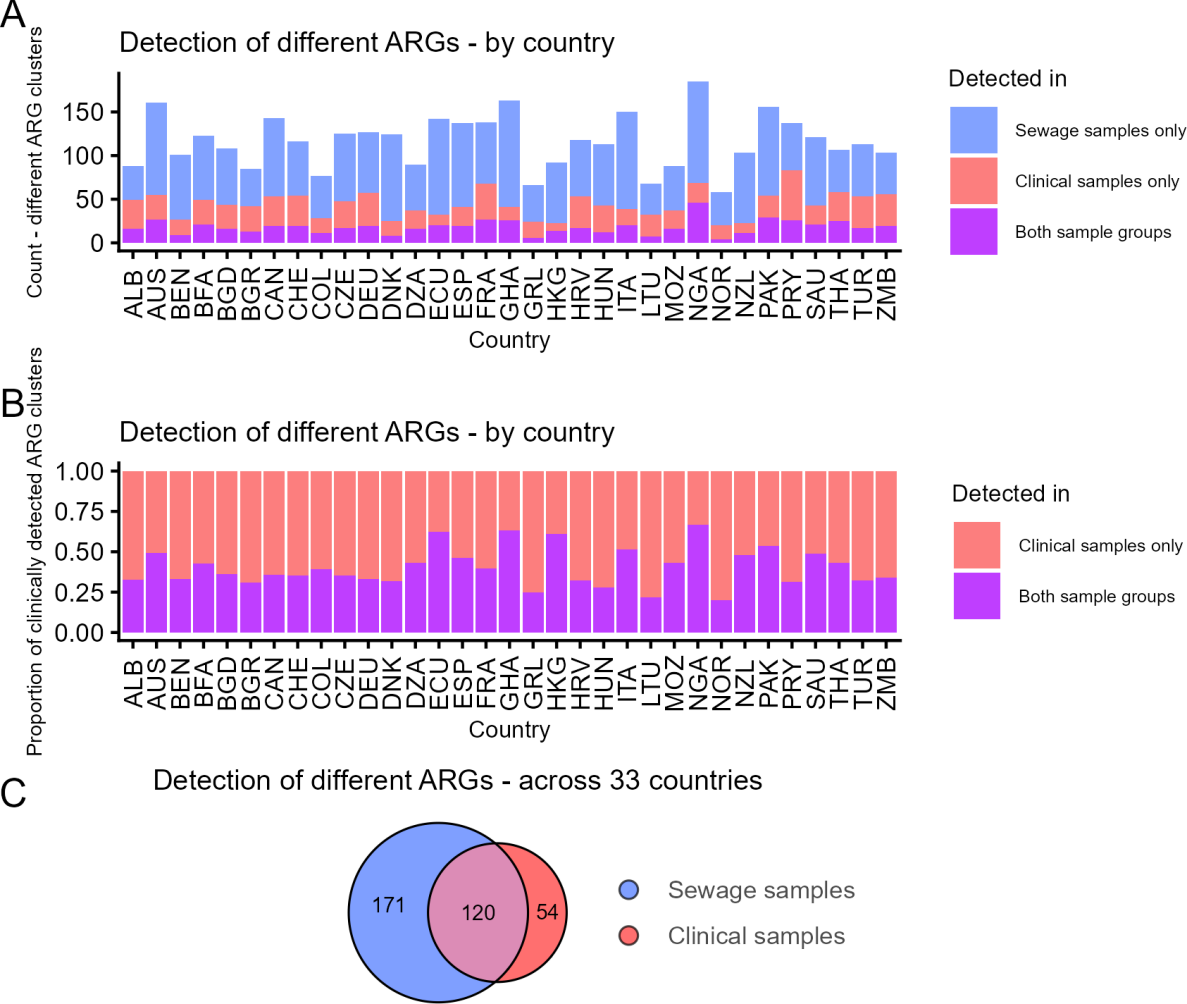
Different ARGs (90% homology clusters) detected in clinical isolates and sewage metagenomes from 33 countries. (**A**) Within each country, the number of different ARGs detected within clinical isolates only (red), sewage metagenomes only (blue) or in both types of samples (purple). (**B**) Country-wise proportion of clinically detected ARGs that were detected in clinical isolates only (red), or in both sewage and clinical isolates (purple). (**C**) Overlap of ARGs detected in sewage metagenomes and clinical isolates across all 33 countries.

Across all the samples from 33 countries, we detected 345 different ARGs. The total number of different ARGs detected per country varied from 58 to 185 (Fig. 2A). The percentage of ARGs detected in clinical isolates that were also detected in sewage within each country was on a similar level for most countries (40.3% on average) (Fig. 2B).

A total of 174 different ARGs were detected in clinical isolates, and 291 were detected in sewage metagenomes (Fig. 2C). Of all 345 ARGs, 34.8% (*n* = 120) were detected in both sewage metagenomes and clinical isolates, 15.7% (*n* = 54) only in clinical isolates, and 49.6% (*n* = 171) only in sewage. Given that sewage contains non-human bacteria, it is unsurprising that some ARGs are exclusively detected in sewage. However, only 69.0% of the ARGs detected in clinical isolates were also detected in sewage from any of the 33 countries (Fig. 2C).

### The number of ARGs detected in both sewage and clinical samples approaches saturation when including more data

The fraction of clinically detected ARGs (90% homology clusters) that were also found in sewage was lower than we expected. We wanted to assess whether this discrepancy could be explained by the number of countries sampled. We used rarefaction analysis on ARG detection data. For each country added, we calculated the number of different ARGs detected. We also calculated how many of those that were among the 120 ARGs detected in both sewage and clinical samples when data from all 33 countries were included.

Over half of the ARGs detected in both sewage and clinical samples had been detected when clinical isolates from just 5 out of 33 countries were included. The curve later approached a plateau, indicating that adding more clinical samples would likely not majorly impact the number of ARGs detected in both sewage and clinical samples (Fig. S8A).

More than half of ARGs detected in both sewage and clinical samples across 33 countries were identified by including sewage metagenomes from just 6 of those countries, after which the detection curve plateaued (Fig. S8B). Expanding the 33-country data set of 468 sewage metagenomes with 765 sewage metagenomes from 77 additional countries only increased the number of ARGs detected in both sewage and clinical isolates from 120 (at 33 countries) to 132 (at 110 countries) (Fig. S8C).

The amount of ARGs detected in both sewage and clinical data appeared to partially saturate with the inclusion of more and more samples. We similarly performed rarefaction analysis on ARG variants (Fig. S9) instead of ARG clusters, yielding similar results.

### Some ARGs that are widespread in clinical samples are not detected in sewage

We investigated whether clinical ARGs that were not detected in sewage were simply less widespread than those detected. Many of the clinically detected ARGs (90% homology clusters) without detection in sewage were nonetheless widespread and detected in multiple countries (Fig. S10A). Among all ARGs that were detected in clinical isolates, the percentage of those detected in clinical isolates from 15 countries was only slightly higher for those with sewage detection (22.5%) than for those with only sewage detection (18.5%). Many ARGs without detection in sewage are detected in numerous clinical isolates (Fig. S10B). 29.6% of ARGs only detected in clinical isolates were detected in at least 0.5% of all clinical isolates.

When grouping the ARGs by the number of countries in which they were detected via the clinical isolates, we found that the average number of clinical isolates with detection was similar or only slightly higher for clinically detected ARGs with sewage detection than those without (Fig. S10C).

Overall, we found that neither the frequency of detection in clinical isolates nor the number of countries with clinical detection appeared to be the primary factor distinguishing clinically detected ARGs that were also detected in sewage from those that were not. Very few of the ARGs that were detected only in clinical isolates belonged to the folate pathway antagonist class, compared to ARGs detected in sewage (Fig. S11). Among ARGs detected only in sewage, the proportion of beta-lactams was much higher than the proportion of aminoglycosides, while the proportions were almost equal among ARGs detected only in clinical isolates.

### Detection proportion across different isolate groups varies between ARGs with and without sewage detection

**Fig 3:**
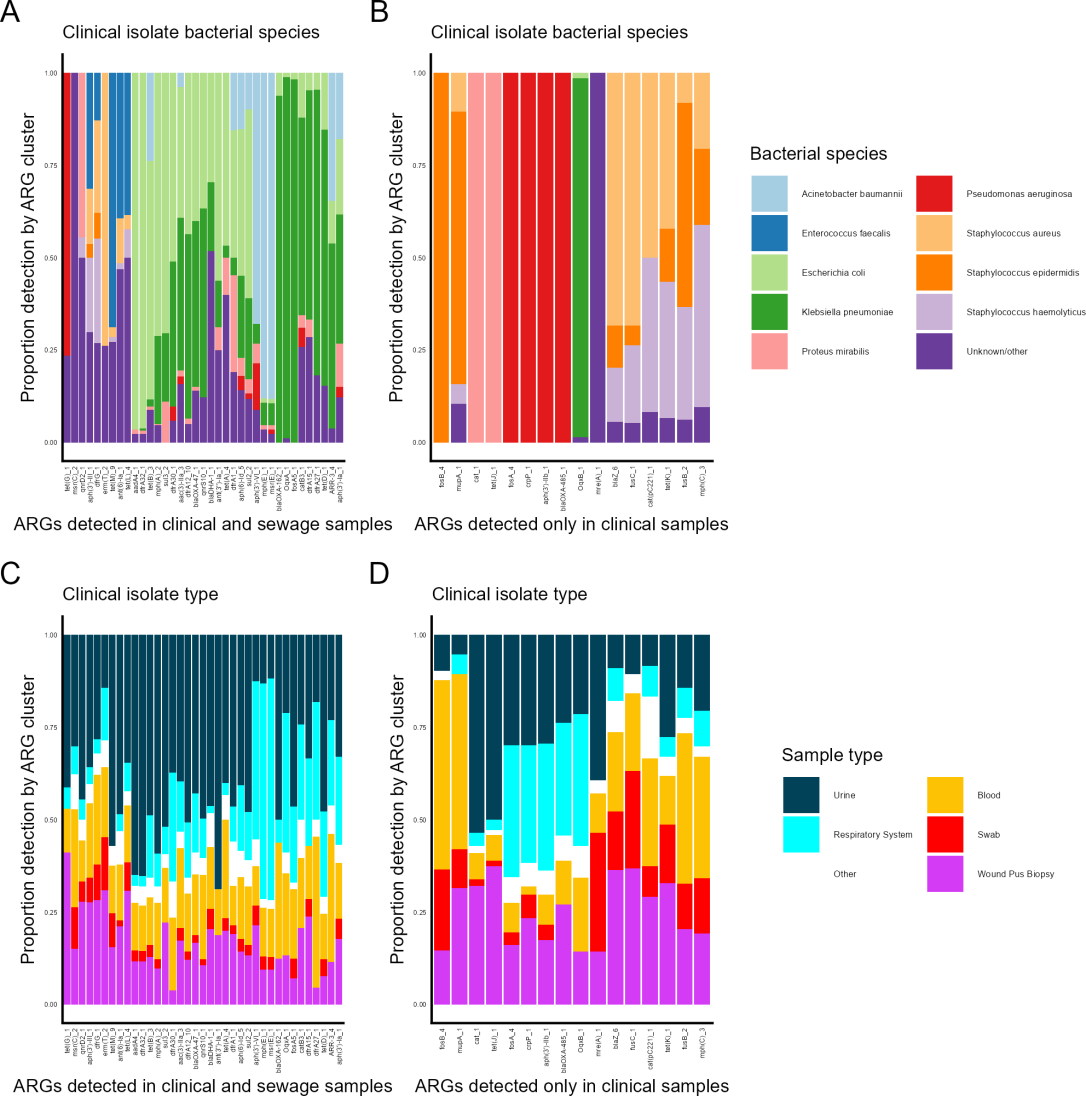
Prevalence of geographically widespread and clinically detected ARGs (90% homology clusters) across different categories of clinical isolates. (**A**) Proportion of detection among different bacterial species for ARGs detected in both sewage and clinical samples. (**B**) Proportion of detection among different bacterial species for ARGs detected only in clinical samples. (**C**) Proportion of detection in different clinical isolate types for ARGs detected in both sewage and clinical samples. (**D**) Proportion of detection in different clinical isolate types for ARGs detected only in clinical samples.

We wanted to study which clinical isolate categories ARGs were most frequently detected in, depending on whether or not they were also detected in sewage. ARGs (90% homology clusters) that were widespread in clinical isolates were divided into two subsets: those that were detected only in clinical isolates (*n* = 16) and those that were widely detected in sewage metagenomes as well (*n* = 37). Most ARGs were mainly detected in isolates of a few bacterial species. Detection in *K. pneumoniae* and *E. coli* was more common among ARGs detected in both sewage metagenomes and clinical isolates (Fig. 3A) than among those detected only in clinical isolates (Fig. 3B). Detection in *P. aeruginosa* or a combination of *S. aureus*, *S. epidermidis*, and *S. haemolyticus* was more associated with ARGs only detected in clinical isolates (Fig. 3B) than those also detected in sewage metagenomes (Fig. 3A).

Most ARGs were detected in all sample types (Fig. 3C and D), but there were differences in the proportion they were detected in the different sample types. More ARGs with detection in both clinical isolates and sewage metagenomes had a high proportion of detection in urine isolates (Fig. 3C) compared to ARGs without sewage detection (Fig. 3D). 70.3% of the ARGs with both sewage and clinical detection and 18.8% of those with only clinical detection had at least one-third of their clinical isolate detections in urine isolates. 16.2% of the ARGs with both sewage and clinical detection and 62.5% of those with only clinical detection had at least one-third of their clinical isolate detections in isolates of swabs, wounds, pus, or biopsies (Fig. 3C and D).

Which bacterial species a clinically relevant ARG is typically carried by, and which body sites it is often found in, appear to influence the ARGs likelihood of being transferred to sewage in detectable quantities. ARGs found in clinical isolates of the same bacterial species often had similar proportions of detection in different isolate types (Fig. 3A through D).

### Correlation between country-wise clinical ARG detection prevalence and abundance in sewage

We assessed whether, for ARGs detected in both sewage and clinical isolates, the abundance in sewage correlated with their prevalence in clinical isolates. To assess correlation rather than presence/absence, only data points where the same ARG was detected in both sewage and in clinical isolates from the same country were included.

**Fig 4:**
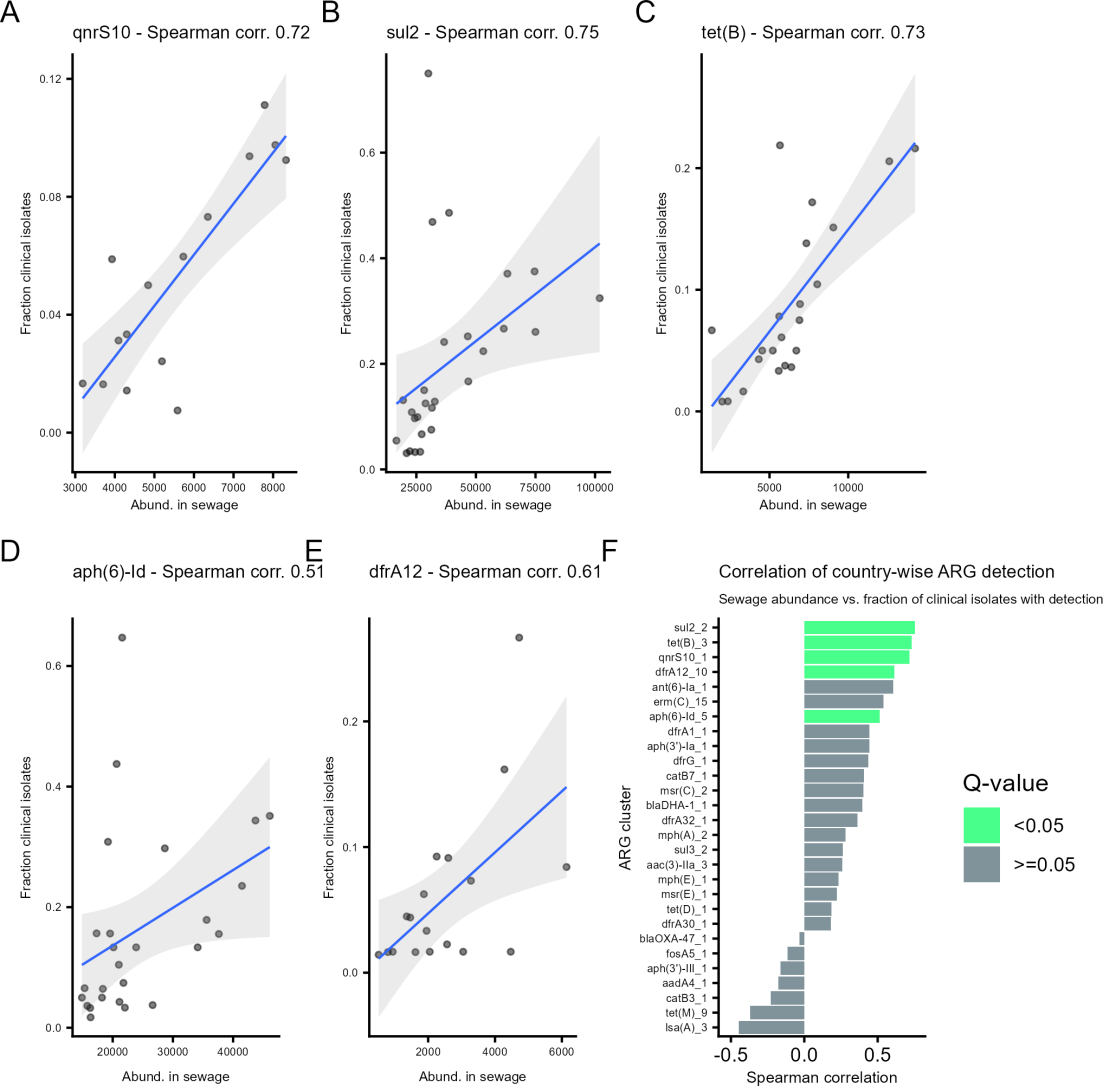
Correlation of country-wise clinical isolate prevalence and sewage abundance of ARGs (90% homology clusters). (**A–E**) Correlation plots for the five ARGs with a significant positive correlation. Each point represents data from one country, with sewage abundance on the *x*-axis and the fraction of clinical isolates with detection on the *y*-axis, and a linear regression line (blue). (**F**) Comparison of Spearman’s rho for all 28 ARGs with enough data for the analysis. *Q*-values (adjusted *P* values) below 0.05 are marked by a green bar.

Analyzing data from all ARGs together, there was a minor positive correlation (Spearman’s *ρ*: 0.28) between the country-wise abundance level in sewage and the fraction of clinical isolates with detection (Fig. S12 top left). Twenty-eight ARGs (90% homology clusters) had sufficient data for correlation analysis (Fig. S12). Five of those showed strong positive and significant correlation between country-wise sewage abundance and clinical prevalence (Fig. 4F): *qnr*S10, *sul*2, *tet*(B), *aph* (6)-ld, and *dfr*A12. Fourteen ARGs had a Spearman’s *ρ* above 0.3.

We also split the analysis to include either only clinical isolates of urine origin (Fig. S13B), all isolates not from urine (Fig. S13C), or all isolates in total (Fig. S13A) when calculating clinical prevalence, the results were similar. Finally, we calculated the correlation for the ARGs grouped by resistance classes (Fig. S14), and significant positive correlation was found for five out of eight classes. The strongest correlation between sewage abundance and clinical prevalence was found for quinolone resistance genes (Fig. S14H).

Overall, we found that for many ARGs, there was a positive correlation between the country-wise abundance in sewage and the percentage of clinical isolates with detection.

## DISCUSSION

The primary objective of this study was to compare the resistome of wastewater-based surveillance with clinically relevant ARGs. So far, limited research has been conducted on this topic, and previous studies have, to the best of our knowledge, only utilized surveillance data from a few selected pathogens generated in different studies (5, 16). Here we sought to compare clinical and sewage data sets, which each have been collected following the same protocol in the different countries in order to enhance comparability (13–15, 17).

The Two Weeks in the World (17) clinical bacterial isolate data set and the Global Sewage data set (13–15) are, however, very expensive and importantly generated in a comparative way across countries. The random sampling strategy employed in the collection of the Two Weeks in the World data set allows the distribution of isolates and sample types to approximate that of the actual clinical landscape. Integrating this data set with other kinds of isolated collections could have compromised this.

Our analysis revealed a moderate overlap between ARGs detected in sewage and those in clinical isolates. Studies have found that human fecal bacteria account for only a subset of the sewage community (19, 20), and it was expected that sewage contained ARGs not detected in clinical isolates. 31% of the ARG clusters detected in clinical isolates were not detected in any sewage samples. Some of those were rare and may not have been detected due to the stochastic nature of sewage sampling. However, among ARGs that were prevalent and widespread in the clinical isolates, there was still a substantial group without detection in sewage. This suggests that reliance on sewage surveillance alone could result in omission of clinically important ARGs. Analysis of exact ARG variants showed that clinical resistomes were more similar to each other than to sewage resistomes.

Various human body sites harbor different bacterial communities (21). One study (22) found that the mobile ARG transfer network was primarily shaped by bacterial phylogeny and to some degree also ecological barriers (e.g., human body sites). Our analysis of ARGs prevalent in clinical isolates confirmed that many were predominantly found in one or two bacterial species and that ARGs within the same species had similar detection proportions across sample types.

The ARGs that were detected in both clinical isolates and sewage metagenomes were more often detected in *E. coli* and *K. pneumoniae* and slightly more often associated with urine samples, compared to those only detected in clinical isolates. The ARGs found only in clinical isolates were detected more in *P. aeruginosa*, *S. aureus, S. epidermidis,* and *S. haemolyticus,* and more of them were strongly associated with clinical samples from swaps, wounds, pus, and biopsies than the ARGs also detected in sewage.

Since humans rarely discharge materials such as pus into the toilet, it makes sense that ARGs associated with bacteria that cause skin and soft-tissue infections are less likely to be detectable in sewage, even if prevalent in clinical settings. ARGs found in bacteria involved in urinary tract infections or native to the gut would have a higher likelihood of being in sewage at detectable levels.

More research is needed, but we recommend that when using sewage for surveillance of clinical ARGs, one is mindful that sewage does not capture the full spectrum of ARGs equally well and consider using it in tandem with clinical surveillance.

ARG variant detection data showed that some major trends of regional differences in resistomes were shared across both sewage and clinical data, and we found that sewage resistomes reflected the clinical resistomes more than expected by chance. In this study, data were aggregated at the national level rather than by individual cities and were collected over a 6-year period. It is plausible that sewage and clinical data collected simultaneously from the same city would be more similar.

We observed a correlation between the abundance of detected ARGs in sewage and the percentage of detection in clinical isolates from the same country. This suggests that sewage levels can serve as a rough indicator of clinical ARG levels for ARGs with an established correlation, and sewage surveillance may function as an early warning system for increasing clinical ARG levels.

In conclusion, country-level sewage ARG data partially reflect the presence and abundance of clinical ARGs in isolates but do not capture the full spectrum of clinical ARGs equally well.

## MATERIALS AND METHODS

### Sewage metagenomes

The Global Sewage Project (13–15) has collected sewage samples from around the world over several years, with the analysis of AMR content as one of its main objectives. In this study, we used contigs from assemblies of the sewage metagenomes for our analyses of ARG variants in sewage. Protocol, DNA purification, and sequencing are explained in Hendriksen et al. (13).

We included all metagenomic assemblies published in Munk et al. (14), excluding negative controls, technical replicates, and a few with naming mistakes. Among the samples that were technical replicates, we chose those with the most fragments sequenced. We also included newer metagenomes published by Martiny et al. (15). Among those, we excluded a small number of assemblies that did not have successful assemblies. All the assemblies were filtered so that only scaffolds longer than 999 base pairs were used for the analyses.

The sewage metagenomes analyzed in this study were collected between 2016 and 2021 from 110 countries. Thirty-three of these countries (Table S1) overlapped with countries from which we also had clinical bacterial isolates (collection described below). A total of 468 sewage metagenomes from these countries comprised the main sewage data set. An additional 765 metagenomes from 77 countries (Table S2) were used exclusively for rarefaction analysis. ENA accession numbers for all metagenomes and more details are provided in Table S4.

Low-abundant organisms are especially hard to recover with metagenomic assembly. Due to the computational demands of metagenomic assembly, we considered a read-based approach for ARG detection in sewage as the more likely choice for routine surveillance. Since it is not ideal for analyzing exact gene variants, we first used assemblies for the variant analysis and then also a read-based method for profiling sewage resistomes. For analysis of ARG (90% homology clusters) prevalence and abundance in sewage, we used ARGprofiler (23) read-mapping results from Martiny et al. (15), which were available for all sewage metagenomes.

### Clinical bacterial isolates

The clinical bacterial isolates used in this study were collected and processed in the Two Weeks in the World (TWIW) Project, which collected clinical bacterial isolates from human patients around the world in 2020. Approximately 60 samples were collected from each of 59 diagnostic units. The samples were randomly selected within each unit in order to avoid targeting specific AMR profiles, bacterial species, or sample source types using a common protocol (17). Only those from the 33 countries that were also represented in the sewage data set (Table S1) were included. In total, 2,989 clinical bacterial isolates were analyzed. Metadata handling, sample collection, DNA extraction, sequencing, *de novo* assembly of genomes, and bioinformatic species identification are described in Nag et al. (17).

The TWIW data set variables “anatomical_origin” and “other_source_indicator” were defined only for a subset of isolates, while “type” served as the primary descriptor. 96% of the isolates were assigned one of the 18 descriptive values, while just 4% defined as “None” or “unspecified diagnostic sample.” The 20 sample categories under “type” were too numerous for effective visualization, and many lacked sufficient anatomical information on their own. To improve clarity, we recoded the sample types into six simplified and more informative categories while preserving the major existing groups (see Table S3).

The bioinformatically inferred “concluding_id” column was used for bacterial species identification, as it was validated using multiple methods (17). Fewer than 0.2% of isolates lacked species annotation. For visualization purposes, we recoded all bacterial species outside the top 10 as “Unknown/Other.” The composition of the clinical isolate data set is visualized in Fig. S15. The ranking was calculated based on the total number of gene detections per species. ENA accession numbers for all clinical isolates and more details are provided in Table S4. Clinical isolate assemblies were used for detection and analysis of both ARG variants and clusters (90% homology).

### Reference database

We focused only on mobile/acquired ARGs, as these are not tied to a specific bacterial species. We did not include ARGs for which resistance depends on the presence of specific point mutations. The ARGs in the data set were detected and identified using the expert-curated database ResFinder (24). (downloaded 20 January 2023), which contains reference sequences for 3,180 acquired ARGs across various resistance classes.

### Detection of ARGs in assemblies with Flankophile

ARGs were detected in *de novo* assemblies of sewage metagenomes and clinical bacterial isolates with the Flankophile bioinformatics pipeline (25), version 0.2.10. Flankophile detected ARGs in assemblies using thresholds of ≥98% sequence identity and ≥98% coverage relative to the reference sequences. This allowed minor genetic variation while preserving gene function. Each ARG hit was assigned to the best-matching reference gene. Flankophile also identified and reported the gene variant of each detected ARG. Each unique DNA sequence was defined as a variant.

### ARG homology clusters

The reference database ResFinder is a redundant database containing many similar sequences. In addition to analyzing the ARG variants, we analyzed the ARGs as clusters, where ARGs with similar reference gene DNA sequences were grouped together. To define the groups, we used the tool USEARCH (26) to cluster the reference database using a threshold of 90% identity and 90% coverage for both target and query.

### Analysis of Flankophile output in R

The Flankophile output file, 1_hits_all.tsv, contained detailed information on all ARGs detected in each sample including the ARG variants identified. It was analyzed using R (version 4.5.1) along with additional sample metadata. For analyses of ARG variants, only those detected at least two times were included to reduce the risk of including sequencing or assembly artifacts.

RStudio version 2023.9.1.494 was used with the following packages: Tidyverse version 2.0.0 (for general data handling and analysis), eulerr version 7.0.2 (for Venn diagram visualization), patchwork version 1.1.3 (for figure combinations), and maps version 3.4.2 (for world map visualization).

### MDS and UMAP based on ARG variants

ARG variant detection data were stratified by country, and a presence/absence table was generated for each variant in either clinical isolates or sewage metagenomes. Classical multidimensional scaling (MDS) and uniform manifold approximation and projection (UMAP) were used to visualize the differences in ARG detected in clinical isolates and sewage metagenomes in countries from different regions. The analyses incorporated all ARG variants that were detected at least two times. The cmdscale function from R stats was used for MDS. R package umap version 0.2.10.0 was used with settings n_components = 2 and random_state = 15.

### Hierarchical clustering of ARG variant detection data

The R package pheatmap (version 1.0.13) was used for hierarchical clustering of country-wise ARG variant detection data from sewage and clinical isolates. Only ARG variants detected in at least 5 out of 33 countries (counting both sewage and clinical detections) were included, so they represented common and widespread variants.

### Read-based ARG detection and abundance in sewage metagenomes

Sewage mapstat files were downloaded from https://zenodo.org/records/14652833 (27). Details of the protocol can be found in the original study (15) and in the paper describing the pipeline ARGprofiler (23) which produced the results. The file panres_counts.csv contained the KMA (28) results for mapping and alignment of the sewage metagenome reads and fragments against the ARG reference database.

The mapstat file kingdom_and motus_agg_counts.csv contained the microbiome profiling data, the number of fragments within each sewage metagenome that aligned to bacteria in the mOTUs3 database (29). We calculated the abundance as fragments per kilobase reference per million bacterial fragments (FPKM) using the formula

.


$$relative\;abundance=\frac{ARG\;fragments}{ARG\;length\times bacterial\;depth}\times 10^{9}$$

We chose the threshold for detection of an ARG to be min. 60% coverage of the reference gene and min. 90% identity compared to the reference gene within a sewage metagenome. This is identical to default ResFinder Tool (24) settings. When any ARG within a cluster reached the detection threshold, we counted the ARG cluster detected.

### Selection of subsets of widespread clinical ARGs

Two subsets of clinically widespread ARGs (90% homology clusters) were selected to assess their prevalence in different bacterial species and sample types (Fig. 3). ARGs were required to have been detected in ≥15 clinical isolates (≥0.5% of all isolates) across a minimum of three countries. ARGs with no detection in sewage were defined as the “clinical detection only” subset (*n* = 16), while those also detected in sewage samples across a minimum of three countries were defined as the “detection in both” subset (*n* = 37). For the analysis, the bacterial species variable was reclassified to include the nine most prevalent species based on ARG hit frequency and the rest as “Unknown/Other.”

### Permutation test

We observed 350 instances where the same ARG variant was detected in both sewage and clinical isolates from the same country. We defined this as the number of codetections, which we calculated by comparing the sewage detection matrix with the clinical detection matrix. We wanted to determine whether the sewage detection data contained any real information about clinical detections or if this number was what one could expect by chance from a shuffled version of the sewage data set with the same inherent properties. We performed a permutation test in which the clinical detection matrix was unaltered, but the sewage detection matrix was shuffled using an algorithm that kept constant the number of different countries in which each variant was detected and the number of different variants detected in each country. Across 1,000 permutations, none of the shuffled sewage data matrices produced a number of codetections equal to or higher than 350, yielding a one-tailed permutation *P* value of 0.001 (Fig. S4). We used the R package RaschSampler–MCMC based sampling of binary matrices with fixed margins (30). The version of the package was version 0.8-10. We used the function rsampler with the following settings: burn_in = 1000, n_eff = 1000, step=1000, seed = 0, and tfixed = FALSE.

### Correlation of sewage abundance to clinical detection percentage

We calculated the correlation between the proportion of clinical isolates with the detection of an ARG and the corresponding abundance (FPKM) in sewage. We included ARGs that were detected in at least 5% of clinical isolates in at least one country. Each data point in the analysis represented one ARG (90% homology clusters) and one country, and data points were only included if the ARG was detected both clinically and in sewage. For each ARG and country, we calculated the geometric mean of the abundance across sewage metagenomes that had reads that mapped to the ARG. Spearman’s rho and two-sided *P* values were calculated using the R stats function cor.test. Adjusted *P* values (*q* values) were calculated using the R stats function p.adjust. For correlation analysis of individual ARGs, we included the 28 ARGs with data points from at least 10 countries to ensure the quality of the analysis. The same criterion was applied to the analyses by AMR class.

## SUPPLEMENTAL MATERIAL

Supplemental Figures msystems.00031-26-s0001.docx Figures S1 to S15.

Supplemental Tables msystems.00031-26-s0002.docx Tables S1 to S5.

ASM does not own the copyrights to Supplemental Material that may be linked to, or accessed through, an article. The authors have granted ASM a non-exclusive, world-wide license to publish the Supplemental Material files. Please contact the corresponding author directly for reuse.
