# Supplementary material for: Urban sewage resistomes partially reflect clinical resistomes
Source: mSystems. 2026 Mar 13;11(4):e00031-26. doi: 10.1128/msystems.00031-26 (PMC13098265; doi:10.1128/msystems.00031-26)
Supplement: Supplemental Figures — Figures S1 to S15. [file msystems.00031-26-s0001.docx]

Supplemental figures

***
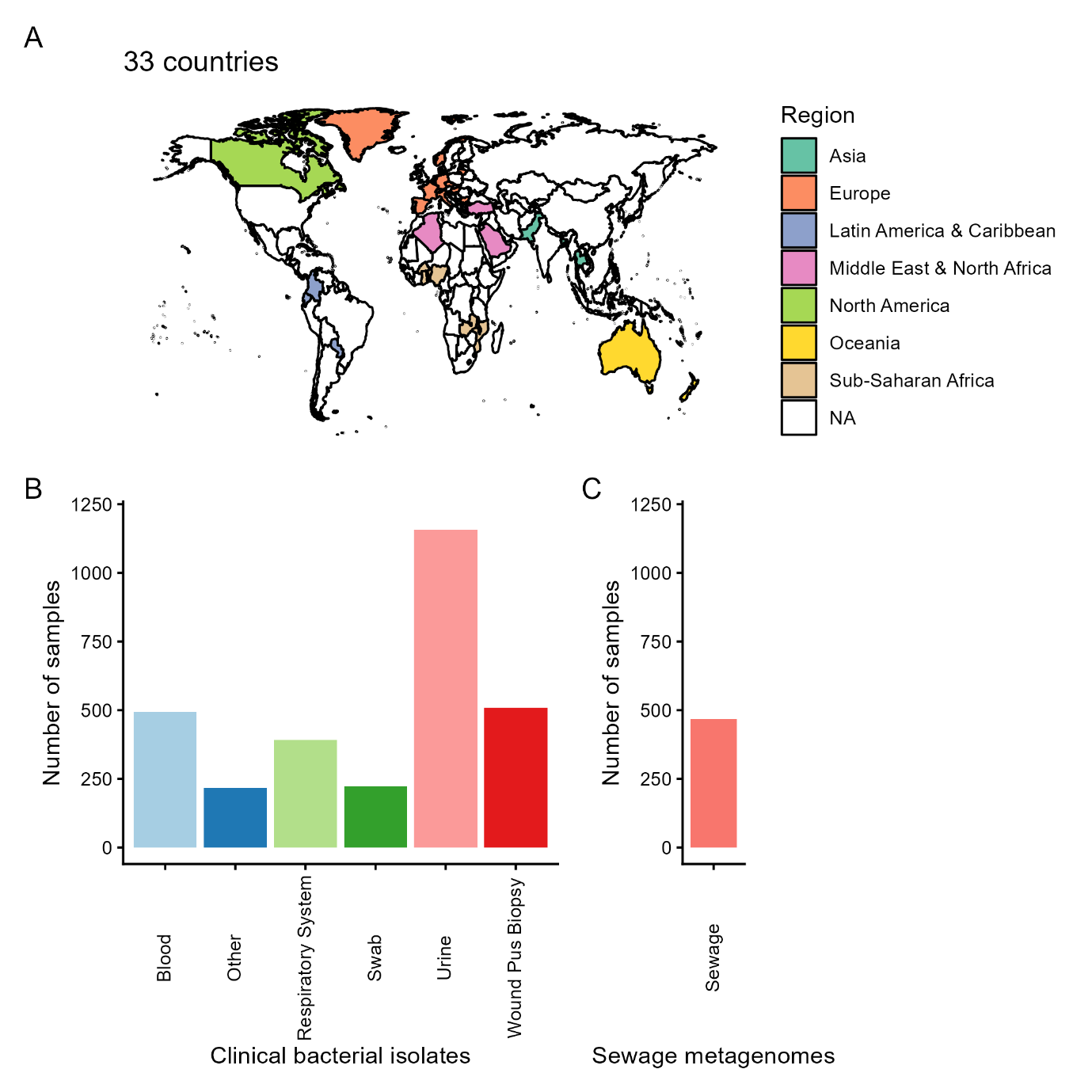
***

***Fig S1*** *A) 33 countries of origin for the samples used in the study. B) Number of clinical bacterial isolates by sample type analyzed in the study. C) Number of sewage metagenomes analyzed in the study.*

***
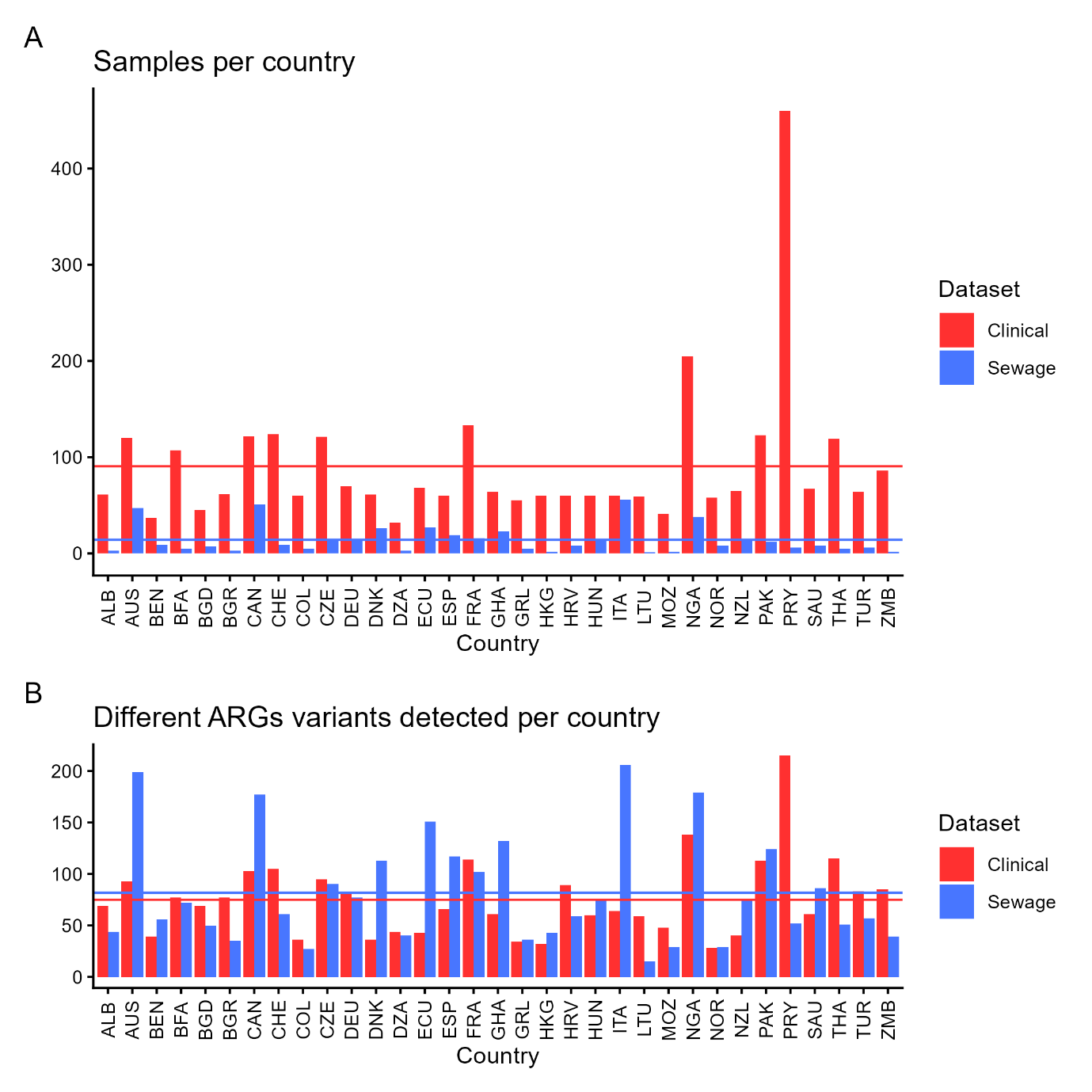
***

***Fig S2*** *(A) Number of clinical isolates (red) and sewage metagenomes (blue) analyzed per country. (B) Number of different ARG variants detected per country in clinical isolates (red) and sewage metagenomes (blue). The horizontal line represents the average across all countries.*


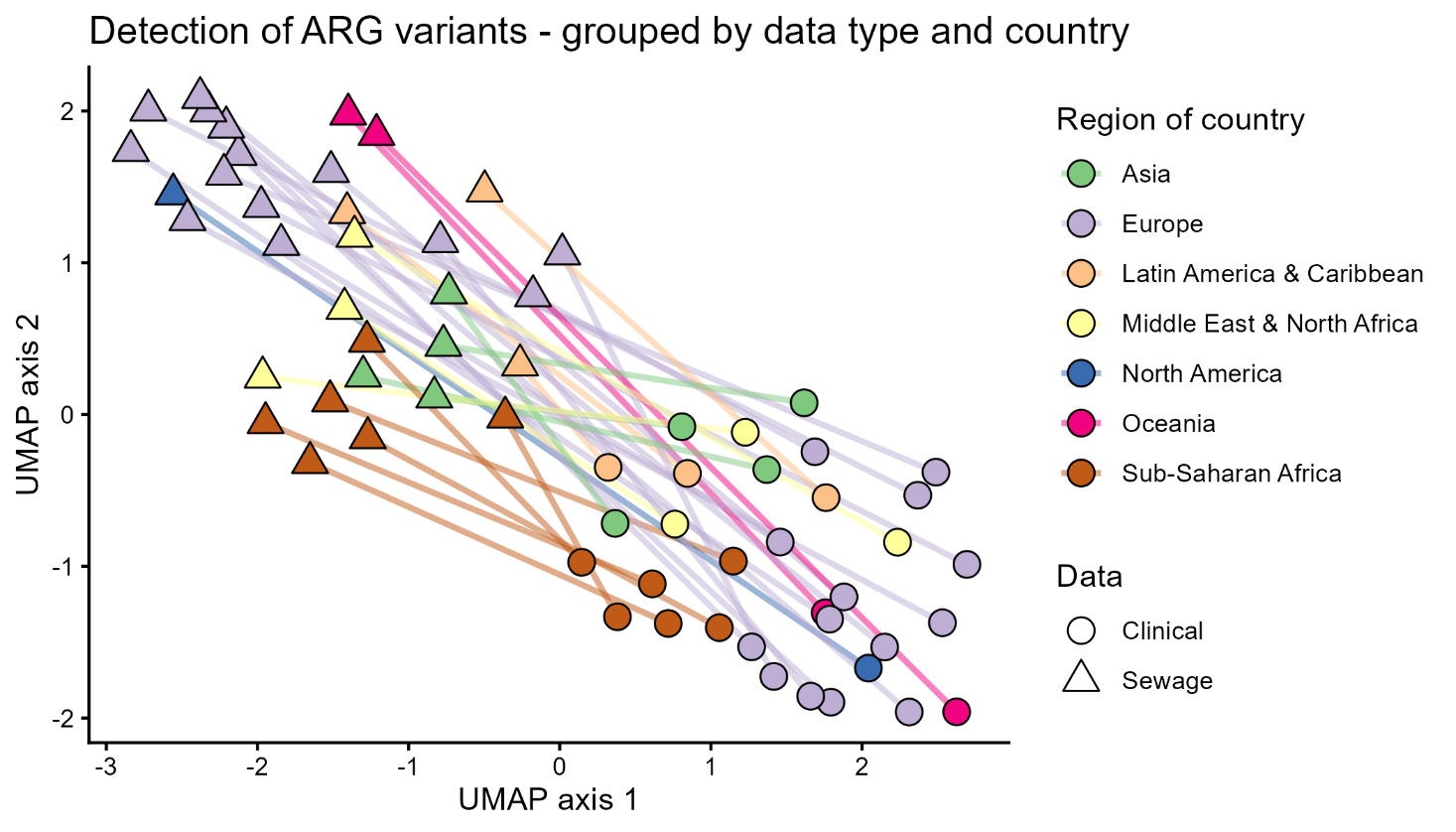


***Fig S3*** *UMAP dimensionality reduction of ARG variant detection data for each country. Every point represents either clinical (circle) or sewage (triangle) detection data. The color indicates the world region, and lines connect sewage and clinical data points for the same country.*


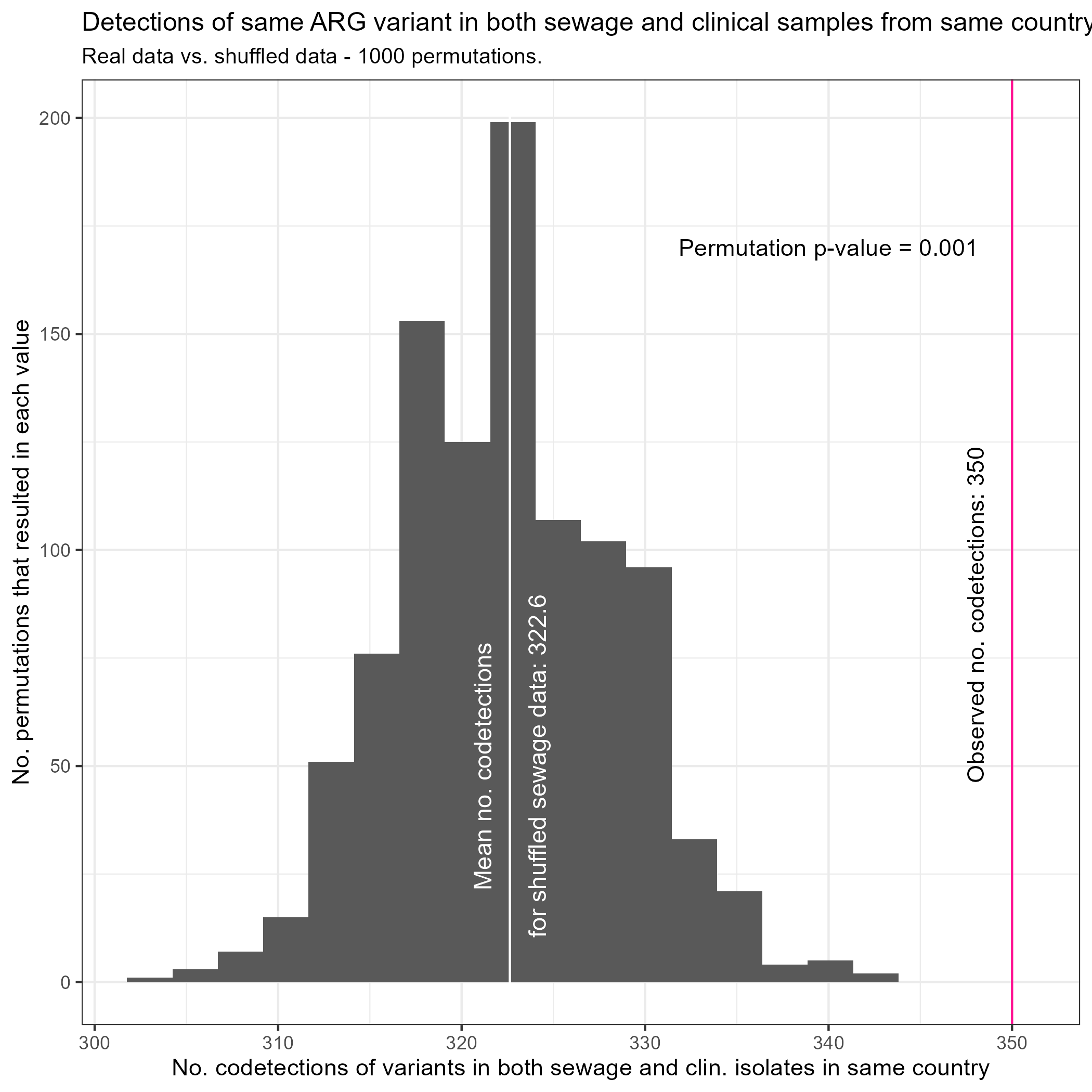


***Fig S4*** *Frequency distribution of the number of codetections when country-wise ARG variant detection data from sewage was randomized—1000 permutations. The number of codetections was defined as cases where the same ARG variant was detected in both sewage and clinical isolates from the same country. None of the permutations resulted in a number of codetections equal to or higher than the real number of codetections (red line). One-sided permutation p-value: 0.001.*

***
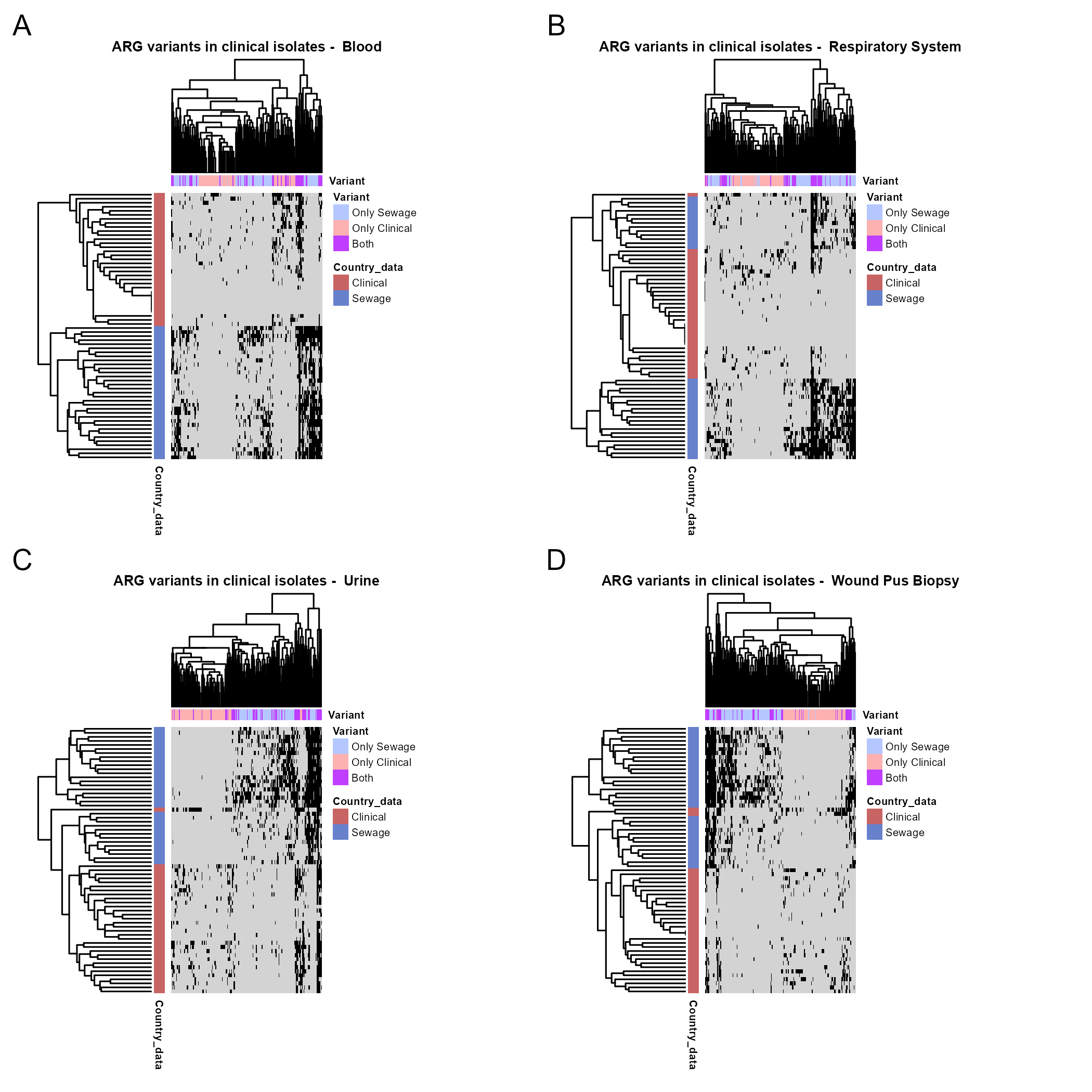
***

***Fig S5*** *Hierarchical clustering of ARG variant detection data grouped by country. Blue rows represent detection data from sewage, and red rows represent detection in clinical isolates of the four most common isolate types in the clinical isolate dataset: "Blood," "Respiratory System," "Urine," or "Wound Pus Biopsy." Column color indicates whether the ARG variant was detected only in sewage (light blue), only in clinical isolates (pale pink), or in both (purple). Black color represents ARG detection in the heatmap.*

***
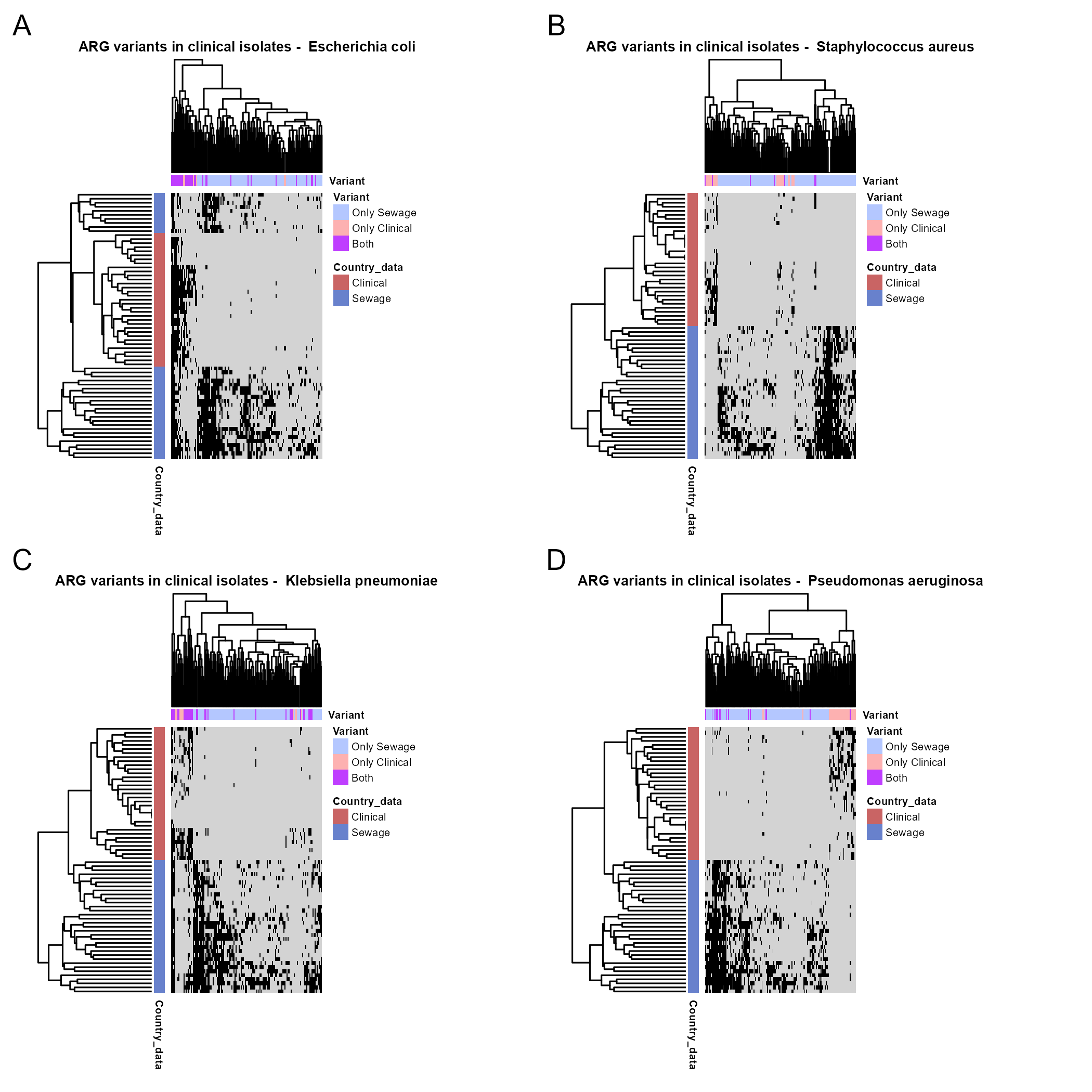
***

***Fig S6*** *Hierarchical clustering of ARG variant detection data grouped by country. Blue rows represent detection data from sewage, and red rows represent detection in clinical isolates of the four most common bacterial species in the clinical isolate dataset: "Escherichia coli," "Staphylococcus aureus," "Klebsiella pneumoniae," or "Pseudomonas aeruginosa." Column color indicates whether the ARG variant was detected only in sewage (light blue), only in clinical isolates (pale pink), or in both (purple). Black color represents ARG detection in the heatmap.*


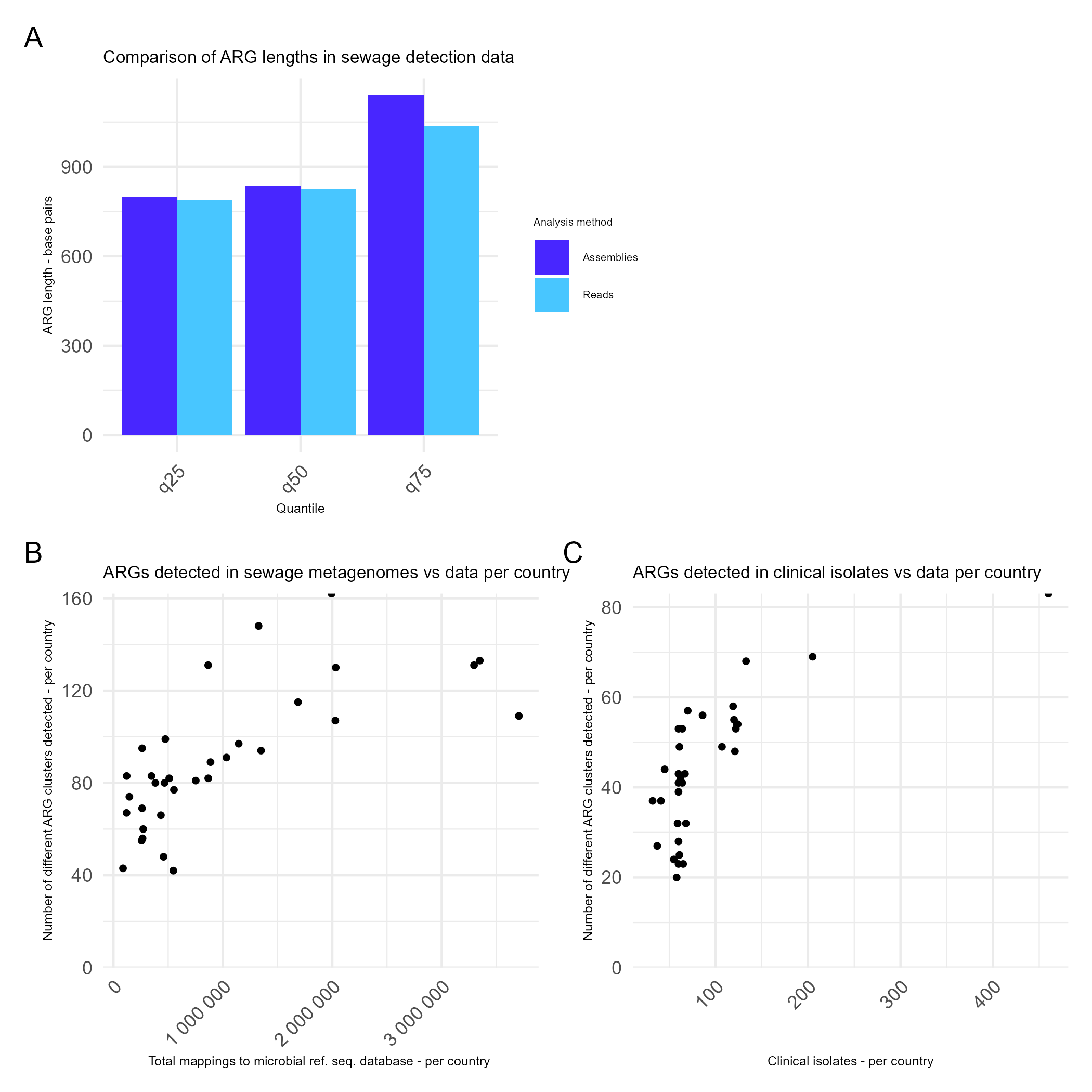


***Fig S7*** *(A) Comparison of the length (in base pairs) of ARGs detected in sewage by an assembly-based method (dark blue) and a read-based method (light blue). (B) Number of different ARGs detected per country in sewage (y-axis) versus the total number of reads mapped to bacteria in sewage metagenomes from that country (x-axis). (C) Number of different ARGs detected per country in clinical isolates (y-axis) versus the total number of clinical isolates analyzed from that country (x-axis).*


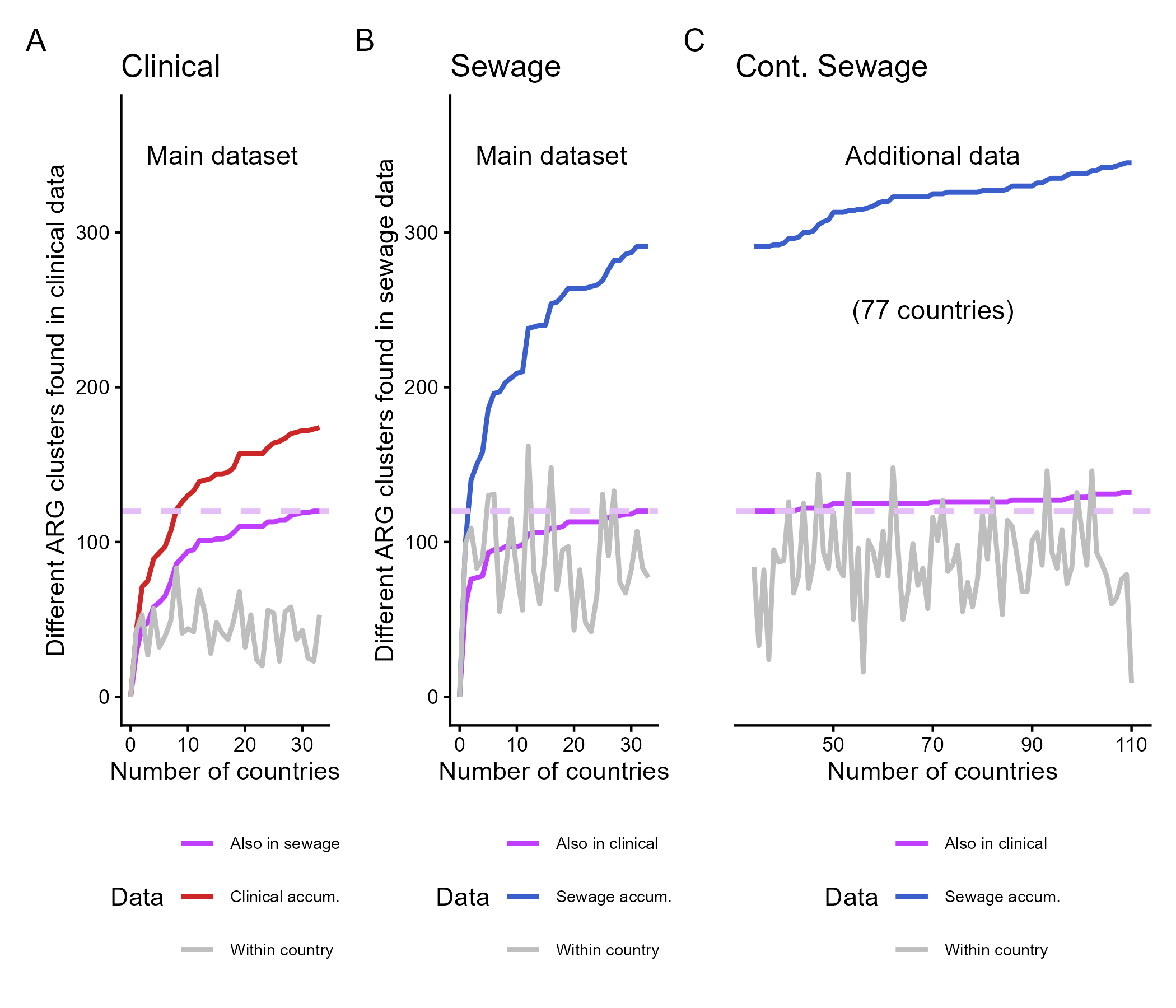


***Fig S8*** *Rarefaction analysis of the number of ARGs (90% homology clusters) detected in clinical or sewage data when including samples from more countries. The y-axis shows the number of different ARGs detected. The x-axis depicts the number of countries included. The order of inclusion is random but kept the same for the 33 countries in plots A and B. The gray line indicates the number of different ARGs detected within each country included. The dashed purple line represents the number of ARGs (n = 120) that were detected in both sewage and clinical isolates from any of the 33 countries. (A) ARGs detected in clinical bacterial isolates from 0 to 33 countries. The red line shows the cumulative number of different ARGs detected, and the purple line shows how many of those had also been detected in sewage samples from any of the 33 countries. (B) ARGs detected in sewage metagenomes from 0 to 33 countries. The blue line shows the cumulative number of different ARGs detected, and the purple line shows how many of those had also been detected in clinical isolates from any of the 33 countries. (C) Continuation of rarefaction in plot B by inclusion of sewage metagenomes from 77 additional countries.*

***
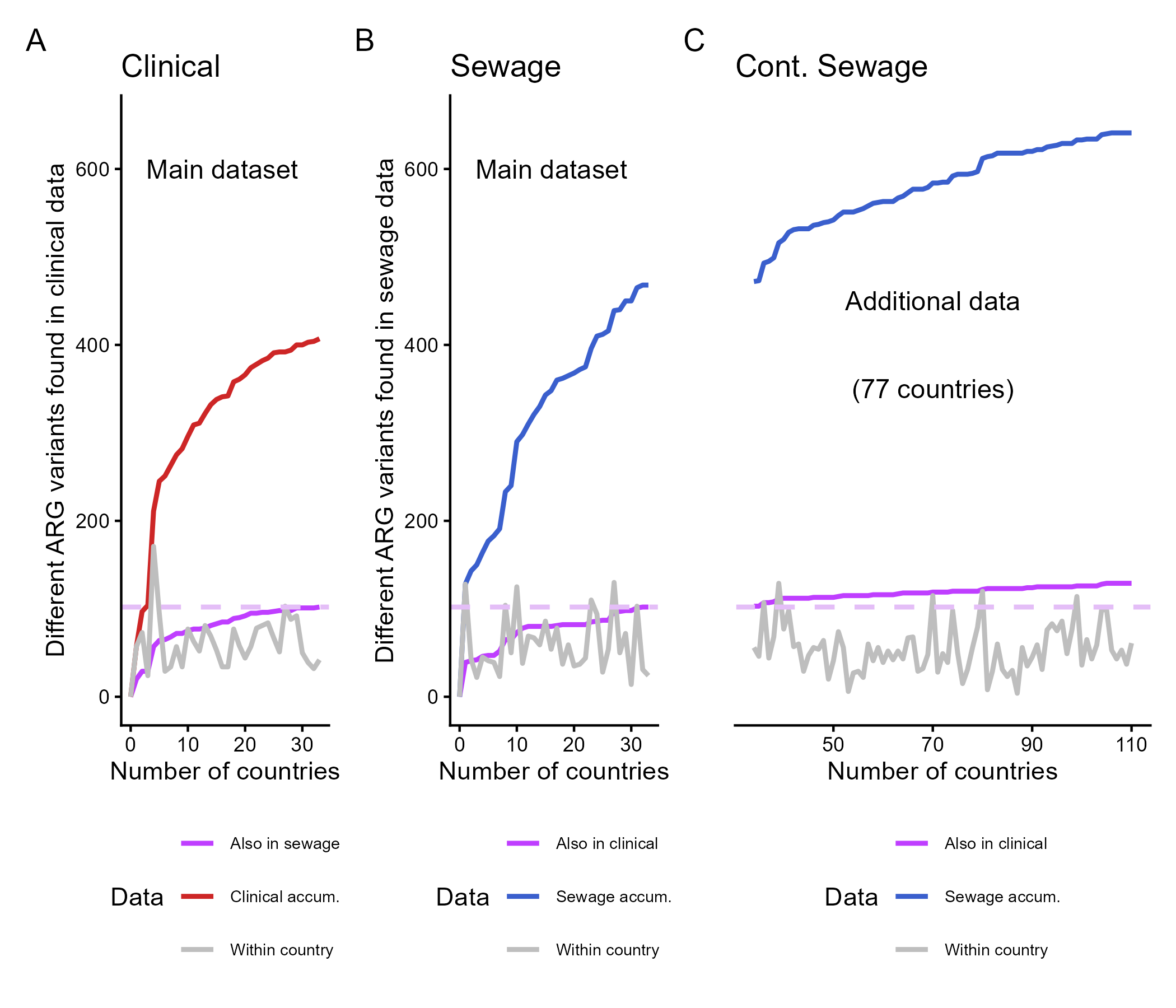
***

***Fig S9*** *Rarefaction analysis of the number of ARGs variants detected in clinical or sewage data when including samples from more countries. The y-axis shows the number of different ARG variants detected. The x-axis depicts the number of countries included. The order of inclusion is random but kept the same for the 33 countries in plots A and B. The gray line indicates the number of different ARG variants detected within each country included. The dashed purple line represents the number of ARG variants that were detected in both sewage and clinical isolates from any of the 33 countries. (A) ARG variants detected in clinical bacterial isolates from 0 to 33 countries. The red line shows the cumulative number of different ARG variants detected, and the purple line shows how many of those had also been detected in sewage samples from any of the 33 countries. (B) ARG variants detected in sewage metagenomes from 0 to 33 countries. The blue line shows the cumulative number of different ARG variants detected, and the purple line shows how many of those had also been detected in clinical isolates from any of the 33 countries. (C) Continuation of rarefaction in plot B by inclusion of sewage metagenomes from 77 additional countries.*


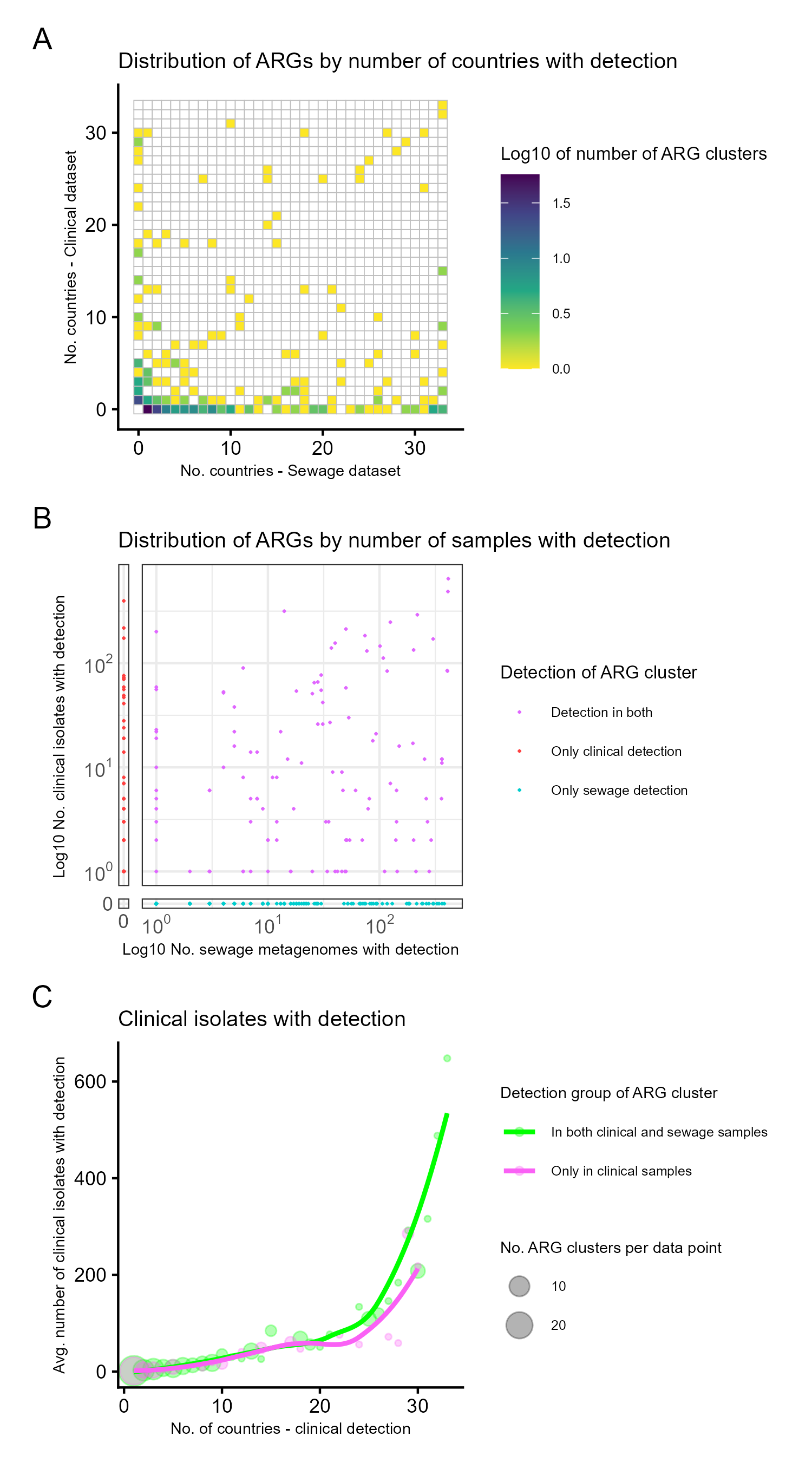


***Fig S10*** *Detection of ARGs (90% homology clusters) in sewage and clinical isolates. (A) Distribution of ARGs by the number of countries in which they were detected via sewage metagenomes (x-axis) and clinical isolates (y-axis). Color represents the log₁₀-transformed counts of ARGs. White cells indicate no ARGs. (B) Distribution of ARGs by the number of samples in which they were detected—sewage metagenomes (x-axis) and clinical isolates (y-axis). The axes are log₁₀-transformed. Each point represents one ARG, and the color indicates where it was detected. (C) Comparison of the average number of clinical isolates with ARG detection between ARGs detected exclusively in clinical isolates and those also detected in sewage metagenomes. Averages are calculated by grouping ARGs based on the number of countries in the clinical dataset in which they were detected. The x-axis shows the number of countries with ARG detection in clinical data. The y-axis shows the average number of clinical isolates with detection for those ARGs. Point size indicates the number of ARGs in each group. Regression lines are made from the grouped averages. The clinical regression line (pink) is shorter than the one for sewage (green) since there was no clinical data for x-values above 30.*

***
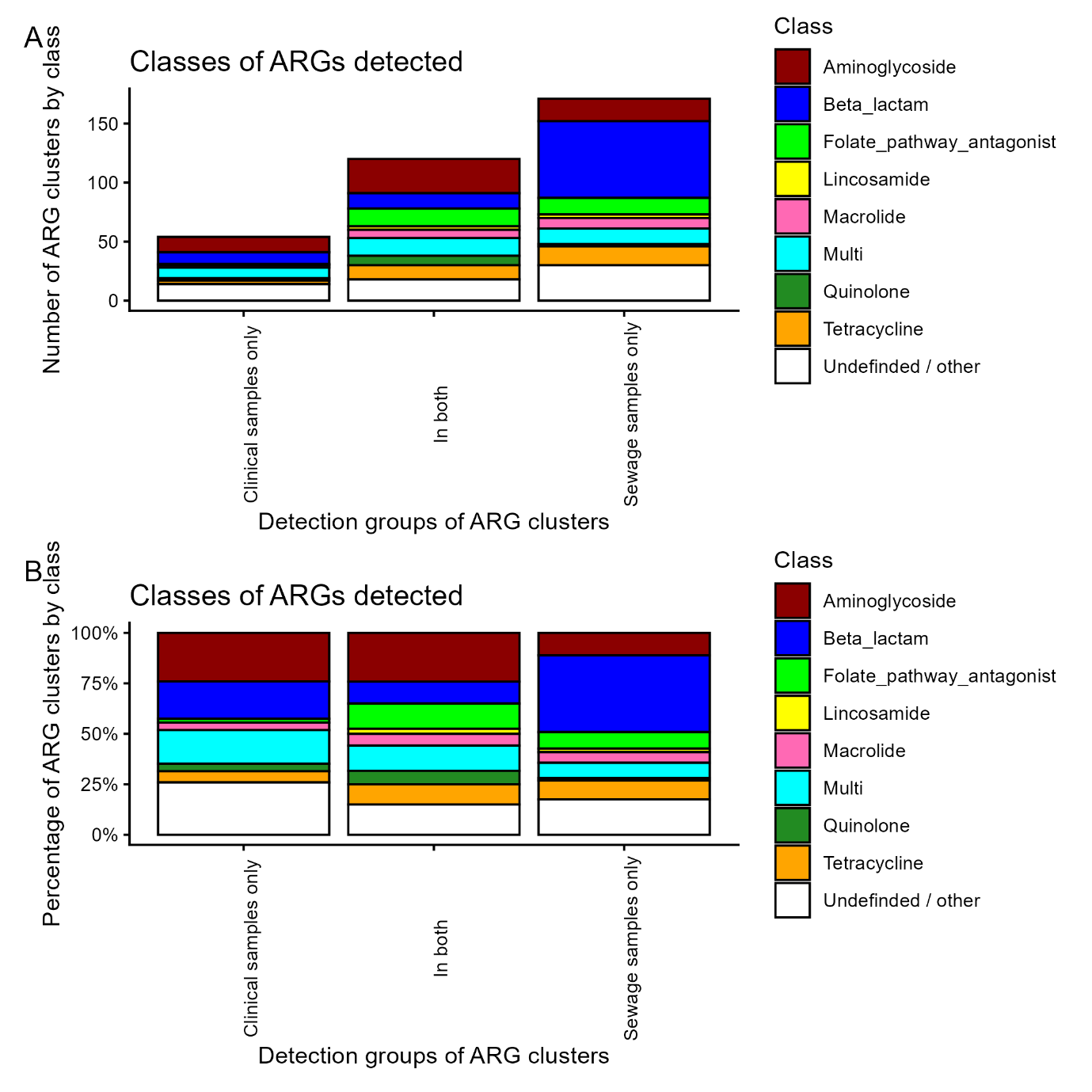
***

***Fig S11*** *Distribution of detected ARGs (90% homology clusters) across AMR classes in samples from 33 countries. ARGs were categorized by detection in clinical samples only, sewage samples only, or both. (A) Counts of ARGs in each category. (B) Percentage of ARGs in each category.*

*
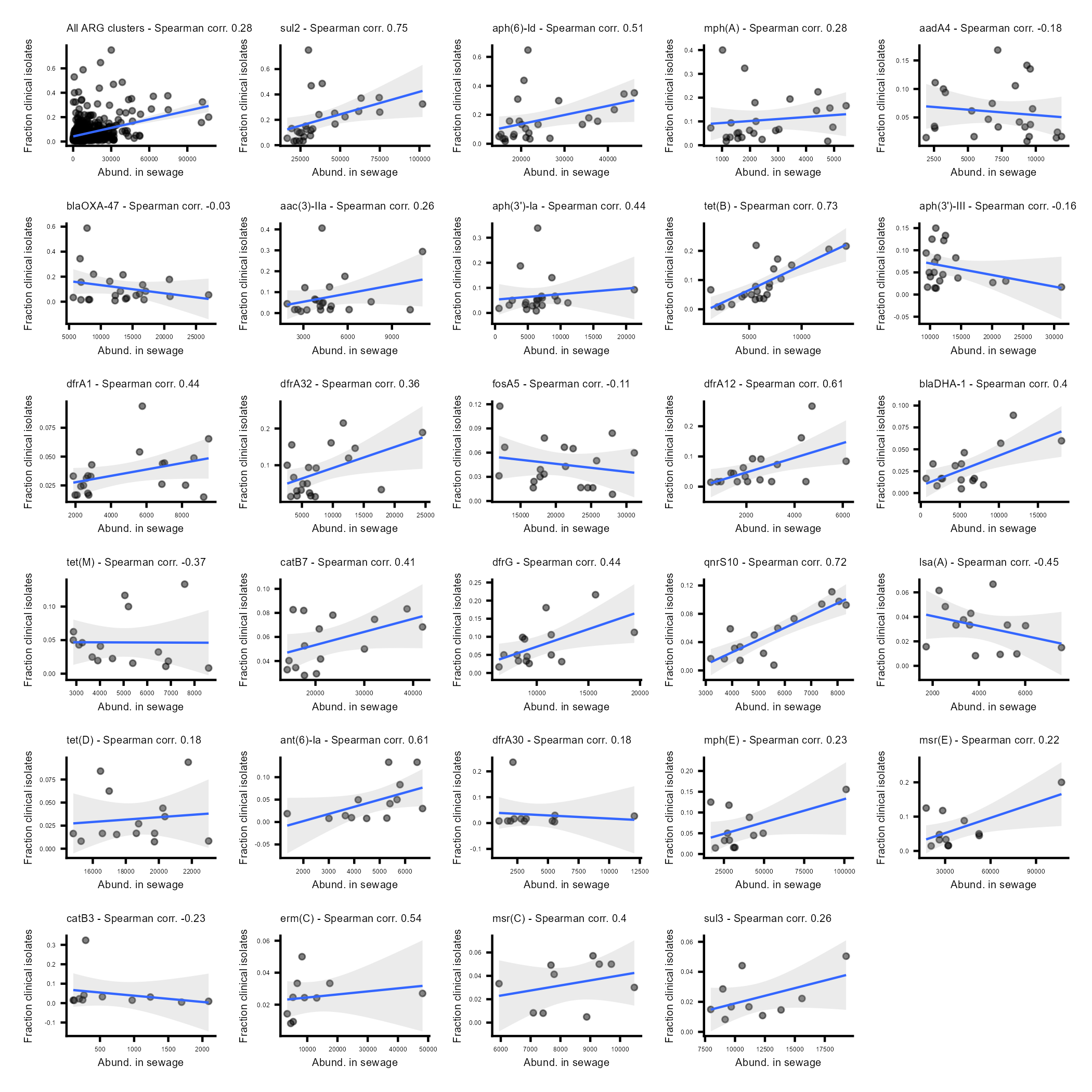
*

***Fig S12*** *Correlation of country-wise clinical isolate prevalence and sewage abundance of ARGs (90% homology clusters). Each point represents data from one country, with sewage abundance on the x-axis and the fraction of clinical isolates with detection on the y-axis, and a linear regression line (blue). The first plot represents data from all ARGs combined, while the rest represent one ARG cluster each.*

*
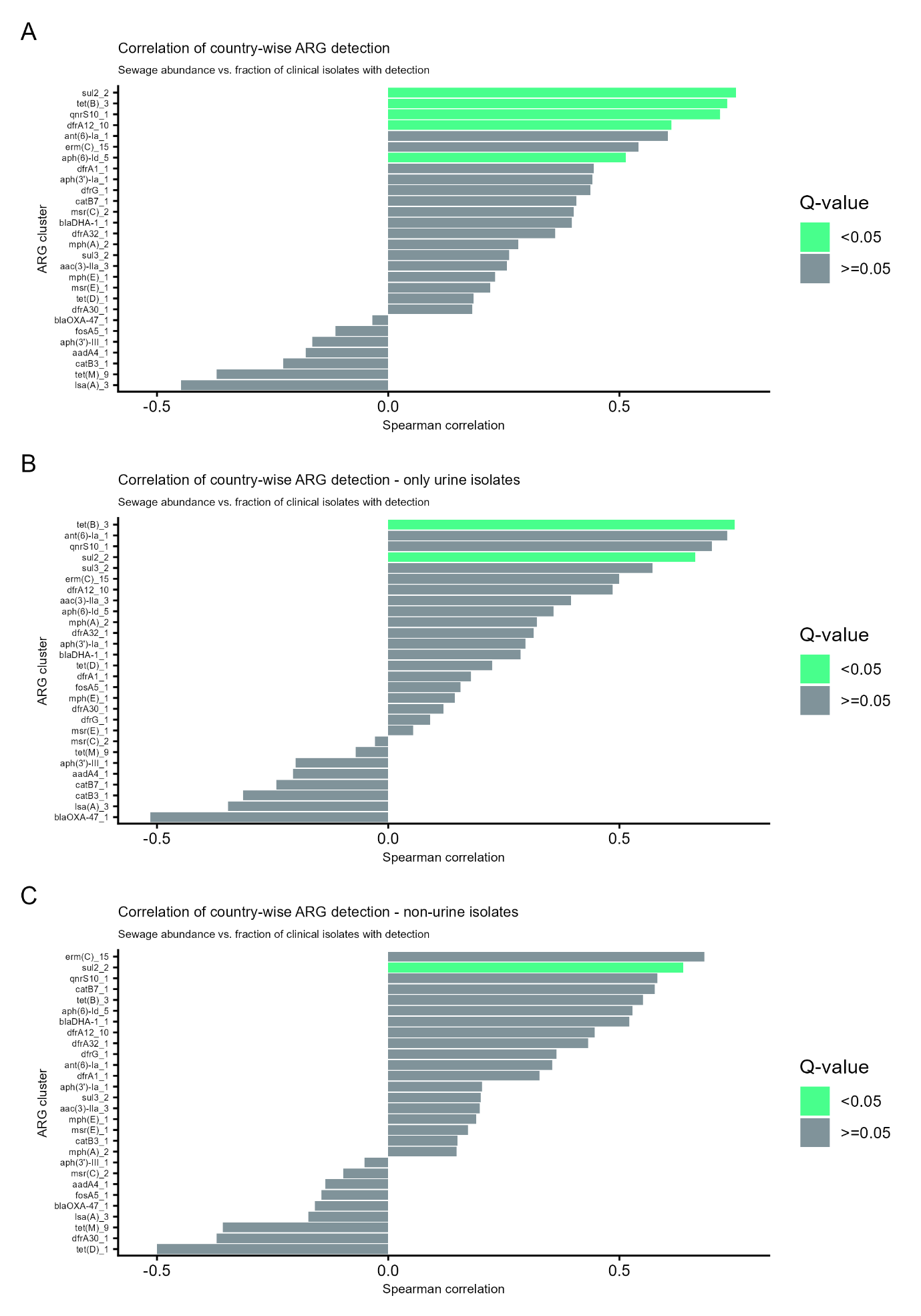
*

***Fig S13*** *Correlation analysis results with split clinical isolate dataset. (A) All clinical isolate types were included; a copy of Fig. 4F. (B) Only clinical isolates of urine origin were included in the clinical isolate dataset. (C) Only clinical isolates not of urine origin were included in the clinical isolate dataset. More details in the legend of Fig. 4.*


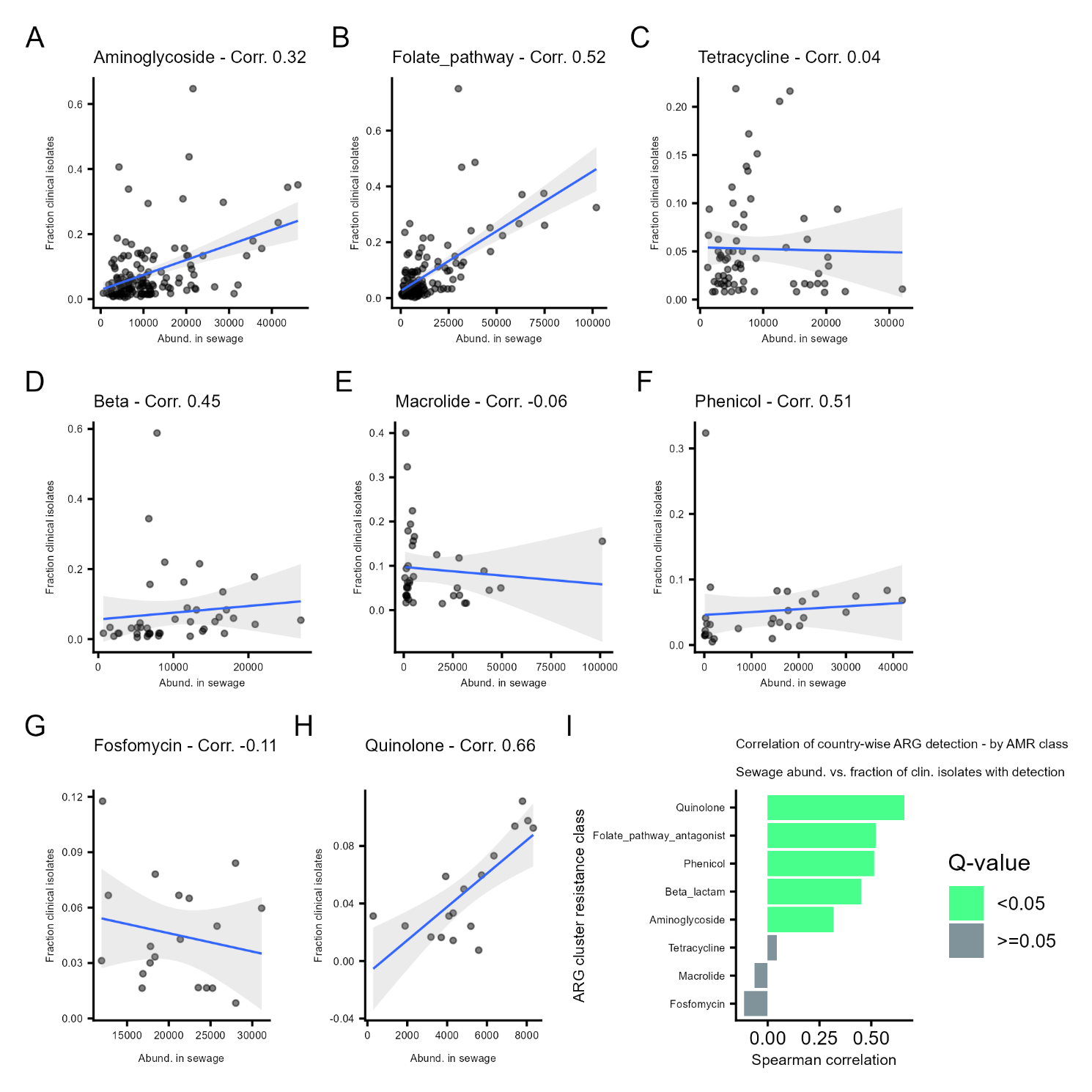


***Fig S14*** *Correlation of country-wise clinical isolate prevalence and sewage abundance of ARGs grouped by resistance class. (A–H) Correlation plots where each point represents data from one country, with sewage abundance on the x-axis and the fraction of clinical isolates with detection on the y-axis, and a linear regression line (blue). (I) Comparison of Spearman’s rho between AMR classes. Q-values (adjusted p-values) below 0.05 are marked with a green bar.*

***
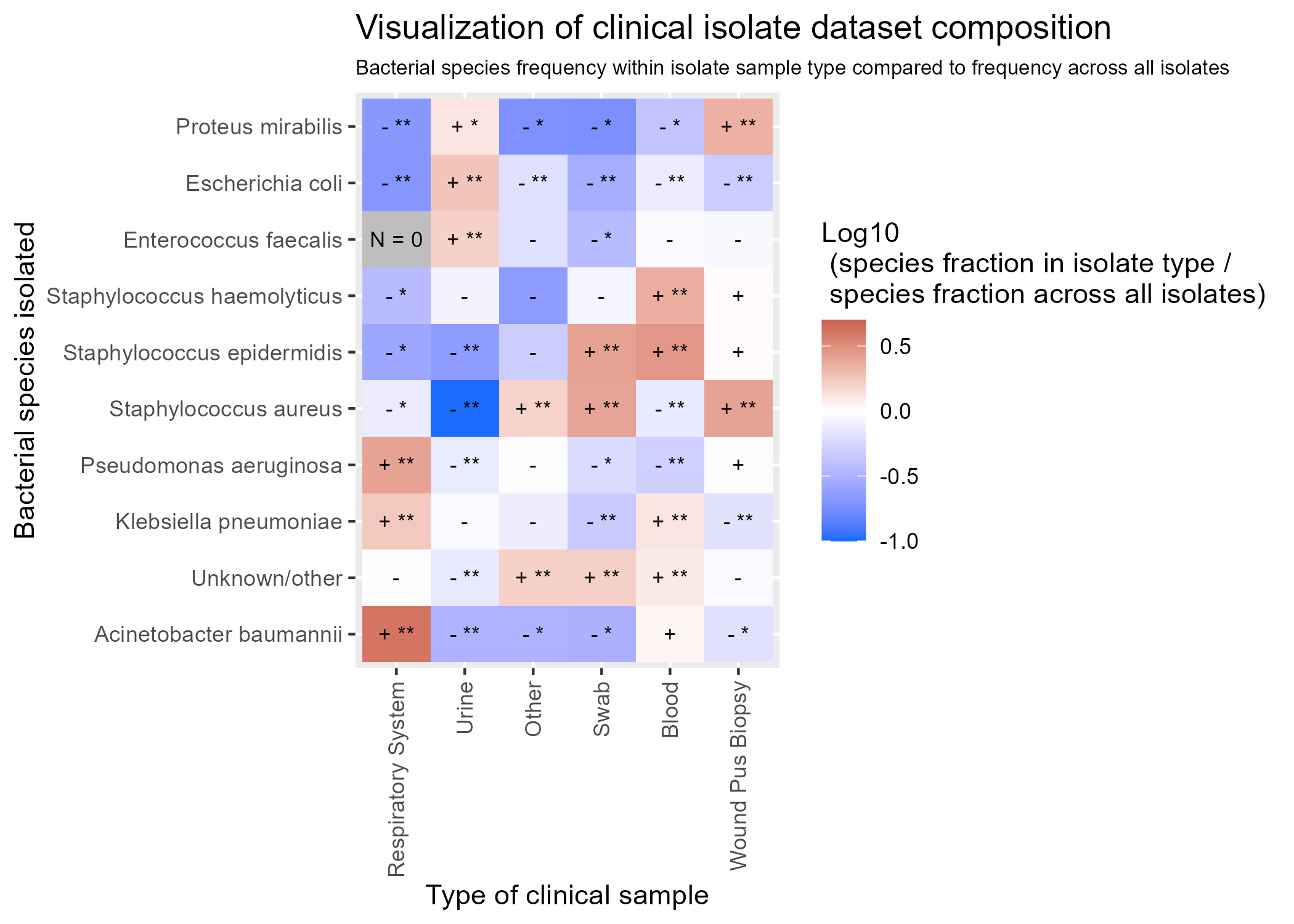
***

***Fig S15*** *Visualization of the composition of the clinical isolate dataset and the frequencies of different combinations of clinical sample types and bacterial species. The heatmap tile colors show whether isolates of a bacterial species are more (red) or less (blue) frequent among isolates of a certain type than they would have been in a hypothetical case where the frequency of each bacterial species was the same across all isolate types. The tile color represents the log₁₀ of the ratio between the fraction of isolates with each combination of isolate type and bacterial species and the fraction under a uniform distribution. A uniform distribution is not expected. Whether the increase or decrease in the number of isolates from each species and type combination compared to a uniform distribution was significant was calculated using Fisher’s exact test. A single asterisk (*) indicates significance at the 0.05 level, and a double asterisk (**) indicates significance at the 0.01 level.*
