# Supplementary material for: Urban sewage resistomes partially reflect clinical resistomes
Source: mSystems. 2026 Mar 13;11(4):e00031-26. doi: 10.1128/msystems.00031-26 (PMC13098265; doi:10.1128/msystems.00031-26)
Supplement: Supplemental Tables — Tables S1 to S5. [file msystems.00031-26-s0002.docx]

Supplemental tables

Urban sewage resistomes partially reflect clinical resistomes

Alix Vincent Thorn, Christian Brinch, Frank M. Aarestrup & Patrick Munk

# Table S1

List of the 33 countries from which the samples of the main dataset originated.

| Albania | ALB |
| --- | --- |
| Australia | AUS |
| Bulgaria | BGR |
| Canada | CAN |
| Switzerland | CHE |
| Czech Republic | CZE |
| Germany | DEU |
| Denmark | DNK |
| Ecuador | ECU |
| Spain | ESP |
| Ghana | GHA |
| Croatia | HRV |
| Hungary | HUN |
| Italy | ITA |
| Nigeria | NGA |
| Norway | NOR |
| New Zealand | NZL |
| Pakistan | PAK |
| Turkey | TUR |
| Zambia | ZMB |
| Burkina Faso | BFA |
| Bangladesh | BGD |
| France | FRA |
| Hong Kong | HKG |
| Lithuania | LTU |
| Paraguay | PRY |
| Saudi Arabia | SAU |
| Thailand | THA |
| Benin | BEN |
| Colombia | COL |
| Greenland | GRL |
| Mozambique | MOZ |
| Algeria | DZA |

# Table S2

List of the 77 additional countries for which only sewage metagenome data was available. The data was used for the rarefaction analysis only.

| Austria | AUT |
| --- | --- |
| Brazil | BRA |
| Botswana | BWA |
| China | CHN |
| Ivory Coast | CIV |
| Ethiopia | ETH |
| Finland | FIN |
| Georgia | GEO |
| Gambia | GMB |
| India | IND |
| Ireland | IRL |
| Iran | IRN |
| Iceland | ISL |
| Israel | ISR |
| Kazakhstan | KAZ |
| Kenya | KEN |
| Cambodia | KHM |
| Sri Lanka | LKA |
| Luxembourg | LUX |
| Latvia | LVA |
| Moldova | MDA |
| North Macedonia | MKD |
| Malta | MLT |
| Malaysia | MYS |
| Netherlands | NLD |
| Nepal | NPL |
| Peru | PER |
| Poland | POL |
| Kosovo | RKS |
| Senegal | SEN |
| Singapore | SGP |
| Serbia | SRB |
| Slovakia | SVK |
| Slovenia | SVN |
| Sweden | SWE |
| Togo | TGO |
| Tanzania | TZA |
| USA | USA |
| Vietnam | VNM |
| South Africa | ZAF |
| Bosnia and Herzegovina | BIH |
| Barbados | BRB |
| Cameroon | CMR |
| Estonia | EST |
| UK | GBR |
| Greece | GRC |
| Guatemala | GTM |
| Japan | JPN |
| South Korea | KOR |
| St. Lucia | LCA |
| Morocco | MAR |
| Montenegro | MNE |
| Mauritius | MUS |
| Malawi | MWI |
| Nicaragua | NIC |
| Philippines | PHL |
| Portugal | PRT |
| Chad | TCD |
| Taiwan | TWN |
| Uganda | UGA |
| Uruguay | URY |
| United Arab Emirates | ARE |
| Argentina | ARG |
| Belgium | BEL |
| Chile | CHL |
| Kuwait | KWT |
| Madagascar | MDG |
| Bolivia | BOL |
| Democratic Republic of the Congo | COD |
| Mexico | MEX |
| Trinidad and Tobago | TTO |
| Bahrain | BHR |
| Comoros | COM |
| Costa Rica | CRI |
| Egypt | EGY |
| Lebanon | LBN |
| Iraq | IRQ |

# Table S3

Recoding of the clinical isolate sample type variable for the Two Weeks in the World 2020 clinical bacterial isolate dataset from the 33 countries listed in Table S1.

| Original sample type | Recoding |
| --- | --- |
| urine | All 1156 isolates from type “urine” were recoded as “Urine” |
| blood | All 493 isolates from type “blood” were recoded as “Blood” |
| swab (wound) | All 345 isolates from type “swab (wound)” were recoded as "Wound Pus Biopsy" |
| aspirate | Of 216 isolates of type “aspirate” the majority 167 were from the respiratory system since they had “anatomical_origin” noted as "trachea" or "bronchi/alveoli" or "respiratory tract". Those were recoded as “Respiratory System” and the remaining isolates as “Other” |
| sputum | All 182 isolates from type “sputum” were recoded as “Respiratory System” |
| swab (cavity) | 136 isolates of “swab (cavity)” type came from many different anatomical_region variables. Some of these had anatomical_origin categories that also overlapped with those of type “swab (superficial and non-surgical)”. We decided to recode the 6 that had the “other_source_indicator” variable values “pus” or “abscess” as "Wound Pus Biopsy" and the remaining as “Swab” |
| unspecified diagnostic sample | 129 isolates with type “unspecified diagnostic sample”. 78 of them had the “other_source_indicator” variable values “pus” or “abscess”. These were recoded as "Wound Pus Biopsy" and the rest as “Other” |
| swab (superficial and non-surgical) | 89 isolates were of type “swab (superficial and non-surgical)”. Some of these had anatomical_origin categories that overlapped with “swab (cavity)”. We decided to recode the 11 that had “other_source_indicator” as “pus” or “abscess” as "Wound Pus Biopsy" and the remaining as “Swab”. |
| biopsy | 59 isolates had type “biopsy”. Those came from different anatomical regions. We decided to recode all as "Wound Pus Biopsy". |
| punctate | 40 isolates had type “punctate” and came from many different anatomical regions and also many different source indicators. We decided to recode the 4 with “other_source_indicator” “pus” or “abscess” to "Wound Pus Biopsy" and the rest to other. |
| faeces | Since we did not want too many categories we decided to recode all 40 “faeces” isolates to “Other” |
| lavage | 29 isolates were of type “lavage”. 28 of them had “anatomic_origin” “bronchi/alveoli” and were recoded as “Respiratory System” and the rest which as no “anatomic_origin” variable as other. |
| expectorate | 11 isolates of type “expectorate” were recoded as “Respiratory System”. |
| swab (unspecified) | 21 isolates were of type “swab (unspecified)”. 6 of them were “other_source_indicator” “pus” to "Wound Pus Biopsy" and the rest to “Swab”. |
| drainage | 17 isolates of type “drainage” and 7 of “type surgical sample” were of mixed origin and “other_source_indicator”. They were recoded as “Other”. |
| semen | 6 isolates of type “semen” were recoded as “Other”. |
| None | 5 isolates of type “None” were recoded as “Other”. |
| secretion | 4 isolates were of type “secretion”. 3 had “anatomical_origin” noted as "trachea" or "bronchi/alveoli" or "respiratory tract". Those were recoded as “Respiratory System” and the remaining isolates as “Other” |
| smear | 4 isolates of type “smear” were recoded as “Other” |

# Table S4

Metadata and ENA accession numbers for clinical isolate and sewage metagenome sequence data used in this study. ENA database is found at https://www.ebi.ac.uk/ena/browser/home.

| Country | Collection | Sample name | Sample type | Ena run acc |
| --- | --- | --- | --- | --- |
| ALB | 2016 | DTU_2016_1_1_MG_ALB-17 | Sewage | ERR2607373 |
| AUS | 2016 | DTU_2016_8_5_MG_AUS-18_4 | Sewage | ERR2607377 |
| AUS | 2016 | DTU_2016_11_3_MG_AUS-18a_2 | Sewage | ERR2607379 |
| AUT | 2016 | DTU_2016_14_1_MG_AUT-70 | Sewage | ERR2607387 |
| BGR | 2016 | DTU_2016_18_1_MG_BGR-66 | Sewage | ERR2607391 |
| BRA | 2016 | DTU_2016_22_1_MG_BRA-53 | Sewage | ERR2607397 |
| BRA | 2016 | DTU_2016_25_1_MG_BRA-53a | Sewage | ERR2607396 |
| BWA | 2016 | DTU_2016_28_1_MG_BWA-19 | Sewage | ERR2607401 |
| CAN | 2016 | DTU_2016_34_3_MG_CAN-22_2 | Sewage | ERR2607403 |
| CAN | 2016 | DTU_2016_37_3_MG_CAN-22a_2 | Sewage | ERR2607405 |
| CAN | 2016 | DTU_2016_44_5_MG_CAN-22b_4 | Sewage | ERR2607412 |
| CAN | 2016 | DTU_2016_45_1_MG_CAN-22c | Sewage | ERR2607414 |
| CHE | 2016 | DTU_2016_52_7_MG_CHE-67_6 | Sewage | ERR2607421 |
| CHN | 2016 | DTU_2016_53_1_MG_CHN-64 | Sewage | ERR2607423 |
| CIV | 2016 | DTU_2016_54_1_MG_CIV-13 | Sewage | ERR2607426 |
| CZE | 2016 | DTU_2016_58_1_MG_CZE-23 | Sewage | ERR2607430 |
| DEU | 2016 | DTU_2016_64_4_MG_DEU-27_3 | Sewage | ERR2607433 |
| DNK | 2016 | DTU_2016_66_1_MG_DNK_71_RL | Sewage | ERR2607438 |
| DNK | 2016 | DTU_2016_67_1_MG_DNK-71_RA | Sewage | ERR2607436 |
| DNK | 2016 | DTU_2016_68_1_MG_DNK-71_RD | Sewage | ERR2607437 |
| ECU | 2016 | DTU_2016_69_1_MG_ECU-14 | Sewage | ERR2607441 |
| ECU | 2016 | DTU_2016_70_1_MG_ECU-14a | Sewage | ERR2607440 |
| ESP | 2016 | DTU_2016_73_2_MG_ESP-75_1 | Sewage | ERR2607442 |
| ETH | 2016 | DTU_2016_78_1_MG_ETH-24 | Sewage | ERR2607448 |
| FIN | 2016 | DTU_2016_79_1_MG_FIN-25 | Sewage | ERR2607451 |
| GEO | 2016 | DTU_2016_82_1_MG_GEO-59 | Sewage | ERR2607455 |
| GHA | 2016 | DTU_2016_86_1_MG_GHA-4 | Sewage | ERR2607456 |
| GMB | 2016 | DTU_2016_87_1_MG_GMB-10 | Sewage | ERR2607457 |
| HRV | 2016 | DTU_2016_88_1_MG_HRV-68 | Sewage | ERR2607462 |
| HUN | 2016 | DTU_2016_93_1_MG_HUN-61 | Sewage | ERR2607463 |
| IND | 2016 | DTU_2016_94_1_MG_IND-11 | Sewage | ERR2607466 |
| IRL | 2016 | DTU_2016_97_1_MG_IRL-69 | Sewage | ERR2607469 |
| IRN | 2016 | DTU_2016_100_1_MG_IRN-12 | Sewage | ERR2607472 |
| ISL | 2016 | DTU_2016_103_1_MG_ISL-28 | Sewage | ERR2607475 |
| ISR | 2016 | DTU_2016_107_2_MG_ISR-29_1 | Sewage | ERR2607476 |
| ITA | 2016 | DTU_2016_109_1_MG_ITA-30 | Sewage | ERR2607479 |
| KAZ | 2016 | DTU_2016_110_1_MG_KAZ-6 | Sewage | ERR2607480 |
| KEN | 2016 | DTU_2016_111_1_MG_KEN-72 | Sewage | ERR2607484 |
| KHM | 2016 | DTU_2016_115_1_MG_KHM-21 | Sewage | ERR2607485 |
| LKA | 2016 | DTU_2016_116_1_MG_LKA-40 | Sewage | ERR2607486 |
| LUX | 2016 | DTU_2016_117_1_MG_LUX-32 | Sewage | ERR2607487 |
| LVA | 2016 | DTU_2016_118_1_MG_LVA-31 | Sewage | ERR2607491 |
| MDA | 2016 | DTU_2016_122_1_MG_MDA-65 | Sewage | ERR2607494 |
| MKD | 2016 | DTU_2016_128_4_MG_MKD-62_3 | Sewage | ERR2607497 |
| MLT | 2016 | DTU_2016_130_2_MG_MLT-63_1 | Sewage | ERR2607499 |
| MYS | 2016 | DTU_2016_135_4_MG_MYS-54_3 | Sewage | ERR2607504 |
| NGA | 2016 | DTU_2016_137_1_MG_NGA-50 | Sewage | ERR2607509 |
| NLD | 2016 | DTU_2016_143_4_MG_NLD-43_3 | Sewage | ERR2607512 |
| NOR | 2016 | DTU_2016_145_1_MG_NOR-34 | Sewage | ERR2607517 |
| NPL | 2016 | DTU_2016_148_1_MG_NPL-33 | Sewage | ERR2607518 |
| NZL | 2016 | DTU_2016_149_1_MG_NZL-56 | Sewage | ERR2607519 |
| PAK | 2016 | DTU_2016_150_1_MG_PAK-7 | Sewage | ERR2607520 |
| PER | 2016 | DTU_2016_151_1_MG_PER-35 | Sewage | ERR2607521 |
| POL | 2016 | DTU_2016_152_1_MG_POL-36 | Sewage | ERR2607524 |
| RKS | 2016 | DTU_2016_222_1_MG_XK-60 | Sewage | ERR2607592 |
| SEN | 2016 | DTU_2016_155_1_MG_SEN-8 | Sewage | ERR2607525 |
| SGP | 2016 | DTU_2016_156_1_MG_SGP-52 | Sewage | ERR2607528 |
| SRB | 2016 | DTU_2016_159_1_MG_SRB-37 | Sewage | ERR2607531 |
| SVK | 2016 | DTU_2016_162_1_MG_SVK-9 | Sewage | ERR2607533 |
| SVN | 2016 | DTU_2016_164_1_MG_SVN-38 | Sewage | ERR2607536 |
| SWE | 2016 | DTU_2016_167_1_MG_SWE-41 | Sewage | ERR2607540 |
| SWE | 2016 | DTU_2016_168_1_MG_SWE-41a | Sewage | ERR2607539 |
| TGO | 2016 | DTU_2016_176_1_MG_TGO-44 | Sewage | ERR2607546 |
| TUR | 2016 | DTU_2016_177_1_MG_TUR-46 | Sewage | ERR2607549 |
| TZA | 2016 | DTU_2016_180_1_MG_TZA-15 | Sewage | ERR2607550 |
| USA | 2016 | DTU_2016_181_1_MG_USA-74 | Sewage | ERR2607590 |
| USA | 2016 | DTU_2016_182_1_MG_USA-74a | Sewage | ERR2607554 |
| USA | 2016 | DTU_2016_188_3_MG_USA-74b_2 | Sewage | ERR2607556 |
| USA | 2016 | DTU_2016_193_1_MG_USA-74c_2 | Sewage | ERR2607563 |
| USA | 2016 | DTU_2016_195_1_MG_USA-74d | Sewage | ERR2607567 |
| USA | 2016 | DTU_2016_202_4_MG_USA-74e_3 | Sewage | ERR2607570 |
| USA | 2016 | DTU_2016_209_7_MG_USA-74f_6 | Sewage | ERR2607577 |
| USA | 2016 | DTU_2016_218_9_MG_USA-74g_8 | Sewage | ERR2607586 |
| USA | 2016 | DTU_2016_219_1_MG_USA-74h | Sewage | ERR2607588 |
| USA | 2016 | DTU_2016_220_1_MG_USA-74i | Sewage | ERR2607589 |
| VNM | 2016 | DTU_2016_221_1_MG_VNM-48 | Sewage | ERR2607591 |
| ZAF | 2016 | DTU_2016_223_1_MG_ZAF-39 | Sewage | ERR2607595 |
| ZMB | 2016 | DTU_2016_226_1_MG_ZMB-49 | Sewage | ERR2607605 |
| ZMB | 2016 | DTU_2016_235_7_MG_ZMB-49b_6 | Sewage | ERR2607603 |
| ALB | 2017 | DTU_2017_340_1_MG_AL_TI | Sewage | ERR2683210 |
| AUS | 2017 | DTU_2017_383_1_MG_AU_MA | Sewage | ERR2683257 |
| AUS | 2017 | DTU_2017_384_1_MG_AU_DD | Sewage | ERR2683259 |
| AUS | 2017 | DTU_2017_437_1_MG_AU_ME | Sewage | ERR2683149 |
| AUS | 2017 | DTU_2017_558_1_MG_AU_ME_254 | Sewage | ERR9833719 |
| AUT | 2017 | DTU_2017_354_1_MG_AT_VI | Sewage | ERR2683226 |
| BFA | 2017 | DTU_2017_452_1_MG_BF_OU | Sewage | ERR2683165 |
| BFA | 2017 | DTU_2017_453_1_MG_BF_BO | Sewage | ERR2683166 |
| BGD | 2017 | DTU_2017_373_1_MG_BD_DH | Sewage | ERR2683246 |
| BGR | 2017 | DTU_2017_444_1_MG_BG_SO | Sewage | ERR2683157 |
| BIH | 2017 | DTU_2017_382_1_MG_BA_BL | Sewage | ERR2683256 |
| BRA | 2017 | DTU_2017_320_1_MG_BR_BE | Sewage | ERR2683188 |
| BRB | 2017 | DTU_2017_368_1_MG_BB_BR | Sewage | ERR2683241 |
| BRB | 2017 | DTU_2017_369_1_MG_BB_WO | Sewage | ERR2683242 |
| BWA | 2017 | DTU_2017_396_1_MG_BW_GA | Sewage | ERR2683272 |
| CAN | 2017 | DTU_2017_440_1_MG_CA_RE | Sewage | ERR2683152 |
| CAN | 2017 | DTU_2017_441_1_MG_CA_VA | Sewage | ERR2683153 |
| CAN | 2017 | DTU_2017_442_1_MG_CA_CA | Sewage | ERR2683154 |
| CAN | 2017 | DTU_2017_443_1_MG_CA_OT | Sewage | ERR2683155 |
| CAN | 2017 | DTU_2017_446_1_MG_CA_TO | Sewage | ERR2683159 |
| CHE | 2017 | DTU_2017_347_1_MG_CH_BR | Sewage | ERR2683218 |
| CHN | 2017 | DTU_2017_487_1_MG_CN_GU_183 | Sewage | ERR9834200 |
| CIV | 2017 | DTU_2017_312_1_MG_CI_AB | Sewage | ERR2683269 |
| CIV | 2017 | DTU_2017_313_1_MG_CI_BO_a | Sewage | ERR2683280 |
| CIV | 2017 | DTU_2017_314_1_MG_CI_YA | Sewage | ERR2683123 |
| CIV | 2017 | DTU_2017_395_2_MG_CI_BO_b | Sewage | ERR2683271 |
| CMR | 2017 | DTU_2017_317_1_MG_CM_YA | Sewage | ERR2683156 |
| CZE | 2017 | DTU_2017_362_1_MG_CZ_PR | Sewage | ERR2683234 |
| CZE | 2017 | DTU_2017_363_1_MG_CZ_BR | Sewage | ERR2683235 |
| DEU | 2017 | DTU_2017_339_1_MG_DE_DR | Sewage | ERR2683209 |
| DEU | 2017 | DTU_2017_379_1_MG_DE_BE | Sewage | ERR2683253 |
| DNK | 2017 | DTU_2017_328_1_MG_DK_AV | Sewage | ERR2683197 |
| ECU | 2017 | DTU_2017_400_1_MG_EC_QU | Sewage | ERR2683276 |
| ECU | 2017 | DTU_2017_402_1_MG_EC_GY | Sewage | ERR2683278 |
| ECU | 2017 | DTU_2017_403_1_MG_EC_CU | Sewage | ERR2683279 |
| ECU | 2017 | DTU_2017_404_1_MG_EC_GA | Sewage | ERR2683114 |
| ESP | 2017 | DTU_2017_307_1_MG_ES_BA | Sewage | ERR2683214 |
| EST | 2017 | DTU_2017_425_1_MG_EE_TA | Sewage | ERR2683136 |
| EST | 2017 | DTU_2017_426_1_MG_EE_TL | Sewage | ERR2683137 |
| EST | 2017 | DTU_2017_427_1_MG_EE_PR | Sewage | ERR2683138 |
| EST | 2017 | DTU_2017_428_1_MG_EE_NA | Sewage | ERR2683139 |
| ETH | 2017 | DTU_2017_357_1_MG_ET_AD | Sewage | ERR2683229 |
| FIN | 2017 | DTU_2017_359_1_MG_FI_TU | Sewage | ERR2683231 |
| FIN | 2017 | DTU_2017_360_1_MG_FI_OU | Sewage | ERR2683232 |
| FIN | 2017 | DTU_2017_361_1_MG_FI_HE | Sewage | ERR2683233 |
| FRA | 2017 | DTU_2017_345_1_MG_FR_NA | Sewage | ERR2683216 |
| FRA | 2017 | DTU_2017_346_1_MG_FR_AU | Sewage | ERR2683217 |
| GBR | 2017 | DTU_2017_309_1_MG_GB_FA | Sewage | ERR2683236 |
| GBR | 2017 | DTU_2017_310_1_MG_GB_CA | Sewage | ERR2683247 |
| GBR | 2017 | DTU_2017_439_1_MG_GB_ED | Sewage | ERR2683151 |
| GBR | 2017 | DTU_2017_445_1_MG_GB_NC | Sewage | ERR2683158 |
| GEO | 2017 | DTU_2017_430_1_MG_GE_KU | Sewage | ERR2683141 |
| GHA | 2017 | DTU_2017_341_1_MG_GH_AC | Sewage | ERR2683211 |
| GHA | 2017 | DTU_2017_342_1_MG_GH_KU | Sewage | ERR2683212 |
| GHA | 2017 | DTU_2017_464_1_MG_GH_TA | Sewage | ERR2683179 |
| GRC | 2017 | DTU_2017_308_1_MG_GR_AT | Sewage | ERR2683225 |
| GRC | 2017 | DTU_2017_436_1_MG_GR_TH | Sewage | ERR2683148 |
| DNK | 2017 | DTU_2017_322_1_MG_GL_SI | Sewage | ERR2683190 |
| GTM | 2017 | DTU_2017_458_1_MG_GT_GU | Sewage | ERR2683172 |
| HKG | 2017 | DTU_2017_318_1_MG_HK_HK | Sewage | ERR2683167 |
| HRV | 2017 | DTU_2017_431_1_MG_HR_ZA | Sewage | ERR2683142 |
| HUN | 2017 | DTU_2017_388_1_MG_HU_BU | Sewage | ERR2683263 |
| IND | 2017 | DTU_2017_392_1_MG_IN_CO | Sewage | ERR2683267 |
| IND | 2017 | DTU_2017_448_1_MG_IN_MP | Sewage | ERR2683161 |
| IRL | 2017 | DTU_2017_353_1_MG_IE_GA | Sewage | ERR2683224 |
| IRN | 2017 | DTU_2017_465_1_MG_IR_TH | Sewage | ERR2683180 |
| IRN | 2017 | DTU_2017_472_1_MG_IR_SH | Sewage | ERR2683187 |
| ISL | 2017 | DTU_2017_409_1_MG_IS_RE | Sewage | ERR2683119 |
| ISR | 2017 | DTU_2017_381_1_MG_IL_BE | Sewage | ERR2683255 |
| ITA | 2017 | DTU_2017_350_1_MG_IT_VE | Sewage | ERR2683221 |
| ITA | 2017 | DTU_2017_351_1_MG_IT_CA | Sewage | ERR2683222 |
| ITA | 2017 | DTU_2017_352_1_MG_IT_NO | Sewage | ERR2683223 |
| ITA | 2017 | DTU_2017_434_1_MG_IT_RO | Sewage | ERR2683146 |
| JPN | 2017 | DTU_2017_327_1_MG_JP_JP | Sewage | ERR2683196 |
| KAZ | 2017 | DTU_2017_461_1_MG_KZ_AL | Sewage | ERR2683175 |
| KEN | 2017 | DTU_2017_460_1_MG_KE_NA | Sewage | ERR2683174 |
| KHM | 2017 | DTU_2017_457_1_MG_KH_PP | Sewage | ERR2683171 |
| KOR | 2017 | DTU_2017_435_1_MG_KR_GW | Sewage | ERR2683147 |
| LCA | 2017 | DTU_2017_390_1_MG_LC_CA | Sewage | ERR2683265 |
| LCA | 2017 | DTU_2017_391_1_MG_LC_GR | Sewage | ERR2683266 |
| LKA | 2017 | DTU_2017_467_1_MG_LK_RA | Sewage | ERR2683182 |
| LKA | 2017 | DTU_2017_468_1_MG_LK_MO | Sewage | ERR2683183 |
| LKA | 2017 | DTU_2017_469_1_MG_LK_BO | Sewage | ERR2683184 |
| LKA | 2017 | DTU_2017_470_1_MG_LK_CO | Sewage | ERR2683185 |
| LKA | 2017 | DTU_2017_471_1_MG_LK_MA | Sewage | ERR2683186 |
| LTU | 2017 | DTU_2017_459_1_MG_LT_VI | Sewage | ERR2683173 |
| LUX | 2017 | DTU_2017_433_1_MG_LU_LU | Sewage | ERR2683144 |
| LVA | 2017 | DTU_2017_377_1_MG_LV_RI | Sewage | ERR2683251 |
| LVA | 2017 | DTU_2017_378_1_MG_LV_LI | Sewage | ERR2683252 |
| MAR | 2017 | DTU_2017_466_1_MG_MA_CA | Sewage | ERR2683181 |
| MKD | 2017 | DTU_2017_463_1_MG_MK_SK | Sewage | ERR2683177 |
| MLT | 2017 | DTU_2017_385_1_MG_MT_MS | Sewage | ERR2683260 |
| MLT | 2017 | DTU_2017_386_1_MG_MT_MN | Sewage | ERR2683261 |
| MLT | 2017 | DTU_2017_387_1_MG_MT_GO | Sewage | ERR2683262 |
| MNE | 2017 | DTU_2017_356_1_MG_ME_PO | Sewage | ERR2683228 |
| MUS | 2017 | DTU_2017_380_1_MG_MU_PL | Sewage | ERR2683254 |
| MWI | 2017 | DTU_2017_405_1_MG_MW_KA | Sewage | ERR2683115 |
| MWI | 2017 | DTU_2017_406_1_MG_MW_MZ | Sewage | ERR2683116 |
| MWI | 2017 | DTU_2017_407_1_MG_MW_ZO | Sewage | ERR2683117 |
| MWI | 2017 | DTU_2017_408_1_MG_MW_BL | Sewage | ERR2683118 |
| MYS | 2017 | DTU_2017_315_1_MG_MY_KL | Sewage | ERR2683134 |
| MYS | 2017 | DTU_2017_316_1_MG_MY_KG | Sewage | ERR2683145 |
| MYS | 2017 | DTU_2017_323_1_MG_MY_AS | Sewage | ERR2683191 |
| MYS | 2017 | DTU_2017_324_1_MG_MY_PG | Sewage | ERR2683193 |
| MYS | 2017 | DTU_2017_325_1_MG_MY_IP | Sewage | ERR2683194 |
| MYS | 2017 | DTU_2017_326_1_MG_MY_PB | Sewage | ERR2683195 |
| NGA | 2017 | DTU_2017_394_1_MG_NG_LA | Sewage | ERR2683270 |
| NGA | 2017 | DTU_2017_411_1_MG_NG_IL_a | Sewage | ERR2683121 |
| NGA | 2017 | DTU_2017_412_2_MG_NG_IL_b | Sewage | ERR2683122 |
| NGA | 2017 | DTU_2017_414_4_MG_NG_IL_d | Sewage | ERR2683124 |
| NGA | 2017 | DTU_2017_415_1_MG_NG_ZA_a | Sewage | ERR2683125 |
| NGA | 2017 | DTU_2017_416_2_MG_NG_ZA_b | Sewage | ERR2683126 |
| NGA | 2017 | DTU_2017_417_3_MG_NG_ZA_c | Sewage | ERR2683127 |
| NGA | 2017 | DTU_2017_418_1_MG_NG_IB_a | Sewage | ERR2683128 |
| NGA | 2017 | DTU_2017_419_2_MG_NG_IB_b | Sewage | ERR2683129 |
| NIC | 2017 | DTU_2017_420_1_MG_NI_MN | Sewage | ERR2683130 |
| NIC | 2017 | DTU_2017_421_1_MG_NI_MT | Sewage | ERR2683131 |
| NIC | 2017 | DTU_2017_422_1_MG_NI_GR | Sewage | ERR2683132 |
| NIC | 2017 | DTU_2017_423_1_MG_NI_MS | Sewage | ERR2683133 |
| NLD | 2017 | DTU_2017_329_1_MG_NL_AM | Sewage | ERR2683198 |
| NLD | 2017 | DTU_2017_330_1_MG_NL_UT | Sewage | ERR2683199 |
| NOR | 2017 | DTU_2017_449_1_MG_NO_OS | Sewage | ERR2683162 |
| NPL | 2017 | DTU_2017_456_1_MG_NP_KA | Sewage | ERR2683170 |
| NZL | 2017 | DTU_2017_343_1_MG_NZ_CH | Sewage | ERR2683213 |
| PAK | 2017 | DTU_2017_450_1_MG_PK_KA | Sewage | ERR2683163 |
| PAK | 2017 | DTU_2017_451_1_MG_PK_HY | Sewage | ERR2683164 |
| PER | 2017 | DTU_2017_401_1_MG_PE_PI | Sewage | ERR2683277 |
| PHL | 2017 | DTU_2017_344_1_MG_PH_QU | Sewage | ERR2683215 |
| POL | 2017 | DTU_2017_410_1_MG_PL_PL | Sewage | ERR2683120 |
| PRT | 2017 | DTU_2017_306_1_MG_PT_LI | Sewage | ERR2683203 |
| PRT | 2017 | DTU_2017_438_1_MG_PT_PO | Sewage | ERR2683150 |
| PRY | 2017 | DTU_2017_366_1_MG_PY_SL | Sewage | ERR2683239 |
| PRY | 2017 | DTU_2017_367_1_MG_PY_AS | Sewage | ERR2683240 |
| PRY | 2017 | DTU_2017_455_1_MG_PY_CO | Sewage | ERR2683169 |
| SAU | 2017 | DTU_2017_389_1_MG_SA_KA | Sewage | ERR2683264 |
| SEN | 2017 | DTU_2017_355_1_MG_SN_DA | Sewage | ERR2683227 |
| SGP | 2017 | DTU_2017_454_1_MG_SG_JU | Sewage | ERR2683168 |
| SRB | 2017 | DTU_2017_358_1_MG_RS_BE | Sewage | ERR2683230 |
| SVK | 2017 | DTU_2017_375_1_MG_SK_BR | Sewage | ERR2683249 |
| SVN | 2017 | DTU_2017_348_1_MG_SI_LJ | Sewage | ERR2683219 |
| SWE | 2017 | DTU_2017_349_1_MG_SE_UP | Sewage | ERR2683220 |
| SWE | 2017 | DTU_2017_376_1_MG_SE_GO | Sewage | ERR2683250 |
| TCD | 2017 | DTU_2017_311_1_MG_TD_ND | Sewage | ERR2683258 |
| TGO | 2017 | DTU_2017_393_1_MG_TG_LO | Sewage | ERR2683268 |
| THA | 2017 | DTU_2017_429_1_MG_TH_PA | Sewage | ERR2683140 |
| TWN | 2017 | DTU_2017_397_1_MG_TW_TA | Sewage | ERR2683273 |
| TZA | 2017 | DTU_2017_364_1_MG_TZ_MW | Sewage | ERR2683237 |
| TZA | 2017 | DTU_2017_365_1_MG_TZ_MO | Sewage | ERR2683238 |
| TZA | 2017 | DTU_2017_424_1_MG_TZ_MS | Sewage | ERR2683135 |
| UGA | 2017 | DTU_2017_447_1_MG_UG_KA | Sewage | ERR2683160 |
| URY | 2017 | DTU_2017_331_1_MG_UY_MV | Sewage | ERR2683200 |
| USA | 2017 | DTU_2017_321_1_MG_US_PO | Sewage | ERR2683189 |
| USA | 2017 | DTU_2017_370_1_MG_US_WA | Sewage | ERR2683243 |
| USA | 2017 | DTU_2017_371_1_MG_US_AU | Sewage | ERR2683244 |
| USA | 2017 | DTU_2017_372_1_MG_US_HO_a | Sewage | ERR2683245 |
| USA | 2017 | DTU_2017_374_2_MG_US_HO_b | Sewage | ERR2683248 |
| USA | 2017 | DTU_2017_398_1_MG_US_SE | Sewage | ERR2683274 |
| USA | 2017 | DTU_2017_399_1_MG_US_WO | Sewage | ERR2683275 |
| USA | 2017 | DTU_2017_462_1_MG_US_CH | Sewage | ERR2683176 |
| VNM | 2017 | DTU_2017_319_1_MG_VN_HO | Sewage | ERR2683178 |
| ZAF | 2017 | DTU_2017_432_1_MG_ZA_PR | Sewage | ERR2683143 |
| AUS | 2017 | DTU_2017_959_1_MG_AU1707_L1 | Sewage | ERR8977341 |
| AUS | 2017 | DTU_2017_960_1_MG_AU1708_L2 | Sewage | ERR8977342 |
| AUS | 2017 | DTU_2017_975_1_MG_AU1709_L20 | Sewage | ERR8977357 |
| AUS | 2017 | DTU_2017_976_1_MG_AU1710_L21 | Sewage | ERR8977358 |
| AUS | 2017 | DTU_2017_998_1_MG_AU1712_L43 | Sewage | ERR8977380 |
| CAN | 2017 | DTU_2017_990_1_MG_CA1707_L35 | Sewage | ERR8977372 |
| CAN | 2017 | DTU_2017_991_1_MG_CA1708_L36 | Sewage | ERR8977373 |
| CAN | 2017 | DTU_2017_992_1_MG_CA1709_L37 | Sewage | ERR8977374 |
| CAN | 2017 | DTU_2017_993_1_MG_CA1710_L38 | Sewage | ERR8977375 |
| CAN | 2017 | DTU_2017_997_1_MG_CA1712_L42 | Sewage | ERR8977379 |
| CHN | 2017 | DTU_2017_969_1_MG_CN1707_L12 | Sewage | ERR8977351 |
| CHN | 2017 | DTU_2017_970_1_MG_CN1708_L13 | Sewage | ERR8977352 |
| CHN | 2017 | DTU_2017_971_1_MG_CN1709_L14 | Sewage | ERR8977353 |
| CHN | 2017 | DTU_2017_972_1_MG_CN1710_L15 | Sewage | ERR8977354 |
| CHN | 2017 | DTU_2017_1001_1_MG_CN1712_L46 | Sewage | ERR8977383 |
| CMR | 2017 | DTU_2017_977_1_MG_CM1710_L22 | Sewage | ERR8977359 |
| CMR | 2017 | DTU_2017_978_1_MG_CM1712_L23 | Sewage | ERR8977360 |
| CMR | 2017 | DTU_2017_980_1_MG_CM1707_L25 | Sewage | ERR8977362 |
| CMR | 2017 | DTU_2017_981_1_MG_CM1708_L26 | Sewage | ERR8977363 |
| CMR | 2017 | DTU_2017_982_1_MG_CM1709_L27 | Sewage | ERR8977364 |
| ECU | 2017 | DTU_2017_994_1_MG_EC1712_L39 | Sewage | ERR8977376 |
| GRC | 2017 | DTU_2017_963_1_MG_GR1707_L5 | Sewage | ERR8977345 |
| GRC | 2017 | DTU_2017_964_1_MG_GR1709_L6 | Sewage | ERR8977346 |
| MYS | 2017 | DTU_2017_965_1_MG_MY1707_L8 | Sewage | ERR8977347 |
| MYS | 2017 | DTU_2017_966_1_MG_MY1708_L9 | Sewage | ERR8977348 |
| MYS | 2017 | DTU_2017_967_1_MG_MY1709_L10 | Sewage | ERR8977349 |
| MYS | 2017 | DTU_2017_968_1_MG_MY1710_L11 | Sewage | ERR8977350 |
| MYS | 2017 | DTU_2017_986_1_MG_MY1712_L31 | Sewage | ERR8977368 |
| MYS | 2017 | DTU_2017_989_1_MG_MY1711_L34 | Sewage | ERR8977371 |
| PAK | 2017 | DTU_2017_961_1_MG_PK1708_L3 | Sewage | ERR8977343 |
| PAK | 2017 | DTU_2017_962_1_MG_PK1707_L4 | Sewage | ERR8977344 |
| PAK | 2017 | DTU_2017_973_1_MG_PK1709_L18 | Sewage | ERR8977355 |
| PAK | 2017 | DTU_2017_974_1_MG_PK1710_L19 | Sewage | ERR8977356 |
| USA | 2017 | DTU_2017_983_1_MG_US1709_L28 | Sewage | ERR8977365 |
| USA | 2017 | DTU_2017_984_1_MG_US1710_L29 | Sewage | ERR8977366 |
| USA | 2017 | DTU_2017_985_1_MG_US1712_L30 | Sewage | ERR8977367 |
| USA | 2017 | DTU_2017_1005_1_MG_US1712_L54 | Sewage | ERR8977387 |
| ALB | 2017 | DTU_2017_537_1_MG_AL_TI_233 | Sewage | ERR4682374 |
| ARE | 2017 | DTU_2017_612_1_MG_AE_AD_308 | Sewage | ERR4682443 |
| ARG | 2017 | DTU_2017_622_1_MG_AR_BA_318 | Sewage | ERR4682453 |
| AUS | 2017 | DTU_2017_500_1_MG_AU_DD_196 | Sewage | ERR4682341 |
| AUS | 2017 | DTU_2017_501_1_MG_AU_MA_197 | Sewage | ERR4682342 |
| AUT | 2017 | DTU_2017_493_1_MG_AT_VI_189 | Sewage | ERR4682334 |
| BEL | 2017 | DTU_2017_502_1_MG_BE_GE_198 | Sewage | ERR4682343 |
| BEL | 2017 | DTU_2017_503_1_MG_BE_BR_199 | Sewage | ERR4682344 |
| BEL | 2017 | DTU_2017_504_1_MG_BE_AN_200 | Sewage | ERR4682345 |
| BEL | 2017 | DTU_2017_505_1_MG_BE_DE_201 | Sewage | ERR4682346 |
| BEL | 2017 | DTU_2017_562_1_MG_BE_BR_258 | Sewage | ERR4682398 |
| BEN | 2017 | DTU_2017_519_1_MG_BJ_SB_215 | Sewage | ERR4682357 |
| BFA | 2018 | DTU_2017_577_1_MG_BF_OU_273 | Sewage | ERR4682408 |
| BFA | 2017 | DTU_2017_578_1_MG_BF_BO_274 | Sewage | ERR4682409 |
| BGD | 2017 | DTU_2017_589_1_MG_BD_DH_285 | Sewage | ERR4682420 |
| BIH | 2017 | DTU_2017_513_1_MG_BA_BL_209 | Sewage | ERR4682354 |
| BRB | 2017 | DTU_2017_550_1_MG_BB_BR_246 | Sewage | ERR4682387 |
| BRB | 2017 | DTU_2017_551_1_MG_BB_WO_247 | Sewage | ERR4682388 |
| BWA | 2017 | DTU_2017_580_1_MG_BW_GA_276 | Sewage | ERR4682411 |
| CAN | 2017 | DTU_2017_556_1_MG_CA_QE_252 | Sewage | ERR4682393 |
| CAN | 2017 | DTU_2017_602_1_MG_CA_OT_298 | Sewage | ERR4682433 |
| CAN | 2017 | DTU_2017_603_1_MG_CA_RE_299 | Sewage | ERR4682434 |
| CAN | 2017 | DTU_2017_604_1_MG_CA_CA_300 | Sewage | ERR4682435 |
| CAN | 2017 | DTU_2017_605_1_MG_CA_VA_301 | Sewage | ERR4682436 |
| CHE | 2017 | DTU_2017_499_1_MG_CH_BR_195 | Sewage | ERR4682340 |
| CHL | 2017 | DTU_2017_598_1_MG_CL_SA_294 | Sewage | ERR4682429 |
| CHL | 2017 | DTU_2017_599_1_MG_CL_PA_295 | Sewage | ERR4682430 |
| CHN | 2017 | DTU_2017_634_1_MG_CN_GU_330 | Sewage | ERR4682465 |
| CIV | 2017 | DTU_2017_522_1_MG_CI_AB_218 | Sewage | ERR4682359 |
| CIV | 2017 | DTU_2017_523_1_MG_CI_BO_219 | Sewage | ERR4682360 |
| CIV | 2017 | DTU_2017_524_1_MG_CI_YA_220 | Sewage | ERR4682361 |
| CMR | 2017 | DTU_2017_595_1_MG_CM_YA_291 | Sewage | ERR4682426 |
| COL | 2017 | DTU_2017_579_1_MG_CO_MO_275 | Sewage | ERR4682410 |
| CZE | 2017 | DTU_2017_509_1_MG_CZ_PR_205 | Sewage | ERR4682350 |
| CZE | 2017 | DTU_2017_510_1_MG_CZ_BR_206 | Sewage | ERR4682351 |
| DEU | 2017 | DTU_2017_538_1_MG_DE_BE_234 | Sewage | ERR4682375 |
| DEU | 2017 | DTU_2017_540_1_MG_DE_DR_236 | Sewage | ERR4682377 |
| DNK | 2017 | DTU_2017_631_1_MG_DK_VA_327 | Sewage | ERR4682462 |
| DNK | 2017 | DTU_2017_632_1_MG_DK_MA_328 | Sewage | ERR4682463 |
| DNK | 2017 | DTU_2017_633_1_MG_DK_AV_329 | Sewage | ERR4682464 |
| ECU | 2017 | DTU_2017_627_1_MG_EC_QU_323 | Sewage | ERR4682458 |
| ESP | 2017 | DTU_2017_527_1_MG_ES_BA_223 | Sewage | ERR4682364 |
| EST | 2017 | DTU_2017_568_1_MG_EE_TL_264 | Sewage | ERR4682399 |
| EST | 2017 | DTU_2017_569_1_MG_EE_TA_265 | Sewage | ERR4682400 |
| EST | 2017 | DTU_2017_570_1_MG_EE_PA_266 | Sewage | ERR4682401 |
| EST | 2017 | DTU_2017_571_1_MG_EE_NA_267 | Sewage | ERR4682402 |
| ETH | 2017 | DTU_2017_625_1_MG_ET_AD_321 | Sewage | ERR4682456 |
| FIN | 2017 | DTU_2017_608_1_MG_FI_OU_304 | Sewage | ERR4682439 |
| FIN | 2017 | DTU_2017_609_1_MG_FI_TU_305 | Sewage | ERR4682440 |
| FIN | 2017 | DTU_2017_610_1_MG_FI_HE_306 | Sewage | ERR4682441 |
| FRA | 2017 | DTU_2017_506_1_MG_FR_NA_202 | Sewage | ERR4682347 |
| FRA | 2017 | DTU_2017_507_1_MG_FR_SA_203 | Sewage | ERR4682348 |
| GBR | 2017 | DTU_2017_545_1_MG_GB_CA_241 | Sewage | ERR4682382 |
| GBR | 2017 | DTU_2017_546_1_MG_GB_FA_242 | Sewage | ERR4682383 |
| GBR | 2017 | DTU_2017_596_1_MG_GB_EB_292 | Sewage | ERR4682427 |
| GBR | 2017 | DTU_2017_607_1_MG_GB_NC_303 | Sewage | ERR4682438 |
| GHA | 2017 | DTU_2017_594_1_MG_GH_TA_290 | Sewage | ERR4682425 |
| GHA | 2017 | DTU_2017_606_1_MG_GH_KU_302 | Sewage | ERR4682437 |
| GRC | 2017 | DTU_2017_497_1_MG_GR_AT_193 | Sewage | ERR4682338 |
| GRC | 2017 | DTU_2017_529_1_MG_GR_TH_225 | Sewage | ERR4682366 |
| GRL | 2017 | DTU_2017_542_1_MG_GL_SI_238 | Sewage | ERR4682379 |
| GTM | 2017 | DTU_2017_511_1_MG_GT_GU_207 | Sewage | ERR4682352 |
| HKG | 2017 | DTU_2017_530_1_MG_HK_HK_226 | Sewage | ERR4682367 |
| HRV | 2017 | DTU_2017_496_1_MG_HR_ZA_192 | Sewage | ERR4682337 |
| HUN | 2017 | DTU_2017_539_1_MG_HU_BU_235 | Sewage | ERR4682376 |
| HUN | 2017 | DTU_2017_555_1_MG_HU_SK_251 | Sewage | ERR4682392 |
| IND | 2017 | DTU_2017_544_1_MG_IN_CO_240 | Sewage | ERR4682381 |
| IND | 2017 | DTU_2017_548_1_MG_IN_MP_244 | Sewage | ERR4682385 |
| IRL | 2017 | DTU_2017_554_1_MG_IE_GA_250 | Sewage | ERR4682391 |
| IRN | 2017 | DTU_2017_573_1_MG_IR_SH_269 | Sewage | ERR4682404 |
| IRN | 2018 | DTU_2017_629_1_MG_IR_TH_325 | Sewage | ERR4682460 |
| ISL | 2017 | DTU_2017_532_1_MG_IS_RE_228 | Sewage | ERR4682369 |
| ISR | 2017 | DTU_2017_601_1_MG_IL_BE_297 | Sewage | ERR4682432 |
| ITA | 2017 | DTU_2017_489_1_MG_IT_NO_185 | Sewage | ERR4682330 |
| ITA | 2017 | DTU_2017_490_1_MG_IT_VE_186 | Sewage | ERR4682331 |
| ITA | 2017 | DTU_2017_491_1_MG_IT_CA_187 | Sewage | ERR4682332 |
| ITA | 2017 | DTU_2017_557_1_MG_IT_RO_253 | Sewage | ERR4682394 |
| JPN | 2017 | DTU_2017_547_1_MG_JP_JP_243 | Sewage | ERR4682384 |
| KHM | 2017 | DTU_2017_495_1_MG_KH_PP_191 | Sewage | ERR4682336 |
| KOR | 2017 | DTU_2017_521_1_MG_KR_DA_217 | Sewage | ERR4682358 |
| KOR | 2017 | DTU_2017_576_1_MG_KR_GW_272 | Sewage | ERR4682407 |
| KWT | 2017 | DTU_2017_590_1_MG_KW_KW_286 | Sewage | ERR4682421 |
| LCA | 2017 | DTU_2017_623_1_MG_LC_CA_319 | Sewage | ERR4682454 |
| LCA | 2017 | DTU_2017_624_1_MG_LC_GR_320 | Sewage | ERR4682455 |
| LUX | 2017 | DTU_2017_574_1_MG_LU_LU_270 | Sewage | ERR4682405 |
| MAR | 2017 | DTU_2017_600_1_MG_MA_CA_296 | Sewage | ERR4682431 |
| MNE | 2017 | DTU_2017_508_1_MG_ME_PO_204 | Sewage | ERR4682349 |
| MOZ | 2017 | DTU_2017_628_1_MG_MZ_MA_324 | Sewage | ERR4682459 |
| MUS | 2017 | DTU_2017_561_1_MG_MU_PL_257 | Sewage | ERR4682397 |
| MWI | 2017 | DTU_2017_581_1_MG_MW_ZO_277 | Sewage | ERR4682412 |
| MWI | 2017 | DTU_2017_582_1_MG_MW_CH_278 | Sewage | ERR4682413 |
| MWI | 2017 | DTU_2017_583_1_MG_MW_MA_279 | Sewage | ERR4682414 |
| MWI | 2017 | DTU_2017_584_1_MG_MW_MC_280 | Sewage | ERR4682415 |
| MWI | 2017 | DTU_2017_586_1_MG_MW_LI_282 | Sewage | ERR4682417 |
| MWI | 2017 | DTU_2017_587_1_MG_MW_KA_283 | Sewage | ERR4682418 |
| MWI | 2017 | DTU_2017_588_1_MG_MW_NS_284 | Sewage | ERR4682419 |
| MYS | 2017 | DTU_2017_534_1_MG_MY_KL_230 | Sewage | ERR4682371 |
| MYS | 2017 | DTU_2017_535_1_MG_MY_KG_231 | Sewage | ERR4682372 |
| MYS | 2017 | DTU_2017_536_1_MG_MY_IP_232 | Sewage | ERR4682373 |
| MYS | 2017 | DTU_2017_617_1_MG_MY_AS_313 | Sewage | ERR4682448 |
| MYS | 2017 | DTU_2017_618_1_MG_MY_PG_314 | Sewage | ERR4682449 |
| MYS | 2017 | DTU_2017_619_1_MG_MY_PB_315 | Sewage | ERR4682450 |
| NGA | 2017 | DTU_2017_560_1_MG_NG_LA_256 | Sewage | ERR4682396 |
| NGA | 2018 | DTU_2017_626_1_MG_NG_IL_322 | Sewage | ERR4682457 |
| NIC | 2017 | DTU_2017_498_1_MG_NI_LE_194 | Sewage | ERR4682339 |
| NLD | 2017 | DTU_2017_637_1_MG_NL_UT_333 | Sewage | ERR4682468 |
| NOR | 2017 | DTU_2017_533_1_MG_NO_OS_229 | Sewage | ERR4682370 |
| NPL | 2018 | DTU_2017_591_1_MG_NP_KA_287 | Sewage | ERR4682422 |
| NPL | 2018 | DTU_2017_592_1_MG_NP_BH_288 | Sewage | ERR4682423 |
| NZL | 2017 | DTU_2017_613_1_MG_NZ_CH_309 | Sewage | ERR4682444 |
| PAK | 2017 | DTU_2017_553_1_MG_PK_KA_249 | Sewage | ERR4682390 |
| PHL | 2017 | DTU_2017_621_1_MG_PH_QU_317 | Sewage | ERR4682452 |
| POL | 2017 | DTU_2017_559_1_MG_PL_PL_255 | Sewage | ERR4682395 |
| PRT | 2017 | DTU_2017_494_1_MG_PT_LI_190 | Sewage | ERR4682335 |
| PRT | 2017 | DTU_2017_575_1_MG_PT_PO_271 | Sewage | ERR4682406 |
| PRY | 2017 | DTU_2017_552_1_MG_PY_SL_248 | Sewage | ERR4682389 |
| SAU | 2017 | DTU_2017_525_1_MG_SA_KA_221 | Sewage | ERR4682362 |
| SEN | 2018 | DTU_2017_630_1_MG_SN_DA_326 | Sewage | ERR4682461 |
| SRB | 2017 | DTU_2017_636_1_MG_RS_BE_332 | Sewage | ERR4682467 |
| SVK | 2017 | DTU_2017_528_1_MG_SK_BR_224 | Sewage | ERR4682365 |
| SVN | 2017 | DTU_2017_526_1_MG_SI_LJ_222 | Sewage | ERR4682363 |
| SWE | 2017 | DTU_2017_492_1_MG_SE_GO_188 | Sewage | ERR4682333 |
| SWE | 2017 | DTU_2017_572_1_MG_SE_UP_268 | Sewage | ERR4682403 |
| TCD | 2017 | DTU_2017_541_1_MG_TD_ND_237 | Sewage | ERR4682378 |
| TGO | 2017 | DTU_2017_585_1_MG_TG_LO_281 | Sewage | ERR4682416 |
| THA | 2017 | DTU_2017_488_1_MG_TH_ST_184 | Sewage | ERR4682329 |
| THA | 2017 | DTU_2017_549_1_MG_TH_PA_245 | Sewage | ERR4682386 |
| TWN | 2017 | DTU_2017_543_1_MG_TW_TA_239 | Sewage | ERR4682380 |
| TZA | 2017 | DTU_2017_514_1_MG_TZ_MW_210 | Sewage | ERR4682355 |
| TZA | 2017 | DTU_2017_515_1_MG_TZ_MO_211 | Sewage | ERR4682356 |
| TZA | 2017 | DTU_2017_620_1_MG_TZ_MS_316 | Sewage | ERR4682451 |
| UGA | 2017 | DTU_2017_531_1_MG_UG_KA_227 | Sewage | ERR4682368 |
| URY | 2017 | DTU_2017_597_1_MG_UY_MV_293 | Sewage | ERR4682428 |
| USA | 2017 | DTU_2017_593_1_MG_US_CH_289 | Sewage | ERR4682424 |
| USA | 2017 | DTU_2017_611_1_MG_US_SE_307 | Sewage | ERR4682442 |
| USA | 2018 | DTU_2017_614_1_MG_US_HO_310 | Sewage | ERR4682445 |
| USA | 2018 | DTU_2017_615_1_MG_US_AU_311 | Sewage | ERR4682446 |
| USA | 2018 | DTU_2017_616_1_MG_US_WA_312 | Sewage | ERR4682447 |
| VNM | 2017 | DTU_2017_635_1_MG_VN_HO_331 | Sewage | ERR4682466 |
| ZAF | 2017 | DTU_2017_512_1_MG_ZA_PR_208 | Sewage | ERR4682353 |
| ARE | 2018 | DTU_2018_780_1_MG_AE_DU_477 | Sewage | ERR4682895 |
| ARG | 2018 | DTU_2018_722_1_MG_AR_BA_419 | Sewage | ERR4682844 |
| AUS | 2018 | DTU_2018_757_1_MG_AU_ME_454 | Sewage | ERR4682877 |
| AUS | 2018 | DTU_2018_758_1_MG_AU_DD_455 | Sewage | ERR4682878 |
| AUS | 2018 | DTU_2018_759_1_MG_AU_MA_456 | Sewage | ERR4682879 |
| AUT | 2018 | DTU_2018_666_1_MG_AT_VI_354 | Sewage | ERR4682791 |
| BEL | 2018 | DTU_2018_655_1_MG_BE_OS_343 | Sewage | ERR4682781 |
| BEL | 2018 | DTU_2018_656_1_MG_BE_LE_344 | Sewage | ERR4682782 |
| BEL | 2018 | DTU_2018_657_1_MG_BE_HA_345 | Sewage | ERR4682783 |
| BEN | 2018 | DTU_2018_747_1_MG_BJ_DJ_444 | Sewage | ERR4682867 |
| BFA | 2018 | DTU_2018_668_1_MG_BF_OU_356 | Sewage | ERR4682793 |
| BGD | 2018 | DTU_2018_749_1_MG_BD_DH_446 | Sewage | ERR4682869 |
| BGR | 2018 | DTU_2018_690_1_MG_BG_SO_378 | Sewage | ERR4682813 |
| BIH | 2018 | DTU_2018_667_1_MG_BA_BL_355 | Sewage | ERR4682792 |
| BRA | 2018 | DTU_2018_711_1_MG_BR_BH_408 | Sewage | ERR4682834 |
| BRA | 2018 | DTU_2018_776_1_MG_BR_RJ_473 | Sewage | ERR4682891 |
| BRA | 2018 | DTU_2018_777_1_MG_BR_BE_474 | Sewage | ERR4682892 |
| BRB | 2018 | DTU_2018_712_1_MG_BB_WO_409 | Sewage | ERR4682835 |
| BRB | 2018 | DTU_2018_713_1_MG_BB_BR_410 | Sewage | ERR4682836 |
| BWA | 2018 | DTU_2018_689_1_MG_BW_GA_377 | Sewage | ERR4682812 |
| CAN | 2018 | DTU_2018_686_1_MG_CA_QE_374 | Sewage | ERR4682809 |
| CAN | 2018 | DTU_2018_707_1_MG_CA_CA_395 | Sewage | ERR4682830 |
| CAN | 2018 | DTU_2018_708_1_MG_CA_RE_396 | Sewage | ERR4682831 |
| CAN | 2018 | DTU_2018_720_1_MG_CA_OT_417 | Sewage | ERR4682842 |
| CAN | 2018 | DTU_2018_721_1_MG_CA_VA_418 | Sewage | ERR4682843 |
| CHE | 2018 | DTU_2018_706_1_MG_CH_BR_394 | Sewage | ERR4682829 |
| CHL | 2018 | DTU_2018_737_1_MG_CL_SA_434 | Sewage | ERR4682859 |
| CHL | 2018 | DTU_2018_738_1_MG_CL_SA_2_435 | Sewage | ERR4682860 |
| CHN | 2018 | DTU_2018_704_1_MG_CN_NA_392 | Sewage | ERR4682827 |
| CHN | 2018 | DTU_2018_705_1_MG_CN_YA_393 | Sewage | ERR4682828 |
| CHN | 2018 | DTU_2018_783_1_MG_CN_GU_480 | Sewage | ERR4682898 |
| CIV | 2018 | DTU_2018_717_1_MG_CI_AB_414 | Sewage | ERR4682839 |
| CIV | 2018 | DTU_2018_718_1_MG_CI_YA_415 | Sewage | ERR4682840 |
| CIV | 2018 | DTU_2018_719_1_MG_CI_BO_416 | Sewage | ERR4682841 |
| CIV | 2018 | DTU_2018_735_1_MG_CI_YA_2_432 | Sewage | ERR4682857 |
| CIV | 2018 | DTU_2018_736_1_MG_CI_BO_2_433 | Sewage | ERR4682858 |
| CMR | 2018 | DTU_2018_761_1_MG_CM_YA_458 | Sewage | ERR4682880 |
| COL | 2018 | DTU_2018_772_1_MG_CO_MO_469 | Sewage | ERR4682887 |
| CZE | 2018 | DTU_2018_658_1_MG_CZ_PR_346 | Sewage | ERR4682784 |
| CZE | 2018 | DTU_2018_659_1_MG_CZ_BR_347 | Sewage | ERR4682785 |
| DEU | 2018 | DTU_2018_660_1_MG_DE_DR_348 | Sewage | ERR4682786 |
| DEU | 2018 | DTU_2018_763_1_MG_DE_BE_460 | Sewage | ERR4682882 |
| DNK | 2018 | DTU_2018_647_1_MG_DK_AV_335 | Sewage | ERR4682773 |
| DNK | 2018 | DTU_2018_648_1_MG_DK_VA_336 | Sewage | ERR4682774 |
| DNK | 2018 | DTU_2018_649_1_MG_DK_AM_337 | Sewage | ERR4682775 |
| ECU | 2018 | DTU_2018_716_1_MG_EC_QU_413 | Sewage | ERR4682838 |
| ESP | 2018 | DTU_2018_654_1_MG_ES_BA_342 | Sewage | ERR4682780 |
| FIN | 2018 | DTU_2018_739_1_MG_FI_TU_436 | Sewage | ERR4682861 |
| FIN | 2018 | DTU_2018_740_1_MG_FI_HE_437 | Sewage | ERR4682862 |
| FIN | 2018 | DTU_2018_741_1_MG_FI_OU_438 | Sewage | ERR4682863 |
| FRA | 2018 | DTU_2018_663_1_MG_FR_NA_351 | Sewage | ERR4682788 |
| FRA | 2018 | DTU_2018_664_1_MG_FR_SA_352 | Sewage | ERR4682789 |
| GBR | 2018 | DTU_2018_684_1_MG_GB_FA_372 | Sewage | ERR4682807 |
| GBR | 2018 | DTU_2018_685_1_MG_GB_CA_373 | Sewage | ERR4682808 |
| GBR | 2018 | DTU_2018_734_1_MG_GB_EB_431 | Sewage | ERR4682856 |
| GBR | 2018 | DTU_2018_748_1_MG_GB_NC_445 | Sewage | ERR4682868 |
| GHA | 2018 | DTU_2018_795_1_MG_GH_AC_561 | Sewage | ERR4682910 |
| GHA | 2018 | DTU_2018_796_1_MG_GH_AC_2_566 | Sewage | ERR4682911 |
| GHA | 2018 | DTU_2018_797_1_MG_GH_AC_3_567 | Sewage | ERR4682912 |
| GRC | 2018 | DTU_2018_652_1_MG_GR_AT_340 | Sewage | ERR4682778 |
| GRC | 2018 | DTU_2018_696_1_MG_GR_TH_385 | Sewage | ERR4682819 |
| GRL | 2018 | DTU_2018_743_1_MG_GL_SI_440 | Sewage | ERR4682865 |
| HRV | 2018 | DTU_2018_677_1_MG_HR_ZA_365 | Sewage | ERR4682802 |
| HUN | 2018 | DTU_2018_671_1_MG_HU_BU_359 | Sewage | ERR4682796 |
| IND | 2018 | DTU_2018_698_1_MG_IN_KO_387 | Sewage | ERR4682821 |
| IND | 2018 | DTU_2018_714_1_MG_IN_UD_411 | Sewage | ERR4682837 |
| ISR | 2018 | DTU_2018_744_1_MG_IL_BE_441 | Sewage | ERR4682866 |
| ITA | 2018 | DTU_2018_672_1_MG_IT_NO_360 | Sewage | ERR4682797 |
| ITA | 2018 | DTU_2018_673_1_MG_IT_VE_361 | Sewage | ERR4682798 |
| ITA | 2018 | DTU_2018_674_1_MG_IT_CA_362 | Sewage | ERR4682799 |
| ITA | 2018 | DTU_2018_703_1_MG_IT_RO_391 | Sewage | ERR4682826 |
| JPN | 2018 | DTU_2018_683_1_MG_JP_JP_371 | Sewage | ERR4682806 |
| KEN | 2018 | DTU_2018_750_1_MG_KE_NA_447 | Sewage | ERR4682870 |
| KHM | 2018 | DTU_2018_653_1_MG_KH_PP_341 | Sewage | ERR4682779 |
| KOR | 2018 | DTU_2018_680_1_MG_KR_DA_368 | Sewage | ERR4682803 |
| KOR | 2018 | DTU_2018_693_1_MG_KR_GW_382 | Sewage | ERR4682816 |
| KWT | 2018 | DTU_2018_709_1_MG_KW_KW_397 | Sewage | ERR4682832 |
| LCA | 2018 | DTU_2018_753_1_MG_LC_CA_450 | Sewage | ERR4682873 |
| LCA | 2018 | DTU_2018_754_1_MG_LC_GR_451 | Sewage | ERR4682874 |
| LVA | 2018 | DTU_2018_732_1_MG_LV_RI_429 | Sewage | ERR4682854 |
| LVA | 2018 | DTU_2018_733_1_MG_LV_LI_430 | Sewage | ERR4682855 |
| MAR | 2018 | DTU_2018_710_1_MG_MA_CA_398 | Sewage | ERR4682833 |
| MDG | 2018 | DTU_2018_765_1_MG_MG_AN_462 | Sewage | ERR4682884 |
| MDG | 2018 | DTU_2018_766_1_MG_MG_AN_1_463 | Sewage | ERR4682885 |
| MDG | 2018 | DTU_2018_773_1_MG_MG_AN_2_470 | Sewage | ERR4682888 |
| MKD | 2018 | DTU_2018_755_1_MG_MK_SK_452 | Sewage | ERR4682875 |
| MKD | 2018 | DTU_2018_756_1_MG_MK_SK_453 | Sewage | ERR4682876 |
| MNE | 2018 | DTU_2018_692_1_MG_ME_PO_381 | Sewage | ERR4682815 |
| MUS | 2018 | DTU_2018_694_1_MG_MU_SM_383 | Sewage | ERR4682817 |
| MUS | 2018 | DTU_2018_695_1_MG_MU_GB_384 | Sewage | ERR4682818 |
| MYS | 2018 | DTU_2018_661_1_MG_MY_KL_349 | Sewage | ERR4682787 |
| MYS | 2018 | DTU_2018_725_1_MG_MY_PB_422 | Sewage | ERR4682847 |
| MYS | 2018 | DTU_2018_726_1_MG_MY_PG_423 | Sewage | ERR4682848 |
| MYS | 2018 | DTU_2018_727_1_MG_MY_SU_424 | Sewage | ERR4682849 |
| MYS | 2018 | DTU_2018_728_1_MG_MY_KL_1_425 | Sewage | ERR4682850 |
| MYS | 2018 | DTU_2018_729_1_MG_MY_IH_426 | Sewage | ERR4682851 |
| MYS | 2018 | DTU_2018_730_1_MG_MY_KG_427 | Sewage | ERR4682852 |
| NGA | 2018 | DTU_2018_724_1_MG_NG_LA_421 | Sewage | ERR4682846 |
| NLD | 2018 | DTU_2018_691_1_MG_NL_AM_380 | Sewage | ERR4682814 |
| NLD | 2018 | DTU_2018_701_1_MG_NL_UT_389 | Sewage | ERR4682824 |
| NOR | 2018 | DTU_2018_742_1_MG_NO_OS_439 | Sewage | ERR4682864 |
| NZL | 2018 | DTU_2018_731_1_MG_NZ_CH_428 | Sewage | ERR4682853 |
| NZL | 2018 | DTU_2018_784_1_MG_NZ_DU_481 | Sewage | ERR4682899 |
| PAK | 2018 | DTU_2018_687_1_MG_PK_FA_375 | Sewage | ERR4682810 |
| PAK | 2018 | DTU_2018_775_1_MG_PK_KA_472 | Sewage | ERR4682890 |
| POL | 2018 | DTU_2018_700_1_MG_PL_PL_389 | Sewage | ERR4682823 |
| PRT | 2018 | DTU_2018_669_1_MG_PT_LI_357 | Sewage | ERR4682794 |
| PRT | 2018 | DTU_2018_697_1_MG_PT_PO_386 | Sewage | ERR4682820 |
| PRY | 2018 | DTU_2018_762_1_MG_PY_SL_459 | Sewage | ERR4682881 |
| SAU | 2018 | DTU_2018_665_1_MG_SA_KA_353 | Sewage | ERR4682790 |
| SEN | 2018 | DTU_2018_782_1_MG_SN_DA_479 | Sewage | ERR4682897 |
| SGP | 2017 | DTU_2018_646_1_MG_SG_JU_334 | Sewage | ERR4682772 |
| SGP | 2018 | DTU_2018_767_1_MG_SG_JU_464 | Sewage | ERR4682886 |
| SRB | 2018 | DTU_2018_688_1_MG_RS_BE_376 | Sewage | ERR4682811 |
| SVK | 2018 | DTU_2018_699_1_MG_SK_BR_388 | Sewage | ERR4682822 |
| SVN | 2018 | DTU_2018_675_1_MG_SI_LJ_363 | Sewage | ERR4682800 |
| SWE | 2018 | DTU_2018_650_1_MG_SE_GO_338 | Sewage | ERR4682776 |
| SWE | 2018 | DTU_2018_651_1_MG_SE_UP_339 | Sewage | ERR4682777 |
| TGO | 2018 | DTU_2018_670_1_MG_TG_LO_358 | Sewage | ERR4682795 |
| THA | 2018 | DTU_2018_774_1_MG_TH_PA_471 | Sewage | ERR4682889 |
| TUR | 2018 | DTU_2018_676_1_MG_TR_HA_364 | Sewage | ERR4682801 |
| TWN | 2018 | DTU_2018_702_1_MG_TW_TA_390 | Sewage | ERR4682825 |
| TZA | 2018 | DTU_2018_681_1_MG_TZ_MO_369 | Sewage | ERR4682804 |
| TZA | 2018 | DTU_2018_682_1_MG_TZ_MW_370 | Sewage | ERR4682805 |
| TZA | 2018 | DTU_2018_785_1_MG_TZ_MS_482 | Sewage | ERR4682900 |
| UGA | 2018 | DTU_2018_723_1_MG_UG_KA_420 | Sewage | ERR4682845 |
| USA | 2018 | DTU_2018_751_1_MG_US_SE_448 | Sewage | ERR4682871 |
| USA | 2018 | DTU_2018_752_1_MG_US_SE_2_449 | Sewage | ERR4682872 |
| USA | 2018 | DTU_2018_764_1_MG_US_CH_461 | Sewage | ERR4682883 |
| USA | 2018 | DTU_2018_778_1_MG_US_AT_475 | Sewage | ERR4682893 |
| USA | 2018 | DTU_2018_779_1_MG_US_WA_476 | Sewage | ERR4682894 |
| VNM | 2018 | DTU_2018_781_1_MG_VN_HO_478 | Sewage | ERR4682896 |
| AUS | 2018 | DTU_2018_999_1_MG_AU1801_L44 | Sewage | ERR8977381 |
| AUS | 2018 | DTU_2018_1000_1_MG_AU1802_L45 | Sewage | ERR8977382 |
| AUS | 2018 | DTU_2018_1009_1_MG_AU1803_L58 | Sewage | ERR8977391 |
| AUS | 2018 | DTU_2018_1010_1_MG_AU1804_L59 | Sewage | ERR8977392 |
| AUS | 2018 | DTU_2018_1011_1_MG_AU1805_L60 | Sewage | ERR8977393 |
| AUS | 2018 | DTU_2018_1019_1_MG_AU1807_L70 | Sewage | ERR8977401 |
| AUS | 2018 | DTU_2018_1020_1_MG_AU1808_L71 | Sewage | ERR8977402 |
| CAN | 2018 | DTU_2018_1023_1_MG_CA1803_L74 | Sewage | ERR8977405 |
| CAN | 2018 | DTU_2018_1024_1_MG_CA1804_L75 | Sewage | ERR8977406 |
| CAN | 2018 | DTU_2018_1025_1_MG_CA1805_L76 | Sewage | ERR8977407 |
| CAN | 2018 | DTU_2018_1026_1_MG_CA1807_L77 | Sewage | ERR8977408 |
| CAN | 2018 | DTU_2018_1027_1_MG_CA1808_L78 | Sewage | ERR8977409 |
| CAN | 2018 | DTU_2018_1028_1_MG_CA1809_L79 | Sewage | ERR8977410 |
| CHN | 2018 | DTU_2018_1002_1_MG_CN1801_L47 | Sewage | ERR8977384 |
| CHN | 2018 | DTU_2018_1003_1_MG_CN1802_L48 | Sewage | ERR8977385 |
| CHN | 2018 | DTU_2018_1004_1_MG_CN1803_L49 | Sewage | ERR8977386 |
| CHN | 2018 | DTU_2018_1034_1_MG_CN1804_L85 | Sewage | ERR8977416 |
| CHN | 2018 | DTU_2018_1035_1_MG_CN1805_L86 | Sewage | ERR8977417 |
| CHN | 2018 | DTU_2018_1036_1_MG_CN1808_L87 | Sewage | ERR8977418 |
| CHN | 2018 | DTU_2018_1037_1_MG_CN1809_L88 | Sewage | ERR8977419 |
| CHN | 2018 | DTU_2018_1038_1_MG_CN1810_L89 | Sewage | ERR8977420 |
| CMR | 2018 | DTU_2018_979_1_MG_CM1801_L24 | Sewage | ERR8977361 |
| CMR | 2018 | DTU_2018_1021_1_MG_CM1807_L72 | Sewage | ERR8977403 |
| CMR | 2018 | DTU_2018_1022_1_MG_CM1808_L73 | Sewage | ERR8977404 |
| CMR | 2018 | DTU_2018_1039_1_MG_CM1804_L96 | Sewage | ERR8977421 |
| CMR | 2018 | DTU_2018_1042_1_MG_CM1803_L99 | Sewage | ERR8977424 |
| ECU | 2018 | DTU_2018_995_1_MG_EC1801_L40 | Sewage | ERR8977377 |
| ECU | 2018 | DTU_2018_996_1_MG_EC1802_L41 | Sewage | ERR8977378 |
| ECU | 2018 | DTU_2018_1029_1_MG_EC1803_L80 | Sewage | ERR8977411 |
| ECU | 2018 | DTU_2018_1030_1_MG_EC1804_L81 | Sewage | ERR8977412 |
| ECU | 2018 | DTU_2018_1031_1_MG_EC1805_L82 | Sewage | ERR8977413 |
| ECU | 2018 | DTU_2018_1032_1_MG_EC1807_L83 | Sewage | ERR8977414 |
| ECU | 2018 | DTU_2018_1033_1_MG_EC1808_L84 | Sewage | ERR8977415 |
| MYS | 2018 | DTU_2018_987_1_MG_MY1801_L32 | Sewage | ERR8977369 |
| MYS | 2018 | DTU_2018_988_1_MG_MY1802_L33 | Sewage | ERR8977370 |
| MYS | 2018 | DTU_2018_1012_1_MG_MY1803_L61 | Sewage | ERR8977394 |
| MYS | 2018 | DTU_2018_1013_1_MG_MY1804_L62 | Sewage | ERR8977395 |
| MYS | 2018 | DTU_2018_1014_1_MG_MY1805_L63 | Sewage | ERR8977396 |
| USA | 2018 | DTU_2018_1006_1_MG_US1801_L55 | Sewage | ERR8977388 |
| USA | 2018 | DTU_2018_1007_1_MG_US1802_L56 | Sewage | ERR8977389 |
| USA | 2018 | DTU_2018_1008_1_MG_US1803_L57 | Sewage | ERR8977390 |
| USA | 2018 | DTU_2018_1015_1_MG_US1804_L66 | Sewage | ERR8977397 |
| USA | 2018 | DTU_2018_1016_1_MG_US1805_L67 | Sewage | ERR8977398 |
| USA | 2018 | DTU_2018_1017_1_MG_US1807_L68 | Sewage | ERR8977399 |
| USA | 2018 | DTU_2018_1018_1_MG_US1808_L69 | Sewage | ERR8977400 |
| ARE | 2018 | DTU_2018_930_1_MG_AE_DU_613 | Sewage | ERR4678681 |
| ARG | 2018 | DTU_2018_909_1_MG_AR_BA_594 | Sewage | ERR4678664 |
| AUS | 2018 | DTU_2018_875_1_MG_AU_DD_559 | Sewage | ERR4678630 |
| AUS | 2018 | DTU_2018_876_1_MG_AU_MA_560 | Sewage | ERR4678631 |
| AUT | 2018 | DTU_2018_799_1_MG_AT_VI_483 | Sewage | ERR4678558 |
| BEL | 2018 | DTU_2018_846_1_MG_BE_OS_530 | Sewage | ERR4678601 |
| BEL | 2018 | DTU_2018_847_1_MG_BE_LE_531 | Sewage | ERR4678602 |
| BEL | 2018 | DTU_2018_848_1_MG_BE_BRU_532 | Sewage | ERR4678603 |
| BEL | 2018 | DTU_2018_849_1_MG_BE_DE_533 | Sewage | ERR4678604 |
| BEL | 2018 | DTU_2018_850_1_MG_BE_GH_534 | Sewage | ERR4678605 |
| BGD | 2018 | DTU_2018_874_1_MG_BD_DH_558 | Sewage | ERR4678629 |
| BIH | 2018 | DTU_2018_877_1_MG_BA_BL_562 | Sewage | ERR4678632 |
| BOL | 2019 | DTU_2018_870_1_MG_BO_LP_554 | Sewage | ERR4678625 |
| BRA | 2018 | DTU_2018_830_1_MG_BR_BH_514 | Sewage | ERR4678588 |
| BRA | 2018 | DTU_2018_837_1_MG_BR_RJ_521 | Sewage | ERR4678595 |
| BRB | 2018 | DTU_2018_888_1_MG_BB_WO_575 | Sewage | ERR4678643 |
| BRB | 2018 | DTU_2018_889_1_MG_BB_BR_576 | Sewage | ERR4678644 |
| BWA | 2018 | DTU_2018_866_1_MG_BW_GA_550 | Sewage | ERR4678621 |
| CAN | 2018 | DTU_2018_902_1_MG_CA_CA_587 | Sewage | ERR4678657 |
| CAN | 2018 | DTU_2018_903_1_MG_CA_RE_588 | Sewage | ERR4678658 |
| CAN | 2018 | DTU_2018_904_1_MG_CA_VA_589 | Sewage | ERR4678659 |
| CAN | 2018 | DTU_2018_905_1_MG_CA_OT_590 | Sewage | ERR4678660 |
| CHE | 2018 | DTU_2018_871_1_MG_CH_BR_555 | Sewage | ERR4678626 |
| CHL | 2019 | DTU_2018_884_1_MG_CL_SA_571 | Sewage | ERR4678639 |
| CHL | 2019 | DTU_2018_885_1_MG_CL_SA_2_572 | Sewage | ERR4678640 |
| CHN | 2018 | DTU_2018_864_1_MG_CN_NA_548 | Sewage | ERR4678619 |
| CHN | 2018 | DTU_2018_868_1_MG_CH_SU_552 | Sewage | ERR4678623 |
| CIV | 2018 | DTU_2018_844_1_MG_CI_BO_528 | Sewage | ERR4678599 |
| CIV | 2018 | DTU_2018_949_1_MG_CI_BO_2_630 | Sewage | ERR4678698 |
| CMR | 2018 | DTU_2018_912_1_MG_CM_YA_597 | Sewage | ERR4678667 |
| COD | 2018 | DTU_2018_934_1_MG_CO_BU_616 | Sewage | ERR4678685 |
| COD | 2018 | DTU_2018_936_1_MG_CO_BU_2_618 | Sewage | ERR4678686 |
| COD | 2018 | DTU_2018_937_1_MG_CO_BU_3_619 | Sewage | ERR4678687 |
| COD | 2018 | DTU_2018_938_1_MG_CO_BU_4_620 | Sewage | ERR4678688 |
| COD | 2018 | DTU_2018_939_1_MG_CO_BU_5_621 | Sewage | ERR4678689 |
| COD | 2018 | DTU_2018_941_1_MG_CO_BU_7_623 | Sewage | ERR4678690 |
| COD | 2018 | DTU_2018_942_1_MG_CO_BU_8_624 | Sewage | ERR4678691 |
| COD | 2018 | DTU_2018_943_1_MG_CO_BU_9_625 | Sewage | ERR4678692 |
| COL | 2019 | DTU_2018_933_1_MG_CO_MO_615 | Sewage | ERR4678684 |
| CZE | 2018 | DTU_2018_819_1_MG_CZ_PR_503 | Sewage | ERR4678577 |
| CZE | 2018 | DTU_2018_820_1_MG_CZ_BR_504 | Sewage | ERR4678578 |
| DEU | 2018 | DTU_2018_818_1_MG_DE_DR_502 | Sewage | ERR4678576 |
| DEU | 2018 | DTU_2018_880_1_MG_DE_BE_565 | Sewage | ERR4678635 |
| DNK | 2018 | DTU_2018_916_1_MG_DK_AV_601 | Sewage | ERR4678671 |
| DNK | 2018 | DTU_2018_917_1_MG_DK_VA_602 | Sewage | ERR4678672 |
| DNK | 2018 | DTU_2018_919_2_MG_DK_AM_603_re_1 | Sewage | ERR4678674 |
| ESP | 2018 | DTU_2018_815_1_MG_ES_BA_499 | Sewage | ERR4678573 |
| ESP | 2018 | DTU_2018_816_1_MG_ES_IL_500 | Sewage | ERR4678574 |
| ESP | 2019 | DTU_2018_894_1_MG_ES_SA_581 | Sewage | ERR4678649 |
| FIN | 2018 | DTU_2018_898_2_MG_FI_OU_584_re_1 | Sewage | ERR4678653 |
| FIN | 2018 | DTU_2018_899_1_MG_FI_TU_585 | Sewage | ERR4678654 |
| FIN | 2018 | DTU_2018_901_2_MG_FI_HE_586_re_1 | Sewage | ERR4678656 |
| FRA | 2018 | DTU_2018_806_1_MG_FR_NA_490 | Sewage | ERR4678565 |
| FRA | 2018 | DTU_2018_807_1_MG_FR_SA_491 | Sewage | ERR4678566 |
| GBR | 2018 | DTU_2018_808_1_MG_GB_FA_492 | Sewage | ERR4678567 |
| GBR | 2018 | DTU_2018_809_1_MG_GB_CA_493 | Sewage | ERR4678568 |
| GBR | 2018 | DTU_2018_872_1_MG_GB_EB_556 | Sewage | ERR4678627 |
| GHA | 2019 | DTU_2018_878_1_MG_GH_TA_563 | Sewage | ERR4678633 |
| GHA | 2018 | DTU_2018_881_1_MG_GH_AC_568 | Sewage | ERR4678636 |
| GHA | 2018 | DTU_2018_882_1_MG_GH_AC_2_569 | Sewage | ERR4678637 |
| GHA | 2018 | DTU_2018_883_1_MG_GH_KA_570 | Sewage | ERR4678638 |
| GRC | 2018 | DTU_2018_825_1_MG_GR_AT_509 | Sewage | ERR4678583 |
| GRC | 2019 | DTU_2018_887_1_MG_GR_TH_574 | Sewage | ERR4678642 |
| GRL | 2018 | DTU_2018_920_1_MG_GL_SI_604 | Sewage | ERR4678675 |
| GTM | 2018 | DTU_2018_810_1_MG_GT_GU_494 | Sewage | ERR4678569 |
| HRV | 2018 | DTU_2018_814_1_MG_HR_ZA_498 | Sewage | ERR4678572 |
| HUN | 2018 | DTU_2018_811_1_MG_HU_BU_495 | Sewage | ERR4678570 |
| HUN | 2018 | DTU_2018_817_1_MG_HU_SK_501 | Sewage | ERR4678575 |
| IND | 2018 | DTU_2018_838_1_MG_IN_UD_522 | Sewage | ERR4678596 |
| IND | 2018 | DTU_2018_851_1_MG_IN_KO_535 | Sewage | ERR4678606 |
| ISL | 2018 | DTU_2018_821_1_MG_IS_RE_505 | Sewage | ERR4678579 |
| ISR | 2018 | DTU_2018_805_1_MG_IL_BE_489 | Sewage | ERR4678564 |
| ITA | 2018 | DTU_2018_860_1_MG_IT_NO_544 | Sewage | ERR4678615 |
| ITA | 2018 | DTU_2018_862_1_MG_IT_VE_546 | Sewage | ERR4678617 |
| ITA | 2018 | DTU_2018_863_1_MG_IT_CA_547 | Sewage | ERR4678618 |
| ITA | 2018 | DTU_2018_896_1_MG_IT_RO_583 | Sewage | ERR4678651 |
| JPN | 2018 | DTU_2018_831_1_MG_JP_HI_AM_515 | Sewage | ERR4678589 |
| JPN | 2018 | DTU_2018_832_1_MG_JP_HI_SB_516 | Sewage | ERR4678590 |
| JPN | 2018 | DTU_2018_833_1_MG_JP_HI_EB_517 | Sewage | ERR4678591 |
| JPN | 2018 | DTU_2018_834_1_MG_JP_HI_SD_518 | Sewage | ERR4678592 |
| JPN | 2018 | DTU_2018_879_1_MG_JP_JP_564 | Sewage | ERR4678634 |
| KHM | 2019 | DTU_2018_854_1_MG_KH_PP_538 | Sewage | ERR4678609 |
| KOR | 2018 | DTU_2018_845_1_MG_KR_DA_529 | Sewage | ERR4678600 |
| KOR | 2018 | DTU_2018_865_1_MG_KR_GW_549 | Sewage | ERR4678620 |
| LVA | 2018 | DTU_2018_803_1_MG_LV_RI_487 | Sewage | ERR4678562 |
| LVA | 2018 | DTU_2018_804_1_MG_LV_LI_488 | Sewage | ERR4678563 |
| MAR | 2018 | DTU_2018_892_1_MG_MA_CA_579 | Sewage | ERR4678647 |
| MDG | 2018 | DTU_2018_813_1_MG_MG_AN_497 | Sewage | ERR4678571 |
| MDG | 2018 | DTU_2018_855_1_MG_MG_AN_3_539 | Sewage | ERR4678610 |
| MDG | 2018 | DTU_2018_857_1_MG_MG_AN_4_541 | Sewage | ERR4678612 |
| MLT | 2018 | DTU_2018_835_1_MG_MT_MT_519 | Sewage | ERR4678593 |
| MNE | 2019 | DTU_2018_910_1_MG_ME_PO_595 | Sewage | ERR4678665 |
| MOZ | 2018 | DTU_2018_906_1_MG_MZ_MA_591 | Sewage | ERR4678661 |
| MUS | 2018 | DTU_2018_858_1_MG_MU_SM_542 | Sewage | ERR4678613 |
| MUS | 2018 | DTU_2018_859_1_MG_MU_GB_543 | Sewage | ERR4678614 |
| MYS | 2018 | DTU_2018_823_1_MG_MY_KG_507 | Sewage | ERR4678581 |
| MYS | 2018 | DTU_2018_824_1_MG_MY_KL_508 | Sewage | ERR4678582 |
| MYS | 2018 | DTU_2018_926_1_MG_MY_PB_609 | Sewage | ERR4678677 |
| MYS | 2018 | DTU_2018_927_1_MG_MY_PG_610 | Sewage | ERR4678678 |
| MYS | 2018 | DTU_2018_928_1_MG_MY_SU_611 | Sewage | ERR4678679 |
| NGA | 2018 | DTU_2018_886_1_MG_NG_IL_573 | Sewage | ERR4678641 |
| NGA | 2018 | DTU_2018_890_1_MG_NG_IL_2_577 | Sewage | ERR4678645 |
| NGA | 2018 | DTU_2018_908_1_MG_NG_LA_593 | Sewage | ERR4678663 |
| NGA | 2017 | DTU_2018_948_2_MG_NG_IB_629_re_1 | Sewage | ERR4678697 |
| NLD | 2018 | DTU_2018_925_1_MG_NL_UT_608 | Sewage | ERR4678676 |
| NPL | 2019 | DTU_2018_907_1_MG_NP_KA_592 | Sewage | ERR4678662 |
| NZL | 2018 | DTU_2018_915_1_MG_NZ_CH_600 | Sewage | ERR4678670 |
| PAK | 2018 | DTU_2018_869_1_MG_PK_FA_553 | Sewage | ERR4678624 |
| PAK | 2017 | DTU_2018_944_1_MG_PK_HY_a_626 | Sewage | ERR4678693 |
| POL | 2018 | DTU_2018_932_2_MG_PL_PL_614_re_1 | Sewage | ERR4678683 |
| PRT | 2018 | DTU_2018_826_1_MG_PT_LI_510 | Sewage | ERR4678584 |
| PRT | 2019 | DTU_2018_895_1_MG_PT_PO_582 | Sewage | ERR4678650 |
| SAU | 2018 | DTU_2018_827_1_MG_SA_KA_511 | Sewage | ERR4678585 |
| SEN | 2019 | DTU_2018_891_1_MG_SN_DA_578 | Sewage | ERR4678646 |
| SGP | 2019 | DTU_2018_911_1_MG_SG_JU_596 | Sewage | ERR4678666 |
| SRB | 2019 | DTU_2018_913_1_MG_RS_BE_598 | Sewage | ERR4678668 |
| SRB | 2019 | DTU_2018_914_1_MG_RS_BE_2_599 | Sewage | ERR4678669 |
| SVK | 2018 | DTU_2018_893_1_MG_SK_BR_580 | Sewage | ERR4678648 |
| SVN | 2018 | DTU_2018_828_1_MG_SI_LJ_512 | Sewage | ERR4678586 |
| SWE | 2018 | DTU_2018_801_1_MG_SE_GO_485 | Sewage | ERR4678560 |
| TCD | 2018 | DTU_2018_802_1_MG_TD_ND_486 | Sewage | ERR4678561 |
| TGO | 2018 | DTU_2018_929_1_MG_TG_LO_612 | Sewage | ERR4678680 |
| THA | 2018 | DTU_2018_829_1_MG_TH_PA_513 | Sewage | ERR4678587 |
| TUR | 2018 | DTU_2018_836_1_MG_TR_HA_520 | Sewage | ERR4678594 |
| TWN | 2018 | DTU_2018_861_1_MG_TW_TA_545 | Sewage | ERR4678616 |
| TZA | 2018 | DTU_2018_839_1_MG_TZ_MO_523 | Sewage | ERR4678597 |
| TZA | 2018 | DTU_2018_840_1_MG_TZ_MW_524 | Sewage | ERR4678598 |
| UGA | 2019 | DTU_2018_856_1_MG_UG_KA_540 | Sewage | ERR4678611 |
| URY | 2018 | DTU_2018_853_1_MG_UY_MO_537 | Sewage | ERR4678608 |
| USA | 2018 | DTU_2018_822_1_MG_US_CO_506 | Sewage | ERR4678580 |
| USA | 2018 | DTU_2018_852_1_MG_US_AT_536 | Sewage | ERR4678607 |
| USA | 2019 | DTU_2018_873_1_MG_US_CH_557 | Sewage | ERR4678628 |
| USA | 2018 | DTU_2018_945_1_MG_US_CH_2_627 | Sewage | ERR4678694 |
| USA | 2018 | DTU_2018_946_1_MG_US_HO_2_628 | Sewage | ERR4678695 |
| ZAF | 2018 | DTU_2018_800_1_MG_ZA_PR_484 | Sewage | ERR4678559 |
| ZAF | 2018 | DTU_2018_867_1_MG_ZA_CT_551 | Sewage | ERR4678622 |
| TZA | 2018 | DTU_2018_1064_1_MG_TZ_MO_2 | Sewage | ERR8977425 |
| TZA | 2018 | DTU_2018_1065_1_MG_TZ_MO_3 | Sewage | ERR8977426 |
| TZA | 2018 | DTU_2018_1066_1_MG_TZ_MO_4 | Sewage | ERR8977427 |
| TZA | 2018 | DTU_2018_1067_1_MG_TZ_MO_5 | Sewage | ERR8977428 |
| TZA | 2018 | DTU_2018_1068_1_MG_TZ_MO_6 | Sewage | ERR8977429 |
| TZA | 2018 | DTU_2018_1069_1_MG_TZ_MO_7 | Sewage | ERR8977430 |
| TZA | 2018 | DTU_2018_1070_1_MG_TZ_MO_8 | Sewage | ERR8977431 |
| TZA | 2018 | DTU_2018_1071_1_MG_TZ_MO_9 | Sewage | ERR8977432 |
| TZA | 2018 | DTU_2018_1072_1_MG_TZ_MO_10 | Sewage | ERR8977433 |
| CMR | 2019 | DTU_2019_1040_1_MG_CM1805_L97 | Sewage | ERR8977422 |
| CMR | 2019 | DTU_2019_1041_1_MG_CM1802_L98 | Sewage | ERR8977423 |
| USA | 2018 | DTU_2021_1011236_1_MG_US_SE_2_631_S0_L001 | Sewage | ERR14127555 |
| USA | 2018 | DTU_2021_1011237_1_MG_US_SE_632_S0_L001 | Sewage | ERR14127749 |
| SWE | 2019 | DTU_2021_1011238_1_MG_SE_GO_633_S0_L001 | Sewage | ERR14128251 |
| MEX | 2019 | DTU_2021_1011239_1_MG_MX_SP_634_S0_L001 | Sewage | ERR14128259 |
| MEX | 2019 | DTU_2021_1011240_1_MG_MX_QU_635_S4_L002 | Sewage | ERR14128267 |
| ESP | 2019 | DTU_2021_1011241_1_MG_ES_BA_636_S5_L002 | Sewage | ERR14128659 |
| AUT | 2019 | DTU_2021_1011242_1_MG_AT_VI_637_S0_L001 | Sewage | ERR14129299 |
| CZE | 2019 | DTU_2021_1011244_1_MG_CZ_PR_639_S0_L001 | Sewage | ERR14129308 |
| CZE | 2019 | DTU_2021_1011245_1_MG_CZ_BR_640_S0_L001 | Sewage | ERR14129309 |
| SAU | 2019 | DTU_2021_1011246_1_MG_SA_KA_641_S1_L002 | Sewage | ERR14129321 |
| SVN | 2019 | DTU_2021_1011247_1_MG_SI_LJ_642_S6_L002 | Sewage | ERR14129343 |
| KOR | 2020 | DTU_2021_1011248_1_MG_KR_DA_643_S27_L002 | Sewage | ERR14129401 |
| NOR | 2020 | DTU_2021_1011249_1_MG_NO_OS_644_S28_L002 | Sewage | ERR14129411 |
| NGA | 2019 | DTU_2021_1011250_1_MG_NG_MA_646_S29_L002 | Sewage | ERR14129420 |
| FRA | 2019 | DTU_2021_1011251_1_MG_FR_NA_647_S7_L002 | Sewage | ERR14129438 |
| FRA | 2019 | DTU_2021_1011252_1_MG_FR_AU_648_S30_L002 | Sewage | ERR14129535 |
| CAN | 2019 | DTU_2021_1011253_1_MG_CA_WA_649_S8_L002 | Sewage | ERR14129572 |
| NLD | 2019 | DTU_2021_1011254_1_MG_NL_RO_650_S9_L002 | Sewage | ERR14129706 |
| ESP | 2019 | DTU_2021_1011255_1_MG_ES_MA_651_S31_L002 | Sewage | ERR14134533 |
| CHE | 2019 | DTU_2021_1011256_1_MG_CH_BR_652_S32_L002 | Sewage | ERR14134559 |
| NGA | 2020 | DTU_2021_1011257_1_MG_NG_NS_654_S10_L002 | Sewage | ERR14137403 |
| TWN | 2020 | DTU_2021_1011258_1_MG_TW_TA_655_S33_L002 | Sewage | ERR14137417 |
| KOR | 2019 | DTU_2021_1011259_1_MG_KR_GW_658_S11_L002 | Sewage | ERR14141069 |
| TUR | 2020 | DTU_2021_1011260_1_MG_TR_HA_659_S12_L002 | Sewage | ERR14141077 |
| FIN | 2020 | DTU_2021_1011261_1_MG_FI_KU_660_S0_L001 | Sewage | ERR14141090 |
| POL | 2020 | DTU_2021_1011262_1_MG_PL_PL_661_S34_L002 | Sewage | ERR14141097 |
| MDA | 2019 | DTU_2021_1011263_1_MG_MD_OR_662_S35_L002 | Sewage | ERR14141105 |
| MDA | 2019 | DTU_2021_1011264_1_MG_MD_CA_663_S13_L002 | Sewage | ERR14141109 |
| MDA | 2019 | DTU_2021_1011265_1_MG_MD_BA_664_S0_L001 | Sewage | ERR14141113 |
| MDA | 2019 | DTU_2021_1011266_1_MG_MD_ED_665_S118_L003 | Sewage | ERR14141118 |
| MDA | 2019 | DTU_2021_1011267_1_MG_MD_CO_666_S14_L002 | Sewage | ERR14141121 |
| MDA | 2019 | DTU_2021_1011268_1_MG_MD_UN_667_S0_L001 | Sewage | ERR14141124 |
| MDA | 2019 | DTU_2021_1011269_1_MG_MD_CI_668_S0_L001 | Sewage | ERR14141125 |
| MDA | 2019 | DTU_2021_1011270_1_MG_MD_HI_669_S2_L002 | Sewage | ERR14141127 |
| MDA | 2020 | DTU_2021_1011271_1_MG_MD_SO_670_S0_L001 | Sewage | ERR14141128 |
| MDA | 2019 | DTU_2021_1011272_1_MG_MD_CH_671_S0_L001 | Sewage | ERR14141129 |
| GBR | 2019 | DTU_2021_1011273_1_MG_GB_FA_672_S38_L002 | Sewage | ERR14141130 |
| GBR | 2019 | DTU_2021_1011274_1_MG_GB_CA_673_S15_L002 | Sewage | ERR14141131 |
| TTO | 2020 | DTU_2021_1011275_1_MG_TT_AR_674_S0_L001 | Sewage | ERR14141134 |
| ISL | 2020 | DTU_2021_1011276_1_MG_IS_RE_675_S39_L002 | Sewage | ERR14141135 |
| TZA | 2020 | DTU_2021_1011277_1_MG_TZ_DS_676_S0_L001 | Sewage | ERR14141136 |
| LVA | 2019 | DTU_2021_1011278_1_MG_LV_LI_677_S0_L001 | Sewage | ERR14141137 |
| LVA | 2019 | DTU_2021_1011279_1_MG_LV_RI_678_S0_L001 | Sewage | ERR14141138 |
| PRT | 2019 | DTU_2021_1011280_1_MG_PT_PO_679_S0_L001 | Sewage | ERR14141139 |
| FIN | 2020 | DTU_2021_1011281_1_MG_FI_ES_680_S0_L001 | Sewage | ERR14141140 |
| FIN | 2020 | DTU_2021_1011282_1_MG_FI_RO_681_S0_L001 | Sewage | ERR14141141 |
| GRC | 2020 | DTU_2021_1011283_1_MG_GR_AT_682_S0_L001 | Sewage | ERR14141143 |
| ITA | 2020 | DTU_2021_1011284_1_MG_IT_RO_683_S0_L001 | Sewage | ERR14141144 |
| BOL | 2020 | DTU_2021_1011285_1_MG_BZ_LP_684_S0_L001 | Sewage | ERR14141145 |
| GHA | 2020 | DTU_2021_1011286_1_MG_GH_TA_685_S0_L001 | Sewage | ERR14141146 |
| GHA | 2020 | DTU_2021_1011287_1_MG_GH_TA_2_686_S0_L001 | Sewage | ERR14141147 |
| ITA | 2020 | DTU_2021_1011288_1_MG_IT_AN_687_S19_L002 | Sewage | ERR14141148 |
| ITA | 2020 | DTU_2021_1011289_1_MG_IT_PE_688_S0_L001 | Sewage | ERR14141149 |
| ITA | 2020 | DTU_2021_1011290_1_MG_IT_MA_689_S0_L001 | Sewage | ERR14141150 |
| ITA | 2020 | DTU_2021_1011291_1_MG_IT_FE_690_S0_L001 | Sewage | ERR14142349 |
| ITA | 2020 | DTU_2021_1011292_1_MG_IT_AP_691_S0_L001 | Sewage | ERR14144744 |
| DNK | 2019 | DTU_2021_1011293_1_MG_DK_AM_692_S0_L001 | Sewage | ERR14144745 |
| DNK | 2019 | DTU_2021_1011294_1_MG_DK_AV_693_S0_L001 | Sewage | ERR14144746 |
| DNK | 2019 | DTU_2021_1011295_1_MG_DK_VA_694_S0_L001 | Sewage | ERR14145978 |
| ESP | 2020 | DTU_2021_1011296_1_MG_ES_VA_695_S0_L001 | Sewage | ERR14145980 |
| AUT | 2020 | DTU_2021_1011297_1_MG_AT_GR_698_S0_L001 | Sewage | ERR14145981 |
| FIN | 2019 | DTU_2021_1011298_1_MG_FI_OU_699_S0_L001 | Sewage | ERR14145982 |
| FIN | 2020 | DTU_2021_1011299_1_MG_FI_TU_700_S0_L001 | Sewage | ERR14145983 |
| FIN | 2019 | DTU_2021_1011300_1_MG_FI_HE_701_S0_L001 | Sewage | ERR14146272 |
| GRL | 2020 | DTU_2021_1011301_1_MG_GL_NU_702_S0_L001 | Sewage | ERR14146274 |
| MLT | 2020 | DTU_2021_1011302_1_MG_MT_GO_703_S0_L001 | Sewage | ERR14146275 |
| MLT | 2020 | DTU_2021_1011303_1_MG_MT_XG_704_S0_L001 | Sewage | ERR14146276 |
| ECU | 2019 | DTU_2021_1011304_1_MG_EC_QU_705_S0_L001 | Sewage | ERR14146277 |
| ECU | 2019 | DTU_2021_1011305_1_MG_EC_QU_2_706_S0_L001 | Sewage | ERR14146279 |
| ECU | 2020 | DTU_2021_1011306_1_MG_EC_AM_707_S0_L001 | Sewage | ERR14146281 |
| ECU | 2020 | DTU_2021_1011307_1_MG_EC_LA_708_S0_L001 | Sewage | ERR14146282 |
| ECU | 2020 | DTU_2021_1011308_1_MG_EC_GY_709_S0_L001 | Sewage | ERR14146284 |
| ECU | 2020 | DTU_2021_1011309_1_MG_EC_GU_710_S0_L001 | Sewage | ERR14146288 |
| ECU | 2019 | DTU_2021_1011310_1_MG_EC_EC_711_S0_L001 | Sewage | ERR14146289 |
| USA | 2020 | DTU_2021_1011311_1_MG_US_SE_712_S0_L001 | Sewage | ERR14146300 |
| NGA | 2020 | DTU_2021_1011312_1_MG_NG_IH_713_S0_L001 | Sewage | ERR14146307 |
| NGA | 2020 | DTU_2021_1011313_1_MG_NG_SA_714_S0_L001 | Sewage | ERR14146311 |
| GHA | 2020 | DTU_2021_1011314_1_MG_GH_TI_715_S0_L001 | Sewage | ERR14146316 |
| NGA | 2020 | DTU_2021_1011315_1_MG_NG_OS_717_S0_L001 | Sewage | ERR14146321 |
| NZL | 2020 | DTU_2021_1011316_1_MG_NZ_CH_718_S0_L001 | Sewage | ERR14146325 |
| NGA | 2020 | DTU_2021_1011317_1_MG_NG_KI_719_S0_L001 | Sewage | ERR14146330 |
| NGA | 2020 | DTU_2021_1011318_1_MG_NG_MI_720_S0_L001 | Sewage | ERR14146331 |
| ITA | 2019 | DTU_2021_1011319_1_MG_IT_TO_721_S0_L001 | Sewage | ERR14146333 |
| ITA | 2020 | DTU_2021_1011320_1_MG_IT_NA_722_S0_L001 | Sewage | ERR14146334 |
| ITA | 2019 | DTU_2021_1011321_1_MG_IT_MI_723_S0_L001 | Sewage | ERR14146335 |
| ITA | 2019 | DTU_2021_1011322_1_MG_IT_MI_2_724_S0_L001 | Sewage | ERR14146337 |
| NPL | 2019 | DTU_2021_1011323_1_MG_NP_KA_725_S0_L001 | Sewage | ERR14146339 |
| BHR | 2019 | DTU_2021_1011324_1_MG_BH_MA_726_S0_L001 | Sewage | ERR14146340 |
| ZAF | 2020 | DTU_2021_1011325_1_MG_ZA_PR_727_S0_L001 | Sewage | ERR14146344 |
| COM | 2020 | DTU_2021_1011326_1_MG_KM_MO_728_S0_L001 | Sewage | ERR14146346 |
| PRY | 2020 | DTU_2021_1011327_1_MG_PY_AS_729_S0_L001 | Sewage | ERR14146348 |
| MDG | 2019 | DTU_2021_1011328_1_MG_MG_AN_2_730_S0_L001 | Sewage | ERR14146351 |
| LUX | 2019 | DTU_2021_1011329_1_MG_LU_LU_731_S0_L001 | Sewage | ERR14146390 |
| GBR | 2019 | DTU_2021_1011330_1_MG_GB_OX_732_S0_L001 | Sewage | ERR14146393 |
| CHL | 2020 | DTU_2021_1011331_1_MG_CL_CO_733_S0_L001 | Sewage | ERR14146397 |
| BRA | 2020 | DTU_2021_1011332_1_MG_BR_PA_734_S0_L001 | Sewage | ERR14146401 |
| NGA | 2020 | DTU_2021_1011333_1_MG_NG_LA_735_S0_L001 | Sewage | ERR14146409 |
| CHL | 2020 | DTU_2021_1011334_1_MG_CL_MO_736_S117_L001 | Sewage | ERR14146414 |
| CHL | 2020 | DTU_2021_1011335_1_MG_CL_BU_737_S0_L001 | Sewage | ERR14146425 |
| CHL | 2020 | DTU_2021_1011336_1_MG_CL_PE_738_S0_L001 | Sewage | ERR14146426 |
| GHA | 2020 | DTU_2021_1011337_1_MG_GH_KU_739_S0_L001 | Sewage | ERR14146428 |
| MEX | 2019 | DTU_2021_1011339_1_MG_MX_MC_741_S0_L001 | Sewage | ERR14146446 |
| NGA | 2020 | DTU_2021_1011340_1_MG_NG_IB_742_S0_L001 | Sewage | ERR14146531 |
| NGA | 2020 | DTU_2021_1011341_1_MG_NG_IL_743_S0_L001 | Sewage | ERR14146536 |
| GBR | 2020 | DTU_2021_1011342_1_MG_GB_BL_744_S0_L001 | Sewage | ERR14146538 |
| PRT | 2020 | DTU_2021_1011343_1_MG_PT_PE_745_S0_L001 | Sewage | ERR14146627 |
| PRT | 2019 | DTU_2021_1011344_1_MG_PT_LO_746_S0_L001 | Sewage | ERR14146630 |
| SRB | 2019 | DTU_2021_1011347_1_MG_RS_BE_2_749_S0_L001 | Sewage | ERR14146667 |
| SRB | 2019 | DTU_2021_1011348_1_MG_RS_BE_3_750_S0_L001 | Sewage | ERR14146670 |
| SRB | 2019 | DTU_2021_1011349_1_MG_RS_BE_4_751_S0_L001 | Sewage | ERR14146673 |
| SRB | 2019 | DTU_2021_1011350_1_MG_RS_NS_752_S0_L001 | Sewage | ERR14146689 |
| SRB | 2019 | DTU_2021_1011351_1_MG_RS_NS_4_755_S0_L001 | Sewage | ERR14146694 |
| NZL | 2020 | DTU_2021_1011352_1_MG_NZ_NE_756_S211_L002 | Sewage | ERR14146698 |
| NZL | 2020 | DTU_2021_1011353_1_MG_NZ_NE_2_757_S0_L001 | Sewage | ERR14146702 |
| MNE | 2019 | DTU_2021_1011354_1_MG_ME_PO_758_S0_L001 | Sewage | ERR14146710 |
| DEU | 2020 | DTU_2021_1011355_1_MG_DE_BE_759_S0_L001 | Sewage | ERR14146715 |
| ISR | 2020 | DTU_2021_1011356_1_MG_IL_SO_760_S0_L001 | Sewage | ERR14146722 |
| NZL | 2020 | DTU_2021_1011357_1_MG_NZ_CH_2_761_S0_L001 | Sewage | ERR14146728 |
| MWI | 2020 | DTU_2021_1011358_1_MG_MW_MU_762_S0_L001 | Sewage | ERR14146731 |
| MWI | 2020 | DTU_2021_1011359_1_MG_MW_MM_764_S70_L001 | Sewage | ERR14146745 |
| MWI | 2020 | DTU_2021_1011360_1_MG_MW_CA_765_S0_L001 | Sewage | ERR14146748 |
| MWI | 2020 | DTU_2021_1011361_1_MG_MW_KH_766_S0_L001 | Sewage | ERR14146753 |
| SVK | 2020 | DTU_2021_1011362_1_MG_SK_BR_767_S212_L002 | Sewage | ERR14146757 |
| GHA | 2020 | DTU_2021_1011363_1_MG_GH_AC_861_S0_L001 | Sewage | ERR14146762 |
| GHA | 2020 | DTU_2021_1011364_1_MG_GH_TE_862_S0_L001 | Sewage | ERR14146766 |
| CRI | 2020 | DTU_2021_1011365_1_MG_CR_SJ_867_S118_L001 | Sewage | ERR14146777 |
| MYS | 2020 | DTU_2021_1011366_1_MG_MY_IH_898_S0_L001 | Sewage | ERR14146781 |
| MYS | 2019 | DTU_2021_1011367_1_MG_MY_PN_899_S0_L001 | Sewage | ERR14146784 |
| MYS | 2020 | DTU_2021_1011368_1_MG_MY_KL_900_S0_L001 | Sewage | ERR14146789 |
| MYS | 2020 | DTU_2021_1011369_1_MG_MY_PB_901_S0_L001 | Sewage | ERR14146793 |
| NZL | 2020 | DTU_2021_1011370_1_MG_NZ_DU_932_S30_L001 | Sewage | ERR14146797 |
| AUT | 2020 | DTU_2021_1011371_1_MG_AT_VI_768_S0_L001 | Sewage | ERR14146801 |
| SWE | 2020 | DTU_2021_1011372_1_MG_SE_GO_769_S213_L002 | Sewage | ERR14146806 |
| DZA | 2020 | DTU_2021_1011373_1_MG_DZ_BC_770_S0_L001 | Sewage | ERR14146808 |
| AUS | 2020 | DTU_2021_1011374_1_MG_AU_SY_1_771_S0_L001 | Sewage | ERR14146815 |
| AUS | 2020 | DTU_2021_1011375_1_MG_AU_SY_2_772_S0_L001 | Sewage | ERR14146821 |
| AUS | 2020 | DTU_2021_1011376_1_MG_AU_SY_3_773_S0_L001 | Sewage | ERR14146826 |
| AUS | 2020 | DTU_2021_1011377_1_MG_AU_SY_4_774_S0_L001 | Sewage | ERR14146828 |
| AUS | 2020 | DTU_2021_1011378_1_MG_AU_WO_1_775_S36_L001 | Sewage | ERR14146829 |
| AUS | 2020 | DTU_2021_1011379_1_MG_AU_WO_2_776_S0_L001 | Sewage | ERR14146834 |
| AUS | 2020 | DTU_2021_1011380_1_MG_AU_WO_3_777_S0_L001 | Sewage | ERR14146838 |
| KOR | 2020 | DTU_2021_1011381_1_MG_KR_DA_778_S214_L002 | Sewage | ERR14146842 |
| NOR | 2020 | DTU_2021_1011382_1_MG_NO_OS_779_S38_L001 | Sewage | ERR14146846 |
| SAU | 2020 | DTU_2021_1011383_1_MG_SA_KA_780_S0_L001 | Sewage | ERR14146847 |
| GBR | 2020 | DTU_2021_1011384_1_MG_GB_FA_781_S0_L001 | Sewage | ERR14146850 |
| GBR | 2020 | DTU_2021_1011385_1_MG_GB_CA_782_S0_L001 | Sewage | ERR14146852 |
| HRV | 2020 | DTU_2021_1011386_1_MG_HR_ZA_783_S0_L001 | Sewage | ERR14146855 |
| NGA | 2020 | DTU_2021_1011387_1_MG_NG_AB_2_784_S0_L001 | Sewage | ERR14146860 |
| NGA | 2020 | DTU_2021_1011388_1_MG_NG_NS_785_S0_L001 | Sewage | ERR14146864 |
| TWN | 2020 | DTU_2021_1011389_1_MG_TW_TA_786_S0_L001 | Sewage | ERR14146867 |
| DEU | 2020 | DTU_2021_1011390_1_MG_DE_DR_787_S0_L001 | Sewage | ERR14146869 |
| IRL | 2020 | DTU_2021_1011391_1_MG_IE_GA_788_S0_L001 | Sewage | ERR14146870 |
| CZE | 2020 | DTU_2021_1011392_1_MG_CZ_BR_789_S0_L001 | Sewage | ERR14146873 |
| CZE | 2020 | DTU_2021_1011393_1_MG_CZ_PR_790_S0_L001 | Sewage | ERR14146881 |
| FRA | 2020 | DTU_2021_1011394_1_MG_FR_NA_791_S0_L001 | Sewage | ERR14146885 |
| FRA | 2020 | DTU_2021_1011395_1_MG_FR_SA_792_S0_L001 | Sewage | ERR14146886 |
| MDA | 2020 | DTU_2021_1011396_1_MG_MD_OR_793_S0_L001 | Sewage | ERR14146890 |
| MDA | 2020 | DTU_2021_1011397_1_MG_MD_CA_794_S0_L001 | Sewage | ERR14146892 |
| MDA | 2020 | DTU_2021_1011398_1_MG_MD_BA_795_S0_L001 | Sewage | ERR14146898 |
| MDA | 2020 | DTU_2021_1011399_1_MG_MD_ED_796_S0_L001 | Sewage | ERR14146902 |
| MDA | 2020 | DTU_2021_1011400_1_MG_MD_CO_797_S0_L001 | Sewage | ERR14146905 |
| MDA | 2020 | DTU_2021_1011401_1_MG_MD_UN_798_S0_L001 | Sewage | ERR14146906 |
| MDA | 2020 | DTU_2021_1011402_1_MG_MD_CI_799_S0_L001 | Sewage | ERR14146908 |
| MDA | 2020 | DTU_2021_1011403_1_MG_MD_CH_800_S0_L001 | Sewage | ERR14146912 |
| MDA | 2020 | DTU_2021_1011404_1_MG_MD_HI_801_S0_L001 | Sewage | ERR14146918 |
| MDA | 2020 | DTU_2021_1011405_1_MG_MD_SO_802_S0_L001 | Sewage | ERR14146946 |
| USA | 2020 | DTU_2021_1011406_1_MG_US_BO_1_803_S0_L001 | Sewage | ERR14146948 |
| USA | 2020 | DTU_2021_1011407_1_MG_US_BO_2_804_S0_L001 | Sewage | ERR14146950 |
| BIH | 2020 | DTU_2021_1011408_1_MG_BA_BL_805_S0_L001 | Sewage | ERR14146952 |
| GRC | 2020 | DTU_2021_1011409_1_MG_GR_MA_806_S0_L001 | Sewage | ERR14146954 |
| SRB | 2020 | DTU_2021_1011410_1_MG_RS_BE_1_807_S0_L001 | Sewage | ERR14146958 |
| SRB | 2020 | DTU_2021_1011411_1_MG_RS_BE_2_808_S0_L001 | Sewage | ERR14146964 |
| SRB | 2020 | DTU_2021_1011412_1_MG_RS_NS_1_809_S16_L001 | Sewage | ERR14146967 |
| SRB | 2020 | DTU_2021_1011413_1_MG_RS_NS_2_810_S0_L001 | Sewage | ERR14146968 |
| SRB | 2020 | DTU_2021_1011414_1_MG_RS_SU_811_S0_L001 | Sewage | ERR14146972 |
| SRB | 2020 | DTU_2021_1011415_1_MG_RS_ZR_812_S0_L001 | Sewage | ERR14146976 |
| SRB | 2020 | DTU_2021_1011416_1_MG_RS_KR_813_S0_L001 | Sewage | ERR14146981 |
| ESP | 2020 | DTU_2021_1011417_1_MG_ES_MA_814_S0_L001 | Sewage | ERR14148161 |
| POL | 2020 | DTU_2021_1011418_1_MG_PL_PL_815_S0_L001 | Sewage | ERR14148750 |
| ZAF | 2020 | DTU_2021_1011419_1_MG_ZA_PR_816_S0_L001 | Sewage | ERR14149338 |
| SVN | 2020 | DTU_2021_1011420_1_MG_SI_LJ_817_S0_L001 | Sewage | ERR14149341 |
| PRT | 2020 | DTU_2021_1011421_1_MG_PT_LI_818_S62_L001 | Sewage | ERR14149344 |
| CHL | 2020 | DTU_2021_1011422_1_MG_CL_PE_819_S215_L002 | Sewage | ERR14149350 |
| CHL | 2020 | DTU_2021_1011423_1_MG_CL_BU_820_S203_L002 | Sewage | ERR14149355 |
| CHL | 2020 | DTU_2021_1011424_1_MG_CL_SA_1_821_S0_L001 | Sewage | ERR14149357 |
| CHL | 2020 | DTU_2021_1011425_1_MG_CL_SA_3_822_S0_L001 | Sewage | ERR14149358 |
| BWA | 2020 | DTU_2021_1011426_1_MG_BW_GA_823_S0_L001 | Sewage | ERR14149362 |
| GHA | 2020 | DTU_2021_1011427_1_MG_GH_TI_824_S0_L001 | Sewage | ERR14149363 |
| TUR | 2020 | DTU_2021_1011428_1_MG_TR_HA_825_S0_L001 | Sewage | ERR14149368 |
| CAN | 2020 | DTU_2021_1011429_1_MG_CA_OT_826_S23_L004 | Sewage | ERR14149374 |
| CAN | 2020 | DTU_2021_1011430_1_MG_CA_RE_827_S61_L004 | Sewage | ERR14149377 |
| CAN | 2020 | DTU_2021_1011432_1_MG_CA_DE_829_S62_L004 | Sewage | ERR14149381 |
| GRL | 2020 | DTU_2021_1011433_1_MG_GL_NU_830_S67_L004 | Sewage | ERR14149384 |
| CHE | 2020 | DTU_2021_1011434_1_MG_CH_BR_831_S43_L004 | Sewage | ERR14149388 |
| GRC | 2020 | DTU_2021_1011435_1_MG_GR_TH_833_S68_L004 | Sewage | ERR14149395 |
| BEN | 2020 | DTU_2021_1011436_1_MG_BJ_AC_835_S117_L003 | Sewage | ERR14149396 |
| HUN | 2020 | DTU_2021_1011437_1_MG_HU_BU_1_840_S31_L004 | Sewage | ERR14149398 |
| HUN | 2020 | DTU_2021_1011438_1_MG_HU_BU_2_841_S0_L001 | Sewage | ERR14149401 |
| HUN | 2020 | DTU_2021_1011439_1_MG_HU_BU_3_842_S2_L004 | Sewage | ERR14149404 |
| GHA | 2020 | DTU_2021_1011440_1_MG_GH_KU_843_S44_L004 | Sewage | ERR14149406 |
| ITA | 2020 | DTU_2021_1011441_1_MG_IT_AN_845_S0_L001 | Sewage | ERR14149412 |
| ITA | 2020 | DTU_2021_1011442_1_MG_IT_MA_846_S0_L001 | Sewage | ERR14149413 |
| ITA | 2020 | DTU_2021_1011443_1_MG_IT_FE_847_S13_L004 | Sewage | ERR14149416 |
| ITA | 2020 | DTU_2021_1011444_1_MG_IT_AP_848_S0_L001 | Sewage | ERR14149420 |
| NGA | 2020 | DTU_2021_1011445_1_MG_NG_IM_849_S3_L004 | Sewage | ERR14149423 |
| ITA | 2020 | DTU_2021_1011446_1_MG_IT_RO_850_S66_L004 | Sewage | ERR14149424 |
| AUT | 2020 | DTU_2021_1011447_1_MG_AT_GR_851_S45_L004 | Sewage | ERR14149429 |
| NGA | 2020 | DTU_2021_1011448_1_MG_NG_LA_852_S0_L001 | Sewage | ERR14149431 |
| ITA | 2020 | DTU_2021_1011449_1_MG_IT_NO_853_S118_L003 | Sewage | ERR14149435 |
| ITA | 2020 | DTU_2021_1011450_1_MG_IT_VE_854_S33_L004 | Sewage | ERR14149437 |
| ITA | 2020 | DTU_2021_1011451_1_MG_IT_CA_855_S46_L004 | Sewage | ERR14149442 |
| LVA | 2020 | DTU_2021_1011452_1_MG_LV_LI_856_S47_L004 | Sewage | ERR14149447 |
| LVA | 2020 | DTU_2021_1011453_1_MG_LV_RI_857_S48_L004 | Sewage | ERR14149450 |
| IND | 2020 | DTU_2021_1011454_1_MG_IN_KO_858_S49_L004 | Sewage | ERR14149452 |
| NGA | 2020 | DTU_2021_1011455_1_MG_NG_IL_859_S0_L001 | Sewage | ERR14149454 |
| EGY | 2020 | DTU_2021_1011456_1_MG_EG_KE_860_S50_L004 | Sewage | ERR14149460 |
| CMR | 2020 | DTU_2021_1011457_1_MG_CM_YA_863_S51_L004 | Sewage | ERR14149466 |
| TTO | 2020 | DTU_2021_1011458_1_MG_TT_SA_1_864_S34_L004 | Sewage | ERR14149468 |
| TTO | 2020 | DTU_2021_1011459_1_MG_TT_SA_2_865_S0_L001 | Sewage | ERR14149470 |
| SGP | 2020 | DTU_2021_1011460_1_MG_SG_JU_866_S52_L004 | Sewage | ERR14149472 |
| PRT | 2020 | DTU_2021_1011461_1_MG_PT_PO_868_S24_L004 | Sewage | ERR14149475 |
| NGA | 2021 | DTU_2021_1011462_1_MG_NG_MA_869_S0_L001 | Sewage | ERR14149482 |
| BGD | 2021 | DTU_2021_1011463_1_MG_BD_DH_870_S22_L004 | Sewage | ERR14149484 |
| ECU | 2020 | DTU_2021_1011464_1_MG_EC_QU_871_S25_L004 | Sewage | ERR14149488 |
| ECU | 2020 | DTU_2021_1011465_1_MG_EC_LA_872_S119_L003 | Sewage | ERR14149490 |
| ECU | 2020 | DTU_2021_1011466_1_MG_EC_AM_873_S53_L004 | Sewage | ERR14149492 |
| ECU | 2020 | DTU_2021_1011468_1_MG_EC_GY_875_S26_L004 | Sewage | ERR14149633 |
| MDG | 2020 | DTU_2021_1011469_1_MG_MG_AN_876_S4_L004 | Sewage | ERR14149635 |
| CAN | 2020 | DTU_2021_1011470_1_MG_CA_QE_877_S27_L004 | Sewage | ERR14149639 |
| CAN | 2020 | DTU_2021_1011471_1_MG_CA_GU_878_S69_L004 | Sewage | ERR14149645 |
| ARE | 2020 | DTU_2021_1011472_1_MG_AE_AD_879_S54_L004 | Sewage | ERR14149648 |
| ARE | 2020 | DTU_2021_1011473_1_MG_AE_DU_880_S55_L004 | Sewage | ERR14149651 |
| DNK | 2020 | DTU_2021_1011474_1_MG_DK_AV_881_S120_L003 | Sewage | ERR14149653 |
| DNK | 2020 | DTU_2021_1011475_1_MG_DK_VA_882_S0_L001 | Sewage | ERR14149655 |
| DNK | 2020 | DTU_2021_1011476_1_MG_DK_AM_883_S121_L003 | Sewage | ERR14149663 |
| COL | 2020 | DTU_2021_1011477_1_MG_CO_MO_1_887_S19_L004 | Sewage | ERR14149665 |
| GBR | 2020 | DTU_2021_1011478_1_MG_GB_OX_889_S0_L001 | Sewage | ERR14149667 |
| SVK | 2020 | DTU_2021_1011479_1_MG_SK_BR_890_S70_L004 | Sewage | ERR14149669 |
| CIV | 2020 | DTU_2021_1011480_1_MG_CI_AB_891_S28_L004 | Sewage | ERR14149671 |
| CIV | 2020 | DTU_2021_1011481_1_MG_CI_YA_1_892_S0_L001 | Sewage | ERR14149676 |
| CIV | 2020 | DTU_2021_1011482_1_MG_CI_YA_2_893_S0_L001 | Sewage | ERR14149680 |
| CIV | 2020 | DTU_2021_1011483_1_MG_CI_BO_1_894_S7_L004 | Sewage | ERR14149684 |
| MEX | 2020 | DTU_2021_1011485_1_MG_MX_QU_896_S29_L004 | Sewage | ERR14149686 |
| MLT | 2021 | DTU_2021_1011486_1_MG_MT_XG_897_S0_L001 | Sewage | ERR14149688 |
| MWI | 2020 | DTU_2021_1011487_1_MG_MW_BL_902_S56_L004 | Sewage | ERR14149693 |
| MWI | 2020 | DTU_2021_1011488_1_MG_MW_ZO_903_S57_L004 | Sewage | ERR14149697 |
| MWI | 2020 | DTU_2021_1011489_1_MG_MW_MZ_904_S58_L004 | Sewage | ERR14149699 |
| MWI | 2020 | DTU_2021_1011490_1_MG_MW_KH_905_S59_L004 | Sewage | ERR14149700 |
| MWI | 2020 | DTU_2021_1011491_1_MG_MW_MA_906_S8_L004 | Sewage | ERR14149704 |
| FIN | 2020 | DTU_2021_1011492_1_MG_FI_HE_933_S63_L004 | Sewage | ERR14149707 |
| ITA | 2020 | DTU_2021_1011493_1_MG_IT_BO_934_S0_L001 | Sewage | ERR14149710 |
| ITA | 2020 | DTU_2021_1011494_1_MG_IT_CO_935_S0_L001 | Sewage | ERR14149713 |
| ITA | 2020 | DTU_2021_1011495_1_MG_IT_CR_936_S38_L004 | Sewage | ERR14149714 |
| ITA | 2020 | DTU_2021_1011496_1_MG_IT_BE_937_S39_L004 | Sewage | ERR14149716 |
| LUX | 2020 | DTU_2021_1011497_1_MG_LU_SC_938_S0_L001 | Sewage | ERR14149719 |
| LUX | 2020 | DTU_2021_1011498_1_MG_LU_BE_939_S0_L001 | Sewage | ERR14149724 |
| ESP | 2020 | DTU_2021_1011499_1_MG_ES_BA_940_S42_L004 | Sewage | ERR14149726 |
| USA | 2020 | DTU_2021_1011500_1_MG_US_CB_941_S30_L004 | Sewage | ERR14149729 |
| BRB | 2020 | DTU_2021_1011501_1_MG_BB_BR_949_S60_L004 | Sewage | ERR14149731 |
| BRB | 2020 | DTU_2021_1011502_1_MG_BB_WO_950_S64_L004 | Sewage | ERR14149735 |
| DZA | 2021 | DTU_2022_1024401_1_MG_DZ_BC_907_S0_L001 | Sewage | ERR14173444 |
| ZAF | 2021 | DTU_2022_1024402_1_MG_ZA_PR_908_S0_L001 | Sewage | ERR14173442 |
| KOR | 2021 | DTU_2022_1024403_1_MG_KR_DA_909_S0_L001 | Sewage | ERR14173445 |
| CZE | 2021 | DTU_2022_1024404_1_MG_CZ_BR_910_S0_L001 | Sewage | ERR14173441 |
| CZE | 2021 | DTU_2022_1024405_1_MG_CZ_PR_911_S0_L001 | Sewage | ERR14173455 |
| AUT | 2021 | DTU_2022_1024406_1_MG_AT_VI_912_S0_L001 | Sewage | ERR14173456 |
| MDA | 2021 | DTU_2022_1024407_1_MG_MD_CH_913_S0_L001 | Sewage | ERR14173440 |
| MDA | 2021 | DTU_2022_1024409_1_MG_MD_CO_915_S8_L001 | Sewage | ERR14173448 |
| MDA | 2021 | DTU_2022_1024410_1_MG_MD_ED_916_S0_L001 | Sewage | ERR14173451 |
| MDA | 2021 | DTU_2022_1024411_1_MG_MD_HI_917_S0_L001 | Sewage | ERR14173443 |
| MDA | 2021 | DTU_2022_1024412_1_MG_MD_OR_918_S0_L001 | Sewage | ERR14173461 |
| MDA | 2021 | DTU_2022_1024413_1_MG_MD_SO_919_S0_L001 | Sewage | ERR14173463 |
| MDA | 2021 | DTU_2022_1024414_1_MG_MD_UN_920_S0_L001 | Sewage | ERR14173473 |
| MDA | 2021 | DTU_2022_1024416_1_MG_MD_BA_922_S0_L001 | Sewage | ERR14173446 |
| IRL | 2021 | DTU_2022_1024417_1_MG_IE_GA_923_S0_L001 | Sewage | ERR14173449 |
| HRV | 2021 | DTU_2022_1024418_1_MG_HR_ZA_925_S0_L001 | Sewage | ERR14173471 |
| FRA | 2021 | DTU_2022_1024419_1_MG_FR_NA_926_S0_L001 | Sewage | ERR14173472 |
| FRA | 2021 | DTU_2022_1024420_1_MG_FR_CR_927_S0_L001 | Sewage | ERR14173447 |
| ESP | 2021 | DTU_2022_1024421_1_MG_ES_MA_928_S0_L001 | Sewage | ERR14173474 |
| BRA | 2021 | DTU_2022_1024422_1_MG_BR_BH_929_S0_L001 | Sewage | ERR14173465 |
| TWN | 2021 | DTU_2022_1024423_1_MG_TW_TA_930_S21_L001 | Sewage | ERR14173454 |
| CHE | 2021 | DTU_2022_1024424_1_MG_CH_BR_931_S22_L001 | Sewage | ERR14173464 |
| BIH | 2021 | DTU_2022_1024425_1_MG_BA_BL_942_S0_L001 | Sewage | ERR14173460 |
| SVN | 2021 | DTU_2022_1024426_1_MG_SI_LJ_943_S0_L001 | Sewage | ERR14173462 |
| CRI | 2021 | DTU_2022_1024427_1_MG_CR_LB_944_S25_L001 | Sewage | ERR14173450 |
| CRI | 2021 | DTU_2022_1024428_1_MG_CR_LI_945_S0_L001 | Sewage | ERR14173457 |
| CRI | 2021 | DTU_2022_1024429_1_MG_CR_SG_946_S0_L001 | Sewage | ERR14173469 |
| CRI | 2021 | DTU_2022_1024430_1_MG_CR_PU_947_S0_L001 | Sewage | ERR14173470 |
| CRI | 2021 | DTU_2022_1024431_1_MG_CR_SJ_948_S29_L001 | Sewage | ERR14173453 |
| NGA | 2021 | DTU_2022_1024432_1_MG_NG_AB_951_S0_L001 | Sewage | ERR14173466 |
| ITA | 2021 | DTU_2022_1024433_1_MG_IT_PE_952_S31_L001 | Sewage | ERR14173467 |
| ITA | 2021 | DTU_2022_1024434_1_MG_IT_AN_953_S0_L001 | Sewage | ERR14173490 |
| ITA | 2021 | DTU_2022_1024435_1_MG_IT_MA_954_S33_L001 | Sewage | ERR14173452 |
| ITA | 2021 | DTU_2022_1024436_1_MG_IT_FE_955_S0_L001 | Sewage | ERR14173459 |
| ITA | 2021 | DTU_2022_1024437_1_MG_IT_BO_957_S35_L001 | Sewage | ERR14173468 |
| LUX | 2021 | DTU_2022_1024438_1_MG_LU_CH_958_S36_L001 | Sewage | ERR14173475 |
| LUX | 2021 | DTU_2022_1024439_1_MG_LU_BE_959_S0_L001 | Sewage | ERR14173477 |
| HUN | 2021 | DTU_2022_1024440_1_MG_HU_BU_1_960_S93_L002 | Sewage | ERR14173597 |
| HUN | 2021 | DTU_2022_1024441_1_MG_HU_BU_2_961_S0_L001 | Sewage | ERR14173476 |
| HUN | 2021 | DTU_2022_1024442_1_MG_HU_BU_3_962_S0_L001 | Sewage | ERR14173480 |
| USA | 2021 | DTU_2022_1024443_1_MG_WA_SE_963_S40_L001 | Sewage | ERR14173528 |
| USA | 2021 | DTU_2022_1024444_1_MG_WA_WO_964_S41_L001 | Sewage | ERR14173496 |
| ESP | 2021 | DTU_2022_1024445_1_MG_ES_BA_965_S42_L001 | Sewage | ERR14173478 |
| SWE | 2021 | DTU_2022_1024446_1_MG_SE_GO_966_S0_L001 | Sewage | ERR14173479 |
| ISL | 2021 | DTU_2022_1024447_1_MG_IS_RE_967_S0_L001 | Sewage | ERR14173486 |
| SAU | 2021 | DTU_2022_1024448_1_MG_SA_KA_968_S0_L001 | Sewage | ERR14173493 |
| USA | 2021 | DTU_2022_1024449_1_MG_WI_SM_969_S46_L001 | Sewage | ERR14173559 |
| GHA | 2021 | DTU_2022_1024450_1_MG_GH_TA_970_S0_L001 | Sewage | ERR14173481 |
| POL | 2021 | DTU_2022_1024451_1_MG_PL_PL_971_S48_L001 | Sewage | ERR14173487 |
| LVA | 2021 | DTU_2022_1024452_1_MG_LV_RI_972_S0_L001 | Sewage | ERR14173482 |
| LVA | 2021 | DTU_2022_1024453_1_MG_LV_LI_973_S50_L001 | Sewage | ERR14173488 |
| DNK | 2021 | DTU_2022_1024454_1_MG_DK_AV_974_S0_L001 | Sewage | ERR14173483 |
| DNK | 2021 | DTU_2022_1024455_1_MG_DK_VA_975_S52_L001 | Sewage | ERR14173485 |
| DNK | 2021 | DTU_2022_1024456_1_MG_DK_AM_976_S0_L001 | Sewage | ERR14173569 |
| AUS | 2021 | DTU_2022_1024457_1_MG_AU_SY_1_977_S54_L001 | Sewage | ERR14173515 |
| AUS | 2021 | DTU_2022_1024458_1_MG_AU_SY_2_978_S0_L001 | Sewage | ERR14173484 |
| AUS | 2021 | DTU_2022_1024459_1_MG_AU_SY_3_979_S0_L001 | Sewage | ERR14173492 |
| AUS | 2021 | DTU_2022_1024460_1_MG_AU_SY_4_980_S57_L001 | Sewage | ERR14173489 |
| AUS | 2021 | DTU_2022_1024461_1_MG_AU_WO_1_981_S58_L001 | Sewage | ERR14173527 |
| AUS | 2021 | DTU_2022_1024462_1_MG_AU_WO_2_982_S0_L001 | Sewage | ERR14173572 |
| AUS | 2021 | DTU_2022_1024463_1_MG_AU_WO_3_983_S0_L001 | Sewage | ERR14173539 |
| AUS | 2021 | DTU_2022_1024464_1_MG_AU_ME_984_S0_L001 | Sewage | ERR14173504 |
| USA | 2021 | DTU_2022_1024465_1_MG_VI_CH_985_S62_L001 | Sewage | ERR14173581 |
| USA | 2021 | DTU_2022_1024466_1_MG_VI_BL_986_S0_L001 | Sewage | ERR14173602 |
| NGA | 2021 | DTU_2022_1024467_1_MG_NG_LA_987_S64_L001 | Sewage | ERR14173495 |
| FIN | 2021 | DTU_2022_1024468_1_MG_FI_KU_988_S0_L001 | Sewage | ERR14173546 |
| COL | 2021 | DTU_2022_1024469_1_MG_CO_BO_989_S0_L001 | Sewage | ERR14173591 |
| SRB | 2021 | DTU_2022_1024470_1_MG_RS_BE_1_990_S0_L001 | Sewage | ERR14173571 |
| SRB | 2021 | DTU_2022_1024471_1_MG_RS_BE_2_991_S0_L001 | Sewage | ERR14173511 |
| SRB | 2021 | DTU_2022_1024475_1_MG_RS_SU_996_S0_L001 | Sewage | ERR14173531 |
| SRB | 2021 | DTU_2022_1024476_1_MG_RS_ZR_997_S70_L001 | Sewage | ERR14173544 |
| TUR | 2021 | DTU_2022_1024477_1_MG_TR_HA_998_S0_L001 | Sewage | ERR14173499 |
| BEN | 2021 | DTU_2022_1024478_1_MG_BJ_CO_999_S72_L001 | Sewage | ERR14173557 |
| BEN | 2021 | DTU_2022_1024479_1_MG_BJ_AC_1000_S0_L001 | Sewage | ERR14173555 |
| BEN | 2021 | DTU_2022_1024480_1_MG_BJ_SK_1001_S0_L001 | Sewage | ERR14173560 |
| DEU | 2021 | DTU_2022_1024481_1_MG_DE_DR_1002_S75_L001 | Sewage | ERR14173548 |
| BGD | 2021 | DTU_2022_1024482_1_MG_BD_DH_1003_S0_L001 | Sewage | ERR14173517 |
| CIV | 2021 | DTU_2022_1024483_1_MG_CI_BO_1004_S0_L001 | Sewage | ERR14173585 |
| CIV | 2021 | DTU_2022_1024484_1_MG_CI_BO_2_1005_S78_L001 | Sewage | ERR14173575 |
| CIV | 2021 | DTU_2022_1024485_1_MG_CI_AB_1006_S0_L001 | Sewage | ERR14173583 |
| CIV | 2021 | DTU_2022_1024486_1_MG_CI_YA_2_1008_S0_L001 | Sewage | ERR14173536 |
| LBN | 2021 | DTU_2022_1024487_1_MG_LB_CH_1009_S0_L001 | Sewage | ERR14173564 |
| LBN | 2021 | DTU_2022_1024488_1_MG_LB_QA_1010_S0_L001 | Sewage | ERR14173566 |
| LBN | 2021 | DTU_2022_1024489_1_MG_LB_BI_1_1012_S0_L001 | Sewage | ERR14173542 |
| LBN | 2021 | DTU_2022_1024490_1_MG_LB_BI_2_1013_S0_L001 | Sewage | ERR14173623 |
| CAN | 2021 | DTU_2022_1024491_1_MG_CA_VA_1014_S0_L001 | Sewage | ERR14173593 |
| CAN | 2021 | DTU_2022_1024492_1_MG_CA_CA_1015_S0_L001 | Sewage | ERR14173600 |
| CAN | 2021 | DTU_2022_1024493_1_MG_CA_RE_1016_S0_L001 | Sewage | ERR14173582 |
| CAN | 2021 | DTU_2022_1024494_1_MG_CA_OT_1017_S0_L001 | Sewage | ERR14173547 |
| NOR | 2021 | DTU_2022_1024495_1_MG_NO_OS_1018_S0_L001 | Sewage | ERR14173562 |
| GBR | 2021 | DTU_2022_1024496_1_MG_GB_FA_1019_S0_L001 | Sewage | ERR14173576 |
| GBR | 2021 | DTU_2022_1024497_1_MG_GB_CA_1020_S0_L001 | Sewage | ERR14173552 |
| USA | 2021 | DTU_2022_1024498_1_MG_US_BO_1021_S0_L001 | Sewage | ERR14173607 |
| USA | 2021 | DTU_2022_1024499_1_MG_US_BO_1022_S0_L001 | Sewage | ERR14173579 |
| ARE | 2021 | DTU_2022_1024500_1_MG_AE_AD_1023_S0_L001 | Sewage | ERR14173574 |
| ARE | 2021 | DTU_2022_1024501_1_MG_AE_DU_1024_S95_L002 | Sewage | ERR14173595 |
| AUT | 2021 | DTU_2022_1024502_1_MG_AT_GR_1026_S0_L001 | Sewage | ERR14173567 |
| ITA | 2021 | DTU_2022_1024503_1_MG_IT_NO_1027_S0_L001 | Sewage | ERR14173624 |
| ITA | 2021 | DTU_2022_1024504_1_MG_IT_VE_1028_S0_L001 | Sewage | ERR14173633 |
| ITA | 2021 | DTU_2022_1024505_1_MG_IT_CA_1029_S0_L001 | Sewage | ERR14173612 |
| MDG | 2021 | DTU_2022_1024506_1_MG_MG_AN_1030_S0_L001 | Sewage | ERR14173654 |
| CHN | 2021 | DTU_2022_1024507_1_MG_CN_NA_1031_S0_L001 | Sewage | ERR14173643 |
| CHN | 2021 | DTU_2022_1024508_1_MG_CN_SU_1032_S0_L001 | Sewage | ERR14173672 |
| IND | 2021 | DTU_2022_1024509_1_MG_IN_KO_1035_S0_L001 | Sewage | ERR14173601 |
| MWI | 2021 | DTU_2022_1024510_1_MG_MW_CO_1036_S0_L001 | Sewage | ERR14173675 |
| MWI | 2021 | DTU_2022_1024511_1_MG_MW_LI_1037_S105_L002 | Sewage | ERR14173625 |
| MWI | 2021 | DTU_2022_1024512_1_MG_MW_MC_1038_S106_L002 | Sewage | ERR14173608 |
| MWI | 2021 | DTU_2022_1024513_1_MG_MW_NI_1039_S0_L001 | Sewage | ERR14173685 |
| MWI | 2021 | DTU_2022_1024514_1_MG_MW_KA_1040_S0_L001 | Sewage | ERR14173670 |
| MWI | 2021 | DTU_2022_1024515_1_MG_MW_NB_1041_S0_L001 | Sewage | ERR14173656 |
| SVK | 2021 | DTU_2022_1024516_1_MG_SK_BR_1042_S0_L001 | Sewage | ERR14173621 |
| ITA | 2021 | DTU_2022_1024517_1_MG_IT_RO_1045_S0_L001 | Sewage | ERR14173619 |
| KOR | 2021 | DTU_2022_1024518_1_MG_KR_DA_1046_S0_L001 | Sewage | ERR14173666 |
| NGA | 2021 | DTU_2022_1024519_1_MG_NG_AB_1047_S0_L001 | Sewage | ERR14173664 |
| ITA | 2021 | DTU_2022_1024520_1_MG_IT_BO_1048_S114_L002 | Sewage | ERR14173628 |
| DZA | 2021 | DTU_2022_1024521_1_MG_DZ_BC_1049_S0_L001 | Sewage | ERR14173630 |
| FIN | 2021 | DTU_2022_1024522_1_MG_FI_HE_1050_S0_L001 | Sewage | ERR14173635 |
| MDA | 2021 | DTU_2022_1024523_1_MG_MD_BA_1051_S0_L001 | Sewage | ERR14173697 |
| MDA | 2021 | DTU_2022_1024524_1_MG_MD_CA_1052_S0_L001 | Sewage | ERR14173637 |
| MDA | 2021 | DTU_2022_1024525_1_MG_MD_CI_1053_S0_L001 | Sewage | ERR14173638 |
| MDA | 2021 | DTU_2022_1024526_1_MG_MD_CH_1054_S0_L001 | Sewage | ERR14173663 |
| MDA | 2021 | DTU_2022_1024527_1_MG_MD_CO_1055_S0_L001 | Sewage | ERR14173642 |
| MDA | 2021 | DTU_2022_1024528_1_MG_MD_ED_1056_S0_L001 | Sewage | ERR14173661 |
| MDA | 2021 | DTU_2022_1024529_1_MG_MD_HI_1057_S0_L001 | Sewage | ERR14173645 |
| MDA | 2021 | DTU_2022_1024530_1_MG_MD_OR_1058_S0_L001 | Sewage | ERR14173678 |
| MDA | 2021 | DTU_2022_1024531_1_MG_MD_SO_1059_S0_L001 | Sewage | ERR14173659 |
| MDA | 2021 | DTU_2022_1024532_1_MG_MD_UN_1060_S0_L001 | Sewage | ERR14173667 |
| SAU | 2021 | DTU_2022_1024533_1_MG_SA_KA_1061_S0_L001 | Sewage | ERR14173636 |
| ESP | 2021 | DTU_2022_1024534_1_MG_ES_MA_1062_S0_L001 | Sewage | ERR14173719 |
| AUS | 2021 | DTU_2022_1024535_1_MG_AU_SY_1_1063_S0_L001 | Sewage | ERR14173648 |
| AUS | 2021 | DTU_2022_1024536_1_MG_AU_SY_2_1064_S0_L001 | Sewage | ERR14173671 |
| AUS | 2021 | DTU_2022_1024537_1_MG_AU_SY_3_1065_S0_L001 | Sewage | ERR14173652 |
| AUS | 2021 | DTU_2022_1024538_1_MG_AU_SY_4_1066_S0_L001 | Sewage | ERR14173647 |
| AUS | 2021 | DTU_2022_1024539_1_MG_AU_WO_1_1067_S0_L001 | Sewage | ERR14173688 |
| AUS | 2021 | DTU_2022_1024540_1_MG_AU_WO_2_1068_S0_L001 | Sewage | ERR14173703 |
| AUS | 2021 | DTU_2022_1024541_1_MG_AU_WO_3_1069_S0_L001 | Sewage | ERR14173651 |
| GBR | 2021 | DTU_2022_1024542_1_MG_GB_FA_1071_S0_L001 | Sewage | ERR14173682 |
| IRL | 2021 | DTU_2022_1024543_1_MG_IE_GA_1072_S0_L001 | Sewage | ERR14173673 |
| MLT | 2021 | DTU_2022_1024544_1_MG_MT_LU_1073_S0_L001 | Sewage | ERR14173683 |
| IRQ | 2021 | DTU_2022_1024545_1_MG_IQ_HA_1074_S0_L001 | Sewage | ERR14173674 |
| IRQ | 2021 | DTU_2022_1024546_1_MG_IQ_SU_1075_S0_L001 | Sewage | ERR14173665 |
| HRV | 2021 | DTU_2022_1024548_1_MG_HR_ZA_1077_S0_L001 | Sewage | ERR14173695 |
| AUS | 2021 | DTU_2022_1024549_1_MG_AU_ME_1078_S0_L001 | Sewage | ERR14173677 |
| FRA | 2021 | DTU_2022_1024550_1_MG_FR_NA_1079_S0_L001 | Sewage | ERR14173679 |
| FRA | 2021 | DTU_2022_1024551_1_MG_FR_SA_1080_S0_L001 | Sewage | ERR14173684 |
| GRC | 2021 | DTU_2022_1024552_1_MG_GR_MA_1081_S145_L002 | Sewage | ERR14173668 |
| TWN | 2021 | DTU_2022_1024553_1_MG_TW_TA_1082_S0_L001 | Sewage | ERR14173691 |
| NGA | 2021 | DTU_2022_1024554_1_MG_NG_JO_1083_S0_L001 | Sewage | ERR14173669 |
| FIN | 2021 | DTU_2022_1024555_1_MG_FI_KU_1084_S0_L001 | Sewage | ERR14173687 |
| GBR | 2021 | DTU_2022_1024556_1_MG_GB_ED_1085_S0_L001 | Sewage | ERR14173738 |
| GBR | 2021 | DTU_2022_1024557_1_MG_GB_GL_1086_S0_L001 | Sewage | ERR14173717 |
| ITA | 2021 | DTU_2022_1024558_1_MG_IT_AN_1087_S0_L001 | Sewage | ERR14173676 |
| ITA | 2021 | DTU_2022_1024559_1_MG_IT_AP_1088_S0_L001 | Sewage | ERR14173681 |
| ITA | 2021 | DTU_2022_1024560_1_MG_IT_FE_1089_S153_L002 | Sewage | ERR14173686 |
| ITA | 2021 | DTU_2022_1024561_1_MG_IT_MA_1090_S0_L001 | Sewage | ERR14173696 |
| LUX | 2021 | DTU_2022_1024562_1_MG_LU_BE_1092_S0_L001 | Sewage | ERR14173698 |
| LUX | 2021 | DTU_2022_1024563_1_MG_LU_CH_1093_S0_L001 | Sewage | ERR14173680 |
| AUT | 2021 | DTU_2022_1024564_1_MG_AT_VI_1094_S0_L001 | Sewage | ERR14173693 |
| BGD | 2021 | DTU_2022_1024565_1_MG_BD_DH_1095_S0_L001 | Sewage | ERR14173690 |
| PRT | 2021 | DTU_2022_1024566_1_MG_PT_LI_1096_S0_L001 | Sewage | ERR14173716 |
| GRC | 2021 | DTU_2022_1024567_1_MG_GR_TH_1097_S0_L001 | Sewage | ERR14173692 |
| TTO | 2021 | DTU_2022_1024568_1_MG_TT_SA_1_1098_S0_L001 | Sewage | ERR14173711 |
| ESP | 2021 | DTU_2022_1024569_1_MG_ES_AL_1100_S0_L001 | Sewage | ERR14173713 |
| ESP | 2021 | DTU_2022_1024571_1_MG_ES_CI_1102_S0_L001 | Sewage | ERR14173702 |
| ESP | 2021 | DTU_2022_1024572_1_MG_ES_SP_1103_S0_L001 | Sewage | ERR14173705 |
| POL | 2021 | DTU_2022_1024573_1_MG_PL_PL_1104_S0_L001 | Sewage | ERR14173725 |
| DNK | 2021 | DTU_2022_1024574_1_MG_DK_AV_1105_S0_L001 | Sewage | ERR14173689 |
| DNK | 2021 | DTU_2022_1024575_1_MG_DK_VA_1106_S168_L002 | Sewage | ERR14173707 |
| DNK | 2021 | DTU_2022_1024576_1_MG_DK_AM_1107_S169_L002 | Sewage | ERR14173718 |
| CHN | 2021 | DTU_2022_1024577_1_MG_CN_NA_1108_S0_L001 | Sewage | ERR14173710 |
| CHN | 2021 | DTU_2022_1024578_1_MG_CN_SU_1109_S171_L002 | Sewage | ERR14173712 |
| LVA | 2021 | DTU_2022_1024579_1_MG_LV_LI_1110_S0_L001 | Sewage | ERR14173708 |
| LVA | 2021 | DTU_2022_1024580_1_MG_LV_RI_1111_S0_L001 | Sewage | ERR14173701 |
| ISL | 2021 | DTU_2022_1024581_1_MG_IS_RE_1112_S174_L002 | Sewage | ERR14173700 |
| SRB | 2021 | DTU_2022_1024582_1_MG_RS_BE_1113_S175_L002 | Sewage | ERR14173706 |
| SRB | 2021 | DTU_2022_1024583_1_MG_RS_BE_2_1114_S0_L001 | Sewage | ERR14173704 |
| SRB | 2021 | DTU_2022_1024585_1_MG_RS_SU_1117_S0_L001 | Sewage | ERR14173749 |
| MUS | 2021 | DTU_2022_1024586_1_MG_MU_SM_1119_S0_L001 | Sewage | ERR14173722 |
| BEN | 2021 | DTU_2022_1024587_1_MG_BJ_AC_1120_S180_L002 | Sewage | ERR14173699 |
| BEN | 2021 | DTU_2022_1024588_1_MG_BJ_AG_1121_S0_L001 | Sewage | ERR14173727 |
| BEN | 2021 | DTU_2022_1024589_1_MG_BJ_CO_1122_S182_L002 | Sewage | ERR14173733 |
| AUT | 2021 | DTU_2022_1024590_1_MG_AT_GR_1123_S0_L001 | Sewage | ERR14173715 |
| NGA | 2021 | DTU_2022_1024591_1_MG_NG_LA_1124_S184_L002 | Sewage | ERR14173723 |
| SWE | 2021 | DTU_2022_1024592_1_MG_SE_GO_1125_S0_L001 | Sewage | ERR14173709 |
| CIV | 2021 | DTU_2022_1024593_1_MG_CI_AB_1126_S259_L004 | Sewage | ERR14173729 |
| CIV | 2021 | DTU_2022_1024594_1_MG_CI_BO_2_1128_S260_L004 | Sewage | ERR14173764 |
| CIV | 2021 | DTU_2022_1024595_1_MG_CI_YA_1_1129_S261_L004 | Sewage | ERR14173730 |
| CIV | 2021 | DTU_2022_1024596_1_MG_CI_YA_2_1130_S262_L004 | Sewage | ERR14173752 |
| SGP | 2021 | DTU_2022_1024597_1_MG_SG_JU_1131_S263_L004 | Sewage | ERR14173743 |
| CHE | 2021 | DTU_2022_1024598_1_MG_CH_BR_1132_S264_L004 | Sewage | ERR14173758 |
| CRI | 2021 | DTU_2022_1024599_1_MG_CR_LB_1133_S0_L001 | Sewage | ERR14173759 |
| CRI | 2021 | DTU_2022_1024600_1_MG_CR_LI_1134_S0_L001 | Sewage | ERR14173721 |
| CRI | 2021 | DTU_2022_1024601_1_MG_CR_PU_1135_S267_L004 | Sewage | ERR14173731 |
| CRI | 2021 | DTU_2022_1024602_1_MG_CR_SG_1136_S268_L004 | Sewage | ERR14173726 |
| CRI | 2021 | DTU_2022_1024603_1_MG_CR_SJ_1137_S269_L004 | Sewage | ERR14173724 |
| LBN | 2021 | DTU_2022_1024604_1_MG_LB_AA_1139_S270_L004 | Sewage | ERR14173760 |
| LBN | 2021 | DTU_2022_1024605_1_MG_LB_HR_1140_S271_L004 | Sewage | ERR14173748 |
| LBN | 2021 | DTU_2022_1024606_1_MG_LB_CM_1141_S272_L004 | Sewage | ERR14173735 |
| BHR | 2021 | DTU_2022_1024607_1_MG_BH_MA_1142_S273_L004 | Sewage | ERR14173720 |
| GHA | 2021 | DTU_2022_1024608_1_MG_GH_KU_1143_S274_L004 | Sewage | ERR14173753 |
| ITA | 2021 | DTU_2022_1024609_1_MG_IT_CA_1144_S275_L004 | Sewage | ERR14173739 |
| ITA | 2021 | DTU_2022_1024610_1_MG_IT_NO_1145_S276_L004 | Sewage | ERR14173741 |
| ITA | 2021 | DTU_2022_1024611_1_MG_IT_VE_1146_S277_L004 | Sewage | ERR14173742 |
| IND | 2021 | DTU_2022_1024612_1_MG_IN_KO_1147_S0_L001 | Sewage | ERR14173768 |
| HUN | 2021 | DTU_2022_1024613_1_MG_HU_BU_3_1148_S0_L001 | Sewage | ERR14173734 |
| USA | 2021 | DTU_2022_1024614_1_MG_WA_RE_1149_S280_L004 | Sewage | ERR14173737 |
| USA | 2021 | DTU_2022_1024615_1_MG_WA_SE_1150_S281_L004 | Sewage | ERR14173761 |
| USA | 2021 | DTU_2022_1024616_1_MG_WA_WO_1151_S282_L004 | Sewage | ERR14173756 |
| USA | 2021 | DTU_2022_1024617_1_MG_WA_RE_1152_S283_L004 | Sewage | ERR14173740 |
| CAN | 2021 | DTU_2022_1024618_1_MG_CA_CA_1153_S284_L004 | Sewage | ERR14173728 |
| CAN | 2021 | DTU_2022_1024619_1_MG_CA_HA_1154_S285_L004 | Sewage | ERR14173773 |
| CAN | 2021 | DTU_2022_1024620_1_MG_CA_OT_1155_S286_L004 | Sewage | ERR14173732 |
| CAN | 2021 | DTU_2022_1024621_1_MG_CA_RE_1156_S287_L004 | Sewage | ERR14173750 |
| CAN | 2021 | DTU_2022_1024622_1_MG_CA_VA_1157_S0_L001 | Sewage | ERR14173736 |
| USA | 2021 | DTU_2022_1024623_1_MG_VI_BL_1158_S289_L004 | Sewage | ERR14173746 |
| USA | 2021 | DTU_2022_1024624_1_MG_VI_CH_1159_S290_L004 | Sewage | ERR14173778 |
| MNE | 2021 | DTU_2022_1024625_1_MG_ME_PO_1160_S291_L004 | Sewage | ERR14173769 |
| ARE | 2021 | DTU_2022_1024626_1_MG_AE_AD_1161_S292_L004 | Sewage | ERR14173774 |
| NOR | 2021 | DTU_2022_1024627_1_MG_NO_OS_1162_S293_L004 | Sewage | ERR14173745 |
| DEU | 2021 | DTU_2022_1024628_1_MG_DE_DR_1163_S294_L004 | Sewage | ERR14173751 |
| ESP | 2021 | DTU_2022_1024629_1_MG_ES_BA_1164_S295_L004 | Sewage | ERR14173763 |
| BRB | 2021 | DTU_2022_1024630_1_MG_BB_BR_1165_S296_L004 | Sewage | ERR14173767 |
| BRB | 2021 | DTU_2022_1024631_1_MG_BB_WO_1166_S297_L004 | Sewage | ERR14173744 |
| MEX | 2021 | DTU_2022_1024632_1_MG_MX_QU_1167_S298_L004 | Sewage | ERR14173755 |
| CAN | 2021 | DTU_2022_1024633_1_MG_CA_QE_1168_S299_L004 | Sewage | ERR14173747 |
| CAN | 2021 | DTU_2022_1024634_1_MG_CA_QE_1169_S300_L004 | Sewage | ERR14173771 |
| ZAF | 2021 | DTU_2022_1024635_1_MG_ZA_PR_1170_S301_L004 | Sewage | ERR14173777 |
| MDG | 2021 | DTU_2022_1024636_1_MG_MG_AN_1171_S302_L004 | Sewage | ERR14173775 |
| NZL | 2021 | DTU_2022_1024637_1_MG_NZ_CH_1172_S303_L004 | Sewage | ERR14173776 |
| DEU | 2021 | DTU_2022_1024638_1_MG_DE_BE_1173_S304_L004 | Sewage | ERR14173770 |
| PRT | 2020 | DTU_2022_1024639_1_MG_PT_PE_1025_S305_L004 | Sewage | ERR14173754 |
| PRT | 2020 | DTU_2022_1024640_1_MG_PT_LO_1033_S306_L004 | Sewage | ERR14173762 |
| NZL | 2020 | DTU_2022_1024641_1_MG_NZ_CH_1034_S307_L004 | Sewage | ERR14173765 |
| CHN | 2020 | DTU_2022_1024642_1_MG_CN_NA_1043_S308_L004 | Sewage | ERR14173766 |
| CHN | 2020 | DTU_2022_1024643_1_MG_CN_SU_1044_S309_L004 | Sewage | ERR14173757 |
| HRV | 2020 | DTU_2020_TWIW_01_HRV_ZAG_001 | Respiratory System | ERR14129313 |
| HRV | 2020 | DTU_2020_TWIW_01_HRV_ZAG_001 | Respiratory System | ERR10441505 |
| HRV | 2020 | DTU_2020_TWIW_01_HRV_ZAG_002 | Respiratory System | ERR10441508 |
| HRV | 2020 | DTU_2020_TWIW_01_HRV_ZAG_003 | Respiratory System | ERR10441543 |
| HRV | 2020 | DTU_2020_TWIW_01_HRV_ZAG_003 | Respiratory System | ERR14129314 |
| HRV | 2020 | DTU_2020_TWIW_01_HRV_ZAG_004 | Respiratory System | ERR10441579 |
| HRV | 2020 | DTU_2020_TWIW_01_HRV_ZAG_004 | Respiratory System | ERR14129315 |
| HRV | 2020 | DTU_2020_TWIW_01_HRV_ZAG_005 | Urine | ERR10441571 |
| HRV | 2020 | DTU_2020_TWIW_01_HRV_ZAG_006 | Urine | ERR10441535 |
| HRV | 2020 | DTU_2020_TWIW_01_HRV_ZAG_006 | Urine | ERR14129317 |
| HRV | 2020 | DTU_2020_TWIW_01_HRV_ZAG_007 | Urine | ERR10441554 |
| HRV | 2020 | DTU_2020_TWIW_01_HRV_ZAG_008 | Urine | ERR10441551 |
| HRV | 2020 | DTU_2020_TWIW_01_HRV_ZAG_009 | Wound Pus Biopsy | ERR10441582 |
| HRV | 2020 | DTU_2020_TWIW_01_HRV_ZAG_010 | Wound Pus Biopsy | ERR10441536 |
| HRV | 2020 | DTU_2020_TWIW_01_HRV_ZAG_010 | Wound Pus Biopsy | ERR14129318 |
| HRV | 2020 | DTU_2020_TWIW_01_HRV_ZAG_011 | Blood | ERR10441559 |
| HRV | 2020 | DTU_2020_TWIW_01_HRV_ZAG_011 | Blood | ERR14129319 |
| HRV | 2020 | DTU_2020_TWIW_01_HRV_ZAG_012 | Blood | ERR10441574 |
| HRV | 2020 | DTU_2020_TWIW_01_HRV_ZAG_013 | Blood | ERR14129320 |
| HRV | 2020 | DTU_2020_TWIW_01_HRV_ZAG_013 | Blood | ERR10441558 |
| HRV | 2020 | DTU_2020_TWIW_01_HRV_ZAG_014 | Respiratory System | ERR10441584 |
| HRV | 2020 | DTU_2020_TWIW_01_HRV_ZAG_015 | Respiratory System | ERR10441578 |
| HRV | 2020 | DTU_2020_TWIW_01_HRV_ZAG_016 | Respiratory System | ERR10441520 |
| HRV | 2020 | DTU_2020_TWIW_01_HRV_ZAG_018 | Urine | ERR10441550 |
| HRV | 2020 | DTU_2020_TWIW_01_HRV_ZAG_019 | Urine | ERR10441563 |
| HRV | 2020 | DTU_2020_TWIW_01_HRV_ZAG_020 | Urine | ERR10441556 |
| HRV | 2020 | DTU_2020_TWIW_01_HRV_ZAG_021 | Wound Pus Biopsy | ERR10441547 |
| HRV | 2020 | DTU_2020_TWIW_01_HRV_ZAG_022 | Wound Pus Biopsy | ERR10441522 |
| HRV | 2020 | DTU_2020_TWIW_01_HRV_ZAG_023 | Blood | ERR10441583 |
| HRV | 2020 | DTU_2020_TWIW_01_HRV_ZAG_024 | Blood | ERR10441523 |
| HRV | 2020 | DTU_2020_TWIW_01_HRV_ZAG_025 | Respiratory System | ERR10441567 |
| HRV | 2020 | DTU_2020_TWIW_01_HRV_ZAG_026 | Respiratory System | ERR10441533 |
| HRV | 2020 | DTU_2020_TWIW_01_HRV_ZAG_027 | Respiratory System | ERR10441552 |
| HRV | 2020 | DTU_2020_TWIW_01_HRV_ZAG_028 | Respiratory System | ERR10441540 |
| HRV | 2020 | DTU_2020_TWIW_01_HRV_ZAG_029 | Urine | ERR10441562 |
| HRV | 2020 | DTU_2020_TWIW_01_HRV_ZAG_031 | Urine | ERR10441560 |
| HRV | 2020 | DTU_2020_TWIW_01_HRV_ZAG_032 | Urine | ERR10441585 |
| HRV | 2020 | DTU_2020_TWIW_01_HRV_ZAG_033 | Wound Pus Biopsy | ERR10441542 |
| HRV | 2020 | DTU_2020_TWIW_01_HRV_ZAG_034 | Wound Pus Biopsy | ERR10441553 |
| HRV | 2020 | DTU_2020_TWIW_01_HRV_ZAG_035 | Blood | ERR10441566 |
| HRV | 2020 | DTU_2020_TWIW_01_HRV_ZAG_036 | Blood | ERR10441546 |
| HRV | 2020 | DTU_2020_TWIW_01_HRV_ZAG_037 | Respiratory System | ERR10441544 |
| HRV | 2020 | DTU_2020_TWIW_01_HRV_ZAG_038 | Respiratory System | ERR10441555 |
| HRV | 2020 | DTU_2020_TWIW_01_HRV_ZAG_039 | Respiratory System | ERR10441564 |
| HRV | 2020 | DTU_2020_TWIW_01_HRV_ZAG_040 | Respiratory System | ERR10441595 |
| HRV | 2020 | DTU_2020_TWIW_01_HRV_ZAG_041 | Urine | ERR10441565 |
| HRV | 2020 | DTU_2020_TWIW_01_HRV_ZAG_045 | Wound Pus Biopsy | ERR10441576 |
| HRV | 2020 | DTU_2020_TWIW_01_HRV_ZAG_046 | Wound Pus Biopsy | ERR10441541 |
| HRV | 2020 | DTU_2020_TWIW_01_HRV_ZAG_047 | Blood | ERR10441580 |
| HRV | 2020 | DTU_2020_TWIW_01_HRV_ZAG_048 | Blood | ERR10441573 |
| HRV | 2020 | DTU_2020_TWIW_01_HRV_ZAG_052 | Respiratory System | ERR10441577 |
| HRV | 2020 | DTU_2020_TWIW_01_HRV_ZAG_053 | Urine | ERR10441568 |
| HRV | 2020 | DTU_2020_TWIW_01_HRV_ZAG_054 | Urine | ERR10441593 |
| HRV | 2020 | DTU_2020_TWIW_01_HRV_ZAG_055 | Urine | ERR10441534 |
| HRV | 2020 | DTU_2020_TWIW_01_HRV_ZAG_056 | Urine | ERR10441572 |
| HRV | 2020 | DTU_2020_TWIW_01_HRV_ZAG_057 | Wound Pus Biopsy | ERR10441569 |
| HRV | 2020 | DTU_2020_TWIW_01_HRV_ZAG_058 | Wound Pus Biopsy | ERR10441575 |
| HRV | 2020 | DTU_2020_TWIW_01_HRV_ZAG_059 | Blood | ERR10441570 |
| HRV | 2020 | DTU_2020_TWIW_01_HRV_ZAG_060 | Blood | ERR10441581 |
| FRA | 2020 | DTU_2020_TWIW_01_FRA_LIL_001 | Other | ERR10437734 |
| FRA | 2020 | DTU_2020_TWIW_01_FRA_LIL_002 | Urine | ERR10437695 |
| FRA | 2020 | DTU_2020_TWIW_01_FRA_LIL_003 | Other | ERR10437640 |
| FRA | 2020 | DTU_2020_TWIW_01_FRA_LIL_004 | Blood | ERR10437488 |
| FRA | 2020 | DTU_2020_TWIW_01_FRA_LIL_005 | Blood | ERR10437382 |
| FRA | 2020 | DTU_2020_TWIW_01_FRA_LIL_006 | Urine | ERR10437429 |
| FRA | 2020 | DTU_2020_TWIW_01_FRA_LIL_006 | Urine | ERR14129322 |
| FRA | 2020 | DTU_2020_TWIW_01_FRA_LIL_007 | Respiratory System | ERR10437452 |
| FRA | 2020 | DTU_2020_TWIW_01_FRA_LIL_008 | Blood | ERR10437705 |
| FRA | 2020 | DTU_2020_TWIW_01_FRA_LIL_009 | Urine | ERR10437740 |
| FRA | 2020 | DTU_2020_TWIW_01_FRA_LIL_010 | Urine | ERR10437455 |
| FRA | 2020 | DTU_2020_TWIW_01_FRA_LIL_011 | Urine | ERR10437713 |
| FRA | 2020 | DTU_2020_TWIW_01_FRA_LIL_012 | Blood | ERR10437453 |
| FRA | 2020 | DTU_2020_TWIW_01_FRA_LIL_013 | Wound Pus Biopsy | ERR10437651 |
| FRA | 2020 | DTU_2020_TWIW_01_FRA_LIL_014 | Other | ERR10437715 |
| FRA | 2020 | DTU_2020_TWIW_01_FRA_LIL_014 | Other | ERR14129323 |
| FRA | 2020 | DTU_2020_TWIW_01_FRA_LIL_015 | Urine | ERR10437454 |
| FRA | 2020 | DTU_2020_TWIW_01_FRA_LIL_016 | Urine | ERR10437467 |
| FRA | 2020 | DTU_2020_TWIW_01_FRA_LIL_017 | Urine | ERR10437379 |
| FRA | 2020 | DTU_2020_TWIW_01_FRA_LIL_018 | Urine | ERR10437473 |
| FRA | 2020 | DTU_2020_TWIW_01_FRA_LIL_019 | Urine | ERR10437475 |
| FRA | 2020 | DTU_2020_TWIW_01_FRA_LIL_020 | Blood | ERR10437724 |
| FRA | 2020 | DTU_2020_TWIW_01_FRA_LIL_022 | Urine | ERR10437472 |
| FRA | 2020 | DTU_2020_TWIW_01_FRA_LIL_023 | Urine | ERR10437487 |
| FRA | 2020 | DTU_2020_TWIW_01_FRA_LIL_024 | Blood | ERR10437711 |
| FRA | 2020 | DTU_2020_TWIW_01_FRA_LIL_026 | Blood | ERR10437709 |
| FRA | 2020 | DTU_2020_TWIW_01_FRA_LIL_027 | Respiratory System | ERR10437643 |
| FRA | 2020 | DTU_2020_TWIW_01_FRA_LIL_028 | Blood | ERR10437381 |
| FRA | 2020 | DTU_2020_TWIW_01_FRA_LIL_029 | Blood | ERR10437702 |
| FRA | 2020 | DTU_2020_TWIW_01_FRA_LIL_030 | Other | ERR10437716 |
| FRA | 2020 | DTU_2020_TWIW_01_FRA_LIL_031 | Blood | ERR10437725 |
| FRA | 2020 | DTU_2020_TWIW_01_FRA_LIL_032 | Blood | ERR10437568 |
| FRA | 2020 | DTU_2020_TWIW_01_FRA_LIL_034 | Other | ERR10437650 |
| FRA | 2020 | DTU_2020_TWIW_01_FRA_LIL_035 | Blood | ERR10437708 |
| FRA | 2020 | DTU_2020_TWIW_01_FRA_LIL_036 | Other | ERR10437620 |
| FRA | 2020 | DTU_2020_TWIW_01_FRA_LIL_037 | Blood | ERR10437573 |
| FRA | 2020 | DTU_2020_TWIW_01_FRA_LIL_038 | Blood | ERR10437481 |
| FRA | 2020 | DTU_2020_TWIW_01_FRA_LIL_039 | Blood | ERR10437484 |
| FRA | 2020 | DTU_2020_TWIW_01_FRA_LIL_040 | Urine | ERR10437377 |
| FRA | 2020 | DTU_2020_TWIW_01_FRA_LIL_041 | Other | ERR10437664 |
| FRA | 2020 | DTU_2020_TWIW_01_FRA_LIL_042 | Other | ERR10437663 |
| FRA | 2020 | DTU_2020_TWIW_01_FRA_LIL_043 | Respiratory System | ERR10437656 |
| FRA | 2020 | DTU_2020_TWIW_01_FRA_LIL_044 | Respiratory System | ERR10437394 |
| FRA | 2020 | DTU_2020_TWIW_01_FRA_LIL_045 | Respiratory System | ERR10437621 |
| FRA | 2020 | DTU_2020_TWIW_01_FRA_LIL_046 | Respiratory System | ERR10437501 |
| FRA | 2020 | DTU_2020_TWIW_01_FRA_LIL_047 | Blood | ERR10437509 |
| FRA | 2020 | DTU_2020_TWIW_01_FRA_LIL_048 | Blood | ERR10437428 |
| FRA | 2020 | DTU_2020_TWIW_01_FRA_LIL_049 | Blood | ERR10437427 |
| FRA | 2020 | DTU_2020_TWIW_01_FRA_LIL_049 | Blood | ERR14129324 |
| FRA | 2020 | DTU_2020_TWIW_01_FRA_LIL_050 | Urine | ERR10437390 |
| FRA | 2020 | DTU_2020_TWIW_01_FRA_LIL_051 | Urine | ERR10437495 |
| FRA | 2020 | DTU_2020_TWIW_01_FRA_LIL_052 | Urine | ERR10437508 |
| FRA | 2020 | DTU_2020_TWIW_01_FRA_LIL_053 | Urine | ERR10437502 |
| FRA | 2020 | DTU_2020_TWIW_01_FRA_LIL_054 | Urine | ERR10437572 |
| FRA | 2020 | DTU_2020_TWIW_01_FRA_LIL_055 | Urine | ERR10437499 |
| FRA | 2020 | DTU_2020_TWIW_01_FRA_LIL_056 | Urine | ERR10437717 |
| FRA | 2020 | DTU_2020_TWIW_01_FRA_LIL_057 | Urine | ERR10437506 |
| FRA | 2020 | DTU_2020_TWIW_01_FRA_LIL_058 | Urine | ERR10437574 |
| FRA | 2020 | DTU_2020_TWIW_01_FRA_LIL_059 | Respiratory System | ERR10437666 |
| FRA | 2020 | DTU_2020_TWIW_01_FRA_LIL_060 | Respiratory System | ERR10437523 |
| FRA | 2020 | DTU_2020_TWIW_01_FRA_LIL_061 | Respiratory System | ERR10437665 |
| FRA | 2020 | DTU_2020_TWIW_01_FRA_LIL_062 | Respiratory System | ERR10437733 |
| FRA | 2020 | DTU_2020_TWIW_01_FRA_LIL_063 | Respiratory System | ERR10437712 |
| FRA | 2020 | DTU_2020_TWIW_01_FRA_LIL_064 | Urine | ERR10437528 |
| FRA | 2020 | DTU_2020_TWIW_01_FRA_LIL_065 | Urine | ERR10437721 |
| ESP | 2020 | DTU_2020_TWIW_01_ESP_BAR_001 | Respiratory System | ERR10432407 |
| ESP | 2020 | DTU_2020_TWIW_01_ESP_BAR_003 | Urine | ERR10432403 |
| ESP | 2020 | DTU_2020_TWIW_01_ESP_BAR_004 | Wound Pus Biopsy | ERR10432445 |
| ESP | 2020 | DTU_2020_TWIW_01_ESP_BAR_005 | Blood | ERR10432435 |
| ESP | 2020 | DTU_2020_TWIW_01_ESP_BAR_006 | Blood | ERR10432423 |
| ESP | 2020 | DTU_2020_TWIW_01_ESP_BAR_007 | Blood | ERR10432408 |
| ESP | 2020 | DTU_2020_TWIW_01_ESP_BAR_008 | Urine | ERR10432404 |
| ESP | 2020 | DTU_2020_TWIW_01_ESP_BAR_009 | Urine | ERR10432405 |
| ESP | 2020 | DTU_2020_TWIW_01_ESP_BAR_010 | Urine | ERR10432410 |
| ESP | 2020 | DTU_2020_TWIW_01_ESP_BAR_011 | Urine | ERR10432446 |
| ESP | 2020 | DTU_2020_TWIW_01_ESP_BAR_012 | Urine | ERR10432415 |
| ESP | 2020 | DTU_2020_TWIW_01_ESP_BAR_013 | Urine | ERR10432421 |
| ESP | 2020 | DTU_2020_TWIW_01_ESP_BAR_014 | Blood | ERR10432406 |
| ESP | 2020 | DTU_2020_TWIW_01_ESP_BAR_015 | Blood | ERR10432447 |
| ESP | 2020 | DTU_2020_TWIW_01_ESP_BAR_016 | Blood | ERR10432409 |
| ESP | 2020 | DTU_2020_TWIW_01_ESP_BAR_017 | Blood | ERR10432453 |
| ESP | 2020 | DTU_2020_TWIW_01_ESP_BAR_018 | Blood | ERR10432431 |
| ESP | 2020 | DTU_2020_TWIW_01_ESP_BAR_021 | Urine | ERR10432438 |
| ESP | 2020 | DTU_2020_TWIW_01_ESP_BAR_024 | Urine | ERR10432411 |
| ESP | 2020 | DTU_2020_TWIW_01_ESP_BAR_025 | Blood | ERR10432416 |
| ESP | 2020 | DTU_2020_TWIW_01_ESP_BAR_026 | Wound Pus Biopsy | ERR10432440 |
| ESP | 2020 | DTU_2020_TWIW_01_ESP_BAR_027 | Wound Pus Biopsy | ERR10432414 |
| ESP | 2020 | DTU_2020_TWIW_01_ESP_BAR_028 | Urine | ERR10432419 |
| ESP | 2020 | DTU_2020_TWIW_01_ESP_BAR_029 | Urine | ERR10432428 |
| ESP | 2020 | DTU_2020_TWIW_01_ESP_BAR_030 | Urine | ERR10432452 |
| ESP | 2020 | DTU_2020_TWIW_01_ESP_BAR_031 | Respiratory System | ERR10432401 |
| ESP | 2020 | DTU_2020_TWIW_01_ESP_BAR_035 | Urine | ERR10432418 |
| ESP | 2020 | DTU_2020_TWIW_01_ESP_BAR_040 | Wound Pus Biopsy | ERR10432444 |
| ESP | 2020 | DTU_2020_TWIW_01_ESP_BAR_041 | Blood | ERR10432441 |
| ESP | 2020 | DTU_2020_TWIW_01_ESP_BAR_042 | Blood | ERR10432449 |
| ESP | 2020 | DTU_2020_TWIW_01_ESP_BAR_043 | Blood | ERR10432443 |
| ESP | 2020 | DTU_2020_TWIW_01_ESP_BAR_044 | Blood | ERR10432437 |
| ESP | 2020 | DTU_2020_TWIW_01_ESP_BAR_045 | Blood | ERR10432432 |
| ESP | 2020 | DTU_2020_TWIW_01_ESP_BAR_046 | Urine | ERR10432433 |
| ESP | 2020 | DTU_2020_TWIW_01_ESP_BAR_047 | Urine | ERR10432426 |
| ESP | 2020 | DTU_2020_TWIW_01_ESP_BAR_048 | Urine | ERR10432420 |
| ESP | 2020 | DTU_2020_TWIW_01_ESP_BAR_049 | Urine | ERR10432427 |
| ESP | 2020 | DTU_2020_TWIW_01_ESP_BAR_050 | Urine | ERR10432425 |
| ESP | 2020 | DTU_2020_TWIW_01_ESP_BAR_051 | Urine | ERR10432429 |
| ESP | 2020 | DTU_2020_TWIW_01_ESP_BAR_052 | Urine | ERR10432424 |
| ESP | 2020 | DTU_2020_TWIW_01_ESP_BAR_053 | Urine | ERR10432422 |
| ESP | 2020 | DTU_2020_TWIW_01_ESP_BAR_054 | Respiratory System | ERR10432448 |
| ESP | 2020 | DTU_2020_TWIW_01_ESP_BAR_055 | Respiratory System | ERR10432434 |
| ESP | 2020 | DTU_2020_TWIW_01_ESP_BAR_056 | Other | ERR10432439 |
| ESP | 2020 | DTU_2020_TWIW_01_ESP_BAR_057 | Wound Pus Biopsy | ERR10432457 |
| ESP | 2020 | DTU_2020_TWIW_01_ESP_BAR_058 | Other | ERR10432442 |
| ESP | 2020 | DTU_2020_TWIW_01_ESP_BAR_059 | Blood | ERR10432455 |
| ESP | 2020 | DTU_2020_TWIW_01_ESP_BAR_060 | Blood | ERR10432412 |
| ITA | 2020 | DTU_2020_TWIW_01_ITA_MIL_001 | Blood | ERR10438631 |
| ITA | 2020 | DTU_2020_TWIW_01_ITA_MIL_002 | Blood | ERR10438595 |
| ITA | 2020 | DTU_2020_TWIW_01_ITA_MIL_003 | Urine | ERR10438596 |
| ITA | 2020 | DTU_2020_TWIW_01_ITA_MIL_004 | Urine | ERR10438608 |
| ITA | 2020 | DTU_2020_TWIW_01_ITA_MIL_005 | Urine | ERR10438639 |
| ITA | 2020 | DTU_2020_TWIW_01_ITA_MIL_006 | Urine | ERR10438597 |
| ITA | 2020 | DTU_2020_TWIW_01_ITA_MIL_007 | Urine | ERR10438632 |
| ITA | 2020 | DTU_2020_TWIW_01_ITA_MIL_008 | Other | ERR10438616 |
| ITA | 2020 | DTU_2020_TWIW_01_ITA_MIL_009 | Respiratory System | ERR10438579 |
| ITA | 2020 | DTU_2020_TWIW_01_ITA_MIL_010 | Respiratory System | ERR10438644 |
| ITA | 2020 | DTU_2020_TWIW_01_ITA_MIL_011 | Respiratory System | ERR10438648 |
| ITA | 2020 | DTU_2020_TWIW_01_ITA_MIL_012 | Urine | ERR10438619 |
| ITA | 2020 | DTU_2020_TWIW_01_ITA_MIL_013 | Urine | ERR10438609 |
| ITA | 2020 | DTU_2020_TWIW_01_ITA_MIL_014 | Urine | ERR10438633 |
| ITA | 2020 | DTU_2020_TWIW_01_ITA_MIL_015 | Urine | ERR10438634 |
| ITA | 2020 | DTU_2020_TWIW_01_ITA_MIL_016 | Urine | ERR10438647 |
| ITA | 2020 | DTU_2020_TWIW_01_ITA_MIL_017 | Urine | ERR10438635 |
| ITA | 2020 | DTU_2020_TWIW_01_ITA_MIL_018 | Urine | ERR14129325 |
| ITA | 2020 | DTU_2020_TWIW_01_ITA_MIL_018 | Urine | ERR10438599 |
| ITA | 2020 | DTU_2020_TWIW_01_ITA_MIL_019 | Urine | ERR10438629 |
| ITA | 2020 | DTU_2020_TWIW_01_ITA_MIL_020 | Urine | ERR10438640 |
| ITA | 2020 | DTU_2020_TWIW_01_ITA_MIL_021 | Urine | ERR10438600 |
| ITA | 2020 | DTU_2020_TWIW_01_ITA_MIL_022 | Wound Pus Biopsy | ERR10438617 |
| ITA | 2020 | DTU_2020_TWIW_01_ITA_MIL_023 | Swab | ERR10438641 |
| ITA | 2020 | DTU_2020_TWIW_01_ITA_MIL_023 | Swab | ERR14129327 |
| ITA | 2020 | DTU_2020_TWIW_01_ITA_MIL_024 | Swab | ERR10438621 |
| ITA | 2020 | DTU_2020_TWIW_01_ITA_MIL_025 | Swab | ERR10438645 |
| ITA | 2020 | DTU_2020_TWIW_01_ITA_MIL_026 | Swab | ERR10438593 |
| ITA | 2020 | DTU_2020_TWIW_01_ITA_MIL_027 | Swab | ERR10438618 |
| ITA | 2020 | DTU_2020_TWIW_01_ITA_MIL_028 | Swab | ERR10438622 |
| ITA | 2020 | DTU_2020_TWIW_01_ITA_MIL_029 | Blood | ERR10438624 |
| ITA | 2020 | DTU_2020_TWIW_01_ITA_MIL_030 | Urine | ERR10438598 |
| ITA | 2020 | DTU_2020_TWIW_01_ITA_MIL_031 | Urine | ERR10438602 |
| ITA | 2020 | DTU_2020_TWIW_01_ITA_MIL_032 | Urine | ERR10438636 |
| ITA | 2020 | DTU_2020_TWIW_01_ITA_MIL_033 | Urine | ERR10438637 |
| ITA | 2020 | DTU_2020_TWIW_01_ITA_MIL_034 | Urine | ERR10438601 |
| ITA | 2020 | DTU_2020_TWIW_01_ITA_MIL_035 | Urine | ERR10438630 |
| ITA | 2020 | DTU_2020_TWIW_01_ITA_MIL_036 | Urine | ERR10438594 |
| ITA | 2020 | DTU_2020_TWIW_01_ITA_MIL_037 | Urine | ERR10438605 |
| ITA | 2020 | DTU_2020_TWIW_01_ITA_MIL_038 | Urine | ERR10438604 |
| ITA | 2020 | DTU_2020_TWIW_01_ITA_MIL_039 | Urine | ERR10438606 |
| ITA | 2020 | DTU_2020_TWIW_01_ITA_MIL_040 | Urine | ERR10438607 |
| ITA | 2020 | DTU_2020_TWIW_01_ITA_MIL_041 | Urine | ERR10438620 |
| ITA | 2020 | DTU_2020_TWIW_01_ITA_MIL_042 | Urine | ERR10438611 |
| ITA | 2020 | DTU_2020_TWIW_01_ITA_MIL_043 | Urine | ERR10438615 |
| ITA | 2020 | DTU_2020_TWIW_01_ITA_MIL_044 | Wound Pus Biopsy | ERR10438625 |
| ITA | 2020 | DTU_2020_TWIW_01_ITA_MIL_045 | Swab | ERR10438588 |
| ITA | 2020 | DTU_2020_TWIW_01_ITA_MIL_046 | Blood | ERR10438612 |
| ITA | 2020 | DTU_2020_TWIW_01_ITA_MIL_047 | Respiratory System | ERR10438623 |
| ITA | 2020 | DTU_2020_TWIW_01_ITA_MIL_048 | Respiratory System | ERR10438643 |
| ITA | 2020 | DTU_2020_TWIW_01_ITA_MIL_049 | Respiratory System | ERR10438642 |
| ITA | 2020 | DTU_2020_TWIW_01_ITA_MIL_050 | Swab | ERR10438613 |
| ITA | 2020 | DTU_2020_TWIW_01_ITA_MIL_051 | Swab | ERR10438626 |
| ITA | 2020 | DTU_2020_TWIW_01_ITA_MIL_052 | Swab | ERR10438627 |
| ITA | 2020 | DTU_2020_TWIW_01_ITA_MIL_053 | Respiratory System | ERR10438589 |
| ITA | 2020 | DTU_2020_TWIW_01_ITA_MIL_054 | Blood | ERR10438610 |
| ITA | 2020 | DTU_2020_TWIW_01_ITA_MIL_055 | Respiratory System | ERR10441715 |
| ITA | 2020 | DTU_2020_TWIW_01_ITA_MIL_056 | Wound Pus Biopsy | ERR10438603 |
| ITA | 2020 | DTU_2020_TWIW_01_ITA_MIL_057 | Wound Pus Biopsy | ERR10438646 |
| ITA | 2020 | DTU_2020_TWIW_01_ITA_MIL_058 | Blood | ERR10438614 |
| ITA | 2020 | DTU_2020_TWIW_01_ITA_MIL_060 | Blood | ERR10438628 |
| LTU | 2020 | DTU_2020_TWIW_01_LTU_KAU_001 | Respiratory System | ERR10438697 |
| LTU | 2020 | DTU_2020_TWIW_01_LTU_KAU_002 | Respiratory System | ERR10438679 |
| LTU | 2020 | DTU_2020_TWIW_01_LTU_KAU_004 | Respiratory System | ERR10438685 |
| LTU | 2020 | DTU_2020_TWIW_01_LTU_KAU_005 | Respiratory System | ERR10438678 |
| LTU | 2020 | DTU_2020_TWIW_01_LTU_KAU_006 | Respiratory System | ERR10438704 |
| LTU | 2020 | DTU_2020_TWIW_01_LTU_KAU_007 | Other | ERR10438665 |
| LTU | 2020 | DTU_2020_TWIW_01_LTU_KAU_008 | Blood | ERR10438681 |
| LTU | 2020 | DTU_2020_TWIW_01_LTU_KAU_009 | Blood | ERR10438687 |
| LTU | 2020 | DTU_2020_TWIW_01_LTU_KAU_010 | Wound Pus Biopsy | ERR10438650 |
| LTU | 2020 | DTU_2020_TWIW_01_LTU_KAU_011 | Other | ERR10438688 |
| LTU | 2020 | DTU_2020_TWIW_01_LTU_KAU_012 | Urine | ERR10438675 |
| LTU | 2020 | DTU_2020_TWIW_01_LTU_KAU_013 | Urine | ERR10438708 |
| LTU | 2020 | DTU_2020_TWIW_01_LTU_KAU_014 | Urine | ERR10438664 |
| LTU | 2020 | DTU_2020_TWIW_01_LTU_KAU_015 | Wound Pus Biopsy | ERR10438699 |
| LTU | 2020 | DTU_2020_TWIW_01_LTU_KAU_016 | Blood | ERR10438695 |
| LTU | 2020 | DTU_2020_TWIW_01_LTU_KAU_017 | Other | ERR10438698 |
| LTU | 2020 | DTU_2020_TWIW_01_LTU_KAU_018 | Respiratory System | ERR10438649 |
| LTU | 2020 | DTU_2020_TWIW_01_LTU_KAU_019 | Respiratory System | ERR10438660 |
| LTU | 2020 | DTU_2020_TWIW_01_LTU_KAU_020 | Respiratory System | ERR10438667 |
| LTU | 2020 | DTU_2020_TWIW_01_LTU_KAU_021 | Urine | ERR10438677 |
| LTU | 2020 | DTU_2020_TWIW_01_LTU_KAU_022 | Urine | ERR10438666 |
| LTU | 2020 | DTU_2020_TWIW_01_LTU_KAU_023 | Swab | ERR10438689 |
| LTU | 2020 | DTU_2020_TWIW_01_LTU_KAU_024 | Blood | ERR10438661 |
| LTU | 2020 | DTU_2020_TWIW_01_LTU_KAU_025 | Urine | ERR10438663 |
| LTU | 2020 | DTU_2020_TWIW_01_LTU_KAU_026 | Other | ERR10438690 |
| LTU | 2020 | DTU_2020_TWIW_01_LTU_KAU_027 | Wound Pus Biopsy | ERR10438686 |
| LTU | 2020 | DTU_2020_TWIW_01_LTU_KAU_028 | Respiratory System | ERR10438676 |
| LTU | 2020 | DTU_2020_TWIW_01_LTU_KAU_029 | Respiratory System | ERR10438684 |
| LTU | 2020 | DTU_2020_TWIW_01_LTU_KAU_030 | Respiratory System | ERR10438696 |
| LTU | 2020 | DTU_2020_TWIW_01_LTU_KAU_031 | Urine | ERR10438668 |
| LTU | 2020 | DTU_2020_TWIW_01_LTU_KAU_032 | Other | ERR10438656 |
| LTU | 2020 | DTU_2020_TWIW_01_LTU_KAU_033 | Blood | ERR10438670 |
| LTU | 2020 | DTU_2020_TWIW_01_LTU_KAU_034 | Blood | ERR10438691 |
| LTU | 2020 | DTU_2020_TWIW_01_LTU_KAU_035 | Urine | ERR10438669 |
| LTU | 2020 | DTU_2020_TWIW_01_LTU_KAU_036 | Respiratory System | ERR10438652 |
| LTU | 2020 | DTU_2020_TWIW_01_LTU_KAU_037 | Respiratory System | ERR10438659 |
| LTU | 2020 | DTU_2020_TWIW_01_LTU_KAU_038 | Respiratory System | ERR10438657 |
| LTU | 2020 | DTU_2020_TWIW_01_LTU_KAU_039 | Wound Pus Biopsy | ERR10438694 |
| LTU | 2020 | DTU_2020_TWIW_01_LTU_KAU_040 | Urine | ERR10438651 |
| LTU | 2020 | DTU_2020_TWIW_01_LTU_KAU_041 | Blood | ERR10438658 |
| LTU | 2020 | DTU_2020_TWIW_01_LTU_KAU_042 | Blood | ERR10438692 |
| LTU | 2020 | DTU_2020_TWIW_01_LTU_KAU_043 | Urine | ERR10438672 |
| LTU | 2020 | DTU_2020_TWIW_01_LTU_KAU_044 | Urine | ERR10438654 |
| LTU | 2020 | DTU_2020_TWIW_01_LTU_KAU_045 | Urine | ERR10438680 |
| LTU | 2020 | DTU_2020_TWIW_01_LTU_KAU_046 | Urine | ERR10438701 |
| LTU | 2020 | DTU_2020_TWIW_01_LTU_KAU_047 | Urine | ERR10438653 |
| LTU | 2020 | DTU_2020_TWIW_01_LTU_KAU_048 | Respiratory System | ERR10438705 |
| LTU | 2020 | DTU_2020_TWIW_01_LTU_KAU_049 | Urine | ERR10438700 |
| LTU | 2020 | DTU_2020_TWIW_01_LTU_KAU_050 | Urine | ERR10438671 |
| LTU | 2020 | DTU_2020_TWIW_01_LTU_KAU_051 | Respiratory System | ERR10438682 |
| LTU | 2020 | DTU_2020_TWIW_01_LTU_KAU_052 | Wound Pus Biopsy | ERR10438702 |
| LTU | 2020 | DTU_2020_TWIW_01_LTU_KAU_053 | Blood | ERR10438673 |
| LTU | 2020 | DTU_2020_TWIW_01_LTU_KAU_054 | Blood | ERR10438693 |
| LTU | 2020 | DTU_2020_TWIW_01_LTU_KAU_055 | Blood | ERR10438662 |
| LTU | 2020 | DTU_2020_TWIW_01_LTU_KAU_056 | Respiratory System | ERR10438655 |
| LTU | 2020 | DTU_2020_TWIW_01_LTU_KAU_057 | Blood | ERR10438674 |
| LTU | 2020 | DTU_2020_TWIW_01_LTU_KAU_059 | Respiratory System | ERR10438703 |
| LTU | 2020 | DTU_2020_TWIW_01_LTU_KAU_060 | Respiratory System | ERR10438683 |
| DEU | 2020 | DTU_2020_TWIW_01_DEU_MAG_001 | Urine | ERR10437746 |
| DEU | 2020 | DTU_2020_TWIW_01_DEU_MAG_002 | Urine | ERR10437827 |
| DEU | 2020 | DTU_2020_TWIW_01_DEU_MAG_003 | Respiratory System | ERR10437750 |
| DEU | 2020 | DTU_2020_TWIW_01_DEU_MAG_004 | Swab | ERR10437789 |
| DEU | 2020 | DTU_2020_TWIW_01_DEU_MAG_005 | Other | ERR10437841 |
| DEU | 2020 | DTU_2020_TWIW_01_DEU_MAG_006 | Blood | ERR10437834 |
| DEU | 2020 | DTU_2020_TWIW_01_DEU_MAG_007 | Urine | ERR10437882 |
| DEU | 2020 | DTU_2020_TWIW_01_DEU_MAG_008 | Urine | ERR10437758 |
| DEU | 2020 | DTU_2020_TWIW_01_DEU_MAG_008 | Urine | ERR14129328 |
| DEU | 2020 | DTU_2020_TWIW_01_DEU_MAG_009 | Wound Pus Biopsy | ERR10437854 |
| DEU | 2020 | DTU_2020_TWIW_01_DEU_MAG_010 | Wound Pus Biopsy | ERR10437795 |
| DEU | 2020 | DTU_2020_TWIW_01_DEU_MAG_011 | Wound Pus Biopsy | ERR10437779 |
| DEU | 2020 | DTU_2020_TWIW_01_DEU_MAG_012 | Swab | ERR10437810 |
| DEU | 2020 | DTU_2020_TWIW_01_DEU_MAG_013 | Respiratory System | ERR10437760 |
| DEU | 2020 | DTU_2020_TWIW_01_DEU_MAG_014 | Urine | ERR10437757 |
| DEU | 2020 | DTU_2020_TWIW_01_DEU_MAG_015 | Urine | ERR10437825 |
| DEU | 2020 | DTU_2020_TWIW_01_DEU_MAG_016 | Urine | ERR10437883 |
| DEU | 2020 | DTU_2020_TWIW_01_DEU_MAG_017 | Urine | ERR10437744 |
| DEU | 2020 | DTU_2020_TWIW_01_DEU_MAG_018 | Wound Pus Biopsy | ERR10437763 |
| DEU | 2020 | DTU_2020_TWIW_01_DEU_MAG_019 | Wound Pus Biopsy | ERR10437818 |
| DEU | 2020 | DTU_2020_TWIW_01_DEU_MAG_020 | Urine | ERR10437855 |
| DEU | 2020 | DTU_2020_TWIW_01_DEU_MAG_021 | Urine | ERR10437766 |
| DEU | 2020 | DTU_2020_TWIW_01_DEU_MAG_022 | Swab | ERR10437824 |
| DEU | 2020 | DTU_2020_TWIW_01_DEU_MAG_023 | Swab | ERR10437842 |
| DEU | 2020 | DTU_2020_TWIW_01_DEU_MAG_024 | Swab | ERR10437820 |
| DEU | 2020 | DTU_2020_TWIW_01_DEU_MAG_025 | Blood | ERR10437853 |
| DEU | 2020 | DTU_2020_TWIW_01_DEU_MAG_026 | Wound Pus Biopsy | ERR10437815 |
| DEU | 2020 | DTU_2020_TWIW_01_DEU_MAG_027 | Other | ERR10437743 |
| DEU | 2020 | DTU_2020_TWIW_01_DEU_MAG_028 | Urine | ERR10437768 |
| DEU | 2020 | DTU_2020_TWIW_01_DEU_MAG_029 | Urine | ERR10437823 |
| DEU | 2020 | DTU_2020_TWIW_01_DEU_MAG_030 | Urine | ERR10437747 |
| DEU | 2020 | DTU_2020_TWIW_01_DEU_MAG_031 | Wound Pus Biopsy | ERR10437786 |
| DEU | 2020 | DTU_2020_TWIW_01_DEU_MAG_032 | Other | ERR10437868 |
| DEU | 2020 | DTU_2020_TWIW_01_DEU_MAG_033 | Respiratory System | ERR10437857 |
| DEU | 2020 | DTU_2020_TWIW_01_DEU_MAG_034 | Wound Pus Biopsy | ERR10437801 |
| DEU | 2020 | DTU_2020_TWIW_01_DEU_MAG_035 | Urine | ERR10437749 |
| DEU | 2020 | DTU_2020_TWIW_01_DEU_MAG_036 | Urine | ERR10437765 |
| DEU | 2020 | DTU_2020_TWIW_01_DEU_MAG_037 | Respiratory System | ERR10437762 |
| DEU | 2020 | DTU_2020_TWIW_01_DEU_MAG_039 | Other | ERR10437819 |
| DEU | 2020 | DTU_2020_TWIW_01_DEU_MAG_040 | Other | ERR10437759 |
| DEU | 2020 | DTU_2020_TWIW_01_DEU_MAG_041 | Urine | ERR10437876 |
| DEU | 2020 | DTU_2020_TWIW_01_DEU_MAG_041 | Urine | ERR14129331 |
| DEU | 2020 | DTU_2020_TWIW_01_DEU_MAG_042 | Other | ERR10437826 |
| DEU | 2020 | DTU_2020_TWIW_01_DEU_MAG_043 | Other | ERR10437751 |
| DEU | 2020 | DTU_2020_TWIW_01_DEU_MAG_044 | Other | ERR10437748 |
| DEU | 2020 | DTU_2020_TWIW_01_DEU_MAG_045 | Other | ERR10437821 |
| DEU | 2020 | DTU_2020_TWIW_01_DEU_MAG_046 | Blood | ERR10437877 |
| DEU | 2020 | DTU_2020_TWIW_01_DEU_MAG_047 | Urine | ERR10437785 |
| DEU | 2020 | DTU_2020_TWIW_01_DEU_MAG_048 | Urine | ERR10437839 |
| DEU | 2020 | DTU_2020_TWIW_01_DEU_MAG_049 | Swab | ERR10437822 |
| DEU | 2020 | DTU_2020_TWIW_01_DEU_MAG_050 | Wound Pus Biopsy | ERR10437767 |
| DEU | 2020 | DTU_2020_TWIW_01_DEU_MAG_051 | Respiratory System | ERR10437856 |
| DEU | 2020 | DTU_2020_TWIW_01_DEU_MAG_052 | Swab | ERR10437761 |
| DEU | 2020 | DTU_2020_TWIW_01_DEU_MAG_053 | Blood | ERR10437803 |
| DEU | 2020 | DTU_2020_TWIW_01_DEU_MAG_054 | Urine | ERR10437764 |
| DEU | 2020 | DTU_2020_TWIW_01_DEU_MAG_055 | Urine | ERR10437778 |
| DEU | 2020 | DTU_2020_TWIW_01_DEU_MAG_056 | Wound Pus Biopsy | ERR10437793 |
| DEU | 2020 | DTU_2020_TWIW_01_DEU_MAG_057 | Blood | ERR10437869 |
| DEU | 2020 | DTU_2020_TWIW_01_DEU_MAG_058 | Urine | ERR10437771 |
| DEU | 2020 | DTU_2020_TWIW_01_DEU_MAG_059 | Urine | ERR10437755 |
| DEU | 2020 | DTU_2020_TWIW_01_DEU_MAG_060 | Wound Pus Biopsy | ERR10437745 |
| DEU | 2020 | DTU_2020_TWIW_01_DEU_MAG_061 | Wound Pus Biopsy | ERR10437769 |
| DEU | 2020 | DTU_2020_TWIW_01_DEU_MAG_062 | Swab | ERR10437753 |
| DEU | 2020 | DTU_2020_TWIW_01_DEU_MAG_063 | Wound Pus Biopsy | ERR10437817 |
| DEU | 2020 | DTU_2020_TWIW_01_DEU_MAG_064 | Other | ERR10437802 |
| DEU | 2020 | DTU_2020_TWIW_01_DEU_MAG_065 | Urine | ERR10437770 |
| DEU | 2020 | DTU_2020_TWIW_01_DEU_MAG_066 | Urine | ERR10437752 |
| DEU | 2020 | DTU_2020_TWIW_01_DEU_MAG_067 | Swab | ERR10437754 |
| DEU | 2020 | DTU_2020_TWIW_01_DEU_MAG_068 | Wound Pus Biopsy | ERR10437840 |
| DEU | 2020 | DTU_2020_TWIW_01_DEU_MAG_069 | Wound Pus Biopsy | ERR10437816 |
| DEU | 2020 | DTU_2020_TWIW_01_DEU_MAG_070 | Blood | ERR10437756 |
| GHA | 2020 | DTU_2020_TWIW_01_GHA_SEK_001 | Urine | ERR10438043 |
| GHA | 2020 | DTU_2020_TWIW_01_GHA_SEK_002 | Urine | ERR10438026 |
| GHA | 2020 | DTU_2020_TWIW_01_GHA_SEK_002A | Urine | ERR10437904 |
| GHA | 2020 | DTU_2020_TWIW_01_GHA_SEK_003 | Swab | ERR10438036 |
| GHA | 2020 | DTU_2020_TWIW_01_GHA_SEK_004 | Respiratory System | ERR10438023 |
| GHA | 2020 | DTU_2020_TWIW_01_GHA_SEK_005 | Wound Pus Biopsy | ERR10438020 |
| GHA | 2020 | DTU_2020_TWIW_01_GHA_SEK_005A | Wound Pus Biopsy | ERR10437903 |
| GHA | 2020 | DTU_2020_TWIW_01_GHA_SEK_006 | Urine | ERR10438024 |
| GHA | 2020 | DTU_2020_TWIW_01_GHA_SEK_007 | Respiratory System | ERR10438022 |
| GHA | 2020 | DTU_2020_TWIW_01_GHA_SEK_008 | Respiratory System | ERR10437918 |
| GHA | 2020 | DTU_2020_TWIW_01_GHA_SEK_009 | Urine | ERR10438019 |
| GHA | 2020 | DTU_2020_TWIW_01_GHA_SEK_010 | Other | ERR10438042 |
| GHA | 2020 | DTU_2020_TWIW_01_GHA_SEK_011 | Respiratory System | ERR10438005 |
| GHA | 2020 | DTU_2020_TWIW_01_GHA_SEK_012 | Urine | ERR10437938 |
| GHA | 2020 | DTU_2020_TWIW_01_GHA_SEK_013 | Respiratory System | ERR10438018 |
| GHA | 2020 | DTU_2020_TWIW_01_GHA_SEK_013 | Respiratory System | ERR14129333 |
| GHA | 2020 | DTU_2020_TWIW_01_GHA_SEK_014 | Respiratory System | ERR10437939 |
| GHA | 2020 | DTU_2020_TWIW_01_GHA_SEK_015 | Urine | ERR10438027 |
| GHA | 2020 | DTU_2020_TWIW_01_GHA_SEK_016 | Urine | ERR10437932 |
| GHA | 2020 | DTU_2020_TWIW_01_GHA_SEK_018 | Swab | ERR10438039 |
| GHA | 2020 | DTU_2020_TWIW_01_GHA_SEK_019 | Respiratory System | ERR10437891 |
| GHA | 2020 | DTU_2020_TWIW_01_GHA_SEK_020 | Respiratory System | ERR10438028 |
| GHA | 2020 | DTU_2020_TWIW_01_GHA_SEK_021 | Swab | ERR10437946 |
| GHA | 2020 | DTU_2020_TWIW_01_GHA_SEK_022 | Urine | ERR10437964 |
| GHA | 2020 | DTU_2020_TWIW_01_GHA_SEK_023 | Swab | ERR10437978 |
| GHA | 2020 | DTU_2020_TWIW_01_GHA_SEK_024 | Urine | ERR10437958 |
| GHA | 2020 | DTU_2020_TWIW_01_GHA_SEK_025 | Urine | ERR10437965 |
| GHA | 2020 | DTU_2020_TWIW_01_GHA_SEK_026 | Wound Pus Biopsy | ERR10437923 |
| GHA | 2020 | DTU_2020_TWIW_01_GHA_SEK_027 | Urine | ERR10438031 |
| GHA | 2020 | DTU_2020_TWIW_01_GHA_SEK_027 | Urine | ERR14129334 |
| GHA | 2020 | DTU_2020_TWIW_01_GHA_SEK_028 | Urine | ERR10437947 |
| GHA | 2020 | DTU_2020_TWIW_01_GHA_SEK_029 | Urine | ERR10437963 |
| GHA | 2020 | DTU_2020_TWIW_01_GHA_SEK_030 | Urine | ERR10438017 |
| GHA | 2020 | DTU_2020_TWIW_01_GHA_SEK_031 | Urine | ERR10437973 |
| GHA | 2020 | DTU_2020_TWIW_01_GHA_SEK_032 | Urine | ERR10437911 |
| GHA | 2020 | DTU_2020_TWIW_01_GHA_SEK_032A | Urine | ERR10438047 |
| GHA | 2020 | DTU_2020_TWIW_01_GHA_SEK_033 | Swab | ERR10437933 |
| GHA | 2020 | DTU_2020_TWIW_01_GHA_SEK_034 | Urine | ERR10438025 |
| GHA | 2020 | DTU_2020_TWIW_01_GHA_SEK_035 | Urine | ERR10437970 |
| GHA | 2020 | DTU_2020_TWIW_01_GHA_SEK_036 | Swab | ERR10437991 |
| GHA | 2020 | DTU_2020_TWIW_01_GHA_SEK_037 | Wound Pus Biopsy | ERR10437892 |
| GHA | 2020 | DTU_2020_TWIW_01_GHA_SEK_038 | Blood | ERR10438045 |
| GHA | 2020 | DTU_2020_TWIW_01_GHA_SEK_038 | Blood | ERR12041378 |
| GHA | 2020 | DTU_2020_TWIW_01_GHA_SEK_039 | Wound Pus Biopsy | ERR10438041 |
| GHA | 2020 | DTU_2020_TWIW_01_GHA_SEK_040 | Blood | ERR10438035 |
| GHA | 2020 | DTU_2020_TWIW_01_GHA_SEK_041A | Urine | ERR10437990 |
| GHA | 2020 | DTU_2020_TWIW_01_GHA_SEK_042 | Swab | ERR10438034 |
| GHA | 2020 | DTU_2020_TWIW_01_GHA_SEK_043 | Wound Pus Biopsy | ERR10437884 |
| GHA | 2020 | DTU_2020_TWIW_01_GHA_SEK_044 | Respiratory System | ERR10438029 |
| GHA | 2020 | DTU_2020_TWIW_01_GHA_SEK_045 | Swab | ERR10438030 |
| GHA | 2020 | DTU_2020_TWIW_01_GHA_SEK_046 | Urine | ERR10437992 |
| GHA | 2020 | DTU_2020_TWIW_01_GHA_SEK_047 | Wound Pus Biopsy | ERR10438044 |
| GHA | 2020 | DTU_2020_TWIW_01_GHA_SEK_048 | Urine | ERR10437925 |
| GHA | 2020 | DTU_2020_TWIW_01_GHA_SEK_049 | Wound Pus Biopsy | ERR10437985 |
| GHA | 2020 | DTU_2020_TWIW_01_GHA_SEK_050 | Other | ERR10438038 |
| GHA | 2020 | DTU_2020_TWIW_01_GHA_SEK_051 | Swab | ERR10438033 |
| GHA | 2020 | DTU_2020_TWIW_01_GHA_SEK_052 | Wound Pus Biopsy | ERR10438006 |
| GHA | 2020 | DTU_2020_TWIW_01_GHA_SEK_053 | Wound Pus Biopsy | ERR10437910 |
| GHA | 2020 | DTU_2020_TWIW_01_GHA_SEK_054 | Respiratory System | ERR10438048 |
| GHA | 2020 | DTU_2020_TWIW_01_GHA_SEK_054A | Respiratory System | ERR10437999 |
| GHA | 2020 | DTU_2020_TWIW_01_GHA_SEK_055 | Wound Pus Biopsy | ERR10438037 |
| GHA | 2020 | DTU_2020_TWIW_01_GHA_SEK_056 | Swab | ERR10437924 |
| GHA | 2020 | DTU_2020_TWIW_01_GHA_SEK_057 | Wound Pus Biopsy | ERR10438004 |
| GHA | 2020 | DTU_2020_TWIW_01_GHA_SEK_058 | Respiratory System | ERR10438032 |
| GHA | 2020 | DTU_2020_TWIW_01_GHA_SEK_059 | Wound Pus Biopsy | ERR10438040 |
| GHA | 2020 | DTU_2020_TWIW_01_GHA_SEK_060 | Wound Pus Biopsy | ERR10437909 |
| CZE | 2020 | DTU_2020_TWIW_01_CZE_PLZ_001 | Wound Pus Biopsy | ERR10441682 |
| CZE | 2020 | DTU_2020_TWIW_01_CZE_PLZ_002 | Other | ERR10436758 |
| CZE | 2020 | DTU_2020_TWIW_01_CZE_PLZ_003 | Swab | ERR10436734 |
| CZE | 2020 | DTU_2020_TWIW_01_CZE_PLZ_004 | Urine | ERR10436522 |
| CZE | 2020 | DTU_2020_TWIW_01_CZE_PLZ_005 | Urine | ERR10436808 |
| CZE | 2020 | DTU_2020_TWIW_01_CZE_PLZ_006 | Urine | ERR10441684 |
| CZE | 2020 | DTU_2020_TWIW_01_CZE_PLZ_007 | Respiratory System | ERR10441673 |
| CZE | 2020 | DTU_2020_TWIW_01_CZE_PLZ_008 | Urine | ERR10441702 |
| CZE | 2020 | DTU_2020_TWIW_01_CZE_PLZ_009 | Urine | ERR10441704 |
| CZE | 2020 | DTU_2020_TWIW_01_CZE_PLZ_010 | Urine | ERR10441703 |
| CZE | 2020 | DTU_2020_TWIW_01_CZE_PLZ_011 | Urine | ERR10441706 |
| CZE | 2020 | DTU_2020_TWIW_01_CZE_PLZ_012 | Urine | ERR10436578 |
| CZE | 2020 | DTU_2020_TWIW_01_CZE_PLZ_013 | Respiratory System | ERR10436656 |
| CZE | 2020 | DTU_2020_TWIW_01_CZE_PLZ_014 | Urine | ERR10441672 |
| CZE | 2020 | DTU_2020_TWIW_01_CZE_PLZ_015 | Other | ERR10441698 |
| CZE | 2020 | DTU_2020_TWIW_01_CZE_PLZ_016 | Blood | ERR10436630 |
| CZE | 2020 | DTU_2020_TWIW_01_CZE_PLZ_017 | Urine | ERR10436515 |
| CZE | 2020 | DTU_2020_TWIW_01_CZE_PLZ_018 | Urine | ERR10441705 |
| CZE | 2020 | DTU_2020_TWIW_01_CZE_PLZ_019 | Blood | ERR10436753 |
| CZE | 2020 | DTU_2020_TWIW_01_CZE_PLZ_020 | Blood | ERR10436623 |
| CZE | 2020 | DTU_2020_TWIW_01_CZE_PLZ_021 | Blood | ERR10441671 |
| CZE | 2020 | DTU_2020_TWIW_01_CZE_PLZ_022 | Respiratory System | ERR10436786 |
| CZE | 2020 | DTU_2020_TWIW_01_CZE_PLZ_024 | Urine | ERR10441675 |
| CZE | 2020 | DTU_2020_TWIW_01_CZE_PLZ_025 | Urine | ERR10441709 |
| CZE | 2020 | DTU_2020_TWIW_01_CZE_PLZ_026 | Urine | ERR10441712 |
| CZE | 2020 | DTU_2020_TWIW_01_CZE_PLZ_027 | Urine | ERR10441714 |
| CZE | 2020 | DTU_2020_TWIW_01_CZE_PLZ_028 | Urine | ERR10436828 |
| CZE | 2020 | DTU_2020_TWIW_01_CZE_PLZ_029 | Urine | ERR10441710 |
| CZE | 2020 | DTU_2020_TWIW_01_CZE_PLZ_030 | Urine | ERR10441708 |
| CZE | 2020 | DTU_2020_TWIW_01_CZE_PLZ_031 | Urine | ERR10436757 |
| CZE | 2020 | DTU_2020_TWIW_01_CZE_PLZ_032 | Wound Pus Biopsy | ERR10436521 |
| CZE | 2020 | DTU_2020_TWIW_01_CZE_PLZ_033 | Swab | ERR10436635 |
| CZE | 2020 | DTU_2020_TWIW_01_CZE_PLZ_034 | Urine | ERR10441693 |
| CZE | 2020 | DTU_2020_TWIW_01_CZE_PLZ_035 | Urine | ERR10441695 |
| CZE | 2020 | DTU_2020_TWIW_01_CZE_PLZ_036 | Urine | ERR10441697 |
| CZE | 2020 | DTU_2020_TWIW_01_CZE_PLZ_037 | Urine | ERR10436659 |
| CZE | 2020 | DTU_2020_TWIW_01_CZE_PLZ_038 | Urine | ERR10436556 |
| CZE | 2020 | DTU_2020_TWIW_01_CZE_PLZ_039 | Urine | ERR10441700 |
| CZE | 2020 | DTU_2020_TWIW_01_CZE_PLZ_040 | Respiratory System | ERR10436780 |
| CZE | 2020 | DTU_2020_TWIW_01_CZE_PLZ_041 | Urine | ERR10441699 |
| CZE | 2020 | DTU_2020_TWIW_01_CZE_PLZ_042 | Urine | ERR10436585 |
| CZE | 2020 | DTU_2020_TWIW_01_CZE_PLZ_043 | Swab | ERR10436738 |
| CZE | 2020 | DTU_2020_TWIW_01_CZE_PLZ_044 | Swab | ERR10436736 |
| CZE | 2020 | DTU_2020_TWIW_01_CZE_PLZ_045 | Wound Pus Biopsy | ERR10441674 |
| CZE | 2020 | DTU_2020_TWIW_01_CZE_PLZ_046 | Wound Pus Biopsy | ERR10436654 |
| CZE | 2020 | DTU_2020_TWIW_01_CZE_PLZ_047 | Wound Pus Biopsy | ERR10436809 |
| CZE | 2020 | DTU_2020_TWIW_01_CZE_PLZ_048 | Urine | ERR10436748 |
| CZE | 2020 | DTU_2020_TWIW_01_CZE_PLZ_048 | Urine | ERR14150299 |
| CZE | 2020 | DTU_2020_TWIW_01_CZE_PLZ_049 | Wound Pus Biopsy | ERR10436655 |
| CZE | 2020 | DTU_2020_TWIW_01_CZE_PLZ_050 | Wound Pus Biopsy | ERR10436662 |
| CZE | 2020 | DTU_2020_TWIW_01_CZE_PLZ_051 | Other | ERR10436735 |
| CZE | 2020 | DTU_2020_TWIW_01_CZE_PLZ_052 | Wound Pus Biopsy | ERR10441680 |
| CZE | 2020 | DTU_2020_TWIW_01_CZE_PLZ_053 | Other | ERR10436523 |
| CZE | 2020 | DTU_2020_TWIW_01_CZE_PLZ_054 | Wound Pus Biopsy | ERR10436665 |
| CZE | 2020 | DTU_2020_TWIW_01_CZE_PLZ_056 | Wound Pus Biopsy | ERR10436672 |
| CZE | 2020 | DTU_2020_TWIW_01_CZE_PLZ_057 | Urine | ERR10441701 |
| CZE | 2020 | DTU_2020_TWIW_01_CZE_PLZ_058 | Respiratory System | ERR10436520 |
| CZE | 2020 | DTU_2020_TWIW_01_CZE_PLZ_059 | Swab | ERR10436737 |
| CZE | 2020 | DTU_2020_TWIW_01_CZE_PLZ_060 | Urine | ERR10441676 |
| FRA | 2020 | DTU_2020_TWIW_01_FRA_LIL_021A | Blood | ERR10437710 |
| THA | 2020 | DTU_2020_TWIW_01_THA_SAR_001 | Blood | ERR10432659 |
| THA | 2020 | DTU_2020_TWIW_01_THA_SAR_002 | Blood | ERR10432630 |
| THA | 2020 | DTU_2020_TWIW_01_THA_SAR_003 | Blood | ERR10432657 |
| THA | 2020 | DTU_2020_TWIW_01_THA_SAR_004 | Urine | ERR10432626 |
| THA | 2020 | DTU_2020_TWIW_01_THA_SAR_005 | Urine | ERR10432694 |
| THA | 2020 | DTU_2020_TWIW_01_THA_SAR_006 | Urine | ERR10432606 |
| THA | 2020 | DTU_2020_TWIW_01_THA_SAR_007 | Urine | ERR10432691 |
| THA | 2020 | DTU_2020_TWIW_01_THA_SAR_008 | Respiratory System | ERR10432611 |
| THA | 2020 | DTU_2020_TWIW_01_THA_SAR_009 | Respiratory System | ERR10432696 |
| THA | 2020 | DTU_2020_TWIW_01_THA_SAR_010 | Respiratory System | ERR10432612 |
| THA | 2020 | DTU_2020_TWIW_01_THA_SAR_011 | Respiratory System | ERR10432577 |
| THA | 2020 | DTU_2020_TWIW_01_THA_SAR_012 | Respiratory System | ERR10432616 |
| THA | 2020 | DTU_2020_TWIW_01_THA_SAR_013 | Respiratory System | ERR10432653 |
| THA | 2020 | DTU_2020_TWIW_01_THA_SAR_014 | Wound Pus Biopsy | ERR10432683 |
| THA | 2020 | DTU_2020_TWIW_01_THA_SAR_015 | Wound Pus Biopsy | ERR10432675 |
| THA | 2020 | DTU_2020_TWIW_01_THA_SAR_016 | Respiratory System | ERR10432687 |
| THA | 2020 | DTU_2020_TWIW_01_THA_SAR_017 | Respiratory System | ERR10432578 |
| THA | 2020 | DTU_2020_TWIW_01_THA_SAR_018 | Respiratory System | ERR10432600 |
| THA | 2020 | DTU_2020_TWIW_01_THA_SAR_019 | Respiratory System | ERR10432579 |
| THA | 2020 | DTU_2020_TWIW_01_THA_SAR_020 | Respiratory System | ERR10432613 |
| THA | 2020 | DTU_2020_TWIW_01_THA_SAR_021 | Wound Pus Biopsy | ERR10432680 |
| THA | 2020 | DTU_2020_TWIW_01_THA_SAR_022 | Respiratory System | ERR10432608 |
| THA | 2020 | DTU_2020_TWIW_01_THA_SAR_024 | Respiratory System | ERR10432610 |
| THA | 2020 | DTU_2020_TWIW_01_THA_SAR_025 | Respiratory System | ERR10432605 |
| THA | 2020 | DTU_2020_TWIW_01_THA_SAR_026 | Respiratory System | ERR10432614 |
| THA | 2020 | DTU_2020_TWIW_01_THA_SAR_027 | Respiratory System | ERR10432617 |
| THA | 2020 | DTU_2020_TWIW_01_THA_SAR_028 | Urine | ERR10432581 |
| THA | 2020 | DTU_2020_TWIW_01_THA_SAR_029 | Urine | ERR10432699 |
| THA | 2020 | DTU_2020_TWIW_01_THA_SAR_030 | Urine | ERR10432618 |
| THA | 2020 | DTU_2020_TWIW_01_THA_SAR_031 | Urine | ERR10432634 |
| THA | 2020 | DTU_2020_TWIW_01_THA_SAR_032 | Blood | ERR10432650 |
| THA | 2020 | DTU_2020_TWIW_01_THA_SAR_033 | Urine | ERR10432629 |
| THA | 2020 | DTU_2020_TWIW_01_THA_SAR_034 | Urine | ERR10432632 |
| THA | 2020 | DTU_2020_TWIW_01_THA_SAR_035 | Urine | ERR10432621 |
| THA | 2020 | DTU_2020_TWIW_01_THA_SAR_036 | Urine | ERR10432628 |
| THA | 2020 | DTU_2020_TWIW_01_THA_SAR_037 | Respiratory System | ERR10432619 |
| THA | 2020 | DTU_2020_TWIW_01_THA_SAR_038 | Respiratory System | ERR10432615 |
| THA | 2020 | DTU_2020_TWIW_01_THA_SAR_039 | Respiratory System | ERR10432661 |
| THA | 2020 | DTU_2020_TWIW_01_THA_SAR_040 | Blood | ERR10432609 |
| THA | 2020 | DTU_2020_TWIW_01_THA_SAR_041 | Blood | ERR10432662 |
| THA | 2020 | DTU_2020_TWIW_01_THA_SAR_042 | Blood | ERR10432645 |
| THA | 2020 | DTU_2020_TWIW_01_THA_SAR_043 | Blood | ERR10432703 |
| THA | 2020 | DTU_2020_TWIW_01_THA_SAR_044 | Blood | ERR10432635 |
| THA | 2020 | DTU_2020_TWIW_01_THA_SAR_045 | Blood | ERR10432636 |
| THA | 2020 | DTU_2020_TWIW_01_THA_SAR_046 | Wound Pus Biopsy | ERR10432643 |
| THA | 2020 | DTU_2020_TWIW_01_THA_SAR_047 | Wound Pus Biopsy | ERR10432695 |
| THA | 2020 | DTU_2020_TWIW_01_THA_SAR_048 | Wound Pus Biopsy | ERR10432640 |
| THA | 2020 | DTU_2020_TWIW_01_THA_SAR_049 | Wound Pus Biopsy | ERR10432658 |
| THA | 2020 | DTU_2020_TWIW_01_THA_SAR_050 | Wound Pus Biopsy | ERR10432644 |
| THA | 2020 | DTU_2020_TWIW_01_THA_SAR_051 | Blood | ERR10432585 |
| THA | 2020 | DTU_2020_TWIW_01_THA_SAR_052 | Blood | ERR10432684 |
| THA | 2020 | DTU_2020_TWIW_01_THA_SAR_053 | Respiratory System | ERR10432633 |
| THA | 2020 | DTU_2020_TWIW_01_THA_SAR_054 | Blood | ERR10432639 |
| THA | 2020 | DTU_2020_TWIW_01_THA_SAR_055 | Blood | ERR10432692 |
| THA | 2020 | DTU_2020_TWIW_01_THA_SAR_056 | Blood | ERR10432700 |
| THA | 2020 | DTU_2020_TWIW_01_THA_SAR_057 | Respiratory System | ERR10432690 |
| THA | 2020 | DTU_2020_TWIW_01_THA_SAR_058 | Urine | ERR10432623 |
| THA | 2020 | DTU_2020_TWIW_01_THA_SAR_059 | Urine | ERR10432642 |
| THA | 2020 | DTU_2020_TWIW_01_THA_SAR_060 | Blood | ERR10432654 |
| SAU | 2020 | DTU_2020_TWIW_01_SAU_A1Q_002 | Blood | ERR10432338 |
| SAU | 2020 | DTU_2020_TWIW_01_SAU_A1Q_003 | Urine | ERR10432369 |
| SAU | 2020 | DTU_2020_TWIW_01_SAU_A1Q_004 | Urine | ERR10432339 |
| SAU | 2020 | DTU_2020_TWIW_01_SAU_A1Q_005 | Blood | ERR10432347 |
| SAU | 2020 | DTU_2020_TWIW_01_SAU_A1Q_006 | Other | ERR10432376 |
| SAU | 2020 | DTU_2020_TWIW_01_SAU_A1Q_007 | Wound Pus Biopsy | ERR10432387 |
| SAU | 2020 | DTU_2020_TWIW_01_SAU_A1Q_008 | Wound Pus Biopsy | ERR10432375 |
| SAU | 2020 | DTU_2020_TWIW_01_SAU_A1Q_008 | Wound Pus Biopsy | ERR14129337 |
| SAU | 2020 | DTU_2020_TWIW_01_SAU_A1Q_009 | Wound Pus Biopsy | ERR10432328 |
| SAU | 2020 | DTU_2020_TWIW_01_SAU_A1Q_010 | Urine | ERR10432373 |
| SAU | 2020 | DTU_2020_TWIW_01_SAU_A1Q_011 | Urine | ERR10432345 |
| SAU | 2020 | DTU_2020_TWIW_01_SAU_A1Q_012 | Urine | ERR10432344 |
| SAU | 2020 | DTU_2020_TWIW_01_SAU_A1Q_013 | Urine | ERR10432377 |
| SAU | 2020 | DTU_2020_TWIW_01_SAU_A1Q_014 | Blood | ERR10432367 |
| SAU | 2020 | DTU_2020_TWIW_01_SAU_A1Q_015 | Blood | ERR10432341 |
| SAU | 2020 | DTU_2020_TWIW_01_SAU_A1Q_016 | Urine | ERR10432362 |
| SAU | 2020 | DTU_2020_TWIW_01_SAU_A1Q_017 | Urine | ERR10432378 |
| SAU | 2020 | DTU_2020_TWIW_01_SAU_A1Q_018 | Swab | ERR10432335 |
| SAU | 2020 | DTU_2020_TWIW_01_SAU_A1Q_019 | Respiratory System | ERR10432340 |
| SAU | 2020 | DTU_2020_TWIW_01_SAU_A1Q_020 | Blood | ERR10432388 |
| SAU | 2020 | DTU_2020_TWIW_01_SAU_A1Q_021 | Blood | ERR10432365 |
| SAU | 2020 | DTU_2020_TWIW_01_SAU_A1Q_022 | Urine | ERR10432331 |
| SAU | 2020 | DTU_2020_TWIW_01_SAU_A1Q_024 | Urine | ERR10432363 |
| SAU | 2020 | DTU_2020_TWIW_01_SAU_A1Q_025 | Urine | ERR10432353 |
| SAU | 2020 | DTU_2020_TWIW_01_SAU_A1Q_026 | Urine | ERR10432346 |
| SAU | 2020 | DTU_2020_TWIW_01_SAU_A1Q_027 | Urine | ERR10432385 |
| SAU | 2020 | DTU_2020_TWIW_01_SAU_A1Q_028 | Urine | ERR10432352 |
| SAU | 2020 | DTU_2020_TWIW_01_SAU_A1Q_029 | Wound Pus Biopsy | ERR10432354 |
| SAU | 2020 | DTU_2020_TWIW_01_SAU_A1Q_030 | Wound Pus Biopsy | ERR10432398 |
| SAU | 2020 | DTU_2020_TWIW_01_SAU_A1Q_031 | Urine | ERR10432348 |
| SAU | 2020 | DTU_2020_TWIW_01_SAU_A1Q_032 | Urine | ERR10432351 |
| SAU | 2020 | DTU_2020_TWIW_01_SAU_A1Q_033 | Urine | ERR10432357 |
| SAU | 2020 | DTU_2020_TWIW_01_SAU_A1Q_034 | Wound Pus Biopsy | ERR10432382 |
| SAU | 2020 | DTU_2020_TWIW_01_SAU_A1Q_035 | Blood | ERR10432393 |
| SAU | 2020 | DTU_2020_TWIW_01_SAU_A1Q_036 | Wound Pus Biopsy | ERR10432396 |
| SAU | 2020 | DTU_2020_TWIW_01_SAU_A1Q_037 | Wound Pus Biopsy | ERR10432350 |
| SAU | 2020 | DTU_2020_TWIW_01_SAU_A1Q_038 | Wound Pus Biopsy | ERR10432356 |
| SAU | 2020 | DTU_2020_TWIW_01_SAU_A1Q_039 | Wound Pus Biopsy | ERR10432389 |
| SAU | 2020 | DTU_2020_TWIW_01_SAU_A1Q_040 | Wound Pus Biopsy | ERR10432417 |
| SAU | 2020 | DTU_2020_TWIW_01_SAU_A1Q_041 | Urine | ERR10432359 |
| SAU | 2020 | DTU_2020_TWIW_01_SAU_A1Q_042 | Respiratory System | ERR10432355 |
| SAU | 2020 | DTU_2020_TWIW_01_SAU_A1Q_043 | Respiratory System | ERR10432386 |
| SAU | 2020 | DTU_2020_TWIW_01_SAU_A1Q_044 | Respiratory System | ERR10432336 |
| SAU | 2020 | DTU_2020_TWIW_01_SAU_A1Q_045 | Blood | ERR10432399 |
| SAU | 2020 | DTU_2020_TWIW_01_SAU_A1Q_046 | Urine | ERR10432360 |
| SAU | 2020 | DTU_2020_TWIW_01_SAU_A1Q_047 | Urine | ERR10432364 |
| SAU | 2020 | DTU_2020_TWIW_01_SAU_A1Q_048 | Wound Pus Biopsy | ERR10432392 |
| SAU | 2020 | DTU_2020_TWIW_01_SAU_A1Q_049 | Wound Pus Biopsy | ERR10432361 |
| SAU | 2020 | DTU_2020_TWIW_01_SAU_A1Q_050 | Other | ERR10432366 |
| SAU | 2020 | DTU_2020_TWIW_01_SAU_A1Q_051 | Blood | ERR10432380 |
| SAU | 2020 | DTU_2020_TWIW_01_SAU_A1Q_052 | Wound Pus Biopsy | ERR10432391 |
| SAU | 2020 | DTU_2020_TWIW_01_SAU_A1Q_053 | Wound Pus Biopsy | ERR10432381 |
| SAU | 2020 | DTU_2020_TWIW_01_SAU_A1Q_054 | Urine | ERR10432397 |
| SAU | 2020 | DTU_2020_TWIW_01_SAU_A1Q_055 | Urine | ERR10432368 |
| SAU | 2020 | DTU_2020_TWIW_01_SAU_A1Q_056 | Urine | ERR10432374 |
| SAU | 2020 | DTU_2020_TWIW_01_SAU_A1Q_057 | Urine | ERR10432371 |
| SAU | 2020 | DTU_2020_TWIW_01_SAU_A1Q_058 | Wound Pus Biopsy | ERR10432394 |
| SAU | 2020 | DTU_2020_TWIW_01_SAU_A1Q_059 | Urine | ERR10432390 |
| SAU | 2020 | DTU_2020_TWIW_01_SAU_A1Q_060 | Urine | ERR10432384 |
| SAU | 2020 | DTU_2020_TWIW_01_SAU_A1Q_061 | Urine | ERR10432370 |
| SAU | 2020 | DTU_2020_TWIW_01_SAU_A1Q_062 | Blood | ERR10432342 |
| SAU | 2020 | DTU_2020_TWIW_01_SAU_A1Q_063 | Urine | ERR10432400 |
| SAU | 2020 | DTU_2020_TWIW_01_SAU_A1Q_064 | Blood | ERR10432372 |
| SAU | 2020 | DTU_2020_TWIW_01_SAU_A1Q_065 | Blood | ERR10432383 |
| SAU | 2020 | DTU_2020_TWIW_01_SAU_A1Q_066 | Blood | ERR10432349 |
| SAU | 2020 | DTU_2020_TWIW_01_SAU_A1Q_047A | Urine | ERR10432358 |
| DNK | 2020 | DTU_2020_TWIW_01_DNK_HVI_001 | Wound Pus Biopsy | ERR10436971 |
| DNK | 2020 | DTU_2020_TWIW_01_DNK_HVI_002 | Wound Pus Biopsy | ERR10436973 |
| DNK | 2020 | DTU_2020_TWIW_01_DNK_HVI_005 | Blood | ERR10436899 |
| DNK | 2020 | DTU_2020_TWIW_01_DNK_HVI_006 | Blood | ERR10436876 |
| DNK | 2020 | DTU_2020_TWIW_01_DNK_HVI_008 | Respiratory System | ERR10436969 |
| DNK | 2020 | DTU_2020_TWIW_01_DNK_HVI_009 | Respiratory System | ERR10437060 |
| DNK | 2020 | DTU_2020_TWIW_01_DNK_HVI_011 | Swab | ERR10437005 |
| DNK | 2020 | DTU_2020_TWIW_01_DNK_HVI_012 | Swab | ERR10436981 |
| DNK | 2020 | DTU_2020_TWIW_01_DNK_HVI_013 | Swab | ERR10437027 |
| DNK | 2020 | DTU_2020_TWIW_01_DNK_HVI_015 | Swab | ERR10436983 |
| DNK | 2020 | DTU_2020_TWIW_01_DNK_HVI_016 | Swab | ERR10436979 |
| DNK | 2020 | DTU_2020_TWIW_01_DNK_HVI_017 | Swab | ERR10436982 |
| DNK | 2020 | DTU_2020_TWIW_01_DNK_HVI_018 | Swab | ERR10436997 |
| DNK | 2020 | DTU_2020_TWIW_01_DNK_HVI_019 | Swab | ERR10437016 |
| DNK | 2020 | DTU_2020_TWIW_01_DNK_HVI_020 | Swab | ERR10437030 |
| DNK | 2020 | DTU_2020_TWIW_01_DNK_HVI_022 | Wound Pus Biopsy | ERR10436995 |
| DNK | 2020 | DTU_2020_TWIW_01_DNK_HVI_023 | Wound Pus Biopsy | ERR10436994 |
| DNK | 2020 | DTU_2020_TWIW_01_DNK_HVI_024 | Wound Pus Biopsy | ERR10436996 |
| DNK | 2020 | DTU_2020_TWIW_01_DNK_HVI_025 | Wound Pus Biopsy | ERR10437006 |
| DNK | 2020 | DTU_2020_TWIW_01_DNK_HVI_026 | Wound Pus Biopsy | ERR10437008 |
| DNK | 2020 | DTU_2020_TWIW_01_DNK_HVI_027 | Wound Pus Biopsy | ERR10436968 |
| DNK | 2020 | DTU_2020_TWIW_01_DNK_HVI_028 | Wound Pus Biopsy | ERR10437015 |
| DNK | 2020 | DTU_2020_TWIW_01_DNK_HVI_030 | Wound Pus Biopsy | ERR10437028 |
| DNK | 2020 | DTU_2020_TWIW_01_DNK_HVI_031 | Wound Pus Biopsy | ERR10437010 |
| DNK | 2020 | DTU_2020_TWIW_01_DNK_HVI_032 | Urine | ERR10436866 |
| DNK | 2020 | DTU_2020_TWIW_01_DNK_HVI_033 | Urine | ERR10436875 |
| DNK | 2020 | DTU_2020_TWIW_01_DNK_HVI_034 | Urine | ERR10436896 |
| DNK | 2020 | DTU_2020_TWIW_01_DNK_HVI_035 | Urine | ERR10436959 |
| DNK | 2020 | DTU_2020_TWIW_01_DNK_HVI_036 | Urine | ERR10436895 |
| DNK | 2020 | DTU_2020_TWIW_01_DNK_HVI_037 | Urine | ERR10436946 |
| DNK | 2020 | DTU_2020_TWIW_01_DNK_HVI_038 | Urine | ERR10437047 |
| DNK | 2020 | DTU_2020_TWIW_01_DNK_HVI_039 | Urine | ERR10436877 |
| DNK | 2020 | DTU_2020_TWIW_01_DNK_HVI_040 | Urine | ERR10436894 |
| DNK | 2020 | DTU_2020_TWIW_01_DNK_HVI_041 | Urine | ERR10436884 |
| DNK | 2020 | DTU_2020_TWIW_01_DNK_HVI_043 | Urine | ERR10436829 |
| DNK | 2020 | DTU_2020_TWIW_01_DNK_HVI_044 | Urine | ERR10437034 |
| DNK | 2020 | DTU_2020_TWIW_01_DNK_HVI_045 | Urine | ERR10437059 |
| DNK | 2020 | DTU_2020_TWIW_01_DNK_HVI_046 | Urine | ERR10436844 |
| DNK | 2020 | DTU_2020_TWIW_01_DNK_HVI_047 | Urine | ERR10436902 |
| DNK | 2020 | DTU_2020_TWIW_01_DNK_HVI_048 | Urine | ERR10436853 |
| DNK | 2020 | DTU_2020_TWIW_01_DNK_HVI_049 | Urine | ERR10436961 |
| DNK | 2020 | DTU_2020_TWIW_01_DNK_HVI_050 | Urine | ERR10436845 |
| DNK | 2020 | DTU_2020_TWIW_01_DNK_HVI_051 | Urine | ERR10436901 |
| DNK | 2020 | DTU_2020_TWIW_01_DNK_HVI_052 | Urine | ERR10436842 |
| DNK | 2020 | DTU_2020_TWIW_01_DNK_HVI_053 | Urine | ERR10436859 |
| DNK | 2020 | DTU_2020_TWIW_01_DNK_HVI_054 | Urine | ERR10436858 |
| DNK | 2020 | DTU_2020_TWIW_01_DNK_HVI_055 | Urine | ERR10436904 |
| DNK | 2020 | DTU_2020_TWIW_01_DNK_HVI_056 | Urine | ERR10436874 |
| DNK | 2020 | DTU_2020_TWIW_01_DNK_HVI_057 | Urine | ERR10436919 |
| DNK | 2020 | DTU_2020_TWIW_01_DNK_HVI_058 | Urine | ERR10436912 |
| DNK | 2020 | DTU_2020_TWIW_01_DNK_HVI_059 | Urine | ERR10436843 |
| DNK | 2020 | DTU_2020_TWIW_01_DNK_HVI_060 | Urine | ERR10436926 |
| DNK | 2020 | DTU_2020_TWIW_01_DNK_HVI_061 | Urine | ERR10436925 |
| DNK | 2020 | DTU_2020_TWIW_01_DNK_HVI_062 | Urine | ERR10436935 |
| DNK | 2020 | DTU_2020_TWIW_01_DNK_HVI_063 | Urine | ERR10436932 |
| DNK | 2020 | DTU_2020_TWIW_01_DNK_HVI_064 | Urine | ERR10436929 |
| DNK | 2020 | DTU_2020_TWIW_01_DNK_HVI_065 | Urine | ERR10436942 |
| FRA | 2020 | DTU_2020_TWIW_01_FRA_VAL_001 | Blood | ERR10437606 |
| FRA | 2020 | DTU_2020_TWIW_01_FRA_VAL_002 | Urine | ERR10437576 |
| FRA | 2020 | DTU_2020_TWIW_01_FRA_VAL_003 | Urine | ERR10437531 |
| FRA | 2020 | DTU_2020_TWIW_01_FRA_VAL_004 | Blood | ERR10437671 |
| FRA | 2020 | DTU_2020_TWIW_01_FRA_VAL_005 | Other | ERR10437739 |
| FRA | 2020 | DTU_2020_TWIW_01_FRA_VAL_006 | Wound Pus Biopsy | ERR10437530 |
| FRA | 2020 | DTU_2020_TWIW_01_FRA_VAL_007 | Urine | ERR10437609 |
| FRA | 2020 | DTU_2020_TWIW_01_FRA_VAL_008 | Urine | ERR10437731 |
| FRA | 2020 | DTU_2020_TWIW_01_FRA_VAL_009 | Urine | ERR10437627 |
| FRA | 2020 | DTU_2020_TWIW_01_FRA_VAL_010 | Urine | ERR10437718 |
| FRA | 2020 | DTU_2020_TWIW_01_FRA_VAL_011 | Blood | ERR10437714 |
| FRA | 2020 | DTU_2020_TWIW_01_FRA_VAL_012 | Blood | ERR10437634 |
| FRA | 2020 | DTU_2020_TWIW_01_FRA_VAL_013 | Urine | ERR10437728 |
| FRA | 2020 | DTU_2020_TWIW_01_FRA_VAL_015 | Swab | ERR10437544 |
| FRA | 2020 | DTU_2020_TWIW_01_FRA_VAL_016 | Urine | ERR10437735 |
| FRA | 2020 | DTU_2020_TWIW_01_FRA_VAL_017 | Urine | ERR10437720 |
| FRA | 2020 | DTU_2020_TWIW_01_FRA_VAL_018 | Blood | ERR10437742 |
| FRA | 2020 | DTU_2020_TWIW_01_FRA_VAL_019 | Urine | ERR10437543 |
| FRA | 2020 | DTU_2020_TWIW_01_FRA_VAL_020 | Blood | ERR10437542 |
| FRA | 2020 | DTU_2020_TWIW_01_FRA_VAL_021 | Urine | ERR10437540 |
| FRA | 2020 | DTU_2020_TWIW_01_FRA_VAL_022 | Respiratory System | ERR10437732 |
| FRA | 2020 | DTU_2020_TWIW_01_FRA_VAL_023 | Blood | ERR10437558 |
| FRA | 2020 | DTU_2020_TWIW_01_FRA_VAL_024 | Urine | ERR10437559 |
| FRA | 2020 | DTU_2020_TWIW_01_FRA_VAL_025 | Blood | ERR10437600 |
| FRA | 2020 | DTU_2020_TWIW_01_FRA_VAL_026 | Blood | ERR10437704 |
| FRA | 2020 | DTU_2020_TWIW_01_FRA_VAL_027 | Urine | ERR10437601 |
| FRA | 2020 | DTU_2020_TWIW_01_FRA_VAL_028 | Respiratory System | ERR10437406 |
| FRA | 2020 | DTU_2020_TWIW_01_FRA_VAL_029 | Wound Pus Biopsy | ERR10437672 |
| FRA | 2020 | DTU_2020_TWIW_01_FRA_VAL_030 | Urine | ERR10437719 |
| FRA | 2020 | DTU_2020_TWIW_01_FRA_VAL_032 | Other | ERR10437674 |
| FRA | 2020 | DTU_2020_TWIW_01_FRA_VAL_033 | Urine | ERR10437722 |
| FRA | 2020 | DTU_2020_TWIW_01_FRA_VAL_034 | Blood | ERR10437703 |
| FRA | 2020 | DTU_2020_TWIW_01_FRA_VAL_035 | Blood | ERR10437706 |
| FRA | 2020 | DTU_2020_TWIW_01_FRA_VAL_036 | Urine | ERR10437556 |
| FRA | 2020 | DTU_2020_TWIW_01_FRA_VAL_037 | Other | ERR10437673 |
| FRA | 2020 | DTU_2020_TWIW_01_FRA_VAL_038 | Other | ERR10437641 |
| FRA | 2020 | DTU_2020_TWIW_01_FRA_VAL_039 | Blood | ERR10437557 |
| FRA | 2020 | DTU_2020_TWIW_01_FRA_VAL_040 | Blood | ERR10437567 |
| FRA | 2020 | DTU_2020_TWIW_01_FRA_VAL_041 | Swab | ERR10437737 |
| FRA | 2020 | DTU_2020_TWIW_01_FRA_VAL_042 | Urine | ERR10437575 |
| FRA | 2020 | DTU_2020_TWIW_01_FRA_VAL_043 | Urine | ERR10437561 |
| FRA | 2020 | DTU_2020_TWIW_01_FRA_VAL_044 | Blood | ERR10437738 |
| FRA | 2020 | DTU_2020_TWIW_01_FRA_VAL_045 | Blood | ERR10437687 |
| FRA | 2020 | DTU_2020_TWIW_01_FRA_VAL_047 | Blood | ERR10437707 |
| FRA | 2020 | DTU_2020_TWIW_01_FRA_VAL_048 | Urine | ERR10437562 |
| FRA | 2020 | DTU_2020_TWIW_01_FRA_VAL_049 | Blood | ERR10437685 |
| FRA | 2020 | DTU_2020_TWIW_01_FRA_VAL_050 | Blood | ERR10437639 |
| FRA | 2020 | DTU_2020_TWIW_01_FRA_VAL_051 | Wound Pus Biopsy | ERR10437741 |
| FRA | 2020 | DTU_2020_TWIW_01_FRA_VAL_052 | Urine | ERR10437578 |
| FRA | 2020 | DTU_2020_TWIW_01_FRA_VAL_053 | Urine | ERR10437560 |
| FRA | 2020 | DTU_2020_TWIW_01_FRA_VAL_054 | Urine | ERR10437565 |
| FRA | 2020 | DTU_2020_TWIW_01_FRA_VAL_055 | Blood | ERR10437577 |
| FRA | 2020 | DTU_2020_TWIW_01_FRA_VAL_056 | Blood | ERR10437564 |
| FRA | 2020 | DTU_2020_TWIW_01_FRA_VAL_057 | Respiratory System | ERR10437686 |
| FRA | 2020 | DTU_2020_TWIW_01_FRA_VAL_058 | Urine | ERR10437563 |
| FRA | 2020 | DTU_2020_TWIW_01_FRA_VAL_059 | Urine | ERR10437694 |
| FRA | 2020 | DTU_2020_TWIW_01_FRA_VAL_060 | Urine | ERR10437697 |
| FRA | 2020 | DTU_2020_TWIW_01_FRA_VAL_061 | Urine | ERR10437566 |
| FRA | 2020 | DTU_2020_TWIW_01_FRA_VAL_062 | Urine | ERR10437626 |
| FRA | 2020 | DTU_2020_TWIW_01_FRA_VAL_063 | Blood | ERR10437726 |
| FRA | 2020 | DTU_2020_TWIW_01_FRA_VAL_064 | Urine | ERR10437729 |
| FRA | 2020 | DTU_2020_TWIW_01_FRA_VAL_065 | Blood | ERR10437570 |
| FRA | 2020 | DTU_2020_TWIW_01_FRA_VAL_066 | Wound Pus Biopsy | ERR10437401 |
| FRA | 2020 | DTU_2020_TWIW_01_FRA_VAL_067 | Wound Pus Biopsy | ERR10437723 |
| FRA | 2020 | DTU_2020_TWIW_01_FRA_VAL_068 | Wound Pus Biopsy | ERR10437696 |
| FRA | 2020 | DTU_2020_TWIW_01_FRA_VAL_069 | Urine | ERR10437569 |
| FRA | 2020 | DTU_2020_TWIW_01_FRA_VAL_070 | Urine | ERR10437571 |
| HKG | 2020 | DTU_2020_TWIW_01_HKG_SHA_001 | Blood | ERR10438196 |
| HKG | 2020 | DTU_2020_TWIW_01_HKG_SHA_002 | Blood | ERR10438217 |
| HKG | 2020 | DTU_2020_TWIW_01_HKG_SHA_003 | Blood | ERR10438221 |
| HKG | 2020 | DTU_2020_TWIW_01_HKG_SHA_004 | Blood | ERR10438232 |
| HKG | 2020 | DTU_2020_TWIW_01_HKG_SHA_005 | Blood | ERR10438178 |
| HKG | 2020 | DTU_2020_TWIW_01_HKG_SHA_006 | Blood | ERR10438343 |
| HKG | 2020 | DTU_2020_TWIW_01_HKG_SHA_007 | Blood | ERR10438198 |
| HKG | 2020 | DTU_2020_TWIW_01_HKG_SHA_008 | Blood | ERR10438205 |
| HKG | 2020 | DTU_2020_TWIW_01_HKG_SHA_009 | Blood | ERR10438367 |
| HKG | 2020 | DTU_2020_TWIW_01_HKG_SHA_010 | Blood | ERR10438246 |
| HKG | 2020 | DTU_2020_TWIW_01_HKG_SHA_011 | Blood | ERR10438229 |
| HKG | 2020 | DTU_2020_TWIW_01_HKG_SHA_012 | Blood | ERR10438230 |
| HKG | 2020 | DTU_2020_TWIW_01_HKG_SHA_013 | Blood | ERR10438244 |
| HKG | 2020 | DTU_2020_TWIW_01_HKG_SHA_014 | Blood | ERR10438408 |
| HKG | 2020 | DTU_2020_TWIW_01_HKG_SHA_015 | Blood | ERR10438361 |
| HKG | 2020 | DTU_2020_TWIW_01_HKG_SHA_016 | Blood | ERR10438256 |
| HKG | 2020 | DTU_2020_TWIW_01_HKG_SHA_017 | Blood | ERR10438258 |
| HKG | 2020 | DTU_2020_TWIW_01_HKG_SHA_018 | Blood | ERR10438261 |
| HKG | 2020 | DTU_2020_TWIW_01_HKG_SHA_019 | Blood | ERR10438362 |
| HKG | 2020 | DTU_2020_TWIW_01_HKG_SHA_020 | Blood | ERR10438285 |
| HKG | 2020 | DTU_2020_TWIW_01_HKG_SHA_021 | Blood | ERR10438360 |
| HKG | 2020 | DTU_2020_TWIW_01_HKG_SHA_022 | Blood | ERR10438281 |
| HKG | 2020 | DTU_2020_TWIW_01_HKG_SHA_023 | Blood | ERR10438268 |
| HKG | 2020 | DTU_2020_TWIW_01_HKG_SHA_024 | Blood | ERR10438352 |
| HKG | 2020 | DTU_2020_TWIW_01_HKG_SHA_025 | Blood | ERR10438286 |
| HKG | 2020 | DTU_2020_TWIW_01_HKG_SHA_026 | Blood | ERR10438359 |
| HKG | 2020 | DTU_2020_TWIW_01_HKG_SHA_027 | Blood | ERR10438394 |
| HKG | 2020 | DTU_2020_TWIW_01_HKG_SHA_028 | Blood | ERR10438274 |
| HKG | 2020 | DTU_2020_TWIW_01_HKG_SHA_029 | Blood | ERR10438298 |
| HKG | 2020 | DTU_2020_TWIW_01_HKG_SHA_030 | Blood | ERR10438297 |
| HKG | 2020 | DTU_2020_TWIW_01_HKG_SHA_031 | Blood | ERR10438305 |
| HKG | 2020 | DTU_2020_TWIW_01_HKG_SHA_032 | Blood | ERR10438293 |
| HKG | 2020 | DTU_2020_TWIW_01_HKG_SHA_033 | Blood | ERR10438307 |
| HKG | 2020 | DTU_2020_TWIW_01_HKG_SHA_034 | Blood | ERR10438280 |
| HKG | 2020 | DTU_2020_TWIW_01_HKG_SHA_035 | Blood | ERR10438304 |
| HKG | 2020 | DTU_2020_TWIW_01_HKG_SHA_036 | Blood | ERR10438396 |
| HKG | 2020 | DTU_2020_TWIW_01_HKG_SHA_037 | Blood | ERR10438323 |
| HKG | 2020 | DTU_2020_TWIW_01_HKG_SHA_038 | Blood | ERR10438321 |
| HKG | 2020 | DTU_2020_TWIW_01_HKG_SHA_039 | Blood | ERR10438340 |
| HKG | 2020 | DTU_2020_TWIW_01_HKG_SHA_040 | Blood | ERR10438316 |
| HKG | 2020 | DTU_2020_TWIW_01_HKG_SHA_041 | Blood | ERR10438320 |
| HKG | 2020 | DTU_2020_TWIW_01_HKG_SHA_042 | Blood | ERR10438397 |
| HKG | 2020 | DTU_2020_TWIW_01_HKG_SHA_043 | Blood | ERR10438388 |
| HKG | 2020 | DTU_2020_TWIW_01_HKG_SHA_044 | Blood | ERR10438324 |
| HKG | 2020 | DTU_2020_TWIW_01_HKG_SHA_045 | Blood | ERR10438405 |
| HKG | 2020 | DTU_2020_TWIW_01_HKG_SHA_046 | Blood | ERR10438331 |
| HKG | 2020 | DTU_2020_TWIW_01_HKG_SHA_047 | Blood | ERR10438333 |
| HKG | 2020 | DTU_2020_TWIW_01_HKG_SHA_048 | Blood | ERR10438332 |
| HKG | 2020 | DTU_2020_TWIW_01_HKG_SHA_049 | Blood | ERR10438346 |
| HKG | 2020 | DTU_2020_TWIW_01_HKG_SHA_050 | Blood | ERR10438179 |
| HKG | 2020 | DTU_2020_TWIW_01_HKG_SHA_051 | Blood | ERR10438387 |
| HKG | 2020 | DTU_2020_TWIW_01_HKG_SHA_052 | Blood | ERR10438345 |
| HKG | 2020 | DTU_2020_TWIW_01_HKG_SHA_053 | Blood | ERR10438220 |
| HKG | 2020 | DTU_2020_TWIW_01_HKG_SHA_054 | Blood | ERR10438197 |
| HKG | 2020 | DTU_2020_TWIW_01_HKG_SHA_055 | Blood | ERR10438225 |
| HKG | 2020 | DTU_2020_TWIW_01_HKG_SHA_056 | Blood | ERR10438211 |
| HKG | 2020 | DTU_2020_TWIW_01_HKG_SHA_057 | Blood | ERR10438243 |
| HKG | 2020 | DTU_2020_TWIW_01_HKG_SHA_058 | Blood | ERR10438245 |
| HKG | 2020 | DTU_2020_TWIW_01_HKG_SHA_059 | Blood | ERR10438259 |
| HKG | 2020 | DTU_2020_TWIW_01_HKG_SHA_060 | Blood | ERR10438260 |
| GRL | 2020 | DTU_2020_TWIW_01_GRL_NUU_001 | Urine | ERR10438058 |
| GRL | 2020 | DTU_2020_TWIW_01_GRL_NUU_002 | Respiratory System | ERR10438153 |
| GRL | 2020 | DTU_2020_TWIW_01_GRL_NUU_003 | Swab | ERR10438152 |
| GRL | 2020 | DTU_2020_TWIW_01_GRL_NUU_004 | Swab | ERR10438104 |
| GRL | 2020 | DTU_2020_TWIW_01_GRL_NUU_005 | Swab | ERR10438103 |
| GRL | 2020 | DTU_2020_TWIW_01_GRL_NUU_006 | Respiratory System | ERR10438049 |
| GRL | 2020 | DTU_2020_TWIW_01_GRL_NUU_006 | Respiratory System | ERR12041383 |
| GRL | 2020 | DTU_2020_TWIW_01_GRL_NUU_007 | Urine | ERR10438050 |
| GRL | 2020 | DTU_2020_TWIW_01_GRL_NUU_008 | Urine | ERR10438054 |
| GRL | 2020 | DTU_2020_TWIW_01_GRL_NUU_009 | Urine | ERR10438046 |
| GRL | 2020 | DTU_2020_TWIW_01_GRL_NUU_010 | Urine | ERR10438052 |
| GRL | 2020 | DTU_2020_TWIW_01_GRL_NUU_011 | Urine | ERR10438151 |
| GRL | 2020 | DTU_2020_TWIW_01_GRL_NUU_012 | Urine | ERR10438055 |
| GRL | 2020 | DTU_2020_TWIW_01_GRL_NUU_013 | Urine | ERR10438056 |
| GRL | 2020 | DTU_2020_TWIW_01_GRL_NUU_014 | Swab | ERR10438120 |
| GRL | 2020 | DTU_2020_TWIW_01_GRL_NUU_016 | Swab | ERR10438129 |
| GRL | 2020 | DTU_2020_TWIW_01_GRL_NUU_017 | Swab | ERR10438162 |
| GRL | 2020 | DTU_2020_TWIW_01_GRL_NUU_018 | Swab | ERR10438163 |
| GRL | 2020 | DTU_2020_TWIW_01_GRL_NUU_020 | Urine | ERR10438057 |
| GRL | 2020 | DTU_2020_TWIW_01_GRL_NUU_021 | Urine | ERR10438059 |
| GRL | 2020 | DTU_2020_TWIW_01_GRL_NUU_022 | Swab | ERR10438164 |
| GRL | 2020 | DTU_2020_TWIW_01_GRL_NUU_023 | Swab | ERR10438086 |
| GRL | 2020 | DTU_2020_TWIW_01_GRL_NUU_024 | Swab | ERR10438113 |
| GRL | 2020 | DTU_2020_TWIW_01_GRL_NUU_025 | Swab | ERR10438112 |
| GRL | 2020 | DTU_2020_TWIW_01_GRL_NUU_027 | Swab | ERR10438121 |
| GRL | 2020 | DTU_2020_TWIW_01_GRL_NUU_028 | Urine | ERR10438060 |
| GRL | 2020 | DTU_2020_TWIW_01_GRL_NUU_029 | Urine | ERR10438061 |
| GRL | 2020 | DTU_2020_TWIW_01_GRL_NUU_030 | Swab | ERR10438122 |
| GRL | 2020 | DTU_2020_TWIW_01_GRL_NUU_031 | Swab | ERR10438131 |
| GRL | 2020 | DTU_2020_TWIW_01_GRL_NUU_032 | Urine | ERR10438062 |
| GRL | 2020 | DTU_2020_TWIW_01_GRL_NUU_033 | Urine | ERR10438063 |
| GRL | 2020 | DTU_2020_TWIW_01_GRL_NUU_034 | Urine | ERR10438066 |
| GRL | 2020 | DTU_2020_TWIW_01_GRL_NUU_035 | Urine | ERR10438064 |
| GRL | 2020 | DTU_2020_TWIW_01_GRL_NUU_037 | Urine | ERR10438172 |
| GRL | 2020 | DTU_2020_TWIW_01_GRL_NUU_038 | Urine | ERR10438073 |
| GRL | 2020 | DTU_2020_TWIW_01_GRL_NUU_039 | Wound Pus Biopsy | ERR10438132 |
| GRL | 2020 | DTU_2020_TWIW_01_GRL_NUU_040 | Wound Pus Biopsy | ERR10438130 |
| GRL | 2020 | DTU_2020_TWIW_01_GRL_NUU_044 | Urine | ERR10438171 |
| GRL | 2020 | DTU_2020_TWIW_01_GRL_NUU_045 | Urine | ERR10438053 |
| GRL | 2020 | DTU_2020_TWIW_01_GRL_NUU_046 | Blood | ERR10438074 |
| GRL | 2020 | DTU_2020_TWIW_01_GRL_NUU_047 | Urine | ERR10438095 |
| GRL | 2020 | DTU_2020_TWIW_01_GRL_NUU_048 | Swab | ERR10438142 |
| GRL | 2020 | DTU_2020_TWIW_01_GRL_NUU_049 | Other | ERR10438141 |
| GRL | 2020 | DTU_2020_TWIW_01_GRL_NUU_050 | Urine | ERR10438161 |
| GRL | 2020 | DTU_2020_TWIW_01_GRL_NUU_051 | Urine | ERR10438105 |
| GRL | 2020 | DTU_2020_TWIW_01_GRL_NUU_052 | Urine | ERR10438075 |
| GRL | 2020 | DTU_2020_TWIW_01_GRL_NUU_053 | Respiratory System | ERR10438143 |
| GRL | 2020 | DTU_2020_TWIW_01_GRL_NUU_055 | Other | ERR10438139 |
| GRL | 2020 | DTU_2020_TWIW_01_GRL_NUU_056 | Other | ERR10438140 |
| GRL | 2020 | DTU_2020_TWIW_01_GRL_NUU_058 | Urine | ERR10438051 |
| GRL | 2020 | DTU_2020_TWIW_01_GRL_NUU_059 | Urine | ERR10438096 |
| GRL | 2020 | DTU_2020_TWIW_01_GRL_NUU_060 | Urine | ERR10438097 |
| GRL | 2020 | DTU_2020_TWIW_01_GRL_NUU_041A | Respiratory System | ERR10438146 |
| GRL | 2020 | DTU_2020_TWIW_01_GRL_NUU_041A | Respiratory System | ERR12075676 |
| NOR | 2020 | DTU_2020_TWIW_01_NOR_LÃ˜R_001 | Urine | ERR10439696 |
| NOR | 2020 | DTU_2020_TWIW_01_NOR_LÃ˜R_002 | Urine | ERR10439697 |
| NOR | 2020 | DTU_2020_TWIW_01_NOR_LÃ˜R_003 | Urine | ERR10439701 |
| NOR | 2020 | DTU_2020_TWIW_01_NOR_LÃ˜R_004 | Urine | ERR10439694 |
| NOR | 2020 | DTU_2020_TWIW_01_NOR_LÃ˜R_005 | Urine | ERR10439702 |
| NOR | 2020 | DTU_2020_TWIW_01_NOR_LÃ˜R_006 | Urine | ERR10439699 |
| NOR | 2020 | DTU_2020_TWIW_01_NOR_LÃ˜R_007 | Urine | ERR10431456 |
| NOR | 2020 | DTU_2020_TWIW_01_NOR_LÃ˜R_008 | Urine | ERR10439700 |
| NOR | 2020 | DTU_2020_TWIW_01_NOR_LÃ˜R_009 | Urine | ERR10431438 |
| NOR | 2020 | DTU_2020_TWIW_01_NOR_LÃ˜R_010 | Urine | ERR10439704 |
| NOR | 2020 | DTU_2020_TWIW_01_NOR_LÃ˜R_011 | Urine | ERR10439055 |
| NOR | 2020 | DTU_2020_TWIW_01_NOR_LÃ˜R_013 | Wound Pus Biopsy | ERR10431451 |
| NOR | 2020 | DTU_2020_TWIW_01_NOR_LÃ˜R_015 | Wound Pus Biopsy | ERR10431423 |
| NOR | 2020 | DTU_2020_TWIW_01_NOR_LÃ˜R_016 | Respiratory System | ERR10431424 |
| NOR | 2020 | DTU_2020_TWIW_01_NOR_LÃ˜R_017 | Wound Pus Biopsy | ERR10431429 |
| NOR | 2020 | DTU_2020_TWIW_01_NOR_LÃ˜R_018 | Wound Pus Biopsy | ERR10431425 |
| NOR | 2020 | DTU_2020_TWIW_01_NOR_LÃ˜R_019 | Urine | ERR10439708 |
| NOR | 2020 | DTU_2020_TWIW_01_NOR_LÃ˜R_020 | Urine | ERR10439706 |
| NOR | 2020 | DTU_2020_TWIW_01_NOR_LÃ˜R_021 | Urine | ERR10431449 |
| NOR | 2020 | DTU_2020_TWIW_01_NOR_LÃ˜R_022 | Urine | ERR10439058 |
| NOR | 2020 | DTU_2020_TWIW_01_NOR_LÃ˜R_023 | Urine | ERR10431440 |
| NOR | 2020 | DTU_2020_TWIW_01_NOR_LÃ˜R_025 | Urine | ERR10431435 |
| NOR | 2020 | DTU_2020_TWIW_01_NOR_LÃ˜R_026 | Urine | ERR10431442 |
| NOR | 2020 | DTU_2020_TWIW_01_NOR_LÃ˜R_027 | Urine | ERR10439710 |
| NOR | 2020 | DTU_2020_TWIW_01_NOR_LÃ˜R_028 | Urine | ERR10431454 |
| NOR | 2020 | DTU_2020_TWIW_01_NOR_LÃ˜R_029 | Urine | ERR10439703 |
| NOR | 2020 | DTU_2020_TWIW_01_NOR_LÃ˜R_030 | Urine | ERR10439705 |
| NOR | 2020 | DTU_2020_TWIW_01_NOR_LÃ˜R_031 | Urine | ERR10431447 |
| NOR | 2020 | DTU_2020_TWIW_01_NOR_LÃ˜R_032 | Urine | ERR10439709 |
| NOR | 2020 | DTU_2020_TWIW_01_NOR_LÃ˜R_033 | Swab | ERR10431450 |
| NOR | 2020 | DTU_2020_TWIW_01_NOR_LÃ˜R_034 | Urine | ERR10431436 |
| NOR | 2020 | DTU_2020_TWIW_01_NOR_LÃ˜R_035 | Wound Pus Biopsy | ERR10431448 |
| NOR | 2020 | DTU_2020_TWIW_01_NOR_LÃ˜R_036 | Respiratory System | ERR10439698 |
| NOR | 2020 | DTU_2020_TWIW_01_NOR_LÃ˜R_037 | Respiratory System | ERR10431452 |
| NOR | 2020 | DTU_2020_TWIW_01_NOR_LÃ˜R_038 | Wound Pus Biopsy | ERR10431432 |
| NOR | 2020 | DTU_2020_TWIW_01_NOR_LÃ˜R_039 | Wound Pus Biopsy | ERR10439718 |
| NOR | 2020 | DTU_2020_TWIW_01_NOR_LÃ˜R_040 | Wound Pus Biopsy | ERR10431441 |
| NOR | 2020 | DTU_2020_TWIW_01_NOR_LÃ˜R_041 | Wound Pus Biopsy | ERR10431427 |
| NOR | 2020 | DTU_2020_TWIW_01_NOR_LÃ˜R_042 | Wound Pus Biopsy | ERR10431421 |
| NOR | 2020 | DTU_2020_TWIW_01_NOR_LÃ˜R_043 | Wound Pus Biopsy | ERR10431428 |
| NOR | 2020 | DTU_2020_TWIW_01_NOR_LÃ˜R_044 | Wound Pus Biopsy | ERR10431431 |
| NOR | 2020 | DTU_2020_TWIW_01_NOR_LÃ˜R_045 | Wound Pus Biopsy | ERR10431433 |
| NOR | 2020 | DTU_2020_TWIW_01_NOR_LÃ˜R_046 | Wound Pus Biopsy | ERR10431437 |
| NOR | 2020 | DTU_2020_TWIW_01_NOR_LÃ˜R_047 | Wound Pus Biopsy | ERR10431444 |
| NOR | 2020 | DTU_2020_TWIW_01_NOR_LÃ˜R_048 | Blood | ERR10439695 |
| NOR | 2020 | DTU_2020_TWIW_01_NOR_LÃ˜R_049 | Blood | ERR10439057 |
| NOR | 2020 | DTU_2020_TWIW_01_NOR_LÃ˜R_050 | Blood | ERR10431453 |
| NOR | 2020 | DTU_2020_TWIW_01_NOR_LÃ˜R_052 | Blood | ERR10439711 |
| NOR | 2020 | DTU_2020_TWIW_01_NOR_LÃ˜R_053 | Blood | ERR10431422 |
| NOR | 2020 | DTU_2020_TWIW_01_NOR_LÃ˜R_054 | Wound Pus Biopsy | ERR10431430 |
| NOR | 2020 | DTU_2020_TWIW_01_NOR_LÃ˜R_055 | Swab | ERR10431445 |
| NOR | 2020 | DTU_2020_TWIW_01_NOR_LÃ˜R_056 | Wound Pus Biopsy | ERR10431446 |
| GRL | 2020 | DTU_2020_TWIW_01_GRL_NUU_026A | Swab | ERR10438156 |
| CAN | 2020 | DTU_2020_TWIW_01_CAN_EDM_002 | Swab | ERR10441355 |
| CAN | 2020 | DTU_2020_TWIW_01_CAN_EDM_003 | Urine | ERR10441284 |
| CAN | 2020 | DTU_2020_TWIW_01_CAN_EDM_004 | Respiratory System | ERR10441274 |
| CAN | 2020 | DTU_2020_TWIW_01_CAN_EDM_005 | Swab | ERR10441314 |
| CAN | 2020 | DTU_2020_TWIW_01_CAN_EDM_007 | Respiratory System | ERR10441397 |
| CAN | 2020 | DTU_2020_TWIW_01_CAN_EDM_008 | Respiratory System | ERR10441399 |
| CAN | 2020 | DTU_2020_TWIW_01_CAN_EDM_009 | Respiratory System | ERR10441408 |
| CAN | 2020 | DTU_2020_TWIW_01_CAN_EDM_010 | Swab | ERR10441380 |
| CAN | 2020 | DTU_2020_TWIW_01_CAN_EDM_011 | Swab | ERR10441307 |
| CAN | 2020 | DTU_2020_TWIW_01_CAN_EDM_012 | Urine | ERR10446190 |
| CAN | 2020 | DTU_2020_TWIW_01_CAN_EDM_013 | Urine | ERR10441363 |
| CAN | 2020 | DTU_2020_TWIW_01_CAN_EDM_014 | Respiratory System | ERR10441313 |
| CAN | 2020 | DTU_2020_TWIW_01_CAN_EDM_015 | Respiratory System | ERR10441337 |
| CAN | 2020 | DTU_2020_TWIW_01_CAN_EDM_016 | Respiratory System | ERR10441393 |
| CAN | 2020 | DTU_2020_TWIW_01_CAN_EDM_017 | Urine | ERR10441373 |
| CAN | 2020 | DTU_2020_TWIW_01_CAN_EDM_018 | Wound Pus Biopsy | ERR10441273 |
| CAN | 2020 | DTU_2020_TWIW_01_CAN_EDM_020 | Blood | ERR10441319 |
| CAN | 2020 | DTU_2020_TWIW_01_CAN_EDM_020 | Blood | ERR14150304 |
| CAN | 2020 | DTU_2020_TWIW_01_CAN_EDM_021 | Other | ERR10441304 |
| CAN | 2020 | DTU_2020_TWIW_01_CAN_EDM_022 | Other | ERR10441288 |
| CAN | 2020 | DTU_2020_TWIW_01_CAN_EDM_023 | Swab | ERR10441356 |
| CAN | 2020 | DTU_2020_TWIW_01_CAN_EDM_024 | Respiratory System | ERR10441353 |
| CAN | 2020 | DTU_2020_TWIW_01_CAN_EDM_025 | Wound Pus Biopsy | ERR10441308 |
| CAN | 2020 | DTU_2020_TWIW_01_CAN_EDM_026 | Other | ERR10441421 |
| CAN | 2020 | DTU_2020_TWIW_01_CAN_EDM_027 | Respiratory System | ERR10441311 |
| CAN | 2020 | DTU_2020_TWIW_01_CAN_EDM_028 | Blood | ERR10441278 |
| CAN | 2020 | DTU_2020_TWIW_01_CAN_EDM_029 | Respiratory System | ERR10441386 |
| CAN | 2020 | DTU_2020_TWIW_01_CAN_EDM_030 | Respiratory System | ERR10441413 |
| CAN | 2020 | DTU_2020_TWIW_01_CAN_EDM_031 | Respiratory System | ERR10441418 |
| CAN | 2020 | DTU_2020_TWIW_01_CAN_EDM_032 | Wound Pus Biopsy | ERR10441366 |
| CAN | 2020 | DTU_2020_TWIW_01_CAN_EDM_033 | Wound Pus Biopsy | ERR10441411 |
| CAN | 2020 | DTU_2020_TWIW_01_CAN_EDM_035 | Blood | ERR10441352 |
| CAN | 2020 | DTU_2020_TWIW_01_CAN_EDM_036 | Other | ERR10441440 |
| CAN | 2020 | DTU_2020_TWIW_01_CAN_EDM_037 | Blood | ERR10441310 |
| CAN | 2020 | DTU_2020_TWIW_01_CAN_EDM_038 | Urine | ERR10441296 |
| CAN | 2020 | DTU_2020_TWIW_01_CAN_EDM_039 | Respiratory System | ERR10441392 |
| CAN | 2020 | DTU_2020_TWIW_01_CAN_EDM_041 | Respiratory System | ERR10441332 |
| CAN | 2020 | DTU_2020_TWIW_01_CAN_EDM_042 | Urine | ERR10441328 |
| CAN | 2020 | DTU_2020_TWIW_01_CAN_EDM_043 | Urine | ERR10441414 |
| CAN | 2020 | DTU_2020_TWIW_01_CAN_EDM_044 | Respiratory System | ERR10441329 |
| CAN | 2020 | DTU_2020_TWIW_01_CAN_EDM_045 | Other | ERR10441404 |
| CAN | 2020 | DTU_2020_TWIW_01_CAN_EDM_046 | Respiratory System | ERR10441370 |
| CAN | 2020 | DTU_2020_TWIW_01_CAN_EDM_047 | Swab | ERR10441372 |
| CAN | 2020 | DTU_2020_TWIW_01_CAN_EDM_048 | Blood | ERR10441325 |
| CAN | 2020 | DTU_2020_TWIW_01_CAN_EDM_049 | Other | ERR10441461 |
| CAN | 2020 | DTU_2020_TWIW_01_CAN_EDM_050 | Other | ERR10441400 |
| CAN | 2020 | DTU_2020_TWIW_01_CAN_EDM_051 | Other | ERR10441403 |
| CAN | 2020 | DTU_2020_TWIW_01_CAN_EDM_052 | Respiratory System | ERR10441280 |
| CAN | 2020 | DTU_2020_TWIW_01_CAN_EDM_053 | Swab | ERR10441326 |
| CAN | 2020 | DTU_2020_TWIW_01_CAN_EDM_054 | Respiratory System | ERR10441323 |
| CAN | 2020 | DTU_2020_TWIW_01_CAN_EDM_055 | Urine | ERR10441281 |
| CAN | 2020 | DTU_2020_TWIW_01_CAN_EDM_056 | Urine | ERR10441279 |
| CAN | 2020 | DTU_2020_TWIW_01_CAN_EDM_058 | Urine | ERR10441420 |
| CAN | 2020 | DTU_2020_TWIW_01_CAN_EDM_059 | Urine | ERR10441344 |
| CAN | 2020 | DTU_2020_TWIW_01_CAN_EDM_060 | Blood | ERR10441367 |
| AUS | 2020 | DTU_2020_TWIW_01_AUS_MUR_001 | Blood | ERR10431325 |
| AUS | 2020 | DTU_2020_TWIW_01_AUS_MUR_002 | Blood | ERR10431317 |
| AUS | 2020 | DTU_2020_TWIW_01_AUS_MUR_003 | Urine | ERR10430843 |
| AUS | 2020 | DTU_2020_TWIW_01_AUS_MUR_004 | Wound Pus Biopsy | ERR10446194 |
| AUS | 2020 | DTU_2020_TWIW_01_AUS_MUR_005 | Wound Pus Biopsy | ERR10431347 |
| AUS | 2020 | DTU_2020_TWIW_01_AUS_MUR_006 | Urine | ERR10431340 |
| AUS | 2020 | DTU_2020_TWIW_01_AUS_MUR_006 | Urine | ERR12075678 |
| AUS | 2020 | DTU_2020_TWIW_01_AUS_MUR_007 | Other | ERR10430849 |
| AUS | 2020 | DTU_2020_TWIW_01_AUS_MUR_008 | Blood | ERR10430795 |
| AUS | 2020 | DTU_2020_TWIW_01_AUS_MUR_009 | Blood | ERR10431349 |
| AUS | 2020 | DTU_2020_TWIW_01_AUS_MUR_010 | Other | ERR10431307 |
| AUS | 2020 | DTU_2020_TWIW_01_AUS_MUR_011 | Urine | ERR10430726 |
| AUS | 2020 | DTU_2020_TWIW_01_AUS_MUR_012 | Swab | ERR10431310 |
| AUS | 2020 | DTU_2020_TWIW_01_AUS_MUR_013 | Other | ERR10430303 |
| AUS | 2020 | DTU_2020_TWIW_01_AUS_MUR_014 | Wound Pus Biopsy | ERR10431321 |
| AUS | 2020 | DTU_2020_TWIW_01_AUS_MUR_015 | Blood | ERR10430800 |
| AUS | 2020 | DTU_2020_TWIW_01_AUS_MUR_016 | Blood | ERR10431302 |
| AUS | 2020 | DTU_2020_TWIW_01_AUS_MUR_017 | Respiratory System | ERR10431356 |
| AUS | 2020 | DTU_2020_TWIW_01_AUS_MUR_018 | Urine | ERR10430797 |
| AUS | 2020 | DTU_2020_TWIW_01_AUS_MUR_019 | Wound Pus Biopsy | ERR10430803 |
| AUS | 2020 | DTU_2020_TWIW_01_AUS_MUR_020 | Wound Pus Biopsy | ERR10431346 |
| AUS | 2020 | DTU_2020_TWIW_01_AUS_MUR_021 | Other | ERR10431344 |
| AUS | 2020 | DTU_2020_TWIW_01_AUS_MUR_023 | Swab | ERR10431303 |
| AUS | 2020 | DTU_2020_TWIW_01_AUS_MUR_024 | Wound Pus Biopsy | ERR10431315 |
| AUS | 2020 | DTU_2020_TWIW_01_AUS_MUR_025 | Wound Pus Biopsy | ERR10446201 |
| AUS | 2020 | DTU_2020_TWIW_01_AUS_MUR_026 | Wound Pus Biopsy | ERR10431305 |
| AUS | 2020 | DTU_2020_TWIW_01_AUS_MUR_026 | Wound Pus Biopsy | ERR14150384 |
| AUS | 2020 | DTU_2020_TWIW_01_AUS_MUR_027 | Urine | ERR10431369 |
| AUS | 2020 | DTU_2020_TWIW_01_AUS_MUR_028 | Blood | ERR10431335 |
| AUS | 2020 | DTU_2020_TWIW_01_AUS_MUR_029 | Wound Pus Biopsy | ERR10431338 |
| AUS | 2020 | DTU_2020_TWIW_01_AUS_MUR_030 | Urine | ERR10431362 |
| AUS | 2020 | DTU_2020_TWIW_01_AUS_MUR_031 | Urine | ERR10430727 |
| AUS | 2020 | DTU_2020_TWIW_01_AUS_MUR_032 | Blood | ERR10430779 |
| AUS | 2020 | DTU_2020_TWIW_01_AUS_MUR_033 | Other | ERR10431312 |
| AUS | 2020 | DTU_2020_TWIW_01_AUS_MUR_034 | Respiratory System | ERR10431314 |
| AUS | 2020 | DTU_2020_TWIW_01_AUS_MUR_035 | Wound Pus Biopsy | ERR10431326 |
| AUS | 2020 | DTU_2020_TWIW_01_AUS_MUR_036 | Wound Pus Biopsy | ERR10431348 |
| AUS | 2020 | DTU_2020_TWIW_01_AUS_MUR_037 | Urine | ERR10430725 |
| AUS | 2020 | DTU_2020_TWIW_01_AUS_MUR_038 | Urine | ERR10431322 |
| AUS | 2020 | DTU_2020_TWIW_01_AUS_MUR_039 | Wound Pus Biopsy | ERR10430799 |
| AUS | 2020 | DTU_2020_TWIW_01_AUS_MUR_040 | Blood | ERR10430723 |
| AUS | 2020 | DTU_2020_TWIW_01_AUS_MUR_041 | Urine | ERR10430844 |
| AUS | 2020 | DTU_2020_TWIW_01_AUS_MUR_042 | Swab | ERR10435897 |
| AUS | 2020 | DTU_2020_TWIW_01_AUS_MUR_043 | Swab | ERR10431323 |
| AUS | 2020 | DTU_2020_TWIW_01_AUS_MUR_044 | Urine | ERR10430798 |
| AUS | 2020 | DTU_2020_TWIW_01_AUS_MUR_045 | Urine | ERR10431372 |
| AUS | 2020 | DTU_2020_TWIW_01_AUS_MUR_046 | Swab | ERR10431311 |
| AUS | 2020 | DTU_2020_TWIW_01_AUS_MUR_047 | Wound Pus Biopsy | ERR10431318 |
| AUS | 2020 | DTU_2020_TWIW_01_AUS_MUR_048 | Urine | ERR10430851 |
| AUS | 2020 | DTU_2020_TWIW_01_AUS_MUR_049 | Swab | ERR10430296 |
| AUS | 2020 | DTU_2020_TWIW_01_AUS_MUR_051 | Urine | ERR10431308 |
| AUS | 2020 | DTU_2020_TWIW_01_AUS_MUR_052 | Urine | ERR10441129 |
| AUS | 2020 | DTU_2020_TWIW_01_AUS_MUR_053 | Urine | ERR10431309 |
| AUS | 2020 | DTU_2020_TWIW_01_AUS_MUR_054 | Urine | ERR10431343 |
| AUS | 2020 | DTU_2020_TWIW_01_AUS_MUR_055 | Urine | ERR10431345 |
| AUS | 2020 | DTU_2020_TWIW_01_AUS_MUR_056 | Other | ERR10430801 |
| AUS | 2020 | DTU_2020_TWIW_01_AUS_MUR_057 | Wound Pus Biopsy | ERR10431339 |
| AUS | 2020 | DTU_2020_TWIW_01_AUS_MUR_058 | Swab | ERR10431319 |
| AUS | 2020 | DTU_2020_TWIW_01_AUS_MUR_059 | Urine | ERR10431328 |
| AUS | 2020 | DTU_2020_TWIW_01_AUS_MUR_060 | Urine | ERR10430842 |
| AUS | 2020 | DTU_2020_TWIW_01_AUS_MUR_061 | Swab | ERR10431353 |
| AUS | 2020 | DTU_2020_TWIW_01_AUS_MUR_062 | Other | ERR10431367 |
| NGA | 2020 | DTU_2020_TWIW_01_NGA_ABU_001 | Swab | ERR10438968 |
| NGA | 2020 | DTU_2020_TWIW_01_NGA_ABU_002 | Urine | ERR10439012 |
| NGA | 2020 | DTU_2020_TWIW_01_NGA_ABU_003 | Blood | ERR10438992 |
| NGA | 2020 | DTU_2020_TWIW_01_NGA_ABU_004A | Wound Pus Biopsy | ERR10439027 |
| NGA | 2020 | DTU_2020_TWIW_01_NGA_ABU_005 | Urine | ERR10438996 |
| NGA | 2020 | DTU_2020_TWIW_01_NGA_ABU_006 | Wound Pus Biopsy | ERR10439022 |
| NGA | 2020 | DTU_2020_TWIW_01_NGA_ABU_008 | Wound Pus Biopsy | ERR10438991 |
| NGA | 2020 | DTU_2020_TWIW_01_NGA_ABU_009 | Blood | ERR10438993 |
| NGA | 2020 | DTU_2020_TWIW_01_NGA_ABU_010A | Blood | ERR10438994 |
| NGA | 2020 | DTU_2020_TWIW_01_NGA_ABU_011 | Wound Pus Biopsy | ERR10438967 |
| NGA | 2020 | DTU_2020_TWIW_01_NGA_ABU_012 | Blood | ERR10438990 |
| NGA | 2020 | DTU_2020_TWIW_01_NGA_ABU_013 | Swab | ERR10438983 |
| NGA | 2020 | DTU_2020_TWIW_01_NGA_ABU_014 | Urine | ERR10439016 |
| NGA | 2020 | DTU_2020_TWIW_01_NGA_ABU_015 | Swab | ERR10438846 |
| NGA | 2020 | DTU_2020_TWIW_01_NGA_ABU_016 | Wound Pus Biopsy | ERR10438856 |
| NGA | 2020 | DTU_2020_TWIW_01_NGA_ABU_017 | Wound Pus Biopsy | ERR10438857 |
| NGA | 2020 | DTU_2020_TWIW_01_NGA_ABU_018 | Wound Pus Biopsy | ERR10438847 |
| NGA | 2020 | DTU_2020_TWIW_01_NGA_ABU_019 | Wound Pus Biopsy | ERR10438910 |
| NGA | 2020 | DTU_2020_TWIW_01_NGA_ABU_020A | Swab | ERR10438853 |
| NGA | 2020 | DTU_2020_TWIW_01_NGA_ABU_022 | Blood | ERR10438912 |
| NGA | 2020 | DTU_2020_TWIW_01_NGA_ABU_023 | Blood | ERR10438817 |
| NGA | 2020 | DTU_2020_TWIW_01_NGA_ABU_024 | Blood | ERR10438855 |
| NGA | 2020 | DTU_2020_TWIW_01_NGA_ABU_025 | Blood | ERR10439021 |
| NGA | 2020 | DTU_2020_TWIW_01_NGA_ABU_026 | Blood | ERR10438918 |
| NGA | 2020 | DTU_2020_TWIW_01_NGA_ABU_027 | Wound Pus Biopsy | ERR10438916 |
| NGA | 2020 | DTU_2020_TWIW_01_NGA_ABU_028 | Urine | ERR10438862 |
| NGA | 2020 | DTU_2020_TWIW_01_NGA_ABU_029 | Urine | ERR10438848 |
| NGA | 2020 | DTU_2020_TWIW_01_NGA_ABU_030 | Urine | ERR10438852 |
| NGA | 2020 | DTU_2020_TWIW_01_NGA_ABU_032 | Wound Pus Biopsy | ERR10438861 |
| NGA | 2020 | DTU_2020_TWIW_01_NGA_ABU_033A | Blood | ERR10438914 |
| NGA | 2020 | DTU_2020_TWIW_01_NGA_ABU_034 | Urine | ERR10438854 |
| NGA | 2020 | DTU_2020_TWIW_01_NGA_ABU_035 | Urine | ERR10438959 |
| NGA | 2020 | DTU_2020_TWIW_01_NGA_ABU_036 | Urine | ERR10438851 |
| NGA | 2020 | DTU_2020_TWIW_01_NGA_ABU_037 | Urine | ERR10439031 |
| NGA | 2020 | DTU_2020_TWIW_01_NGA_ABU_038 | Respiratory System | ERR10438908 |
| NGA | 2020 | DTU_2020_TWIW_01_NGA_ABU_039 | Blood | ERR10439033 |
| NGA | 2020 | DTU_2020_TWIW_01_NGA_ABU_040 | Blood | ERR14129341 |
| NGA | 2020 | DTU_2020_TWIW_01_NGA_ABU_040 | Blood | ERR10438913 |
| NGA | 2020 | DTU_2020_TWIW_01_NGA_ABU_041 | Wound Pus Biopsy | ERR10438957 |
| NGA | 2020 | DTU_2020_TWIW_01_NGA_ABU_042 | Blood | ERR10438984 |
| NGA | 2020 | DTU_2020_TWIW_01_NGA_ABU_043 | Wound Pus Biopsy | ERR10438995 |
| NGA | 2020 | DTU_2020_TWIW_01_NGA_ABU_045 | Wound Pus Biopsy | ERR10438966 |
| NGA | 2020 | DTU_2020_TWIW_01_NGA_ABU_047 | Urine | ERR10438859 |
| NGA | 2020 | DTU_2020_TWIW_01_NGA_ABU_048 | Blood | ERR10438998 |
| NGA | 2020 | DTU_2020_TWIW_01_NGA_ABU_049 | Urine | ERR10438986 |
| NGA | 2020 | DTU_2020_TWIW_01_NGA_ABU_050 | Blood | ERR10438909 |
| NGA | 2020 | DTU_2020_TWIW_01_NGA_ABU_051 | Blood | ERR10438915 |
| NGA | 2020 | DTU_2020_TWIW_01_NGA_ABU_052 | Wound Pus Biopsy | ERR10439036 |
| NGA | 2020 | DTU_2020_TWIW_01_NGA_ABU_053 | Urine | ERR10438849 |
| NGA | 2020 | DTU_2020_TWIW_01_NGA_ABU_054 | Urine | ERR10438964 |
| NGA | 2020 | DTU_2020_TWIW_01_NGA_ABU_04B | Wound Pus Biopsy | ERR10439034 |
| NGA | 2020 | DTU_2020_TWIW_01_NGA_ABU_010B | Blood | ERR10439042 |
| NGA | 2020 | DTU_2020_TWIW_01_NGA_ABU_010C | Blood | ERR10438845 |
| NGA | 2020 | DTU_2020_TWIW_01_NGA_ABU_020B | Swab | ERR10438961 |
| NGA | 2020 | DTU_2020_TWIW_01_NGA_ABU_020C | Swab | ERR10438917 |
| NGA | 2020 | DTU_2020_TWIW_01_NGA_ABU_033B | Urine | ERR10438850 |
| CAN | 2020 | DTU_2020_TWIW_01_CAN_EDM_051B | Other | ERR10441321 |
| NGA | 2020 | DTU_2020_TWIW_01_NGA_ABU_001A | Swab | ERR10438969 |
| NGA | 2020 | DTU_2020_TWIW_01_NGA_ABU_033C | Blood | ERR10439017 |
| ALB | 2020 | DTU_2020_TWIW_01_ALB_TIR_003 | Respiratory System | ERR10430236 |
| ALB | 2020 | DTU_2020_TWIW_01_ALB_TIR_004 | Urine | ERR10430227 |
| ALB | 2020 | DTU_2020_TWIW_01_ALB_TIR_005 | Respiratory System | ERR10430234 |
| ALB | 2020 | DTU_2020_TWIW_01_ALB_TIR_006 | Swab | ERR10430209 |
| ALB | 2020 | DTU_2020_TWIW_01_ALB_TIR_007 | Swab | ERR10430211 |
| ALB | 2020 | DTU_2020_TWIW_01_ALB_TIR_009 | Respiratory System | ERR10423588 |
| ALB | 2020 | DTU_2020_TWIW_01_ALB_TIR_010 | Wound Pus Biopsy | ERR10430230 |
| ALB | 2020 | DTU_2020_TWIW_01_ALB_TIR_011 | Respiratory System | ERR10430233 |
| ALB | 2020 | DTU_2020_TWIW_01_ALB_TIR_012 | Respiratory System | ERR10430243 |
| ALB | 2020 | DTU_2020_TWIW_01_ALB_TIR_013 | Wound Pus Biopsy | ERR10430208 |
| ALB | 2020 | DTU_2020_TWIW_01_ALB_TIR_014 | Wound Pus Biopsy | ERR10430207 |
| ALB | 2020 | DTU_2020_TWIW_01_ALB_TIR_015 | Urine | ERR10436005 |
| ALB | 2020 | DTU_2020_TWIW_01_ALB_TIR_016 | Wound Pus Biopsy | ERR10430225 |
| ALB | 2020 | DTU_2020_TWIW_01_ALB_TIR_016 | Wound Pus Biopsy | ERR12075675 |
| ALB | 2020 | DTU_2020_TWIW_01_ALB_TIR_018 | Swab | ERR10430228 |
| ALB | 2020 | DTU_2020_TWIW_01_ALB_TIR_019 | Blood | ERR10441117 |
| ALB | 2020 | DTU_2020_TWIW_01_ALB_TIR_020 | Swab | ERR10441082 |
| ALB | 2020 | DTU_2020_TWIW_01_ALB_TIR_021 | Respiratory System | ERR10430216 |
| ALB | 2020 | DTU_2020_TWIW_01_ALB_TIR_023 | Respiratory System | ERR10430223 |
| ALB | 2020 | DTU_2020_TWIW_01_ALB_TIR_024 | Respiratory System | ERR10430204 |
| ALB | 2020 | DTU_2020_TWIW_01_ALB_TIR_025 | Respiratory System | ERR10430206 |
| ALB | 2020 | DTU_2020_TWIW_01_ALB_TIR_026 | Respiratory System | ERR10430224 |
| ALB | 2020 | DTU_2020_TWIW_01_ALB_TIR_027 | Respiratory System | ERR10430235 |
| ALB | 2020 | DTU_2020_TWIW_01_ALB_TIR_028 | Other | ERR10430214 |
| ALB | 2020 | DTU_2020_TWIW_01_ALB_TIR_029 | Respiratory System | ERR10441116 |
| ALB | 2020 | DTU_2020_TWIW_01_ALB_TIR_030 | Respiratory System | ERR10430238 |
| ALB | 2020 | DTU_2020_TWIW_01_ALB_TIR_031 | Respiratory System | ERR10430220 |
| ALB | 2020 | DTU_2020_TWIW_01_ALB_TIR_032 | Respiratory System | ERR10441089 |
| ALB | 2020 | DTU_2020_TWIW_01_ALB_TIR_033 | Respiratory System | ERR10435996 |
| ALB | 2020 | DTU_2020_TWIW_01_ALB_TIR_034 | Urine | ERR10430221 |
| ALB | 2020 | DTU_2020_TWIW_01_ALB_TIR_035 | Swab | ERR10430213 |
| ALB | 2020 | DTU_2020_TWIW_01_ALB_TIR_036 | Respiratory System | ERR10430218 |
| ALB | 2020 | DTU_2020_TWIW_01_ALB_TIR_037 | Blood | ERR10430231 |
| ALB | 2020 | DTU_2020_TWIW_01_ALB_TIR_038 | Respiratory System | ERR10441128 |
| ALB | 2020 | DTU_2020_TWIW_01_ALB_TIR_041 | Respiratory System | ERR10446198 |
| ALB | 2020 | DTU_2020_TWIW_01_ALB_TIR_042 | Respiratory System | ERR10430210 |
| ALB | 2020 | DTU_2020_TWIW_01_ALB_TIR_043 | Respiratory System | ERR10430219 |
| ALB | 2020 | DTU_2020_TWIW_01_ALB_TIR_044 | Urine | ERR10430215 |
| ALB | 2020 | DTU_2020_TWIW_01_ALB_TIR_045 | Swab | ERR10430205 |
| ALB | 2020 | DTU_2020_TWIW_01_ALB_TIR_046 | Wound Pus Biopsy | ERR10430226 |
| ALB | 2020 | DTU_2020_TWIW_01_ALB_TIR_047 | Urine | ERR10430229 |
| ALB | 2020 | DTU_2020_TWIW_01_ALB_TIR_048 | Respiratory System | ERR10430240 |
| ALB | 2020 | DTU_2020_TWIW_01_ALB_TIR_049 | Respiratory System | ERR10441100 |
| ALB | 2020 | DTU_2020_TWIW_01_ALB_TIR_050 | Respiratory System | ERR10430301 |
| ALB | 2020 | DTU_2020_TWIW_01_ALB_TIR_051 | Swab | ERR10430217 |
| ALB | 2020 | DTU_2020_TWIW_01_ALB_TIR_052 | Swab | ERR10441112 |
| ALB | 2020 | DTU_2020_TWIW_01_ALB_TIR_053 | Urine | ERR10441121 |
| ALB | 2020 | DTU_2020_TWIW_01_ALB_TIR_054 | Respiratory System | ERR10430237 |
| ALB | 2020 | DTU_2020_TWIW_01_ALB_TIR_055 | Respiratory System | ERR10441120 |
| ALB | 2020 | DTU_2020_TWIW_01_ALB_TIR_056 | Wound Pus Biopsy | ERR10441091 |
| ALB | 2020 | DTU_2020_TWIW_01_ALB_TIR_057 | Wound Pus Biopsy | ERR10430284 |
| ALB | 2020 | DTU_2020_TWIW_01_ALB_TIR_058 | Respiratory System | ERR10436060 |
| ALB | 2020 | DTU_2020_TWIW_01_ALB_TIR_060 | Swab | ERR10441088 |
| ALB | 2020 | DTU_2020_TWIW_01_ALB_TIR_008A | Respiratory System | ERR10441124 |
| ALB | 2020 | DTU_2020_TWIW_01_ALB_TIR_032A | Respiratory System | ERR10441067 |
| ALB | 2020 | DTU_2020_TWIW_01_ALB_TIR_033A | Respiratory System | ERR10430232 |
| ALB | 2020 | DTU_2020_TWIW_01_ALB_TIR_037A | Blood | ERR10441114 |
| ALB | 2020 | DTU_2020_TWIW_01_ALB_TIR_049A | Respiratory System | ERR10430305 |
| BFA | 2020 | DTU_2020_TWIN_01_BFA_DED_015 | Urine | ERR10441267 |
| BFA | 2020 | DTU_2020_TWIN_01_BFA_DED_016 | Urine | ERR10441236 |
| BFA | 2020 | DTU_2020_TWIN_01_BFA_DED_017 | Urine | ERR10441251 |
| BFA | 2020 | DTU_2020_TWIN_01_BFA_DED_018 | Urine | ERR10441225 |
| BFA | 2020 | DTU_2020_TWIN_01_BFA_DED_019 | Wound Pus Biopsy | ERR10441259 |
| BFA | 2020 | DTU_2020_TWIN_01_BFA_DED_020 | Urine | ERR10441242 |
| BFA | 2020 | DTU_2020_TWIN_01_BFA_DED_021 | Swab | ERR10441227 |
| BFA | 2020 | DTU_2020_TWIN_01_BFA_OUA_001 | Urine | ERR10441258 |
| BFA | 2020 | DTU_2020_TWIN_01_BFA_OUA_002 | Urine | ERR10441214 |
| BFA | 2020 | DTU_2020_TWIN_01_BFA_OUA_003 | Urine | ERR10441266 |
| BFA | 2020 | DTU_2020_TWIN_01_BFA_OUA_004 | Urine | ERR10441239 |
| BFA | 2020 | DTU_2020_TWIN_01_BFA_OUA_005 | Urine | ERR10441191 |
| BFA | 2020 | DTU_2020_TWIN_01_BFA_OUA_006 | Urine | ERR10441264 |
| BFA | 2020 | DTU_2020_TWIN_01_BFA_OUA_007 | Urine | ERR10441318 |
| BFA | 2020 | DTU_2020_TWIN_01_BFA_OUA_008 | Urine | ERR10441150 |
| BFA | 2020 | DTU_2020_TWIN_01_BFA_OUA_009 | Other | ERR10446195 |
| BFA | 2020 | DTU_2020_TWIN_01_BFA_OUA_010 | Urine | ERR10441250 |
| BFA | 2020 | DTU_2020_TWIN_01_BFA_OUA_011 | Wound Pus Biopsy | ERR14150391 |
| BFA | 2020 | DTU_2020_TWIN_01_BFA_OUA_011 | Wound Pus Biopsy | ERR10441196 |
| BFA | 2020 | DTU_2020_TWIN_01_BFA_OUA_012 | Urine | ERR10441213 |
| BFA | 2020 | DTU_2020_TWIN_01_BFA_OUA_013 | Urine | ERR10441232 |
| BFA | 2020 | DTU_2020_TWIN_01_BFA_OUA_015 | Urine | ERR10441198 |
| BFA | 2020 | DTU_2020_TWIN_01_BFA_OUA_017 | Urine | ERR10446197 |
| BFA | 2020 | DTU_2020_TWIN_01_BFA_OUA_018 | Wound Pus Biopsy | ERR10441282 |
| BFA | 2020 | DTU_2020_TWIN_01_BFA_OUA_019 | Urine | ERR10441269 |
| BFA | 2020 | DTU_2020_TWIN_01_BFA_OUA_020 | Urine | ERR10441272 |
| BFA | 2020 | DTU_2020_TWIN_01_BFA_OUA_021 | Urine | ERR10441271 |
| BFA | 2020 | DTU_2020_TWIN_01_BFA_OUA_022 | Urine | ERR10441203 |
| BFA | 2020 | DTU_2020_TWIN_01_BFA_OUA_023 | Other | ERR10441268 |
| BFA | 2020 | DTU_2020_TWIN_01_BFA_OUA_024 | Urine | ERR10441235 |
| BFA | 2020 | DTU_2020_TWIN_01_BFA_OUA_025 | Urine | ERR10441158 |
| BFA | 2020 | DTU_2020_TWIN_01_BFA_OUA_026 | Urine | ERR10441228 |
| BFA | 2020 | DTU_2020_TWIN_01_BFA_OUA_027 | Urine | ERR10441200 |
| BFA | 2020 | DTU_2020_TWIN_01_BFA_OUA_028 | Wound Pus Biopsy | ERR10441452 |
| BFA | 2020 | DTU_2020_TWIN_01_BFA_OUA_029 | Urine | ERR10441224 |
| BFA | 2020 | DTU_2020_TWIN_01_BFA_OUA_030 | Urine | ERR10441226 |
| BFA | 2020 | DTU_2020_TWIN_01_BFA_OUA_032 | Swab | ERR10441167 |
| BFA | 2020 | DTU_2020_TWIN_01_BFA_OUA_033 | Urine | ERR10441222 |
| BFA | 2020 | DTU_2020_TWIN_01_BFA_OUA_035 | Other | ERR10441221 |
| BFA | 2020 | DTU_2020_TWIN_01_BFA_OUA_036 | Urine | ERR10441290 |
| BFA | 2020 | DTU_2020_TWIN_01_BFA_OUA_003A | Urine | ERR10441175 |
| BFA | 2020 | DTU_2020_TWIN_01_BFA_OUA_008A | Urine | ERR10441169 |
| BFA | 2020 | DTU_2020_TWIN_01_BFA_OUA_010A | Urine | ERR10441216 |
| BFA | 2020 | DTU_2020_TWIN_01_BFA_OUA_013A | Urine | ERR10441275 |
| BFA | 2020 | DTU_2020_TWIN_01_BFA_OUA_020A | Urine | ERR10441276 |
| BFA | 2020 | DTU_2020_TWIN_01_BFA_OUA_025A | Urine | ERR10441133 |
| CHE | 2020 | DTU_2020_TWIW_01_CHE_LUZ_001 | Urine | ERR10432463 |
| CHE | 2020 | DTU_2020_TWIW_01_CHE_LUZ_002 | Wound Pus Biopsy | ERR10432497 |
| CHE | 2020 | DTU_2020_TWIW_01_CHE_LUZ_004 | Urine | ERR10432501 |
| CHE | 2020 | DTU_2020_TWIW_01_CHE_LUZ_005 | Wound Pus Biopsy | ERR10432567 |
| CHE | 2020 | DTU_2020_TWIW_01_CHE_LUZ_006 | Urine | ERR10432478 |
| CHE | 2020 | DTU_2020_TWIW_01_CHE_LUZ_007 | Wound Pus Biopsy | ERR10432574 |
| CHE | 2020 | DTU_2020_TWIW_01_CHE_LUZ_008 | Swab | ERR10432562 |
| CHE | 2020 | DTU_2020_TWIW_01_CHE_LUZ_010 | Wound Pus Biopsy | ERR10432516 |
| CHE | 2020 | DTU_2020_TWIW_01_CHE_LUZ_012 | Swab | ERR10432461 |
| CHE | 2020 | DTU_2020_TWIW_01_CHE_LUZ_013 | Urine | ERR10432462 |
| CHE | 2020 | DTU_2020_TWIW_01_CHE_LUZ_015 | Urine | ERR10432564 |
| CHE | 2020 | DTU_2020_TWIW_01_CHE_LUZ_016 | Other | ERR10432563 |
| CHE | 2020 | DTU_2020_TWIW_01_CHE_LUZ_017 | Urine | ERR10432510 |
| CHE | 2020 | DTU_2020_TWIW_01_CHE_LUZ_018 | Wound Pus Biopsy | ERR10432464 |
| CHE | 2020 | DTU_2020_TWIW_01_CHE_LUZ_019 | Urine | ERR10432540 |
| CHE | 2020 | DTU_2020_TWIW_01_CHE_LUZ_020 | Urine | ERR10432458 |
| CHE | 2020 | DTU_2020_TWIW_01_CHE_LUZ_022 | Urine | ERR10432490 |
| CHE | 2020 | DTU_2020_TWIW_01_CHE_LUZ_023 | Urine | ERR10432474 |
| CHE | 2020 | DTU_2020_TWIW_01_CHE_LUZ_024 | Urine | ERR10432509 |
| CHE | 2020 | DTU_2020_TWIW_01_CHE_LUZ_025 | Wound Pus Biopsy | ERR10432505 |
| CHE | 2020 | DTU_2020_TWIW_01_CHE_LUZ_026 | Wound Pus Biopsy | ERR10432515 |
| CHE | 2020 | DTU_2020_TWIW_01_CHE_LUZ_027 | Urine | ERR10432559 |
| CHE | 2020 | DTU_2020_TWIW_01_CHE_LUZ_028 | Urine | ERR10432470 |
| CHE | 2020 | DTU_2020_TWIW_01_CHE_LUZ_029 | Urine | ERR10432466 |
| CHE | 2020 | DTU_2020_TWIW_01_CHE_LUZ_030 | Urine | ERR10432561 |
| CHE | 2020 | DTU_2020_TWIW_01_CHE_LUZ_031 | Urine | ERR10432589 |
| CHE | 2020 | DTU_2020_TWIW_01_CHE_LUZ_032 | Urine | ERR10432592 |
| CHE | 2020 | DTU_2020_TWIW_01_CHE_LUZ_033 | Blood | ERR10432506 |
| CHE | 2020 | DTU_2020_TWIW_01_CHE_LUZ_034 | Other | ERR10432534 |
| CHE | 2020 | DTU_2020_TWIW_01_CHE_LUZ_035 | Blood | ERR10432486 |
| CHE | 2020 | DTU_2020_TWIW_01_CHE_LUZ_036 | Urine | ERR10432468 |
| CHE | 2020 | DTU_2020_TWIW_01_CHE_LUZ_037 | Urine | ERR10432570 |
| CHE | 2020 | DTU_2020_TWIW_01_CHE_LUZ_038 | Swab | ERR10432544 |
| CHE | 2020 | DTU_2020_TWIW_01_CHE_LUZ_039 | Urine | ERR10432572 |
| CHE | 2020 | DTU_2020_TWIW_01_CHE_LUZ_040 | Blood | ERR10432569 |
| CHE | 2020 | DTU_2020_TWIW_01_CHE_LUZ_042 | Swab | ERR10432513 |
| CHE | 2020 | DTU_2020_TWIW_01_CHE_LUZ_043 | Wound Pus Biopsy | ERR10432546 |
| CHE | 2020 | DTU_2020_TWIW_01_CHE_LUZ_044 | Swab | ERR10432551 |
| CHE | 2020 | DTU_2020_TWIW_01_CHE_LUZ_045 | Wound Pus Biopsy | ERR10432526 |
| CHE | 2020 | DTU_2020_TWIW_01_CHE_LUZ_046 | Urine | ERR10432475 |
| CHE | 2020 | DTU_2020_TWIW_01_CHE_LUZ_047 | Urine | ERR10432555 |
| CHE | 2020 | DTU_2020_TWIW_01_CHE_LUZ_048 | Wound Pus Biopsy | ERR10432473 |
| CHE | 2020 | DTU_2020_TWIW_01_CHE_LUZ_049 | Wound Pus Biopsy | ERR10432553 |
| CHE | 2020 | DTU_2020_TWIW_01_CHE_LUZ_050 | Swab | ERR10432543 |
| CHE | 2020 | DTU_2020_TWIW_01_CHE_LUZ_052 | Urine | ERR10432547 |
| CHE | 2020 | DTU_2020_TWIW_01_CHE_LUZ_053 | Urine | ERR10432469 |
| CHE | 2020 | DTU_2020_TWIW_01_CHE_LUZ_054 | Urine | ERR10432471 |
| CHE | 2020 | DTU_2020_TWIW_01_CHE_LUZ_055 | Urine | ERR10432550 |
| CHE | 2020 | DTU_2020_TWIW_01_CHE_LUZ_056 | Urine | ERR10432454 |
| CHE | 2020 | DTU_2020_TWIW_01_CHE_LUZ_057 | Urine | ERR10432472 |
| CHE | 2020 | DTU_2020_TWIW_01_CHE_LUZ_058 | Swab | ERR10432548 |
| CHE | 2020 | DTU_2020_TWIW_01_CHE_LUZ_059 | Urine | ERR10432557 |
| CHE | 2020 | DTU_2020_TWIW_01_CHE_LUZ_060 | Swab | ERR10432541 |
| PAK | 2020 | DTU_2020_TWIW_01_PAK_PES_002 | Urine | ERR10431519 |
| PAK | 2020 | DTU_2020_TWIW_01_PAK_PES_004 | Urine | ERR10431475 |
| PAK | 2020 | DTU_2020_TWIW_01_PAK_PES_005 | Wound Pus Biopsy | ERR10431540 |
| PAK | 2020 | DTU_2020_TWIW_01_PAK_PES_006 | Urine | ERR10431505 |
| PAK | 2020 | DTU_2020_TWIW_01_PAK_PES_007 | Respiratory System | ERR10431457 |
| PAK | 2020 | DTU_2020_TWIW_01_PAK_PES_008 | Urine | ERR10431480 |
| PAK | 2020 | DTU_2020_TWIW_01_PAK_PES_009 | Wound Pus Biopsy | ERR10431563 |
| PAK | 2020 | DTU_2020_TWIW_01_PAK_PES_010 | Wound Pus Biopsy | ERR10431482 |
| PAK | 2020 | DTU_2020_TWIW_01_PAK_PES_011 | Urine | ERR10431488 |
| PAK | 2020 | DTU_2020_TWIW_01_PAK_PES_012 | Urine | ERR10431491 |
| PAK | 2020 | DTU_2020_TWIW_01_PAK_PES_013 | Urine | ERR10431500 |
| PAK | 2020 | DTU_2020_TWIW_01_PAK_PES_014 | Urine | ERR10431533 |
| PAK | 2020 | DTU_2020_TWIW_01_PAK_PES_015 | Wound Pus Biopsy | ERR10431529 |
| PAK | 2020 | DTU_2020_TWIW_01_PAK_PES_016 | Urine | ERR10431490 |
| PAK | 2020 | DTU_2020_TWIW_01_PAK_PES_017 | Wound Pus Biopsy | ERR10431579 |
| PAK | 2020 | DTU_2020_TWIW_01_PAK_PES_018 | Wound Pus Biopsy | ERR10431499 |
| PAK | 2020 | DTU_2020_TWIW_01_PAK_PES_019 | Wound Pus Biopsy | ERR10431547 |
| PAK | 2020 | DTU_2020_TWIW_01_PAK_PES_020 | Urine | ERR10431544 |
| PAK | 2020 | DTU_2020_TWIW_01_PAK_PES_021 | Wound Pus Biopsy | ERR10431545 |
| PAK | 2020 | DTU_2020_TWIW_01_PAK_PES_022 | Urine | ERR10431514 |
| PAK | 2020 | DTU_2020_TWIW_01_PAK_PES_023 | Wound Pus Biopsy | ERR10431472 |
| PAK | 2020 | DTU_2020_TWIW_01_PAK_PES_024 | Wound Pus Biopsy | ERR10431516 |
| PAK | 2020 | DTU_2020_TWIW_01_PAK_PES_025 | Urine | ERR10431485 |
| PAK | 2020 | DTU_2020_TWIW_01_PAK_PES_026 | Wound Pus Biopsy | ERR10431467 |
| PAK | 2020 | DTU_2020_TWIW_01_PAK_PES_027 | Blood | ERR10431469 |
| PAK | 2020 | DTU_2020_TWIW_01_PAK_PES_031 | Wound Pus Biopsy | ERR10431515 |
| PAK | 2020 | DTU_2020_TWIW_01_PAK_PES_032 | Urine | ERR10431483 |
| PAK | 2020 | DTU_2020_TWIW_01_PAK_PES_033 | Wound Pus Biopsy | ERR10431570 |
| PAK | 2020 | DTU_2020_TWIW_01_PAK_PES_034 | Blood | ERR10431573 |
| PAK | 2020 | DTU_2020_TWIW_01_PAK_PES_035 | Wound Pus Biopsy | ERR10431492 |
| PAK | 2020 | DTU_2020_TWIW_01_PAK_PES_036 | Urine | ERR10431496 |
| PAK | 2020 | DTU_2020_TWIW_01_PAK_PES_037 | Wound Pus Biopsy | ERR10431460 |
| PAK | 2020 | DTU_2020_TWIW_01_PAK_PES_038 | Other | ERR10431495 |
| PAK | 2020 | DTU_2020_TWIW_01_PAK_PES_039 | Wound Pus Biopsy | ERR10431470 |
| PAK | 2020 | DTU_2020_TWIW_01_PAK_PES_040 | Wound Pus Biopsy | ERR10431489 |
| PAK | 2020 | DTU_2020_TWIW_01_PAK_PES_041 | Urine | ERR10431530 |
| PAK | 2020 | DTU_2020_TWIW_01_PAK_PES_042 | Urine | ERR10431526 |
| PAK | 2020 | DTU_2020_TWIW_01_PAK_PES_043 | Urine | ERR10431494 |
| PAK | 2020 | DTU_2020_TWIW_01_PAK_PES_047 | Blood | ERR10431577 |
| PAK | 2020 | DTU_2020_TWIW_01_PAK_PES_048 | Urine | ERR10431576 |
| PAK | 2020 | DTU_2020_TWIW_01_PAK_PES_049 | Blood | ERR10431561 |
| PAK | 2020 | DTU_2020_TWIW_01_PAK_PES_050 | Blood | ERR10431493 |
| PAK | 2020 | DTU_2020_TWIW_01_PAK_PES_051 | Urine | ERR10431486 |
| PAK | 2020 | DTU_2020_TWIW_01_PAK_PES_052 | Other | ERR10431539 |
| PAK | 2020 | DTU_2020_TWIW_01_PAK_PES_053 | Other | ERR10431465 |
| PAK | 2020 | DTU_2020_TWIW_01_PAK_PES_054 | Other | ERR10431553 |
| PAK | 2020 | DTU_2020_TWIW_01_PAK_PES_055 | Other | ERR10431543 |
| PAK | 2020 | DTU_2020_TWIW_01_PAK_PES_056 | Urine | NA |
| PAK | 2020 | DTU_2020_TWIW_01_PAK_PES_057 | Urine | ERR10431502 |
| PAK | 2020 | DTU_2020_TWIW_01_PAK_PES_058 | Urine | NA |
| PAK | 2020 | DTU_2020_TWIW_01_PAK_PES_059 | Urine | ERR10431504 |
| PAK | 2020 | DTU_2020_TWIW_01_PAK_PES_060 | Other | ERR10431479 |
| PAK | 2020 | DTU_2020_TWIW_01_PAK_PES_061 | Wound Pus Biopsy | ERR10439758 |
| PAK | 2020 | DTU_2020_TWIW_01_PAK_PES_061 | Wound Pus Biopsy | ERR14129346 |
| PAK | 2020 | DTU_2020_TWIW_01_PAK_PES_062 | Wound Pus Biopsy | ERR10431464 |
| PAK | 2020 | DTU_2020_TWIW_01_PAK_PES_063 | Urine | ERR10431528 |
| PAK | 2020 | DTU_2020_TWIW_01_PAK_PES_064 | Other | ERR10431555 |
| PAK | 2020 | DTU_2020_TWIW_01_PAK_PES_065 | Wound Pus Biopsy | ERR10431506 |
| PAK | 2020 | DTU_2020_TWIW_01_PAK_PES_066 | Wound Pus Biopsy | ERR10431535 |
| PAK | 2020 | DTU_2020_TWIW_01_PAK_PES_067 | Wound Pus Biopsy | ERR10431550 |
| CHE | 2020 | DTU_2020_TWIW_01_CHE_BAS_001 | Urine | ERR10432460 |
| CHE | 2020 | DTU_2020_TWIW_01_CHE_BAS_002 | Urine | ERR10432495 |
| CHE | 2020 | DTU_2020_TWIW_01_CHE_BAS_003 | Urine | ERR10432479 |
| CHE | 2020 | DTU_2020_TWIW_01_CHE_BAS_004 | Urine | ERR10432493 |
| CHE | 2020 | DTU_2020_TWIW_01_CHE_BAS_006 | Urine | ERR10432480 |
| CHE | 2020 | DTU_2020_TWIW_01_CHE_BAS_007 | Urine | ERR10432492 |
| CHE | 2020 | DTU_2020_TWIW_01_CHE_BAS_008 | Wound Pus Biopsy | ERR10432542 |
| CHE | 2020 | DTU_2020_TWIW_01_CHE_BAS_009 | Swab | ERR10432518 |
| CHE | 2020 | DTU_2020_TWIW_01_CHE_BAS_010 | Swab | ERR10432549 |
| CHE | 2020 | DTU_2020_TWIW_01_CHE_BAS_011 | Swab | ERR10432476 |
| CHE | 2020 | DTU_2020_TWIW_01_CHE_BAS_012 | Wound Pus Biopsy | ERR10432521 |
| CHE | 2020 | DTU_2020_TWIW_01_CHE_BAS_013 | Blood | ERR10432459 |
| CHE | 2020 | DTU_2020_TWIW_01_CHE_BAS_014 | Blood | ERR10432527 |
| CHE | 2020 | DTU_2020_TWIW_01_CHE_BAS_015 | Blood | ERR10432498 |
| CHE | 2020 | DTU_2020_TWIW_01_CHE_BAS_016 | Respiratory System | ERR10432519 |
| CHE | 2020 | DTU_2020_TWIW_01_CHE_BAS_017 | Swab | ERR10432483 |
| CHE | 2020 | DTU_2020_TWIW_01_CHE_BAS_018 | Urine | ERR10432477 |
| CHE | 2020 | DTU_2020_TWIW_01_CHE_BAS_019 | Other | ERR10432568 |
| CHE | 2020 | DTU_2020_TWIW_01_CHE_BAS_020 | Urine | ERR10432594 |
| CHE | 2020 | DTU_2020_TWIW_01_CHE_BAS_021 | Urine | ERR10432491 |
| CHE | 2020 | DTU_2020_TWIW_01_CHE_BAS_022 | Urine | ERR10432576 |
| CHE | 2020 | DTU_2020_TWIW_01_CHE_BAS_023 | Urine | ERR10432484 |
| CHE | 2020 | DTU_2020_TWIW_01_CHE_BAS_024 | Wound Pus Biopsy | ERR10432520 |
| CHE | 2020 | DTU_2020_TWIW_01_CHE_BAS_026 | Wound Pus Biopsy | ERR10432573 |
| CHE | 2020 | DTU_2020_TWIW_01_CHE_BAS_027 | Blood | ERR10432504 |
| CHE | 2020 | DTU_2020_TWIW_01_CHE_BAS_028 | Blood | ERR10432503 |
| CHE | 2020 | DTU_2020_TWIW_01_CHE_BAS_029 | Respiratory System | ERR10432529 |
| CHE | 2020 | DTU_2020_TWIW_01_CHE_BAS_030 | Urine | ERR10432511 |
| CHE | 2020 | DTU_2020_TWIW_01_CHE_BAS_031 | Urine | ERR10432482 |
| CHE | 2020 | DTU_2020_TWIW_01_CHE_BAS_032 | Urine | ERR10432456 |
| CHE | 2020 | DTU_2020_TWIW_01_CHE_BAS_033 | Swab | ERR10432558 |
| CHE | 2020 | DTU_2020_TWIW_01_CHE_BAS_035 | Blood | ERR10432528 |
| CHE | 2020 | DTU_2020_TWIW_01_CHE_BAS_036 | Blood | ERR10432539 |
| CHE | 2020 | DTU_2020_TWIW_01_CHE_BAS_037 | Wound Pus Biopsy | ERR10432571 |
| CHE | 2020 | DTU_2020_TWIW_01_CHE_BAS_038 | Wound Pus Biopsy | ERR10432537 |
| CHE | 2020 | DTU_2020_TWIW_01_CHE_BAS_039 | Wound Pus Biopsy | ERR10432525 |
| CHE | 2020 | DTU_2020_TWIW_01_CHE_BAS_041 | Respiratory System | ERR10432499 |
| CHE | 2020 | DTU_2020_TWIW_01_CHE_BAS_042 | Swab | ERR10432481 |
| CHE | 2020 | DTU_2020_TWIW_01_CHE_BAS_043 | Urine | ERR10432485 |
| CHE | 2020 | DTU_2020_TWIW_01_CHE_BAS_044 | Wound Pus Biopsy | ERR10432514 |
| CHE | 2020 | DTU_2020_TWIW_01_CHE_BAS_045 | Swab | ERR10432524 |
| CHE | 2020 | DTU_2020_TWIW_01_CHE_BAS_046 | Wound Pus Biopsy | ERR10432488 |
| CHE | 2020 | DTU_2020_TWIW_01_CHE_BAS_047 | Swab | ERR10432517 |
| CHE | 2020 | DTU_2020_TWIW_01_CHE_BAS_048 | Blood | ERR10432500 |
| CHE | 2020 | DTU_2020_TWIW_01_CHE_BAS_049 | Blood | ERR10432538 |
| CHE | 2020 | DTU_2020_TWIW_01_CHE_BAS_050 | Swab | ERR10432580 |
| CHE | 2020 | DTU_2020_TWIW_01_CHE_BAS_051 | Wound Pus Biopsy | ERR10432545 |
| CHE | 2020 | DTU_2020_TWIW_01_CHE_BAS_052 | Wound Pus Biopsy | ERR10432552 |
| CHE | 2020 | DTU_2020_TWIW_01_CHE_BAS_053 | Wound Pus Biopsy | ERR10432487 |
| CHE | 2020 | DTU_2020_TWIW_01_CHE_BAS_054 | Wound Pus Biopsy | ERR10432554 |
| CHE | 2020 | DTU_2020_TWIW_01_CHE_BAS_055 | Wound Pus Biopsy | ERR10432522 |
| CHE | 2020 | DTU_2020_TWIW_01_CHE_BAS_056 | Swab | ERR10432535 |
| CHE | 2020 | DTU_2020_TWIW_01_CHE_BAS_057 | Wound Pus Biopsy | ERR10432494 |
| CHE | 2020 | DTU_2020_TWIW_01_CHE_BAS_058 | Respiratory System | ERR10432465 |
| CHE | 2020 | DTU_2020_TWIW_01_CHE_BAS_060 | Wound Pus Biopsy | ERR10432512 |
| CHE | 2020 | DTU_2020_TWIW_01_CHE_BAS_061 | Blood | ERR10439815 |
| CHE | 2020 | DTU_2020_TWIW_01_CHE_BAS_062 | Blood | ERR10432575 |
| CHE | 2020 | DTU_2020_TWIW_01_CHE_BAS_063 | Blood | ERR10432531 |
| CHE | 2020 | DTU_2020_TWIW_01_CHE_BAS_064 | Blood | ERR10432502 |
| CHE | 2020 | DTU_2020_TWIW_01_CHE_BAS_065 | Blood | ERR10432489 |
| CHE | 2020 | DTU_2020_TWIW_01_CHE_BAS_066 | Swab | ERR10432508 |
| CHE | 2020 | DTU_2020_TWIW_01_CHE_BAS_067 | Wound Pus Biopsy | ERR10432556 |
| CHE | 2020 | DTU_2020_TWIW_01_CHE_BAS_068 | Respiratory System | ERR10432533 |
| CHE | 2020 | DTU_2020_TWIW_01_CHE_BAS_069 | Swab | ERR10432566 |
| CHE | 2020 | DTU_2020_TWIW_01_CHE_BAS_070 | Respiratory System | ERR10432532 |
| AUS | 2020 | DTU_2020_TWIW_01_AUS_CAN_001 | Urine | ERR10446193 |
| AUS | 2020 | DTU_2020_TWIW_01_AUS_CAN_002 | Urine | ERR10430796 |
| AUS | 2020 | DTU_2020_TWIW_01_AUS_CAN_003 | Urine | ERR10430804 |
| AUS | 2020 | DTU_2020_TWIW_01_AUS_CAN_004 | Urine | ERR10430847 |
| AUS | 2020 | DTU_2020_TWIW_01_AUS_CAN_005 | Swab | ERR10431368 |
| AUS | 2020 | DTU_2020_TWIW_01_AUS_CAN_006 | Urine | ERR10430846 |
| AUS | 2020 | DTU_2020_TWIW_01_AUS_CAN_007 | Urine | ERR10431371 |
| AUS | 2020 | DTU_2020_TWIW_01_AUS_CAN_008 | Urine | ERR10430858 |
| AUS | 2020 | DTU_2020_TWIW_01_AUS_CAN_009 | Urine | ERR10430852 |
| AUS | 2020 | DTU_2020_TWIW_01_AUS_CAN_010 | Urine | ERR10430856 |
| AUS | 2020 | DTU_2020_TWIW_01_AUS_CAN_011 | Urine | ERR10430845 |
| AUS | 2020 | DTU_2020_TWIW_01_AUS_CAN_012 | Swab | ERR10431358 |
| AUS | 2020 | DTU_2020_TWIW_01_AUS_CAN_013 | Respiratory System | ERR10430721 |
| AUS | 2020 | DTU_2020_TWIW_01_AUS_CAN_014 | Urine | ERR10430859 |
| AUS | 2020 | DTU_2020_TWIW_01_AUS_CAN_015 | Urine | ERR10430778 |
| AUS | 2020 | DTU_2020_TWIW_01_AUS_CAN_016 | Blood | ERR10431342 |
| AUS | 2020 | DTU_2020_TWIW_01_AUS_CAN_017 | Swab | ERR10431332 |
| AUS | 2020 | DTU_2020_TWIW_01_AUS_CAN_018 | Urine | ERR10430850 |
| AUS | 2020 | DTU_2020_TWIW_01_AUS_CAN_019 | Other | ERR10431351 |
| AUS | 2020 | DTU_2020_TWIW_01_AUS_CAN_020 | Urine | ERR10430854 |
| AUS | 2020 | DTU_2020_TWIW_01_AUS_CAN_021 | Urine | ERR10430863 |
| AUS | 2020 | DTU_2020_TWIW_01_AUS_CAN_022 | Wound Pus Biopsy | ERR10431350 |
| AUS | 2020 | DTU_2020_TWIW_01_AUS_CAN_023 | Swab | ERR10431366 |
| AUS | 2020 | DTU_2020_TWIW_01_AUS_CAN_024 | Urine | ERR10431320 |
| AUS | 2020 | DTU_2020_TWIW_01_AUS_CAN_025 | Urine | ERR10430870 |
| AUS | 2020 | DTU_2020_TWIW_01_AUS_CAN_026 | Urine | ERR10430865 |
| AUS | 2020 | DTU_2020_TWIW_01_AUS_CAN_027 | Urine | ERR10430864 |
| AUS | 2020 | DTU_2020_TWIW_01_AUS_CAN_028 | Urine | ERR10430306 |
| AUS | 2020 | DTU_2020_TWIW_01_AUS_CAN_029 | Urine | ERR10430728 |
| AUS | 2020 | DTU_2020_TWIW_01_AUS_CAN_030 | Urine | ERR10430848 |
| AUS | 2020 | DTU_2020_TWIW_01_AUS_CAN_031 | Other | ERR10431324 |
| AUS | 2020 | DTU_2020_TWIW_01_AUS_CAN_032 | Urine | ERR10431304 |
| AUS | 2020 | DTU_2020_TWIW_01_AUS_CAN_033 | Swab | ERR10431354 |
| AUS | 2020 | DTU_2020_TWIW_01_AUS_CAN_034 | Wound Pus Biopsy | ERR10431334 |
| AUS | 2020 | DTU_2020_TWIW_01_AUS_CAN_036 | Blood | ERR10431337 |
| AUS | 2020 | DTU_2020_TWIW_01_AUS_CAN_037 | Urine | ERR10431316 |
| AUS | 2020 | DTU_2020_TWIW_01_AUS_CAN_038 | Wound Pus Biopsy | ERR10431327 |
| AUS | 2020 | DTU_2020_TWIW_01_AUS_CAN_039 | Urine | ERR10430853 |
| AUS | 2020 | DTU_2020_TWIW_01_AUS_CAN_040 | Other | ERR10430855 |
| AUS | 2020 | DTU_2020_TWIW_01_AUS_CAN_041 | Urine | ERR10430860 |
| AUS | 2020 | DTU_2020_TWIW_01_AUS_CAN_042 | Other | ERR10431329 |
| AUS | 2020 | DTU_2020_TWIW_01_AUS_CAN_043 | Urine | ERR10430857 |
| AUS | 2020 | DTU_2020_TWIW_01_AUS_CAN_044 | Other | ERR10431336 |
| AUS | 2020 | DTU_2020_TWIW_01_AUS_CAN_045 | Wound Pus Biopsy | ERR10430862 |
| AUS | 2020 | DTU_2020_TWIW_01_AUS_CAN_047 | Urine | ERR10430861 |
| AUS | 2020 | DTU_2020_TWIW_01_AUS_CAN_048 | Urine | ERR10430867 |
| AUS | 2020 | DTU_2020_TWIW_01_AUS_CAN_049 | Wound Pus Biopsy | ERR10431330 |
| AUS | 2020 | DTU_2020_TWIW_01_AUS_CAN_050 | Urine | ERR10430868 |
| AUS | 2020 | DTU_2020_TWIW_01_AUS_CAN_051 | Swab | ERR10431341 |
| AUS | 2020 | DTU_2020_TWIW_01_AUS_CAN_052 | Urine | ERR10430866 |
| AUS | 2020 | DTU_2020_TWIW_01_AUS_CAN_054 | Urine | ERR10430869 |
| AUS | 2020 | DTU_2020_TWIW_01_AUS_CAN_055 | Urine | ERR10430876 |
| AUS | 2020 | DTU_2020_TWIW_01_AUS_CAN_056 | Urine | ERR10430878 |
| AUS | 2020 | DTU_2020_TWIW_01_AUS_CAN_057 | Urine | ERR10430877 |
| AUS | 2020 | DTU_2020_TWIW_01_AUS_CAN_058 | Urine | ERR10431306 |
| AUS | 2020 | DTU_2020_TWIW_01_AUS_CAN_059 | Blood | ERR10431313 |
| AUS | 2020 | DTU_2020_TWIW_01_AUS_CAN_060 | Other | ERR10431331 |
| PAK | 2020 | DTU_2020_TWIW_01_PAK_PES_020A | Urine | ERR10431484 |
| PAK | 2020 | DTU_2020_TWIW_01_PAK_PES_022A | Urine | ERR10431481 |
| PAK | 2020 | DTU_2020_TWIW_01_PAK_PES_028A | Other | ERR10431487 |
| PAK | 2020 | DTU_2020_TWIW_01_PAK_PES_036A | Urine | ERR10431503 |
| PAK | 2020 | DTU_2020_TWIW_01_PAK_PES_039A | Wound Pus Biopsy | ERR10431474 |
| PAK | 2020 | DTU_2020_TWIW_01_PAK_PES_056A | Urine | NA |
| PAK | 2020 | DTU_2020_TWIW_01_PAK_PES_056B | Urine | NA |
| BEN | 2020 | DTU_2020_TWIW_01_BEN_COT_003 | Urine | ERR10436300 |
| BEN | 2020 | DTU_2020_TWIW_01_BEN_COT_004 | Urine | ERR10436305 |
| BEN | 2020 | DTU_2020_TWIW_01_BEN_COT_007 | Other | ERR10436190 |
| BEN | 2020 | DTU_2020_TWIW_01_BEN_COT_011 | Other | ERR10436186 |
| BEN | 2020 | DTU_2020_TWIW_01_BEN_COT_012 | Other | ERR10436316 |
| BEN | 2020 | DTU_2020_TWIW_01_BEN_COT_014 | Urine | ERR10436202 |
| BEN | 2020 | DTU_2020_TWIW_01_BEN_COT_015 | Urine | ERR10436350 |
| BEN | 2020 | DTU_2020_TWIW_01_BEN_COT_016 | Urine | ERR10436220 |
| BEN | 2020 | DTU_2020_TWIW_01_BEN_COT_020 | Urine | ERR10436230 |
| BEN | 2020 | DTU_2020_TWIW_01_BEN_COT_021 | Urine | ERR10436238 |
| BEN | 2020 | DTU_2020_TWIW_01_BEN_COT_023 | Urine | ERR10436323 |
| BEN | 2020 | DTU_2020_TWIW_01_BEN_COT_024 | Urine | ERR10436287 |
| BEN | 2020 | DTU_2020_TWIW_01_BEN_COT_025 | Urine | ERR10436242 |
| BEN | 2020 | DTU_2020_TWIW_01_BEN_COT_026 | Urine | ERR10436172 |
| BEN | 2020 | DTU_2020_TWIW_01_BEN_COT_027 | Urine | ERR10436245 |
| BEN | 2020 | DTU_2020_TWIW_01_BEN_COT_031 | Urine | ERR10436237 |
| BEN | 2020 | DTU_2020_TWIW_01_BEN_COT_032 | Urine | ERR10436345 |
| BEN | 2020 | DTU_2020_TWIW_01_BEN_COT_038 | Urine | ERR10436175 |
| BEN | 2020 | DTU_2020_TWIW_01_BEN_COT_040 | Urine | ERR10436344 |
| BEN | 2020 | DTU_2020_TWIW_01_BEN_COT_043 | Urine | ERR10436353 |
| BEN | 2020 | DTU_2020_TWIW_01_BEN_COT_045 | Urine | ERR10436355 |
| BEN | 2020 | DTU_2020_TWIW_01_BEN_COT_048 | Urine | ERR10436276 |
| BEN | 2020 | DTU_2020_TWIW_01_BEN_COT_049 | Urine | ERR10436292 |
| BEN | 2020 | DTU_2020_TWIW_01_BEN_COT_052 | Urine | ERR10436329 |
| BEN | 2020 | DTU_2020_TWIW_01_BEN_COT_053 | Urine | ERR10436352 |
| BEN | 2020 | DTU_2020_TWIW_01_BEN_COT_054 | Urine | ERR10436346 |
| BEN | 2020 | DTU_2020_TWIW_01_BEN_COT_055 | Urine | ERR10436310 |
| BEN | 2020 | DTU_2020_TWIW_01_BEN_COT_056 | Urine | ERR10436347 |
| BEN | 2020 | DTU_2020_TWIW_01_BEN_COT_061 | Urine | ERR10436250 |
| BEN | 2020 | DTU_2020_TWIW_01_BEN_COT_062 | Urine | ERR10436349 |
| BEN | 2020 | DTU_2020_TWIW_01_BEN_COT_063 | Urine | ERR10436348 |
| BEN | 2020 | DTU_2020_TWIW_01_BEN_COT_066 | Urine | ERR10436306 |
| BEN | 2020 | DTU_2020_TWIW_01_BEN_COT_067 | Urine | ERR10436351 |
| BEN | 2020 | DTU_2020_TWIW_01_BEN_COT_070 | Urine | ERR10436299 |
| PAK | 2020 | DTU_2020_TWIW_01_PAK_RAW_001 | Blood | ERR10431560 |
| PAK | 2020 | DTU_2020_TWIW_01_PAK_RAW_002 | Blood | ERR10431558 |
| PAK | 2020 | DTU_2020_TWIW_01_PAK_RAW_003 | Urine | ERR10431564 |
| PAK | 2020 | DTU_2020_TWIW_01_PAK_RAW_004 | Urine | ERR10431455 |
| PAK | 2020 | DTU_2020_TWIW_01_PAK_RAW_005 | Wound Pus Biopsy | ERR10431568 |
| PAK | 2020 | DTU_2020_TWIW_01_PAK_RAW_006 | Wound Pus Biopsy | ERR10431468 |
| PAK | 2020 | DTU_2020_TWIW_01_PAK_RAW_008 | Swab | ERR10431510 |
| PAK | 2020 | DTU_2020_TWIW_01_PAK_RAW_009 | Urine | ERR10431507 |
| PAK | 2020 | DTU_2020_TWIW_01_PAK_RAW_009 | Urine | ERR14129347 |
| PAK | 2020 | DTU_2020_TWIW_01_PAK_RAW_010 | Urine | ERR10431525 |
| PAK | 2020 | DTU_2020_TWIW_01_PAK_RAW_011 | Urine | ERR10431509 |
| PAK | 2020 | DTU_2020_TWIW_01_PAK_RAW_013 | Urine | ERR10431527 |
| PAK | 2020 | DTU_2020_TWIW_01_PAK_RAW_014 | Wound Pus Biopsy | ERR10431565 |
| PAK | 2020 | DTU_2020_TWIW_01_PAK_RAW_015 | Wound Pus Biopsy | ERR10431567 |
| PAK | 2020 | DTU_2020_TWIW_01_PAK_RAW_016 | Wound Pus Biopsy | ERR10431548 |
| PAK | 2020 | DTU_2020_TWIW_01_PAK_RAW_017 | Other | ERR10431551 |
| PAK | 2020 | DTU_2020_TWIW_01_PAK_RAW_018 | Urine | ERR10431524 |
| PAK | 2020 | DTU_2020_TWIW_01_PAK_RAW_019 | Urine | ERR10431501 |
| PAK | 2020 | DTU_2020_TWIW_01_PAK_RAW_020 | Urine | ERR10431463 |
| PAK | 2020 | DTU_2020_TWIW_01_PAK_RAW_021 | Respiratory System | ERR10431471 |
| PAK | 2020 | DTU_2020_TWIW_01_PAK_RAW_022 | Wound Pus Biopsy | ERR10431557 |
| PAK | 2020 | DTU_2020_TWIW_01_PAK_RAW_023 | Wound Pus Biopsy | ERR10431536 |
| PAK | 2020 | DTU_2020_TWIW_01_PAK_RAW_024 | Urine | ERR10431508 |
| PAK | 2020 | DTU_2020_TWIW_01_PAK_RAW_025 | Urine | ERR10431554 |
| PAK | 2020 | DTU_2020_TWIW_01_PAK_RAW_026 | Urine | ERR10431556 |
| PAK | 2020 | DTU_2020_TWIW_01_PAK_RAW_027 | Urine | ERR10431522 |
| PAK | 2020 | DTU_2020_TWIW_01_PAK_RAW_028 | Urine | ERR10431511 |
| PAK | 2020 | DTU_2020_TWIW_01_PAK_RAW_029 | Urine | ERR10431512 |
| PAK | 2020 | DTU_2020_TWIW_01_PAK_RAW_030 | Urine | ERR10431517 |
| PAK | 2020 | DTU_2020_TWIW_01_PAK_RAW_032 | Urine | ERR10431534 |
| PAK | 2020 | DTU_2020_TWIW_01_PAK_RAW_033 | Wound Pus Biopsy | ERR10431461 |
| PAK | 2020 | DTU_2020_TWIW_01_PAK_RAW_034 | Respiratory System | ERR10431459 |
| PAK | 2020 | DTU_2020_TWIW_01_PAK_RAW_036 | Wound Pus Biopsy | ERR10431473 |
| PAK | 2020 | DTU_2020_TWIW_01_PAK_RAW_037 | Wound Pus Biopsy | ERR10431571 |
| PAK | 2020 | DTU_2020_TWIW_01_PAK_RAW_038 | Wound Pus Biopsy | ERR10431538 |
| PAK | 2020 | DTU_2020_TWIW_01_PAK_RAW_039 | Wound Pus Biopsy | ERR10431532 |
| PAK | 2020 | DTU_2020_TWIW_01_PAK_RAW_040 | Respiratory System | ERR10431537 |
| PAK | 2020 | DTU_2020_TWIW_01_PAK_RAW_041 | Urine | ERR10431575 |
| PAK | 2020 | DTU_2020_TWIW_01_PAK_RAW_043 | Respiratory System | ERR10431520 |
| PAK | 2020 | DTU_2020_TWIW_01_PAK_RAW_045 | Wound Pus Biopsy | ERR10431569 |
| PAK | 2020 | DTU_2020_TWIW_01_PAK_RAW_046 | Wound Pus Biopsy | ERR10431559 |
| PAK | 2020 | DTU_2020_TWIW_01_PAK_RAW_047 | Wound Pus Biopsy | ERR10431572 |
| PAK | 2020 | DTU_2020_TWIW_01_PAK_RAW_048 | Urine | ERR10431523 |
| PAK | 2020 | DTU_2020_TWIW_01_PAK_RAW_049 | Wound Pus Biopsy | ERR10431476 |
| PAK | 2020 | DTU_2020_TWIW_01_PAK_RAW_050 | Urine | ERR10431513 |
| PAK | 2020 | DTU_2020_TWIW_01_PAK_RAW_007A | Urine | ERR10431498 |
| PAK | 2020 | DTU_2020_TWIW_01_PAK_RAW_041A | Urine | ERR10431566 |
| PAK | 2020 | DTU_2020_TWIW_01_PAK_RAW_042A | Wound Pus Biopsy | ERR10431531 |
| PAK | 2020 | DTU_2020_TWIW_01_PAK_RAW_042A | Wound Pus Biopsy | ERR14129390 |
| PAK | 2020 | DTU_2020_TWIW_01_PAK_RAW_044A | Wound Pus Biopsy | ERR10431518 |
| PAK | 2020 | DTU_2020_TWIW_01_PAK_RAW_044A | Wound Pus Biopsy | ERR14129391 |
| BGR | 2020 | DTU_2020_TWIW_01_BGR_SOF_005 | Respiratory System | ERR10436357 |
| BGR | 2020 | DTU_2020_TWIW_01_BGR_SOF_007 | Respiratory System | ERR10441136 |
| BGR | 2020 | DTU_2020_TWIW_01_BGR_SOF_011 | Blood | ERR10441115 |
| BGR | 2020 | DTU_2020_TWIW_01_BGR_SOF_012 | Swab | ERR10441137 |
| BGR | 2020 | DTU_2020_TWIW_01_BGR_SOF_015 | Respiratory System | ERR10436356 |
| BGR | 2020 | DTU_2020_TWIW_01_BGR_SOF_016 | Respiratory System | ERR10436362 |
| BGR | 2020 | DTU_2020_TWIW_01_BGR_SOF_017 | Respiratory System | ERR10441126 |
| BGR | 2020 | DTU_2020_TWIW_01_BGR_SOF_019 | Wound Pus Biopsy | ERR10436372 |
| BGR | 2020 | DTU_2020_TWIW_01_BGR_SOF_020 | Wound Pus Biopsy | ERR10436364 |
| BGR | 2020 | DTU_2020_TWIW_01_BGR_SOF_020 | Wound Pus Biopsy | ERR14150401 |
| BGR | 2020 | DTU_2020_TWIW_01_BGR_SOF_022 | Respiratory System | ERR10436358 |
| BGR | 2020 | DTU_2020_TWIW_01_BGR_SOF_023 | Urine | ERR10436359 |
| BGR | 2020 | DTU_2020_TWIW_01_BGR_SOF_024 | Swab | ERR10436378 |
| BGR | 2020 | DTU_2020_TWIW_01_BGR_SOF_025 | Urine | ERR10441144 |
| BGR | 2020 | DTU_2020_TWIW_01_BGR_SOF_026 | Urine | ERR10436361 |
| BGR | 2020 | DTU_2020_TWIW_01_BGR_SOF_027 | Swab | ERR10436370 |
| BGR | 2020 | DTU_2020_TWIW_01_BGR_SOF_029 | Swab | ERR10441065 |
| BGR | 2020 | DTU_2020_TWIW_01_BGR_SOF_030 | Urine | ERR10436365 |
| BGR | 2020 | DTU_2020_TWIW_01_BGR_SOF_033 | Swab | ERR10441106 |
| BGR | 2020 | DTU_2020_TWIW_01_BGR_SOF_035 | Other | ERR10436366 |
| BGR | 2020 | DTU_2020_TWIW_01_BGR_SOF_036 | Swab | ERR10441105 |
| BGR | 2020 | DTU_2020_TWIW_01_BGR_SOF_038 | Respiratory System | ERR10441122 |
| BGR | 2020 | DTU_2020_TWIW_01_BGR_SOF_040 | Urine | ERR10436368 |
| BGR | 2020 | DTU_2020_TWIW_01_BGR_SOF_044 | Urine | ERR10436367 |
| BGR | 2020 | DTU_2020_TWIW_01_BGR_SOF_047 | Other | ERR10441109 |
| BGR | 2020 | DTU_2020_TWIW_01_BGR_SOF_048 | Blood | ERR10441119 |
| BGR | 2020 | DTU_2020_TWIW_01_BGR_SOF_050 | Wound Pus Biopsy | ERR10441098 |
| BGR | 2020 | DTU_2020_TWIW_01_BGR_SOF_056 | Wound Pus Biopsy | ERR10436375 |
| BGR | 2020 | DTU_2020_TWIW_01_BGR_SOF_058 | Urine | ERR10441064 |
| BGR | 2020 | DTU_2020_TWIW_01_BGR_SOF_060 | Wound Pus Biopsy | ERR10436373 |
| CAN | 2020 | DTU_2020_TWIW_01_CAN_SHE_001 | Swab | ERR10441334 |
| CAN | 2020 | DTU_2020_TWIW_01_CAN_SHE_002 | Swab | ERR10441348 |
| CAN | 2020 | DTU_2020_TWIW_01_CAN_SHE_003 | Wound Pus Biopsy | ERR10441346 |
| CAN | 2020 | DTU_2020_TWIW_01_CAN_SHE_004 | Wound Pus Biopsy | ERR10441349 |
| CAN | 2020 | DTU_2020_TWIW_01_CAN_SHE_005 | Wound Pus Biopsy | ERR10441347 |
| CAN | 2020 | DTU_2020_TWIW_01_CAN_SHE_006 | Other | ERR10441350 |
| CAN | 2020 | DTU_2020_TWIW_01_CAN_SHE_007 | Wound Pus Biopsy | ERR10441351 |
| CAN | 2020 | DTU_2020_TWIW_01_CAN_SHE_008 | Swab | ERR10441345 |
| CAN | 2020 | DTU_2020_TWIW_01_CAN_SHE_009 | Respiratory System | ERR10441333 |
| CAN | 2020 | DTU_2020_TWIW_01_CAN_SHE_010 | Other | ERR10441330 |
| CAN | 2020 | DTU_2020_TWIW_01_CAN_SHE_014 | Other | ERR10441354 |
| CAN | 2020 | DTU_2020_TWIW_01_CAN_SHE_015 | Wound Pus Biopsy | ERR10441398 |
| CAN | 2020 | DTU_2020_TWIW_01_CAN_SHE_016 | Wound Pus Biopsy | ERR10441385 |
| CAN | 2020 | DTU_2020_TWIW_01_CAN_SHE_018 | Wound Pus Biopsy | ERR10441357 |
| CAN | 2020 | DTU_2020_TWIW_01_CAN_SHE_019 | Wound Pus Biopsy | ERR10441371 |
| CAN | 2020 | DTU_2020_TWIW_01_CAN_SHE_025 | Blood | ERR10441362 |
| CAN | 2020 | DTU_2020_TWIW_01_CAN_SHE_029 | Wound Pus Biopsy | ERR10441381 |
| CAN | 2020 | DTU_2020_TWIW_01_CAN_SHE_030 | Swab | ERR10441382 |
| CAN | 2020 | DTU_2020_TWIW_01_CAN_SHE_049 | Urine | ERR10441336 |
| CAN | 2020 | DTU_2020_TWIW_01_CAN_SHE_054 | Urine | ERR10441364 |
| NGA | 2020 | DTU_2020_TWIW_01_NGA_ILE_010 | Urine | ERR10439019 |
| NGA | 2020 | DTU_2020_TWIW_01_NGA_ILE_013 | Blood | ERR10439011 |
| NGA | 2020 | DTU_2020_TWIW_01_NGA_ILE_015 | Urine | ERR10439001 |
| NGA | 2020 | DTU_2020_TWIW_01_NGA_ILE_016 | Urine | ERR10439004 |
| NGA | 2020 | DTU_2020_TWIW_01_NGA_ILE_018 | Blood | ERR10438999 |
| NGA | 2020 | DTU_2020_TWIW_01_NGA_ILE_019 | Blood | ERR10438974 |
| NGA | 2020 | DTU_2020_TWIW_01_NGA_ILE_020 | Blood | ERR10438965 |
| NGA | 2020 | DTU_2020_TWIW_01_NGA_ILE_021 | Blood | ERR10439008 |
| NGA | 2020 | DTU_2020_TWIW_01_NGA_ILE_022 | Blood | ERR10438997 |
| NGA | 2020 | DTU_2020_TWIW_01_NGA_ILE_026 | Blood | ERR10439010 |
| NGA | 2020 | DTU_2020_TWIW_01_NGA_ILE_032 | Urine | ERR10439020 |
| HUN | 2020 | DTU_2020_TWIW_01_HUN_PEC_005 | Respiratory System | ERR10438533 |
| HUN | 2020 | DTU_2020_TWIW_01_HUN_PEC_006 | Wound Pus Biopsy | ERR10438531 |
| HUN | 2020 | DTU_2020_TWIW_01_HUN_PEC_008 | Other | ERR10438562 |
| HUN | 2020 | DTU_2020_TWIW_01_HUN_PEC_018 | Wound Pus Biopsy | ERR10438528 |
| HUN | 2020 | DTU_2020_TWIW_01_HUN_PEC_022 | Urine | ERR10438551 |
| HUN | 2020 | DTU_2020_TWIW_01_HUN_PEC_024 | Urine | ERR10438561 |
| HUN | 2020 | DTU_2020_TWIW_01_HUN_PEC_025 | Urine | ERR10438550 |
| HUN | 2020 | DTU_2020_TWIW_01_HUN_PEC_030 | Wound Pus Biopsy | ERR10438567 |
| HUN | 2020 | DTU_2020_TWIW_01_HUN_PEC_034 | Wound Pus Biopsy | ERR14150440 |
| HUN | 2020 | DTU_2020_TWIW_01_HUN_PEC_034 | Wound Pus Biopsy | ERR10438537 |
| HUN | 2020 | DTU_2020_TWIW_01_HUN_PEC_035 | Urine | ERR10438554 |
| HUN | 2020 | DTU_2020_TWIW_01_HUN_PEC_037 | Wound Pus Biopsy | ERR10438553 |
| HUN | 2020 | DTU_2020_TWIW_01_HUN_PEC_040 | Other | ERR10438564 |
| HUN | 2020 | DTU_2020_TWIW_01_HUN_PEC_043 | Wound Pus Biopsy | ERR10438545 |
| HUN | 2020 | DTU_2020_TWIW_01_HUN_PEC_044 | Wound Pus Biopsy | ERR10438560 |
| HUN | 2020 | DTU_2020_TWIW_01_HUN_PEC_045 | Blood | ERR10438555 |
| HUN | 2020 | DTU_2020_TWIW_01_HUN_PEC_048 | Urine | ERR10438563 |
| HUN | 2020 | DTU_2020_TWIW_01_HUN_PEC_051 | Wound Pus Biopsy | ERR10438548 |
| HUN | 2020 | DTU_2020_TWIW_01_HUN_PEC_053 | Wound Pus Biopsy | ERR10438547 |
| HUN | 2020 | DTU_2020_TWIW_01_HUN_PEC_057 | Wound Pus Biopsy | ERR10438546 |
| HUN | 2020 | DTU_2020_TWIW_01_HUN_PEC_059 | Urine | ERR10438557 |
| CZE | 2020 | DTU_2020_TWIW_01_CZE_PRA_001A | Swab | ERR10436664 |
| CZE | 2020 | DTU_2020_TWIW_01_CZE_PRA_002 | Respiratory System | ERR10436673 |
| CZE | 2020 | DTU_2020_TWIW_01_CZE_PRA_003 | Blood | ERR10436674 |
| CZE | 2020 | DTU_2020_TWIW_01_CZE_PRA_006 | Blood | ERR10436682 |
| CZE | 2020 | DTU_2020_TWIW_01_CZE_PRA_007 | Other | ERR10436683 |
| CZE | 2020 | DTU_2020_TWIW_01_CZE_PRA_011 | Wound Pus Biopsy | ERR10436688 |
| CZE | 2020 | DTU_2020_TWIW_01_CZE_PRA_012 | Wound Pus Biopsy | ERR10436733 |
| CZE | 2020 | DTU_2020_TWIW_01_CZE_PRA_014 | Blood | ERR10436747 |
| CZE | 2020 | DTU_2020_TWIW_01_CZE_PRA_016 | Wound Pus Biopsy | ERR10436692 |
| CZE | 2020 | DTU_2020_TWIW_01_CZE_PRA_017 | Wound Pus Biopsy | ERR10436694 |
| CZE | 2020 | DTU_2020_TWIW_01_CZE_PRA_019 | Wound Pus Biopsy | ERR10436675 |
| CZE | 2020 | DTU_2020_TWIW_01_CZE_PRA_021 | Wound Pus Biopsy | ERR10436696 |
| CZE | 2020 | DTU_2020_TWIW_01_CZE_PRA_022 | Wound Pus Biopsy | ERR10436695 |
| CZE | 2020 | DTU_2020_TWIW_01_CZE_PRA_023 | Wound Pus Biopsy | ERR10436697 |
| CZE | 2020 | DTU_2020_TWIW_01_CZE_PRA_024 | Wound Pus Biopsy | ERR10436704 |
| CZE | 2020 | DTU_2020_TWIW_01_CZE_PRA_025 | Wound Pus Biopsy | ERR10436712 |
| CZE | 2020 | DTU_2020_TWIW_01_CZE_PRA_026 | Other | ERR10436709 |
| CZE | 2020 | DTU_2020_TWIW_01_CZE_PRA_027 | Wound Pus Biopsy | ERR10436724 |
| CZE | 2020 | DTU_2020_TWIW_01_CZE_PRA_029 | Wound Pus Biopsy | ERR10436723 |
| CZE | 2020 | DTU_2020_TWIW_01_CZE_PRA_030 | Blood | ERR10436746 |
| CZE | 2020 | DTU_2020_TWIW_01_CZE_PRA_031 | Blood | ERR10436755 |
| CZE | 2020 | DTU_2020_TWIW_01_CZE_PRA_032 | Wound Pus Biopsy | ERR10436754 |
| CZE | 2020 | DTU_2020_TWIW_01_CZE_PRA_035 | Swab | ERR10436720 |
| CZE | 2020 | DTU_2020_TWIW_01_CZE_PRA_036 | Swab | ERR10436719 |
| CZE | 2020 | DTU_2020_TWIW_01_CZE_PRA_040 | Wound Pus Biopsy | ERR10436727 |
| CZE | 2020 | DTU_2020_TWIW_01_CZE_PRA_042 | Wound Pus Biopsy | ERR10436725 |
| CZE | 2020 | DTU_2020_TWIW_01_CZE_PRA_046 | Wound Pus Biopsy | ERR10436726 |
| CZE | 2020 | DTU_2020_TWIW_01_CZE_PRA_048 | Wound Pus Biopsy | ERR10436728 |
| CZE | 2020 | DTU_2020_TWIW_01_CZE_PRA_055 | Wound Pus Biopsy | ERR10436729 |
| CZE | 2020 | DTU_2020_TWIW_01_CZE_PRA_057B | Wound Pus Biopsy | ERR10436771 |
| CZE | 2020 | DTU_2020_TWIW_01_CZE_PRA_058 | Swab | ERR10436730 |
| CZE | 2020 | DTU_2020_TWIW_01_CZE_PRA_059 | Wound Pus Biopsy | ERR10436731 |
| CZE | 2020 | DTU_2020_TWIW_01_CZE_PRA_060 | Other | ERR10436763 |
| NZL | 2020 | DTU_2020_TWIW_01_NZL_DUN_002 | Blood | ERR10438786 |
| NZL | 2020 | DTU_2020_TWIW_01_NZL_DUN_004 | Swab | ERR10438779 |
| NZL | 2020 | DTU_2020_TWIW_01_NZL_DUN_005 | Swab | ERR10438780 |
| NZL | 2020 | DTU_2020_TWIW_01_NZL_DUN_006 | Swab | ERR10438785 |
| NZL | 2020 | DTU_2020_TWIW_01_NZL_DUN_007 | Swab | ERR10438781 |
| NZL | 2020 | DTU_2020_TWIW_01_NZL_DUN_008 | Swab | ERR10438783 |
| NZL | 2020 | DTU_2020_TWIW_01_NZL_DUN_009 | Swab | ERR10438801 |
| NZL | 2020 | DTU_2020_TWIW_01_NZL_DUN_012 | Urine | ERR10438810 |
| NZL | 2020 | DTU_2020_TWIW_01_NZL_DUN_019 | Urine | ERR10438800 |
| NZL | 2020 | DTU_2020_TWIW_01_NZL_DUN_020 | Respiratory System | ERR10438784 |
| NZL | 2020 | DTU_2020_TWIW_01_NZL_DUN_022 | Urine | ERR10438759 |
| NZL | 2020 | DTU_2020_TWIW_01_NZL_DUN_023 | Urine | ERR10438803 |
| NZL | 2020 | DTU_2020_TWIW_01_NZL_DUN_032 | Swab | ERR10438798 |
| NZL | 2020 | DTU_2020_TWIW_01_NZL_DUN_033 | Swab | ERR10438787 |
| NZL | 2020 | DTU_2020_TWIW_01_NZL_DUN_034 | Swab | ERR10438788 |
| NZL | 2020 | DTU_2020_TWIW_01_NZL_DUN_035 | Swab | ERR10438778 |
| NZL | 2020 | DTU_2020_TWIW_01_NZL_DUN_040 | Swab | ERR10438797 |
| NZL | 2020 | DTU_2020_TWIW_01_NZL_DUN_041 | Swab | ERR10438796 |
| NZL | 2020 | DTU_2020_TWIW_01_NZL_DUN_042 | Swab | ERR10438789 |
| NZL | 2020 | DTU_2020_TWIW_01_NZL_DUN_047 | Swab | ERR10438804 |
| NZL | 2020 | DTU_2020_TWIW_01_NZL_DUN_048 | Wound Pus Biopsy | ERR10438790 |
| NZL | 2020 | DTU_2020_TWIW_01_NZL_DUN_049 | Swab | ERR10438812 |
| NZL | 2020 | DTU_2020_TWIW_01_NZL_DUN_050 | Wound Pus Biopsy | ERR10438794 |
| NZL | 2020 | DTU_2020_TWIW_01_NZL_DUN_051 | Swab | ERR10438792 |
| NZL | 2020 | DTU_2020_TWIW_01_NZL_DUN_052 | Swab | ERR10438793 |
| NZL | 2020 | DTU_2020_TWIW_01_NZL_DUN_060 | Urine | ERR10438795 |
| NZL | 2020 | DTU_2020_TWIW_01_NZL_DUN_061 | Swab | ERR10438809 |
| NZL | 2020 | DTU_2020_TWIW_01_NZL_DUN_062 | Swab | ERR10438799 |
| NZL | 2020 | DTU_2020_TWIW_01_NZL_DUN_063 | Swab | ERR10438802 |
| NZL | 2020 | DTU_2020_TWIW_01_NZL_DUN_064 | Urine | ERR10438811 |
| NZL | 2020 | DTU_2020_TWIW_01_NZL_DUN_065 | Urine | ERR10438808 |
| COL | 2020 | DTU_2020_TWIW_01_COL_BOG_005 | Blood | ERR10441476 |
| COL | 2020 | DTU_2020_TWIW_01_COL_BOG_008 | Blood | ERR10441475 |
| COL | 2020 | DTU_2020_TWIW_01_COL_BOG_010 | Blood | ERR10441478 |
| COL | 2020 | DTU_2020_TWIW_01_COL_BOG_019 | Blood | ERR10441469 |
| TUR | 2020 | DTU_2020_TWIW_01_TUR_ORT_005 | Urine | ERR10432770 |
| TUR | 2020 | DTU_2020_TWIW_01_TUR_ORT_006 | Blood | ERR10432762 |
| TUR | 2020 | DTU_2020_TWIW_01_TUR_ORT_009 | Respiratory System | ERR10432733 |
| TUR | 2020 | DTU_2020_TWIW_01_TUR_ORT_010 | Blood | ERR10432755 |
| TUR | 2020 | DTU_2020_TWIW_01_TUR_ORT_013 | Urine | ERR10432768 |
| TUR | 2020 | DTU_2020_TWIW_01_TUR_ORT_017 | Respiratory System | ERR10432751 |
| TUR | 2020 | DTU_2020_TWIW_01_TUR_ORT_022 | Respiratory System | ERR10432754 |
| TUR | 2020 | DTU_2020_TWIW_01_TUR_ORT_023 | Wound Pus Biopsy | ERR10432756 |
| TUR | 2020 | DTU_2020_TWIW_01_TUR_ORT_028 | Respiratory System | ERR10432760 |
| TUR | 2020 | DTU_2020_TWIW_01_TUR_ORT_030 | Urine | ERR10432774 |
| TUR | 2020 | DTU_2020_TWIW_01_TUR_ORT_035 | Blood | ERR10432758 |
| TUR | 2020 | DTU_2020_TWIW_01_TUR_ORT_036 | Blood | ERR10432761 |
| TUR | 2020 | DTU_2020_TWIW_01_TUR_ORT_037 | Blood | ERR10432773 |
| TUR | 2020 | DTU_2020_TWIW_01_TUR_ORT_039 | Respiratory System | ERR10432772 |
| TUR | 2020 | DTU_2020_TWIW_01_TUR_ORT_042 | Blood | ERR10432776 |
| TUR | 2020 | DTU_2020_TWIW_01_TUR_ORT_043 | Wound Pus Biopsy | ERR10432766 |
| TUR | 2020 | DTU_2020_TWIW_01_TUR_ORT_049 | Urine | ERR10432765 |
| TUR | 2020 | DTU_2020_TWIW_01_TUR_ORT_055 | Other | ERR10432781 |
| HRV | 2020 | DTU_2020_TWIW_01_HRV_ZAG_017 | Urine | ERR10441516 |
| HRV | 2020 | DTU_2020_TWIW_01_HRV_ZAG_030 | Urine | ERR10441537 |
| HRV | 2020 | DTU_2020_TWIW_01_HRV_ZAG_042 | Urine | ERR10441538 |
| HRV | 2020 | DTU_2020_TWIW_01_HRV_ZAG_043 | Urine | ERR10441597 |
| HRV | 2020 | DTU_2020_TWIW_01_HRV_ZAG_044 | Urine | ERR10441548 |
| HRV | 2020 | DTU_2020_TWIW_01_HRV_ZAG_049 | Respiratory System | ERR10441545 |
| HRV | 2020 | DTU_2020_TWIW_01_HRV_ZAG_050 | Respiratory System | ERR10441561 |
| HRV | 2020 | DTU_2020_TWIW_01_HRV_ZAG_051 | Respiratory System | ERR10441586 |
| FRA | 2020 | DTU_2020_TWIW_01_FRA_LIL_033 | Respiratory System | ERR10437730 |
| ESP | 2020 | DTU_2020_TWIW_01_ESP_BAR_002 | Respiratory System | ERR10432402 |
| ESP | 2020 | DTU_2020_TWIW_01_ESP_BAR_019 | Blood | ERR10432430 |
| ESP | 2020 | DTU_2020_TWIW_01_ESP_BAR_020 | Blood | ERR10432451 |
| ESP | 2020 | DTU_2020_TWIW_01_ESP_BAR_022 | Urine | ERR10432467 |
| ESP | 2020 | DTU_2020_TWIW_01_ESP_BAR_023 | Urine | ERR10432436 |
| ESP | 2020 | DTU_2020_TWIW_01_ESP_BAR_032 | Urine | NA |
| ESP | 2020 | DTU_2020_TWIW_01_ESP_BAR_033 | Blood | NA |
| ESP | 2020 | DTU_2020_TWIW_01_ESP_BAR_034 | Urine | NA |
| ESP | 2020 | DTU_2020_TWIW_01_ESP_BAR_036 | Urine | NA |
| ESP | 2020 | DTU_2020_TWIW_01_ESP_BAR_037 | Wound Pus Biopsy | NA |
| ESP | 2020 | DTU_2020_TWIW_01_ESP_BAR_038 | Urine | NA |
| ESP | 2020 | DTU_2020_TWIW_01_ESP_BAR_039 | Wound Pus Biopsy | ERR10432450 |
| ITA | 2020 | DTU_2020_TWIW_01_ITA_MIL_059 | Blood | ERR10438638 |
| LTU | 2020 | DTU_2020_TWIW_01_LTU_KAU_058 | Respiratory System | ERR10438706 |
| DEU | 2020 | DTU_2020_TWIW_01_DEU_MAG_038 | Wound Pus Biopsy | ERR10437800 |
| GHA | 2020 | DTU_2020_TWIW_01_GHA_SEK_017 | Respiratory System | ERR10438021 |
| CZE | 2020 | DTU_2020_TWIW_01_CZE_PLZ_023 | Respiratory System | ERR10441679 |
| CZE | 2020 | DTU_2020_TWIW_01_CZE_PLZ_055 | Wound Pus Biopsy | ERR10436732 |
| THA | 2020 | DTU_2020_TWIW_01_THA_SAR_023 | Respiratory System | ERR10432708 |
| SAU | 2020 | DTU_2020_TWIW_01_SAU_A1Q_001 | Blood | ERR10432395 |
| SAU | 2020 | DTU_2020_TWIW_01_SAU_A1Q_023 | Urine | ERR10432413 |
| DNK | 2020 | DTU_2020_TWIW_01_DNK_HVI_003 | Wound Pus Biopsy | ERR10437041 |
| DNK | 2020 | DTU_2020_TWIW_01_DNK_HVI_007 | Blood | ERR10437046 |
| DNK | 2020 | DTU_2020_TWIW_01_DNK_HVI_029 | Wound Pus Biopsy | ERR10437029 |
| DNK | 2020 | DTU_2020_TWIW_01_DNK_HVI_066 | Urine | ERR10436954 |
| FRA | 2020 | DTU_2020_TWIW_01_FRA_VAL_031 | Blood | ERR10437418 |
| FRA | 2020 | DTU_2020_TWIW_01_FRA_VAL_046 | Blood | ERR10437579 |
| GRL | 2020 | DTU_2020_TWIW_01_GRL_NUU_036 | Urine | ERR10438065 |
| GRL | 2020 | DTU_2020_TWIW_01_GRL_NUU_057 | Other | ERR10438173 |
| NOR | 2020 | DTU_2020_TWIW_01_NOR_LÃ˜R_012 | Urine | ERR10431462 |
| NOR | 2020 | DTU_2020_TWIW_01_NOR_LÃ˜R_014 | Wound Pus Biopsy | ERR10431434 |
| NOR | 2020 | DTU_2020_TWIW_01_NOR_LÃ˜R_024 | Urine | ERR10439707 |
| NOR | 2020 | DTU_2020_TWIW_01_NOR_LÃ˜R_051 | Other | ERR10431426 |
| NOR | 2020 | DTU_2020_TWIW_01_NOR_LÃ˜R_057 | Respiratory System | ERR10431439 |
| NOR | 2020 | DTU_2020_TWIW_01_NOR_LÃ˜R_058 | Wound Pus Biopsy | ERR10431443 |
| CAN | 2020 | DTU_2020_TWIW_01_CAN_EDM_001 | Urine | ERR10441298 |
| CAN | 2020 | DTU_2020_TWIW_01_CAN_EDM_006 | Other | ERR10441387 |
| CAN | 2020 | DTU_2020_TWIW_01_CAN_EDM_006 | Other | ERR14129340 |
| CAN | 2020 | DTU_2020_TWIW_01_CAN_EDM_019 | Respiratory System | ERR10441401 |
| CAN | 2020 | DTU_2020_TWIW_01_CAN_EDM_034 | Urine | ERR10441368 |
| CAN | 2020 | DTU_2020_TWIW_01_CAN_EDM_040 | Swab | ERR10441384 |
| CAN | 2020 | DTU_2020_TWIW_01_CAN_EDM_057 | Urine | ERR10441407 |
| AUS | 2020 | DTU_2020_TWIW_01_AUS_MUR_022 | Urine | ERR10431365 |
| AUS | 2020 | DTU_2020_TWIW_01_AUS_MUR_050 | Respiratory System | ERR10431357 |
| NGA | 2020 | DTU_2020_TWIW_01_NGA_ABU_007 | Wound Pus Biopsy | ERR10438985 |
| NGA | 2020 | DTU_2020_TWIW_01_NGA_ABU_021 | Blood | ERR10439030 |
| NGA | 2020 | DTU_2020_TWIW_01_NGA_ABU_031 | Urine | ERR10438843 |
| NGA | 2020 | DTU_2020_TWIW_01_NGA_ABU_046 | Blood | ERR10439000 |
| CAN | 2020 | DTU_2020_TWIW_01_CAN_EDM_025A | Wound Pus Biopsy | ERR10441412 |
| ALB | 2020 | DTU_2020_TWIW_01_ALB_TIR_001 | Blood | ERR10435931 |
| ALB | 2020 | DTU_2020_TWIW_01_ALB_TIR_002 | Respiratory System | ERR10430212 |
| ALB | 2020 | DTU_2020_TWIW_01_ALB_TIR_039 | Urine | ERR10435943 |
| ALB | 2020 | DTU_2020_TWIW_01_ALB_TIR_040 | Urine | ERR10430222 |
| BFA | 2020 | DTU_2020_TWIN_01_BFA_OUA_016 | Wound Pus Biopsy | ERR10441270 |
| BFA | 2020 | DTU_2020_TWIN_01_BFA_OUA_031 | Wound Pus Biopsy | ERR10441212 |
| CHE | 2020 | DTU_2020_TWIW_01_CHE_LUZ_003 | Blood | ERR10432523 |
| CHE | 2020 | DTU_2020_TWIW_01_CHE_LUZ_014 | Urine | ERR10432496 |
| PAK | 2020 | DTU_2020_TWIW_01_PAK_PES_001 | Urine | ERR10431458 |
| PAK | 2020 | DTU_2020_TWIW_01_PAK_PES_003 | Wound Pus Biopsy | ERR10431542 |
| PAK | 2020 | DTU_2020_TWIW_01_PAK_PES_029 | Wound Pus Biopsy | ERR10431466 |
| PAK | 2020 | DTU_2020_TWIW_01_PAK_PES_030 | Urine | ERR10431521 |
| PAK | 2020 | DTU_2020_TWIW_01_PAK_PES_044 | Urine | ERR10431477 |
| PAK | 2020 | DTU_2020_TWIW_01_PAK_PES_045 | Blood | ERR10431497 |
| PAK | 2020 | DTU_2020_TWIW_01_PAK_PES_046 | Other | ERR10431552 |
| CHE | 2020 | DTU_2020_TWIW_01_CHE_BAS_025 | Wound Pus Biopsy | ERR10432565 |
| CHE | 2020 | DTU_2020_TWIW_01_CHE_BAS_034 | Blood | ERR10432530 |
| CHE | 2020 | DTU_2020_TWIW_01_CHE_BAS_040 | Swab | ERR10432536 |
| CHE | 2020 | DTU_2020_TWIW_01_CHE_BAS_059 | Wound Pus Biopsy | ERR10432560 |
| AUS | 2020 | DTU_2020_TWIW_01_AUS_CAN_053 | Swab | ERR10431333 |
| BEN | 2020 | DTU_2020_TWIW_01_BEN_COT_009 | Urine | ERR10436246 |
| BEN | 2020 | DTU_2020_TWIW_01_BEN_COT_010 | Urine | ERR10436260 |
| BEN | 2020 | DTU_2020_TWIW_01_BEN_COT_057 | Urine | ERR10436291 |
| PAK | 2020 | DTU_2020_TWIW_01_PAK_RAW_031 | Urine | ERR10431585 |
| PAK | 2020 | DTU_2020_TWIW_01_PAK_RAW_035 | Blood | ERR10431478 |
| BFA | 2020 | DTU_2020_TWIW_01_BFA_NAN_001 | Other | ERR10441165 |
| BFA | 2020 | DTU_2020_TWIW_01_BFA_NAN_002 | Urine | ERR10441173 |
| BFA | 2020 | DTU_2020_TWIW_01_BFA_NAN_003 | Urine | ERR10441302 |
| BFA | 2020 | DTU_2020_TWIW_01_BFA_NAN_004 | Urine | ERR10441190 |
| BFA | 2020 | DTU_2020_TWIW_01_BFA_NAN_007 | Urine | ERR10441160 |
| BFA | 2020 | DTU_2020_TWIW_01_BFA_NAN_008 | Other | ERR10441234 |
| BFA | 2020 | DTU_2020_TWIW_01_BFA_NAN_009 | Other | ERR10441145 |
| BFA | 2020 | DTU_2020_TWIW_01_BFA_NAN_010 | Wound Pus Biopsy | ERR10441300 |
| BFA | 2020 | DTU_2020_TWIW_01_BFA_NAN_011 | Urine | ERR10441202 |
| BFA | 2020 | DTU_2020_TWIW_01_BFA_NAN_012 | Urine | ERR10441174 |
| BFA | 2020 | DTU_2020_TWIW_01_BFA_NAN_013 | Urine | ERR10441163 |
| BFA | 2020 | DTU_2020_TWIW_01_BFA_NAN_014 | Wound Pus Biopsy | ERR10446196 |
| BFA | 2020 | DTU_2020_TWIW_01_BFA_NAN_015 | Swab | ERR10441161 |
| BFA | 2020 | DTU_2020_TWIW_01_BFA_NAN_016 | Urine | ERR10441183 |
| BFA | 2020 | DTU_2020_TWIW_01_BFA_NAN_019 | Urine | ERR10441246 |
| BFA | 2020 | DTU_2020_TWIW_01_BFA_NAN_020 | Urine | ERR10441199 |
| BFA | 2020 | DTU_2020_TWIW_01_BFA_NAN_021 | Urine | ERR10441192 |
| BFA | 2020 | DTU_2020_TWIW_01_BFA_NAN_022 | Urine | ERR10441243 |
| BFA | 2020 | DTU_2020_TWIW_01_BFA_NAN_023 | Urine | ERR10441248 |
| BFA | 2020 | DTU_2020_TWIW_01_BFA_NAN_024 | Urine | ERR10441241 |
| BFA | 2020 | DTU_2020_TWIW_01_BFA_NAN_025 | Swab | ERR10441238 |
| BFA | 2020 | DTU_2020_TWIW_01_BFA_NAN_026 | Urine | ERR10441211 |
| BFA | 2020 | DTU_2020_TWIW_01_BFA_NAN_027 | Urine | ERR10441195 |
| BFA | 2020 | DTU_2020_TWIW_01_BFA_NAN_029 | Urine | ERR10441215 |
| BFA | 2020 | DTU_2020_TWIW_01_BFA_NAN_030 | Swab | ERR10441237 |
| BFA | 2020 | DTU_2020_TWIW_01_BFA_NAN_031 | Urine | ERR10441186 |
| BFA | 2020 | DTU_2020_TWIW_01_BFA_NAN_032 | Other | ERR10446191 |
| BFA | 2020 | DTU_2020_TWIW_01_BFA_NAN_033 | Urine | ERR10441277 |
| BFA | 2020 | DTU_2020_TWIW_01_BFA_NAN_034 | Urine | ERR10441134 |
| BFA | 2020 | DTU_2020_TWIW_01_BFA_NAN_036 | Wound Pus Biopsy | ERR10441256 |
| BFA | 2020 | DTU_2020_TWIW_01_BFA_NAN_037 | Urine | ERR10446192 |
| BFA | 2020 | DTU_2020_TWIW_01_BFA_NAN_038 | Urine | ERR10441262 |
| BFA | 2020 | DTU_2020_TWIW_01_BFA_NAN_039 | Urine | ERR10441166 |
| BFA | 2020 | DTU_2020_TWIW_01_BFA_NAN_040 | Urine | ERR10441181 |
| BFA | 2020 | DTU_2020_TWIW_01_BFA_NAN_041 | Urine | ERR10441162 |
| BFA | 2020 | DTU_2020_TWIW_01_BFA_NAN_042 | Urine | ERR10441179 |
| BFA | 2020 | DTU_2020_TWIW_01_BFA_NAN_043 | Urine | ERR10441182 |
| BFA | 2020 | DTU_2020_TWIW_01_BFA_NAN_044 | Urine | ERR10446199 |
| BFA | 2020 | DTU_2020_TWIW_01_BFA_NAN_046 | Urine | ERR10441223 |
| BFA | 2020 | DTU_2020_TWIW_01_BFA_NAN_048 | Urine | ERR10441184 |
| BFA | 2020 | DTU_2020_TWIW_01_BFA_NAN_049 | Urine | ERR10441156 |
| BFA | 2020 | DTU_2020_TWIW_01_BFA_NAN_050 | Urine | ERR10441249 |
| BFA | 2020 | DTU_2020_TWIW_01_BFA_NAN_051 | Urine | ERR10441180 |
| BFA | 2020 | DTU_2020_TWIW_01_BFA_NAN_052 | Urine | ERR10441185 |
| BFA | 2020 | DTU_2020_TWIW_01_BFA_NAN_053 | Urine | ERR10441230 |
| BFA | 2020 | DTU_2020_TWIW_01_BFA_NAN_054 | Urine | ERR10441176 |
| BFA | 2020 | DTU_2020_TWIW_01_BFA_NAN_055 | Urine | ERR10441201 |
| BFA | 2020 | DTU_2020_TWIW_01_BFA_NAN_056 | Urine | ERR10441178 |
| BFA | 2020 | DTU_2020_TWIW_01_BFA_NAN_057 | Other | ERR10441255 |
| BFA | 2020 | DTU_2020_TWIW_01_BFA_NAN_058 | Urine | ERR10441233 |
| BFA | 2020 | DTU_2020_TWIW_01_BFA_NAN_061 | Urine | ERR10441189 |
| BFA | 2020 | DTU_2020_TWIW_01_BFA_NAN_062 | Urine | ERR10441265 |
| BFA | 2020 | DTU_2020_TWIW_01_BFA_NAN_063 | Urine | ERR10441254 |
| BFA | 2020 | DTU_2020_TWIW_01_BFA_NAN_064 | Wound Pus Biopsy | ERR10441257 |
| BFA | 2020 | DTU_2020_TWIW_01_BFA_NAN_065 | Urine | ERR10441159 |
| BFA | 2020 | DTU_2020_TWIW_01_BFA_NAN_066 | Urine | ERR10441146 |
| BFA | 2020 | DTU_2020_TWIW_01_BFA_NAN_067 | Urine | ERR10441171 |
| BFA | 2020 | DTU_2020_TWIW_01_BFA_NAN_068 | Swab | ERR10441245 |
| BFA | 2020 | DTU_2020_TWIW_01_BFA_NAN_069 | Wound Pus Biopsy | ERR10441261 |
| BFA | 2020 | DTU_2020_TWIW_01_BFA_NAN_070 | Wound Pus Biopsy | ERR10441260 |
| BGR | 2020 | DTU_2020_TWIW_01_BGR_SOF_001 | Swab | ERR10436414 |
| BGR | 2020 | DTU_2020_TWIW_01_BGR_SOF_002 | Swab | ERR10441057 |
| BGR | 2020 | DTU_2020_TWIW_01_BGR_SOF_003 | Other | ERR10436360 |
| BGR | 2020 | DTU_2020_TWIW_01_BGR_SOF_004 | Blood | ERR10441111 |
| BGR | 2020 | DTU_2020_TWIW_01_BGR_SOF_006 | Swab | ERR10441090 |
| BGR | 2020 | DTU_2020_TWIW_01_BGR_SOF_008 | Swab | ERR10436376 |
| BGR | 2020 | DTU_2020_TWIW_01_BGR_SOF_009 | Swab | ERR10441097 |
| BGR | 2020 | DTU_2020_TWIW_01_BGR_SOF_010 | Wound Pus Biopsy | ERR10436380 |
| BGR | 2020 | DTU_2020_TWIW_01_BGR_SOF_013 | Swab | ERR10436379 |
| BGR | 2020 | DTU_2020_TWIW_01_BGR_SOF_014 | Swab | ERR10441061 |
| BGR | 2020 | DTU_2020_TWIW_01_BGR_SOF_018 | Urine | ERR10441107 |
| BGR | 2020 | DTU_2020_TWIW_01_BGR_SOF_021 | Swab | ERR10436416 |
| BGR | 2020 | DTU_2020_TWIW_01_BGR_SOF_028 | Swab | ERR10441079 |
| BGR | 2020 | DTU_2020_TWIW_01_BGR_SOF_031 | Urine | ERR10441096 |
| BGR | 2020 | DTU_2020_TWIW_01_BGR_SOF_032 | Swab | ERR10441151 |
| BGR | 2020 | DTU_2020_TWIW_01_BGR_SOF_034 | Swab | ERR10441108 |
| BGR | 2020 | DTU_2020_TWIW_01_BGR_SOF_037 | Swab | ERR10436422 |
| BGR | 2020 | DTU_2020_TWIW_01_BGR_SOF_039 | Swab | ERR10436418 |
| BGR | 2020 | DTU_2020_TWIW_01_BGR_SOF_043 | Swab | ERR10436419 |
| BGR | 2020 | DTU_2020_TWIW_01_BGR_SOF_045 | Urine | ERR10441135 |
| BGR | 2020 | DTU_2020_TWIW_01_BGR_SOF_046 | Urine | ERR10441197 |
| BGR | 2020 | DTU_2020_TWIW_01_BGR_SOF_049 | Wound Pus Biopsy | ERR10436417 |
| BGR | 2020 | DTU_2020_TWIW_01_BGR_SOF_051 | Other | ERR10441127 |
| BGR | 2020 | DTU_2020_TWIW_01_BGR_SOF_052 | Swab | ERR10436420 |
| BGR | 2020 | DTU_2020_TWIW_01_BGR_SOF_053 | Swab | ERR10436421 |
| BGR | 2020 | DTU_2020_TWIW_01_BGR_SOF_054 | Swab | ERR10436423 |
| BGR | 2020 | DTU_2020_TWIW_01_BGR_SOF_055 | Swab | ERR10441092 |
| BGR | 2020 | DTU_2020_TWIW_01_BGR_SOF_057 | Urine | ERR10441118 |
| BGR | 2020 | DTU_2020_TWIW_01_BGR_SOF_059 | Urine | ERR10441083 |
| BGR | 2020 | DTU_2020_TWIW_01_BGR_SOF_061 | Urine | ERR10441157 |
| BGR | 2020 | DTU_2020_TWIW_01_BGR_SOF_062 | Urine | ERR10436369 |
| BGR | 2020 | DTU_2020_TWIW_01_BGR_SOF_063 | Wound Pus Biopsy | ERR10436415 |
| BGR | 2020 | DTU_2020_TWIW_01_BGR_SOF_064 | Urine | ERR10441168 |
| CAN | 2020 | DTU_2020_TWIW_01_CAN_SHE_011 | Other | ERR10441306 |
| CAN | 2020 | DTU_2020_TWIW_01_CAN_SHE_012 | Respiratory System | ERR10441286 |
| CAN | 2020 | DTU_2020_TWIW_01_CAN_SHE_013 | Urine | ERR10441294 |
| CAN | 2020 | DTU_2020_TWIW_01_CAN_SHE_017 | Blood | ERR10446208 |
| CAN | 2020 | DTU_2020_TWIW_01_CAN_SHE_020 | Blood | ERR10441312 |
| CAN | 2020 | DTU_2020_TWIW_01_CAN_SHE_021 | Other | ERR10441409 |
| CAN | 2020 | DTU_2020_TWIW_01_CAN_SHE_022 | Other | ERR10441331 |
| CAN | 2020 | DTU_2020_TWIW_01_CAN_SHE_023 | Other | ERR10441406 |
| CAN | 2020 | DTU_2020_TWIW_01_CAN_SHE_024 | Other | ERR10441405 |
| CAN | 2020 | DTU_2020_TWIW_01_CAN_SHE_026 | Other | ERR10441316 |
| CAN | 2020 | DTU_2020_TWIW_01_CAN_SHE_027 | Wound Pus Biopsy | ERR10441295 |
| CAN | 2020 | DTU_2020_TWIW_01_CAN_SHE_028 | Other | ERR10441287 |
| CAN | 2020 | DTU_2020_TWIW_01_CAN_SHE_031 | Urine | ERR10441417 |
| CAN | 2020 | DTU_2020_TWIW_01_CAN_SHE_032 | Urine | ERR10441283 |
| CAN | 2020 | DTU_2020_TWIW_01_CAN_SHE_033 | Urine | ERR10441303 |
| CAN | 2020 | DTU_2020_TWIW_01_CAN_SHE_034 | Urine | ERR10441285 |
| CAN | 2020 | DTU_2020_TWIW_01_CAN_SHE_035 | Urine | ERR10441315 |
| CAN | 2020 | DTU_2020_TWIW_01_CAN_SHE_036 | Urine | ERR10441291 |
| CAN | 2020 | DTU_2020_TWIW_01_CAN_SHE_037 | Urine | ERR10441293 |
| CAN | 2020 | DTU_2020_TWIW_01_CAN_SHE_038 | Urine | ERR10441292 |
| CAN | 2020 | DTU_2020_TWIW_01_CAN_SHE_039 | Urine | ERR10441322 |
| CAN | 2020 | DTU_2020_TWIW_01_CAN_SHE_040 | Urine | ERR10441309 |
| CAN | 2020 | DTU_2020_TWIW_01_CAN_SHE_041 | Urine | ERR10441416 |
| CAN | 2020 | DTU_2020_TWIW_01_CAN_SHE_042 | Urine | ERR10441394 |
| CAN | 2020 | DTU_2020_TWIW_01_CAN_SHE_043 | Urine | ERR10441402 |
| CAN | 2020 | DTU_2020_TWIW_01_CAN_SHE_044 | Urine | ERR10441444 |
| CAN | 2020 | DTU_2020_TWIW_01_CAN_SHE_045 | Urine | ERR10441430 |
| CAN | 2020 | DTU_2020_TWIW_01_CAN_SHE_046 | Urine | ERR10441297 |
| CAN | 2020 | DTU_2020_TWIW_01_CAN_SHE_047 | Urine | ERR10441410 |
| CAN | 2020 | DTU_2020_TWIW_01_CAN_SHE_048 | Urine | ERR10441299 |
| CAN | 2020 | DTU_2020_TWIW_01_CAN_SHE_050 | Urine | ERR10441365 |
| CAN | 2020 | DTU_2020_TWIW_01_CAN_SHE_051 | Urine | ERR10446351 |
| CAN | 2020 | DTU_2020_TWIW_01_CAN_SHE_052 | Urine | ERR10441419 |
| CAN | 2020 | DTU_2020_TWIW_01_CAN_SHE_053 | Urine | ERR10441320 |
| CAN | 2020 | DTU_2020_TWIW_01_CAN_SHE_055 | Urine | ERR10441289 |
| CAN | 2020 | DTU_2020_TWIW_01_CAN_SHE_056 | Urine | ERR10441415 |
| CAN | 2020 | DTU_2020_TWIW_01_CAN_SHE_057 | Wound Pus Biopsy | ERR10441447 |
| CAN | 2020 | DTU_2020_TWIW_01_CAN_SHE_058 | Wound Pus Biopsy | ERR10441472 |
| CAN | 2020 | DTU_2020_TWIW_01_CAN_SHE_059 | Wound Pus Biopsy | ERR10441335 |
| CAN | 2020 | DTU_2020_TWIW_01_CAN_SHE_060 | Other | ERR10441383 |
| NGA | 2020 | DTU_2020_TWIW_01_NGA_ILE_001 | Blood | ERR10438816 |
| NGA | 2020 | DTU_2020_TWIW_01_NGA_ILE_001 | Blood | ERR12041388 |
| NGA | 2020 | DTU_2020_TWIW_01_NGA_ILE_003 | Wound Pus Biopsy | ERR10438960 |
| NGA | 2020 | DTU_2020_TWIW_01_NGA_ILE_004 | Blood | ERR10439009 |
| NGA | 2020 | DTU_2020_TWIW_01_NGA_ILE_005 | Urine | ERR10438870 |
| NGA | 2020 | DTU_2020_TWIW_01_NGA_ILE_006 | Wound Pus Biopsy | ERR10438822 |
| NGA | 2020 | DTU_2020_TWIW_01_NGA_ILE_007 | Urine | ERR10438871 |
| NGA | 2020 | DTU_2020_TWIW_01_NGA_ILE_008 | Urine | ERR10438874 |
| NGA | 2020 | DTU_2020_TWIW_01_NGA_ILE_009 | Blood | ERR10438989 |
| NGA | 2020 | DTU_2020_TWIW_01_NGA_ILE_011 | Respiratory System | ERR10438934 |
| NGA | 2020 | DTU_2020_TWIW_01_NGA_ILE_012 | Urine | ERR10446210 |
| NGA | 2020 | DTU_2020_TWIW_01_NGA_ILE_014 | Urine | ERR10439006 |
| NGA | 2020 | DTU_2020_TWIW_01_NGA_ILE_017 | Urine | ERR10438844 |
| NGA | 2020 | DTU_2020_TWIW_01_NGA_ILE_023 | Urine | ERR10438884 |
| NGA | 2020 | DTU_2020_TWIW_01_NGA_ILE_024 | Urine | ERR10438886 |
| NGA | 2020 | DTU_2020_TWIW_01_NGA_ILE_025 | Urine | ERR10438885 |
| NGA | 2020 | DTU_2020_TWIW_01_NGA_ILE_027 | Blood | ERR10446204 |
| NGA | 2020 | DTU_2020_TWIW_01_NGA_ILE_028 | Wound Pus Biopsy | ERR10439056 |
| NGA | 2020 | DTU_2020_TWIW_01_NGA_ILE_029 | Respiratory System | ERR10438948 |
| NGA | 2020 | DTU_2020_TWIW_01_NGA_ILE_030 | Wound Pus Biopsy | ERR10438828 |
| NGA | 2020 | DTU_2020_TWIW_01_NGA_ILE_031 | Swab | ERR10439025 |
| NGA | 2020 | DTU_2020_TWIW_01_NGA_ILE_033 | Urine | ERR10439002 |
| NGA | 2020 | DTU_2020_TWIW_01_NGA_ILE_034 | Wound Pus Biopsy | ERR10438827 |
| NGA | 2020 | DTU_2020_TWIW_01_NGA_ILE_035 | Wound Pus Biopsy | ERR10439005 |
| NGA | 2020 | DTU_2020_TWIW_01_NGA_ILE_036 | Urine | ERR10439014 |
| NGA | 2020 | DTU_2020_TWIW_01_NGA_ILE_037 | Blood | ERR10439052 |
| NGA | 2020 | DTU_2020_TWIW_01_NGA_ILE_038 | Wound Pus Biopsy | ERR10438981 |
| NGA | 2020 | DTU_2020_TWIW_01_NGA_ILE_039 | Wound Pus Biopsy | ERR10438888 |
| NGA | 2020 | DTU_2020_TWIW_01_NGA_ILE_040 | Urine | ERR10439024 |
| NGA | 2020 | DTU_2020_TWIW_01_NGA_ILE_041 | Urine | ERR10438952 |
| NGA | 2020 | DTU_2020_TWIW_01_NGA_ILE_042 | Urine | ERR10439032 |
| NGA | 2020 | DTU_2020_TWIW_01_NGA_ILE_043 | Wound Pus Biopsy | ERR10439029 |
| NGA | 2020 | DTU_2020_TWIW_01_NGA_ILE_044 | Urine | ERR10439035 |
| NGA | 2020 | DTU_2020_TWIW_01_NGA_ILE_045 | Urine | ERR10438841 |
| NGA | 2020 | DTU_2020_TWIW_01_NGA_ILE_046 | Urine | ERR10438896 |
| NGA | 2020 | DTU_2020_TWIW_01_NGA_ILE_047 | Urine | ERR10438893 |
| NGA | 2020 | DTU_2020_TWIW_01_NGA_ILE_048 | Urine | ERR10438897 |
| NGA | 2020 | DTU_2020_TWIW_01_NGA_ILE_049 | Respiratory System | ERR10438954 |
| NGA | 2020 | DTU_2020_TWIW_01_NGA_ILE_050 | Urine | ERR10438832 |
| NGA | 2020 | DTU_2020_TWIW_01_NGA_ILE_051 | Blood | ERR10438898 |
| NGA | 2020 | DTU_2020_TWIW_01_NGA_ILE_052 | Respiratory System | ERR10438838 |
| NGA | 2020 | DTU_2020_TWIW_01_NGA_ILE_053 | Urine | ERR10438953 |
| NGA | 2020 | DTU_2020_TWIW_01_NGA_ILE_054 | Urine | ERR10438900 |
| NGA | 2020 | DTU_2020_TWIW_01_NGA_ILE_055 | Respiratory System | ERR12041384 |
| NGA | 2020 | DTU_2020_TWIW_01_NGA_ILE_055 | Respiratory System | ERR10438842 |
| NGA | 2020 | DTU_2020_TWIW_01_NGA_ILE_056 | Urine | ERR10438899 |
| NGA | 2020 | DTU_2020_TWIW_01_NGA_ILE_057 | Blood | ERR10438987 |
| NGA | 2020 | DTU_2020_TWIW_01_NGA_ILE_058 | Blood | ERR10439028 |
| NGA | 2020 | DTU_2020_TWIW_01_NGA_ILE_059 | Wound Pus Biopsy | ERR10438951 |
| NGA | 2020 | DTU_2020_TWIW_01_NGA_ILE_060 | Urine | ERR10438901 |
| HUN | 2020 | DTU_2020_TWIW_01_HUN_PEC_001 | Blood | ERR10438549 |
| HUN | 2020 | DTU_2020_TWIW_01_HUN_PEC_002 | Other | ERR10438444 |
| HUN | 2020 | DTU_2020_TWIW_01_HUN_PEC_003 | Wound Pus Biopsy | ERR10438556 |
| HUN | 2020 | DTU_2020_TWIW_01_HUN_PEC_004 | Urine | ERR10438431 |
| HUN | 2020 | DTU_2020_TWIW_01_HUN_PEC_007 | Other | ERR10438517 |
| HUN | 2020 | DTU_2020_TWIW_01_HUN_PEC_009 | Wound Pus Biopsy | ERR10438446 |
| HUN | 2020 | DTU_2020_TWIW_01_HUN_PEC_010 | Other | ERR10438447 |
| HUN | 2020 | DTU_2020_TWIW_01_HUN_PEC_011 | Urine | ERR10438509 |
| HUN | 2020 | DTU_2020_TWIW_01_HUN_PEC_012 | Urine | ERR10438460 |
| HUN | 2020 | DTU_2020_TWIW_01_HUN_PEC_013 | Urine | ERR10438456 |
| HUN | 2020 | DTU_2020_TWIW_01_HUN_PEC_014 | Wound Pus Biopsy | ERR10438459 |
| HUN | 2020 | DTU_2020_TWIW_01_HUN_PEC_015 | Blood | ERR10438568 |
| HUN | 2020 | DTU_2020_TWIW_01_HUN_PEC_016 | Urine | ERR10438489 |
| HUN | 2020 | DTU_2020_TWIW_01_HUN_PEC_017 | Urine | ERR10438473 |
| HUN | 2020 | DTU_2020_TWIW_01_HUN_PEC_019 | Respiratory System | ERR10438410 |
| HUN | 2020 | DTU_2020_TWIW_01_HUN_PEC_020 | Blood | ERR10438402 |
| HUN | 2020 | DTU_2020_TWIW_01_HUN_PEC_021 | Urine | ERR10438586 |
| HUN | 2020 | DTU_2020_TWIW_01_HUN_PEC_023 | Urine | ERR10438474 |
| HUN | 2020 | DTU_2020_TWIW_01_HUN_PEC_026 | Blood | ERR10438505 |
| HUN | 2020 | DTU_2020_TWIW_01_HUN_PEC_027 | Urine | ERR10438482 |
| HUN | 2020 | DTU_2020_TWIW_01_HUN_PEC_028 | Blood | ERR10438574 |
| HUN | 2020 | DTU_2020_TWIW_01_HUN_PEC_029 | Other | ERR10438430 |
| HUN | 2020 | DTU_2020_TWIW_01_HUN_PEC_031 | Blood | ERR10438422 |
| HUN | 2020 | DTU_2020_TWIW_01_HUN_PEC_032 | Blood | ERR10438538 |
| HUN | 2020 | DTU_2020_TWIW_01_HUN_PEC_033 | Urine | ERR10438481 |
| HUN | 2020 | DTU_2020_TWIW_01_HUN_PEC_036 | Wound Pus Biopsy | ERR10438497 |
| HUN | 2020 | DTU_2020_TWIW_01_HUN_PEC_038 | Wound Pus Biopsy | ERR10438510 |
| HUN | 2020 | DTU_2020_TWIW_01_HUN_PEC_039 | Blood | ERR10438445 |
| HUN | 2020 | DTU_2020_TWIW_01_HUN_PEC_041 | Urine | ERR10438521 |
| HUN | 2020 | DTU_2020_TWIW_01_HUN_PEC_042 | Urine | ERR10438407 |
| HUN | 2020 | DTU_2020_TWIW_01_HUN_PEC_046 | Urine | ERR10438499 |
| HUN | 2020 | DTU_2020_TWIW_01_HUN_PEC_047 | Urine | ERR10438496 |
| HUN | 2020 | DTU_2020_TWIW_01_HUN_PEC_049 | Wound Pus Biopsy | ERR10438495 |
| HUN | 2020 | DTU_2020_TWIW_01_HUN_PEC_050 | Urine | ERR10438500 |
| HUN | 2020 | DTU_2020_TWIW_01_HUN_PEC_052 | Respiratory System | ERR10438587 |
| HUN | 2020 | DTU_2020_TWIW_01_HUN_PEC_054 | Blood | ERR10438429 |
| HUN | 2020 | DTU_2020_TWIW_01_HUN_PEC_055 | Blood | ERR10438498 |
| HUN | 2020 | DTU_2020_TWIW_01_HUN_PEC_056 | Blood | ERR10438507 |
| HUN | 2020 | DTU_2020_TWIW_01_HUN_PEC_058 | Blood | ERR10438573 |
| HUN | 2020 | DTU_2020_TWIW_01_HUN_PEC_060 | Respiratory System | ERR10438420 |
| CZE | 2020 | DTU_2020_TWIW_01_CZE_PRA_001B | Swab | ERR10436739 |
| CZE | 2020 | DTU_2020_TWIW_01_CZE_PRA_004 | Swab | ERR10441665 |
| CZE | 2020 | DTU_2020_TWIW_01_CZE_PRA_005 | Urine | ERR10441711 |
| CZE | 2020 | DTU_2020_TWIW_01_CZE_PRA_008 | Urine | ERR10436827 |
| CZE | 2020 | DTU_2020_TWIW_01_CZE_PRA_009 | Urine | ERR10436590 |
| CZE | 2020 | DTU_2020_TWIW_01_CZE_PRA_010 | Blood | ERR10436830 |
| CZE | 2020 | DTU_2020_TWIW_01_CZE_PRA_013 | Urine | ERR10441707 |
| CZE | 2020 | DTU_2020_TWIW_01_CZE_PRA_015 | Wound Pus Biopsy | ERR10436571 |
| CZE | 2020 | DTU_2020_TWIW_01_CZE_PRA_018 | Wound Pus Biopsy | ERR10441681 |
| CZE | 2020 | DTU_2020_TWIW_01_CZE_PRA_020 | Wound Pus Biopsy | ERR10436549 |
| CZE | 2020 | DTU_2020_TWIW_01_CZE_PRA_028 | Wound Pus Biopsy | ERR10441692 |
| CZE | 2020 | DTU_2020_TWIW_01_CZE_PRA_033 | Blood | ERR10436534 |
| CZE | 2020 | DTU_2020_TWIW_01_CZE_PRA_034 | Blood | ERR10436619 |
| CZE | 2020 | DTU_2020_TWIW_01_CZE_PRA_037 | Wound Pus Biopsy | ERR10436536 |
| CZE | 2020 | DTU_2020_TWIW_01_CZE_PRA_038 | Swab | ERR10436822 |
| CZE | 2020 | DTU_2020_TWIW_01_CZE_PRA_039 | Wound Pus Biopsy | ERR10436785 |
| CZE | 2020 | DTU_2020_TWIW_01_CZE_PRA_041 | Wound Pus Biopsy | ERR10436810 |
| CZE | 2020 | DTU_2020_TWIW_01_CZE_PRA_044 | Other | ERR10436643 |
| CZE | 2020 | DTU_2020_TWIW_01_CZE_PRA_045 | Blood | ERR10441685 |
| CZE | 2020 | DTU_2020_TWIW_01_CZE_PRA_047 | Urine | ERR10441713 |
| CZE | 2020 | DTU_2020_TWIW_01_CZE_PRA_049 | Other | ERR10436540 |
| CZE | 2020 | DTU_2020_TWIW_01_CZE_PRA_050 | Urine | ERR10436597 |
| CZE | 2020 | DTU_2020_TWIW_01_CZE_PRA_051 | Urine | ERR10441694 |
| CZE | 2020 | DTU_2020_TWIW_01_CZE_PRA_052 | Urine | ERR10441683 |
| CZE | 2020 | DTU_2020_TWIW_01_CZE_PRA_053 | Urine | ERR10441696 |
| CZE | 2020 | DTU_2020_TWIW_01_CZE_PRA_054 | Urine | ERR10436799 |
| CZE | 2020 | DTU_2020_TWIW_01_CZE_PRA_056 | Other | ERR10436772 |
| CZE | 2020 | DTU_2020_TWIW_01_CZE_PRA_061 | Other | ERR10436765 |
| DZA | 2020 | DTU_2020_TWIW_01_DZA_ALG_001 | Respiratory System | ERR10430239 |
| DZA | 2020 | DTU_2020_TWIW_01_DZA_ALG_002 | Other | ERR10430295 |
| DZA | 2020 | DTU_2020_TWIW_01_DZA_ALG_003 | Respiratory System | ERR10441164 |
| DZA | 2020 | DTU_2020_TWIW_01_DZA_ALG_004 | Wound Pus Biopsy | ERR10430241 |
| DZA | 2020 | DTU_2020_TWIW_01_DZA_ALG_005 | Urine | ERR10430244 |
| DZA | 2020 | DTU_2020_TWIW_01_DZA_ALG_006 | Respiratory System | ERR10430282 |
| DZA | 2020 | DTU_2020_TWIW_01_DZA_ALG_007 | Respiratory System | ERR10430292 |
| DZA | 2020 | DTU_2020_TWIW_01_DZA_ALG_008 | Wound Pus Biopsy | ERR10430802 |
| DZA | 2020 | DTU_2020_TWIW_01_DZA_ALG_009 | Wound Pus Biopsy | ERR10430304 |
| DZA | 2020 | DTU_2020_TWIW_01_DZA_ALG_011 | Wound Pus Biopsy | ERR10430302 |
| DZA | 2020 | DTU_2020_TWIW_01_DZA_ALG_012 | Wound Pus Biopsy | ERR10430307 |
| DZA | 2020 | DTU_2020_TWIW_01_DZA_ALG_013 | Urine | ERR10430279 |
| DZA | 2020 | DTU_2020_TWIW_01_DZA_ALG_014 | Urine | ERR10430729 |
| DZA | 2020 | DTU_2020_TWIW_01_DZA_ALG_015 | Wound Pus Biopsy | ERR10430293 |
| DZA | 2020 | DTU_2020_TWIW_01_DZA_ALG_016 | Wound Pus Biopsy | ERR10430287 |
| DZA | 2020 | DTU_2020_TWIW_01_DZA_ALG_017 | Wound Pus Biopsy | ERR10430722 |
| DZA | 2020 | DTU_2020_TWIW_01_DZA_ALG_018 | Urine | ERR10430285 |
| DZA | 2020 | DTU_2020_TWIW_01_DZA_ALG_019 | Urine | ERR10430300 |
| DZA | 2020 | DTU_2020_TWIW_01_DZA_ALG_020 | Urine | ERR10430297 |
| DZA | 2020 | DTU_2020_TWIW_01_DZA_ALG_021 | Wound Pus Biopsy | ERR10430298 |
| DZA | 2020 | DTU_2020_TWIW_01_DZA_ALG_022 | Blood | ERR10430291 |
| DZA | 2020 | DTU_2020_TWIW_01_DZA_ALG_023 | Wound Pus Biopsy | ERR10430288 |
| DZA | 2020 | DTU_2020_TWIW_01_DZA_ALG_024 | Urine | ERR10430289 |
| DZA | 2020 | DTU_2020_TWIW_01_DZA_ALG_025 | Urine | ERR10430290 |
| DZA | 2020 | DTU_2020_TWIW_01_DZA_ALG_026 | Wound Pus Biopsy | ERR10430245 |
| DZA | 2020 | DTU_2020_TWIW_01_DZA_ALG_027 | Urine | ERR10430299 |
| DZA | 2020 | DTU_2020_TWIW_01_DZA_ALG_029 | Urine | ERR10430286 |
| DZA | 2020 | DTU_2020_TWIW_01_DZA_ALG_030 | Urine | ERR10430281 |
| DZA | 2020 | DTU_2020_TWIW_01_DZA_ALG_031 | Blood | ERR10430280 |
| DZA | 2020 | DTU_2020_TWIW_01_DZA_ALG_032 | Other | ERR10430242 |
| DZA | 2020 | DTU_2020_TWIW_01_DZA_ALG_033 | Wound Pus Biopsy | ERR10430283 |
| DZA | 2020 | DTU_2020_TWIW_01_DZA_ALG_034 | Urine | ERR10430294 |
| NZL | 2020 | DTU_2020_TWIW_01_NZL_DUN_001 | Blood | ERR10438752 |
| NZL | 2020 | DTU_2020_TWIW_01_NZL_DUN_003 | Blood | ERR10438755 |
| NZL | 2020 | DTU_2020_TWIW_01_NZL_DUN_010 | Urine | ERR10438754 |
| NZL | 2020 | DTU_2020_TWIW_01_NZL_DUN_011 | Urine | ERR10438753 |
| NZL | 2020 | DTU_2020_TWIW_01_NZL_DUN_013 | Urine | ERR10438756 |
| NZL | 2020 | DTU_2020_TWIW_01_NZL_DUN_014 | Urine | ERR10438750 |
| NZL | 2020 | DTU_2020_TWIW_01_NZL_DUN_015 | Urine | ERR10438776 |
| NZL | 2020 | DTU_2020_TWIW_01_NZL_DUN_016 | Urine | ERR10438757 |
| NZL | 2020 | DTU_2020_TWIW_01_NZL_DUN_017 | Urine | ERR10438758 |
| NZL | 2020 | DTU_2020_TWIW_01_NZL_DUN_018 | Urine | ERR10438748 |
| NZL | 2020 | DTU_2020_TWIW_01_NZL_DUN_024 | Urine | ERR10438762 |
| NZL | 2020 | DTU_2020_TWIW_01_NZL_DUN_025 | Urine | ERR10438760 |
| NZL | 2020 | DTU_2020_TWIW_01_NZL_DUN_026 | Urine | ERR10438761 |
| NZL | 2020 | DTU_2020_TWIW_01_NZL_DUN_027 | Urine | ERR10438814 |
| NZL | 2020 | DTU_2020_TWIW_01_NZL_DUN_028 | Urine | ERR10438774 |
| NZL | 2020 | DTU_2020_TWIW_01_NZL_DUN_029 | Urine | ERR10438775 |
| NZL | 2020 | DTU_2020_TWIW_01_NZL_DUN_030 | Urine | ERR10438763 |
| NZL | 2020 | DTU_2020_TWIW_01_NZL_DUN_031 | Urine | ERR10438764 |
| NZL | 2020 | DTU_2020_TWIW_01_NZL_DUN_036 | Swab | ERR10438813 |
| NZL | 2020 | DTU_2020_TWIW_01_NZL_DUN_043 | Swab | ERR10438772 |
| NZL | 2020 | DTU_2020_TWIW_01_NZL_DUN_044 | Swab | ERR10438791 |
| NZL | 2020 | DTU_2020_TWIW_01_NZL_DUN_045 | Swab | ERR10438751 |
| NZL | 2020 | DTU_2020_TWIW_01_NZL_DUN_046 | Swab | ERR10438815 |
| NZL | 2020 | DTU_2020_TWIW_01_NZL_DUN_053 | Blood | ERR10438766 |
| NZL | 2020 | DTU_2020_TWIW_01_NZL_DUN_054 | Urine | ERR10438768 |
| NZL | 2020 | DTU_2020_TWIW_01_NZL_DUN_055 | Urine | ERR10438769 |
| NZL | 2020 | DTU_2020_TWIW_01_NZL_DUN_056 | Urine | ERR10438767 |
| NZL | 2020 | DTU_2020_TWIW_01_NZL_DUN_057 | Urine | ERR10438765 |
| NZL | 2020 | DTU_2020_TWIW_01_NZL_DUN_058 | Urine | ERR10438782 |
| NZL | 2020 | DTU_2020_TWIW_01_NZL_DUN_059 | Urine | ERR10438777 |
| NZL | 2020 | DTU_2020_TWIW_01_NZL_DUN_066 | Urine | ERR10438773 |
| NZL | 2020 | DTU_2020_TWIW_01_NZL_DUN_067 | Urine | ERR10438770 |
| NZL | 2020 | DTU_2020_TWIW_01_NZL_DUN_068 | Urine | ERR10438771 |
| NZL | 2020 | DTU_2020_TWIW_01_NZL_DUN_046A | Swab | ERR10438749 |
| COL | 2020 | DTU_2020_TWIW_01_COL_BOG_001 | Wound Pus Biopsy | ERR10441442 |
| COL | 2020 | DTU_2020_TWIW_01_COL_BOG_002 | Blood | ERR10441479 |
| COL | 2020 | DTU_2020_TWIW_01_COL_BOG_003 | Blood | ERR14129393 |
| COL | 2020 | DTU_2020_TWIW_01_COL_BOG_003 | Blood | ERR10441457 |
| COL | 2020 | DTU_2020_TWIW_01_COL_BOG_004 | Swab | ERR10441443 |
| COL | 2020 | DTU_2020_TWIW_01_COL_BOG_006 | Blood | ERR10441487 |
| COL | 2020 | DTU_2020_TWIW_01_COL_BOG_007 | Blood | ERR10441446 |
| COL | 2020 | DTU_2020_TWIW_01_COL_BOG_009 | Other | ERR10441488 |
| COL | 2020 | DTU_2020_TWIW_01_COL_BOG_011 | Swab | ERR10441449 |
| COL | 2020 | DTU_2020_TWIW_01_COL_BOG_012 | Swab | ERR10441451 |
| COL | 2020 | DTU_2020_TWIW_01_COL_BOG_013 | Urine | ERR10441504 |
| COL | 2020 | DTU_2020_TWIW_01_COL_BOG_014 | Urine | ERR10441441 |
| COL | 2020 | DTU_2020_TWIW_01_COL_BOG_015 | Blood | ERR10441502 |
| COL | 2020 | DTU_2020_TWIW_01_COL_BOG_016 | Blood | ERR10441491 |
| COL | 2020 | DTU_2020_TWIW_01_COL_BOG_017 | Blood | ERR10441490 |
| COL | 2020 | DTU_2020_TWIW_01_COL_BOG_018 | Swab | ERR10441467 |
| COL | 2020 | DTU_2020_TWIW_01_COL_BOG_020 | Blood | ERR10441484 |
| COL | 2020 | DTU_2020_TWIW_01_COL_BOG_021 | Other | ERR10441450 |
| COL | 2020 | DTU_2020_TWIW_01_COL_BOG_022 | Blood | ERR10441539 |
| COL | 2020 | DTU_2020_TWIW_01_COL_BOG_023 | Blood | ERR10441471 |
| COL | 2020 | DTU_2020_TWIW_01_COL_BOG_024 | Blood | ERR10441521 |
| COL | 2020 | DTU_2020_TWIW_01_COL_BOG_025 | Swab | ERR10441499 |
| COL | 2020 | DTU_2020_TWIW_01_COL_BOG_026 | Urine | ERR10441455 |
| COL | 2020 | DTU_2020_TWIW_01_COL_BOG_027 | Swab | ERR10441445 |
| COL | 2020 | DTU_2020_TWIW_01_COL_BOG_028 | Other | ERR10441458 |
| COL | 2020 | DTU_2020_TWIW_01_COL_BOG_029 | Other | ERR10441531 |
| COL | 2020 | DTU_2020_TWIW_01_COL_BOG_030 | Blood | ERR10441515 |
| COL | 2020 | DTU_2020_TWIW_01_COL_BOG_031 | Other | ERR10441506 |
| COL | 2020 | DTU_2020_TWIW_01_COL_BOG_032 | Urine | ERR10441507 |
| COL | 2020 | DTU_2020_TWIW_01_COL_BOG_033 | Other | ERR10441528 |
| COL | 2020 | DTU_2020_TWIW_01_COL_BOG_034 | Other | ERR10441477 |
| COL | 2020 | DTU_2020_TWIW_01_COL_BOG_035 | Other | ERR10441503 |
| COL | 2020 | DTU_2020_TWIW_01_COL_BOG_036 | Other | ERR10441456 |
| COL | 2020 | DTU_2020_TWIW_01_COL_BOG_037 | Other | ERR10441468 |
| COL | 2020 | DTU_2020_TWIW_01_COL_BOG_038 | Swab | ERR10441513 |
| COL | 2020 | DTU_2020_TWIW_01_COL_BOG_039 | Urine | ERR10441437 |
| COL | 2020 | DTU_2020_TWIW_01_COL_BOG_039 | Urine | ERR14129394 |
| COL | 2020 | DTU_2020_TWIW_01_COL_BOG_040 | Other | ERR10441474 |
| COL | 2020 | DTU_2020_TWIW_01_COL_BOG_041 | Other | ERR10441529 |
| COL | 2020 | DTU_2020_TWIW_01_COL_BOG_042 | Blood | ERR10441519 |
| COL | 2020 | DTU_2020_TWIW_01_COL_BOG_043 | Other | ERR10441526 |
| COL | 2020 | DTU_2020_TWIW_01_COL_BOG_044 | Urine | ERR10441517 |
| COL | 2020 | DTU_2020_TWIW_01_COL_BOG_045 | Wound Pus Biopsy | ERR10441486 |
| COL | 2020 | DTU_2020_TWIW_01_COL_BOG_046 | Other | ERR10441470 |
| COL | 2020 | DTU_2020_TWIW_01_COL_BOG_047 | Blood | ERR10441501 |
| COL | 2020 | DTU_2020_TWIW_01_COL_BOG_048 | Other | ERR10441473 |
| COL | 2020 | DTU_2020_TWIW_01_COL_BOG_049 | Urine | ERR10441439 |
| COL | 2020 | DTU_2020_TWIW_01_COL_BOG_050 | Other | ERR10441557 |
| COL | 2020 | DTU_2020_TWIW_01_COL_BOG_051 | Blood | ERR10441525 |
| COL | 2020 | DTU_2020_TWIW_01_COL_BOG_052 | Blood | ERR10441518 |
| COL | 2020 | DTU_2020_TWIW_01_COL_BOG_053 | Swab | ERR10441454 |
| COL | 2020 | DTU_2020_TWIW_01_COL_BOG_054 | Wound Pus Biopsy | ERR10441448 |
| COL | 2020 | DTU_2020_TWIW_01_COL_BOG_055 | Swab | ERR10441453 |
| COL | 2020 | DTU_2020_TWIW_01_COL_BOG_056 | Blood | ERR10441500 |
| COL | 2020 | DTU_2020_TWIW_01_COL_BOG_057 | Other | ERR10441532 |
| COL | 2020 | DTU_2020_TWIW_01_COL_BOG_058 | Blood | ERR10441527 |
| COL | 2020 | DTU_2020_TWIW_01_COL_BOG_059 | Blood | ERR10441489 |
| COL | 2020 | DTU_2020_TWIW_01_COL_BOG_060 | Other | ERR10441459 |
| ECU | 2020 | DTU_2020_TWIW_01_ECU_QUI_CAMH_001 | Other | ERR10437168 |
| ECU | 2020 | DTU_2020_TWIW_01_ECU_QUITO_CAMH_002 | Respiratory System | ERR10437268 |
| ECU | 2020 | DTU_2020_TWIW_01_ECU_QUITO_CAMH_003 | Urine | ERR10437196 |
| ECU | 2020 | DTU_2020_TWIW_01_ECU_QUITO_CAMH_004 | Other | ERR10437258 |
| ECU | 2020 | DTU_2020_TWIW_01_ECU_QUITO_CAMH_005 | Respiratory System | ERR10437267 |
| ECU | 2020 | DTU_2020_TWIW_01_ECU_QUITO_CAMH_006 | Wound Pus Biopsy | ERR10437141 |
| ECU | 2020 | DTU_2020_TWIW_01_ECU_QUITO_CAMH_007 | Respiratory System | ERR10437312 |
| ECU | 2020 | DTU_2020_TWIW_01_ECU_QUITO_CAMH_008 | Respiratory System | ERR10437254 |
| ECU | 2020 | DTU_2020_TWIW_01_ECU_QUITO_CAMH_009 | Wound Pus Biopsy | ERR10437319 |
| ECU | 2020 | DTU_2020_TWIW_01_ECU_QUITO_CAMH_010 | Respiratory System | ERR10437323 |
| ECU | 2020 | DTU_2020_TWIW_01_ECU_QUITO_CAMH_011 | Respiratory System | ERR10437338 |
| ECU | 2020 | DTU_2020_TWIW_01_ECU_QUITO_CAMH_012 | Respiratory System | ERR10437160 |
| ECU | 2020 | DTU_2020_TWIW_01_ECU_QUITO_CAMH_013 | Blood | ERR10437065 |
| ECU | 2020 | DTU_2020_TWIW_01_ECU_QUITO_CAMH_014 | Blood | ERR10437269 |
| ECU | 2020 | DTU_2020_TWIW_01_ECU_QUITO_CAMH_015 | Blood | ERR10437324 |
| ECU | 2020 | DTU_2020_TWIW_01_ECU_QUITO_CAMH_016 | Urine | ERR10437213 |
| ECU | 2020 | DTU_2020_TWIW_01_ECU_QUITO_CAMH_017 | Respiratory System | ERR10437193 |
| ECU | 2020 | DTU_2020_TWIW_01_ECU_QUITO_CAMH_018 | Other | ERR10437276 |
| ECU | 2020 | DTU_2020_TWIW_01_ECU_QUITO_CAMH_019 | Urine | ERR10437194 |
| ECU | 2020 | DTU_2020_TWIW_01_ECU_QUITO_CAMH_020 | Respiratory System | ERR10437192 |
| ECU | 2020 | DTU_2020_TWIW_01_ECU_QUITO_CAMH_021 | Respiratory System | ERR10437201 |
| ECU | 2020 | DTU_2020_TWIW_01_ECU_QUITO_CAMH_022 | Respiratory System | ERR10437161 |
| ECU | 2020 | DTU_2020_TWIW_01_ECU_QUITO_CAMH_023 | Swab | ERR10437106 |
| ECU | 2020 | DTU_2020_TWIW_01_ECU_QUITO_HDR_001 | Blood | ERR10437162 |
| ECU | 2020 | DTU_2020_TWIW_01_ECU_QUITO_HDR_002 | Blood | ERR10437163 |
| ECU | 2020 | DTU_2020_TWIW_01_ECU_QUITO_HDR_003 | Blood | ERR10437140 |
| ECU | 2020 | DTU_2020_TWIW_01_ECU_QUITO_HDR_004 | Blood | ERR10437301 |
| ECU | 2020 | DTU_2020_TWIW_01_ECU_QUITO_HDR_005 | Blood | ERR10437121 |
| ECU | 2020 | DTU_2020_TWIW_01_ECU_QUITO_HDR_006 | Blood | ERR10437175 |
| ECU | 2020 | DTU_2020_TWIW_01_ECU_QUITO_HDR_007 | Blood | ERR10437138 |
| ECU | 2020 | DTU_2020_TWIW_01_ECU_QUITO_HDR_008 | Blood | ERR10437127 |
| ECU | 2020 | DTU_2020_TWIW_01_ECU_QUITO_HDR_009 | Blood | ERR10437302 |
| ECU | 2020 | DTU_2020_TWIW_01_ECU_QUITO_HDR_010 | Other | ERR10437314 |
| ECU | 2020 | DTU_2020_TWIW_01_ECU_QUITO_EEH_001 | Respiratory System | ERR10437083 |
| ECU | 2020 | DTU_2020_TWIW_01_ECU_QUITO_EEH_002 | Respiratory System | ERR10437181 |
| ECU | 2020 | DTU_2020_TWIW_01_ECU_QUITO_EEH_003 | Respiratory System | ERR10437152 |
| ECU | 2020 | DTU_2020_TWIW_01_ECU_QUITO_EEH_004 | Other | ERR10437358 |
| ECU | 2020 | DTU_2020_TWIW_01_ECU_QUITO_EEH_005 | Blood | ERR10437331 |
| ECU | 2020 | DTU_2020_TWIW_01_ECU_QUITO_EEH_006 | Blood | ERR10437085 |
| ECU | 2020 | DTU_2020_TWIW_01_ECU_QUITO_EEH_007 | Respiratory System | ERR10437084 |
| ECU | 2020 | DTU_2020_TWIW_01_ECU_QUITO_EEH_008 | Respiratory System | ERR10437119 |
| ECU | 2020 | DTU_2020_TWIW_01_ECU_QUITO_EEH_009 | Respiratory System | ERR10437363 |
| ECU | 2020 | DTU_2020_TWIW_01_ECU_QUITO_EEH_010 | Blood | ERR10437235 |
| ECU | 2020 | DTU_2020_TWIW_01_ECU_QUITO_EEH_011 | Blood | ERR10437365 |
| ECU | 2020 | DTU_2020_TWIW_01_ECU_QUITO_EEH_012 | Blood | ERR10437094 |
| ECU | 2020 | DTU_2020_TWIW_01_ECU_QUITO_EEH_013 | Respiratory System | ERR10437086 |
| ECU | 2020 | DTU_2020_TWIW_01_ECU_QUITO_EEH_014 | Respiratory System | ERR10437099 |
| ECU | 2020 | DTU_2020_TWIW_01_ECU_QUITO_EEH_015 | Urine | ERR10437335 |
| ECU | 2020 | DTU_2020_TWIW_01_ECU_QUITO_EEH_016 | Respiratory System | ERR10437364 |
| ECU | 2020 | DTU_2020_TWIW_01_ECU_QUITO_EEH_017 | Blood | ERR10437266 |
| ECU | 2020 | DTU_2020_TWIW_01_ECU_QUITO_EEH_018 | Blood | ERR10437351 |
| ECU | 2020 | DTU_2020_TWIW_01_ECU_QUITO_EEH_019 | Blood | ERR10437337 |
| ECU | 2020 | DTU_2020_TWIW_01_ECU_QUITO_EEH_020 | Respiratory System | ERR10437124 |
| ECU | 2020 | DTU_2020_TWIW_01_ECU_QUITO_EEH_021 | Respiratory System | ERR10437353 |
| ECU | 2020 | DTU_2020_TWIW_01_ECU_QUITO_EEH_022 | Respiratory System | ERR10437357 |
| ECU | 2020 | DTU_2020_TWIW_01_ECU_QUITO_EEH_023 | Respiratory System | ERR10437352 |
| ECU | 2020 | DTU_2020_TWIW_01_ECU_GUA_001 | Respiratory System | ERR10437299 |
| ECU | 2020 | DTU_2020_TWIW_01_ECU_GUA_002 | Blood | ERR10437211 |
| ECU | 2020 | DTU_2020_TWIW_01_ECU_GUA_003 | Respiratory System | ERR10437223 |
| ECU | 2020 | DTU_2020_TWIW_01_ECU_GUA_004 | Other | ERR10437233 |
| ECU | 2020 | DTU_2020_TWIW_01_ECU_GUA_006 | Urine | ERR10437231 |
| ECU | 2020 | DTU_2020_TWIW_01_ECU_GUA_007 | Other | ERR10437142 |
| ECU | 2020 | DTU_2020_TWIW_01_ECU_GUA_008 | Other | ERR10437064 |
| ECU | 2020 | DTU_2020_TWIW_01_ECU_GUA_009 | Blood | ERR10437234 |
| ECU | 2020 | DTU_2020_TWIW_01_ECU_GUA_010 | Other | ERR10437245 |
| ECU | 2020 | DTU_2020_TWIW_01_ECU_GUA_011 | Respiratory System | ERR10437243 |
| ECU | 2020 | DTU_2020_TWIW_01_ECU_GUA_012 | Respiratory System | ERR10437246 |
| ECU | 2020 | DTU_2020_TWIW_01_ECU_GUA_013 | Blood | ERR10437259 |
| TUR | 2020 | DTU_2020_TWIW_01_TUR_ORT_002 | Blood | ERR10432725 |
| TUR | 2020 | DTU_2020_TWIW_01_TUR_ORT_003 | Wound Pus Biopsy | ERR10432740 |
| TUR | 2020 | DTU_2020_TWIW_01_TUR_ORT_004 | Urine | ERR10432719 |
| TUR | 2020 | DTU_2020_TWIW_01_TUR_ORT_007 | Blood | ERR10432736 |
| TUR | 2020 | DTU_2020_TWIW_01_TUR_ORT_008 | Blood | ERR10432743 |
| TUR | 2020 | DTU_2020_TWIW_01_TUR_ORT_011 | Blood | ERR10432717 |
| TUR | 2020 | DTU_2020_TWIW_01_TUR_ORT_012 | Urine | ERR10432738 |
| TUR | 2020 | DTU_2020_TWIW_01_TUR_ORT_014 | Urine | ERR10432729 |
| TUR | 2020 | DTU_2020_TWIW_01_TUR_ORT_015 | Wound Pus Biopsy | ERR10432722 |
| TUR | 2020 | DTU_2020_TWIW_01_TUR_ORT_016 | Respiratory System | ERR10432726 |
| TUR | 2020 | DTU_2020_TWIW_01_TUR_ORT_018 | Urine | ERR10432741 |
| TUR | 2020 | DTU_2020_TWIW_01_TUR_ORT_019 | Urine | ERR10432742 |
| TUR | 2020 | DTU_2020_TWIW_01_TUR_ORT_020 | Respiratory System | ERR10432763 |
| TUR | 2020 | DTU_2020_TWIW_01_TUR_ORT_021 | Respiratory System | ERR10432724 |
| TUR | 2020 | DTU_2020_TWIW_01_TUR_ORT_024 | Wound Pus Biopsy | ERR10432757 |
| TUR | 2020 | DTU_2020_TWIW_01_TUR_ORT_026 | Urine | ERR10432748 |
| TUR | 2020 | DTU_2020_TWIW_01_TUR_ORT_027 | Wound Pus Biopsy | ERR10432780 |
| TUR | 2020 | DTU_2020_TWIW_01_TUR_ORT_029 | Urine | ERR10432712 |
| TUR | 2020 | DTU_2020_TWIW_01_TUR_ORT_031 | Urine | ERR10432779 |
| TUR | 2020 | DTU_2020_TWIW_01_TUR_ORT_032 | Urine | ERR10432730 |
| TUR | 2020 | DTU_2020_TWIW_01_TUR_ORT_033 | Respiratory System | ERR10432721 |
| TUR | 2020 | DTU_2020_TWIW_01_TUR_ORT_034 | Urine | ERR10432727 |
| TUR | 2020 | DTU_2020_TWIW_01_TUR_ORT_038 | Urine | ERR10432739 |
| TUR | 2020 | DTU_2020_TWIW_01_TUR_ORT_040 | Urine | ERR10432737 |
| TUR | 2020 | DTU_2020_TWIW_01_TUR_ORT_041 | Urine | ERR10432752 |
| TUR | 2020 | DTU_2020_TWIW_01_TUR_ORT_044 | Respiratory System | ERR10432728 |
| TUR | 2020 | DTU_2020_TWIW_01_TUR_ORT_045 | Urine | ERR10432749 |
| TUR | 2020 | DTU_2020_TWIW_01_TUR_ORT_046 | Urine | ERR10432746 |
| TUR | 2020 | DTU_2020_TWIW_01_TUR_ORT_047 | Wound Pus Biopsy | ERR10436371 |
| TUR | 2020 | DTU_2020_TWIW_01_TUR_ORT_048 | Other | ERR10432778 |
| TUR | 2020 | DTU_2020_TWIW_01_TUR_ORT_050 | Urine | ERR10432716 |
| TUR | 2020 | DTU_2020_TWIW_01_TUR_ORT_051 | Urine | ERR10432777 |
| TUR | 2020 | DTU_2020_TWIW_01_TUR_ORT_052 | Respiratory System | ERR10432732 |
| TUR | 2020 | DTU_2020_TWIW_01_TUR_ORT_053 | Urine | ERR10432809 |
| TUR | 2020 | DTU_2020_TWIW_01_TUR_ORT_054 | Urine | ERR10432744 |
| TUR | 2020 | DTU_2020_TWIW_01_TUR_ORT_056 | Urine | ERR10432714 |
| TUR | 2020 | DTU_2020_TWIW_01_TUR_ORT_057 | Respiratory System | ERR10439773 |
| TUR | 2020 | DTU_2020_TWIW_01_TUR_ORT_058 | Urine | ERR10432764 |
| TUR | 2020 | DTU_2020_TWIW_01_TUR_ORT_059 | Wound Pus Biopsy | ERR10432753 |
| TUR | 2020 | DTU_2020_TWIW_01_TUR_ORT_060 | Wound Pus Biopsy | ERR10432759 |
| TUR | 2020 | DTU_2020_TWIW_01_TUR_ORT_061 | Urine | ERR10432734 |
| TUR | 2020 | DTU_2020_TWIW_01_TUR_ORT_062 | Wound Pus Biopsy | ERR10432771 |
| TUR | 2020 | DTU_2020_TWIW_01_TUR_ORT_063 | Respiratory System | ERR10432750 |
| TUR | 2020 | DTU_2020_TWIW_01_TUR_ORT_064 | Urine | ERR10432747 |
| TUR | 2020 | DTU_2020_TWIW_01_TUR_ORT_065 | Blood | ERR10432767 |
| NGA | 2020 | DTU_2020_TWIW_01_NGA_IBADAN_001 | Blood | ERR10439023 |
| NGA | 2020 | DTU_2020_TWIW_01_NGA_IBADAN_002 | Wound Pus Biopsy | ERR10438970 |
| NGA | 2020 | DTU_2020_TWIW_01_NGA_IBADAN_003 | Wound Pus Biopsy | ERR10439044 |
| NGA | 2020 | DTU_2020_TWIW_01_NGA_IBADAN_004 | Wound Pus Biopsy | ERR10438821 |
| NGA | 2020 | DTU_2020_TWIW_01_NGA_IBADAN_005 | Wound Pus Biopsy | ERR10438971 |
| NGA | 2020 | DTU_2020_TWIW_01_NGA_IBADAN_006 | Urine | ERR10439026 |
| NGA | 2020 | DTU_2020_TWIW_01_NGA_IBADAN_007 | Respiratory System | ERR10438919 |
| NGA | 2020 | DTU_2020_TWIW_01_NGA_IBADAN_008 | Respiratory System | ERR10438922 |
| NGA | 2020 | DTU_2020_TWIW_01_NGA_IBADAN_009 | Wound Pus Biopsy | ERR10438962 |
| NGA | 2020 | DTU_2020_TWIW_01_NGA_IBADAN_010 | Urine | ERR10438860 |
| NGA | 2020 | DTU_2020_TWIW_01_NGA_IBADAN_011 | Urine | ERR10438920 |
| NGA | 2020 | DTU_2020_TWIW_01_NGA_IBADAN_012 | Other | ERR10438973 |
| NGA | 2020 | DTU_2020_TWIW_01_NGA_IBADAN_013 | Wound Pus Biopsy | ERR10439018 |
| NGA | 2020 | DTU_2020_TWIW_01_NGA_IBADAN_014 | Urine | ERR10438921 |
| NGA | 2020 | DTU_2020_TWIW_01_NGA_IBADAN_015 | Other | ERR10439043 |
| NGA | 2020 | DTU_2020_TWIW_01_NGA_IBADAN_016 | Other | ERR10439015 |
| NGA | 2020 | DTU_2020_TWIW_01_NGA_IBADAN_017 | Urine | ERR10438819 |
| NGA | 2020 | DTU_2020_TWIW_01_NGA_IBADAN_018 | Urine | ERR10438820 |
| NGA | 2020 | DTU_2020_TWIW_01_NGA_IBADAN_019 | Swab | ERR10438936 |
| NGA | 2020 | DTU_2020_TWIW_01_NGA_IBADAN_020 | Swab | ERR10438972 |
| NGA | 2020 | DTU_2020_TWIW_01_NGA_IBADAN_022 | Wound Pus Biopsy | ERR10438818 |
| NGA | 2020 | DTU_2020_TWIW_01_NGA_IBADAN_023 | Urine | ERR10438867 |
| NGA | 2020 | DTU_2020_TWIW_01_NGA_IBADAN_024 | Wound Pus Biopsy | ERR10438824 |
| NGA | 2020 | DTU_2020_TWIW_01_NGA_IBADAN_025 | Urine | ERR10438863 |
| NGA | 2020 | DTU_2020_TWIW_01_NGA_IBADAN_026 | Urine | ERR10438833 |
| NGA | 2020 | DTU_2020_TWIW_01_NGA_IBADAN_027 | Swab | ERR10438933 |
| NGA | 2020 | DTU_2020_TWIW_01_NGA_IBADAN_028 | Swab | ERR10439045 |
| NGA | 2020 | DTU_2020_TWIW_01_NGA_IBADAN_029 | Urine | ERR10438905 |
| NGA | 2020 | DTU_2020_TWIW_01_NGA_IBADAN_030 | Other | ERR10446211 |
| NGA | 2020 | DTU_2020_TWIW_01_NGA_IBADAN_031 | Urine | ERR10438866 |
| NGA | 2020 | DTU_2020_TWIW_01_NGA_IBADAN_032 | Urine | ERR10438869 |
| NGA | 2020 | DTU_2020_TWIW_01_NGA_IBADAN_033 | Urine | ERR10438868 |
| NGA | 2020 | DTU_2020_TWIW_01_NGA_IBADAN_034 | Urine | ERR10438872 |
| NGA | 2020 | DTU_2020_TWIW_01_NGA_IBADAN_035 | Other | ERR10438876 |
| NGA | 2020 | DTU_2020_TWIW_01_NGA_IBADAN_036 | Urine | ERR10438875 |
| NGA | 2020 | DTU_2020_TWIW_01_NGA_IBADAN_037 | Urine | ERR10446206 |
| NGA | 2020 | DTU_2020_TWIW_01_NGA_IBADAN_037 | Urine | ERR12041390 |
| NGA | 2020 | DTU_2020_TWIW_01_NGA_IBADAN_038 | Urine | ERR10438950 |
| NGA | 2020 | DTU_2020_TWIW_01_NGA_IBADAN_039 | Wound Pus Biopsy | ERR10438975 |
| NGA | 2020 | DTU_2020_TWIW_01_NGA_IBADAN_040 | Urine | ERR10439054 |
| NGA | 2020 | DTU_2020_TWIW_01_NGA_IBADAN_041 | Urine | ERR10438825 |
| NGA | 2020 | DTU_2020_TWIW_01_NGA_IBADAN_042 | Urine | ERR10438873 |
| NGA | 2020 | DTU_2020_TWIW_01_NGA_IBADAN_043 | Wound Pus Biopsy | ERR10438977 |
| NGA | 2020 | DTU_2020_TWIW_01_NGA_IBADAN_044 | Other | ERR10438935 |
| NGA | 2020 | DTU_2020_TWIW_01_NGA_IBADAN_045 | Other | ERR10438829 |
| NGA | 2020 | DTU_2020_TWIW_01_NGA_IBADAN_046 | Urine | ERR10439046 |
| NGA | 2020 | DTU_2020_TWIW_01_NGA_IBADAN_047 | Respiratory System | ERR10438947 |
| NGA | 2020 | DTU_2020_TWIW_01_NGA_IBADAN_048 | Urine | ERR10438887 |
| NGA | 2020 | DTU_2020_TWIW_01_NGA_IBADAN_049 | Respiratory System | ERR10438949 |
| NGA | 2020 | DTU_2020_TWIW_01_NGA_IBADAN_050 | Wound Pus Biopsy | ERR10438837 |
| NGA | 2020 | DTU_2020_TWIW_01_NGA_IBADAN_051 | Wound Pus Biopsy | ERR10438937 |
| NGA | 2020 | DTU_2020_TWIW_01_NGA_IBADAN_052 | Wound Pus Biopsy | ERR10438979 |
| NGA | 2020 | DTU_2020_TWIW_01_NGA_IBADAN_053 | Wound Pus Biopsy | ERR10438830 |
| NGA | 2020 | DTU_2020_TWIW_01_NGA_IBADAN_054 | Urine | ERR10438831 |
| NGA | 2020 | DTU_2020_TWIW_01_NGA_IBADAN_055 | Swab | ERR10438980 |
| NGA | 2020 | DTU_2020_TWIW_01_NGA_IBADAN_056 | Urine | ERR10438894 |
| NGA | 2020 | DTU_2020_TWIW_01_NGA_IBADAN_057 | Wound Pus Biopsy | ERR10438826 |
| NGA | 2020 | DTU_2020_TWIW_01_NGA_IBADAN_058 | Wound Pus Biopsy | ERR10438976 |
| NGA | 2020 | DTU_2020_TWIW_01_NGA_IBADAN_059 | Wound Pus Biopsy | ERR10439050 |
| NGA | 2020 | DTU_2020_TWIW_01_NGA_IBADAN_060 | Wound Pus Biopsy | ERR10438895 |
| TUR | 2020 | DTU_2020_TWIW_01_TUR_ORT_001 | Respiratory System | ERR10432775 |
| NGA | 2020 | DTU_2020_TWIW_01_NGA_IBADAN_048A | Urine | ERR10446209 |
| ZMB | 2020 | DTU_2020_TWIW_01_ZMB_LUS_UTH_001 | Urine | ERR10432808 |
| ZMB | 2020 | DTU_2020_TWIW_01_ZMB_LUS_UTH_002 | Wound Pus Biopsy | ERR10432861 |
| ZMB | 2020 | DTU_2020_TWIW_01_ZMB_LUS_UTH_003 | Urine | ERR10432870 |
| ZMB | 2020 | DTU_2020_TWIW_01_ZMB_LUS_UTH_004 | Wound Pus Biopsy | ERR10432854 |
| ZMB | 2020 | DTU_2020_TWIW_01_ZMB_LUS_UTH_005 | Blood | ERR10432812 |
| ZMB | 2020 | DTU_2020_TWIW_01_ZMB_LUS_UTH_006 | Blood | ERR10432783 |
| ZMB | 2020 | DTU_2020_TWIW_01_ZMB_LUS_UTH_007 | Blood | ERR10432871 |
| ZMB | 2020 | DTU_2020_TWIW_01_ZMB_LUS_UTH_008 | Blood | ERR10432865 |
| ZMB | 2020 | DTU_2020_TWIW_01_ZMB_LUS_UTH_009 | Blood | ERR10432839 |
| ZMB | 2020 | DTU_2020_TWIW_01_ZMB_LUS_UTH_010 | Blood | ERR10432807 |
| ZMB | 2020 | DTU_2020_TWIW_01_ZMB_LUS_UTH_011 | Blood | ERR10432856 |
| ZMB | 2020 | DTU_2020_TWIW_01_ZMB_LUS_UTH_012 | Blood | ERR10432785 |
| ZMB | 2020 | DTU_2020_TWIW_01_ZMB_LUS_UTH_013 | Blood | ERR10432832 |
| ZMB | 2020 | DTU_2020_TWIW_01_ZMB_LUS_UTH_014 | Blood | ERR10432872 |
| ZMB | 2020 | DTU_2020_TWIW_01_ZMB_LUS_UTH_015 | Wound Pus Biopsy | ERR10432834 |
| ZMB | 2020 | DTU_2020_TWIW_01_ZMB_LUS_UTH_016 | Wound Pus Biopsy | ERR10432837 |
| ZMB | 2020 | DTU_2020_TWIW_01_ZMB_LUS_UTH_017 | Wound Pus Biopsy | ERR10432794 |
| ZMB | 2020 | DTU_2020_TWIW_01_ZMB_LUS_UTH_018 | Wound Pus Biopsy | ERR10432810 |
| ZMB | 2020 | DTU_2020_TWIW_01_ZMB_LUS_UTH_019 | Wound Pus Biopsy | ERR10432791 |
| ZMB | 2020 | DTU_2020_TWIW_01_ZMB_LUS_UTH_020 | Wound Pus Biopsy | ERR10432805 |
| ZMB | 2020 | DTU_2020_TWIW_01_ZMB_LUS_UTH_022 | Wound Pus Biopsy | ERR10432836 |
| ZMB | 2020 | DTU_2020_TWIW_01_ZMB_LUS_UTH_023 | Wound Pus Biopsy | ERR10432789 |
| ZMB | 2020 | DTU_2020_TWIW_01_ZMB_LUS_UTH_024 | Wound Pus Biopsy | ERR10432831 |
| ZMB | 2020 | DTU_2020_TWIW_01_ZMB_LUS_UTH_025 | Urine | ERR10446205 |
| ZMB | 2020 | DTU_2020_TWIW_01_ZMB_LUS_UTH_026 | Urine | ERR10432806 |
| ZMB | 2020 | DTU_2020_TWIW_01_ZMB_LUS_UTH_027 | Urine | ERR10432793 |
| ZMB | 2020 | DTU_2020_TWIW_01_ZMB_LUS_UTH_028 | Urine | ERR10432866 |
| ZMB | 2020 | DTU_2020_TWIW_01_ZMB_LUS_UTH_029 | Urine | ERR10432798 |
| ZMB | 2020 | DTU_2020_TWIW_01_ZMB_LUS_UTH_030 | Urine | ERR10432784 |
| ZMB | 2020 | DTU_2020_TWIW_01_ZMB_LUS_UTH_031 | Urine | ERR10432811 |
| ZMB | 2020 | DTU_2020_TWIW_01_ZMB_LUS_UTH_032 | Wound Pus Biopsy | ERR10432841 |
| ZMB | 2020 | DTU_2020_TWIW_01_ZMB_LUS_UTH_033 | Wound Pus Biopsy | ERR10432843 |
| ZMB | 2020 | DTU_2020_TWIW_01_ZMB_LUS_UTH_034 | Wound Pus Biopsy | ERR10432846 |
| ZMB | 2020 | DTU_2020_TWIW_01_ZMB_LUS_UTH_035 | Blood | ERR10432804 |
| ZMB | 2020 | DTU_2020_TWIW_01_ZMB_LUS_UTH_036 | Blood | ERR10432842 |
| ZMB | 2020 | DTU_2020_TWIW_01_ZMB_LUS_UTH_037 | Blood | ERR10432828 |
| ZMB | 2020 | DTU_2020_TWIW_01_ZMB_LUS_UTH_038 | Blood | ERR10432852 |
| ZMB | 2020 | DTU_2020_TWIW_01_ZMB_LUS_UTH_039 | Blood | ERR10432855 |
| ZMB | 2020 | DTU_2020_TWIW_01_ZMB_LUS_UTH_040 | Blood | ERR10432788 |
| ZMB | 2020 | DTU_2020_TWIW_01_ZMB_LUS_UTH_041 | Blood | ERR10446207 |
| ZMB | 2020 | DTU_2020_TWIW_01_ZMB_LUS_UTH_042 | Blood | ERR10432815 |
| ZMB | 2020 | DTU_2020_TWIW_01_ZMB_LUS_UTH_043 | Urine | ERR10432850 |
| ZMB | 2020 | DTU_2020_TWIW_01_ZMB_LUS_UTH_044 | Urine | ERR10432799 |
| ZMB | 2020 | DTU_2020_TWIW_01_ZMB_LUS_UTH_045 | Urine | ERR10432790 |
| ZMB | 2020 | DTU_2020_TWIW_01_ZMB_LUS_UTH_046 | Urine | ERR10432827 |
| ZMB | 2020 | DTU_2020_TWIW_01_ZMB_LUS_UTH_047 | Urine | ERR10432796 |
| ZMB | 2020 | DTU_2020_TWIW_01_ZMB_LUS_UTH_048 | Urine | ERR10432830 |
| ZMB | 2020 | DTU_2020_TWIW_01_ZMB_LUS_UTH_049 | Urine | ERR10432833 |
| ZMB | 2020 | DTU_2020_TWIW_01_ZMB_LUS_UTH_050 | Other | ERR10432782 |
| ZMB | 2020 | DTU_2020_TWIW_01_ZMB_LUS_UTH_051 | Wound Pus Biopsy | ERR10432802 |
| ZMB | 2020 | DTU_2020_TWIW_01_ZMB_LUS_UTH_052 | Wound Pus Biopsy | ERR10432859 |
| ZMB | 2020 | DTU_2020_TWIW_01_ZMB_LUS_UTH_053 | Wound Pus Biopsy | ERR10432803 |
| ZMB | 2020 | DTU_2020_TWIW_01_ZMB_LUS_UTH_054 | Wound Pus Biopsy | ERR10432824 |
| ZMB | 2020 | DTU_2020_TWIW_01_ZMB_LUS_UTH_055 | Wound Pus Biopsy | ERR10432868 |
| ZMB | 2020 | DTU_2020_TWIW_01_ZMB_LUS_UTH_056 | Wound Pus Biopsy | ERR10432857 |
| ZMB | 2020 | DTU_2020_TWIW_01_ZMB_LUS_UTH_057 | Wound Pus Biopsy | ERR10432835 |
| ZMB | 2020 | DTU_2020_TWIW_01_ZMB_LUS_UTH_058 | Blood | ERR10432847 |
| ZMB | 2020 | DTU_2020_TWIW_01_ZMB_LUS_UTH_059 | Urine | ERR10432819 |
| ZMB | 2020 | DTU_2020_TWIW_01_ZMB_LUS_UTH_060 | Urine | ERR10432814 |
| ZMB | 2020 | DTU_2020_TWIW_01_ZMB_LUS_UTH_020A | Wound Pus Biopsy | ERR10502525 |
| ZMB | 2020 | DTU_2020_TWIW_01_ZMB_LUS_LMTH_001 | Blood | ERR10432816 |
| ZMB | 2020 | DTU_2020_TWIW_01_ZMB_LUS_LMTH_002 | Urine | ERR10432786 |
| ZMB | 2020 | DTU_2020_TWIW_01_ZMB_LUS_LMTH_003 | Urine | ERR10432813 |
| ZMB | 2020 | DTU_2020_TWIW_01_ZMB_LUS_LMTH_004 | Other | ERR10432853 |
| ZMB | 2020 | DTU_2020_TWIW_01_ZMB_LUS_LMTH_005 | Wound Pus Biopsy | ERR10432862 |
| ZMB | 2020 | DTU_2020_TWIW_01_ZMB_LUS_LMTH_006 | Wound Pus Biopsy | ERR10432838 |
| ZMB | 2020 | DTU_2020_TWIW_01_ZMB_LUS_LMTH_007 | Urine | ERR10432826 |
| ZMB | 2020 | DTU_2020_TWIW_01_ZMB_LUS_LMTH_008 | Urine | ERR10432792 |
| ZMB | 2020 | DTU_2020_TWIW_01_ZMB_LUS_LMTH_009 | Urine | ERR10432800 |
| ZMB | 2020 | DTU_2020_TWIW_01_ZMB_LUS_LMTH_010 | Urine | ERR10432801 |
| ZMB | 2020 | DTU_2020_TWIW_01_ZMB_LUS_LMTH_011 | Wound Pus Biopsy | ERR10432864 |
| ZMB | 2020 | DTU_2020_TWIW_01_ZMB_LUS_LMTH_012 | Swab | ERR10432858 |
| ZMB | 2020 | DTU_2020_TWIW_01_ZMB_LUS_LMTH_013 | Respiratory System | ERR10432863 |
| ZMB | 2020 | DTU_2020_TWIW_01_ZMB_LUS_LMTH_014 | Urine | ERR10432795 |
| ZMB | 2020 | DTU_2020_TWIW_01_ZMB_LUS_LMTH_015 | Urine | ERR10432822 |
| ZMB | 2020 | DTU_2020_TWIW_01_ZMB_LUS_LMTH_016 | Urine | ERR10432840 |
| ZMB | 2020 | DTU_2020_TWIW_01_ZMB_LUS_LMTH_017 | Wound Pus Biopsy | ERR10432823 |
| ZMB | 2020 | DTU_2020_TWIW_01_ZMB_LUS_LMTH_018 | Urine | ERR10432848 |
| ZMB | 2020 | DTU_2020_TWIW_01_ZMB_LUS_LMTH_019 | Other | ERR10432845 |
| ZMB | 2020 | DTU_2020_TWIW_01_ZMB_LUS_LMTH_020 | Wound Pus Biopsy | ERR10432851 |
| ZMB | 2020 | DTU_2020_TWIW_01_ZMB_LUS_LMTH_021 | Urine | ERR10432817 |
| ZMB | 2020 | DTU_2020_TWIW_01_ZMB_LUS_LMTH_022 | Urine | ERR10432818 |
| ZMB | 2020 | DTU_2020_TWIW_01_ZMB_LUS_LMTH_023 | Wound Pus Biopsy | ERR10432825 |
| ZMB | 2020 | DTU_2020_TWIW_01_ZMB_LUS_UTH_061 | Urine | ERR10432867 |
| ZMB | 2020 | DTU_2020_TWIW_01_ZMB_LUS_UTH_062 | Urine | ERR10432821 |
| ZMB | 2020 | DTU_2020_TWIW_01_ZMB_LUS_UTH_063 | Urine | ERR10432829 |
| PRY | 2020 | DTU_2020_TWIW_01_PRY_ASU_LCPMC_001 | Wound Pus Biopsy | ERR10431949 |
| PRY | 2020 | DTU_2020_TWIW_01_PRY_ASU_LCPMC_002 | Urine | ERR10431714 |
| PRY | 2020 | DTU_2020_TWIW_01_PRY_ASU_LCPMC_003 | Urine | ERR10431855 |
| PRY | 2020 | DTU_2020_TWIW_01_PRY_ASU_LCPMC_004 | Other | ERR10431954 |
| PRY | 2020 | DTU_2020_TWIW_01_PRY_ASU_LCPMC_005 | Swab | ERR10431712 |
| PRY | 2020 | DTU_2020_TWIW_01_PRY_ASU_LCPMC_006 | Urine | ERR10431719 |
| PRY | 2020 | DTU_2020_TWIW_01_PRY_ASU_LCPMC_007 | Urine | ERR10446203 |
| PRY | 2020 | DTU_2020_TWIW_01_PRY_ASU_LCPMC_008 | Urine | ERR10432329 |
| PRY | 2020 | DTU_2020_TWIW_01_PRY_ASU_LCPMC_009 | Urine | ERR10431872 |
| PRY | 2020 | DTU_2020_TWIW_01_PRY_ASU_LCPMC_010 | Wound Pus Biopsy | ERR10431948 |
| PRY | 2020 | DTU_2020_TWIW_01_PRY_ASU_LCPMC_011 | Wound Pus Biopsy | ERR10431629 |
| PRY | 2020 | DTU_2020_TWIW_01_PRY_ASU_LCPMC_012 | Urine | ERR10431746 |
| PRY | 2020 | DTU_2020_TWIW_01_PRY_ASU_LCPMC_013 | Wound Pus Biopsy | ERR10431730 |
| PRY | 2020 | DTU_2020_TWIW_01_PRY_ASU_LCPMC_013 | Wound Pus Biopsy | ERR12075677 |
| PRY | 2020 | DTU_2020_TWIW_01_PRY_ASU_LCPMC_014 | Wound Pus Biopsy | ERR10431969 |
| PRY | 2020 | DTU_2020_TWIW_01_PRY_ASU_LCPMC_015 | Urine | ERR10431800 |
| PRY | 2020 | DTU_2020_TWIW_01_PRY_ASU_LCPMC_016 | Urine | ERR10431764 |
| PRY | 2020 | DTU_2020_TWIW_01_PRY_ASU_LCPMC_017 | Urine | ERR10431765 |
| PRY | 2020 | DTU_2020_TWIW_01_PRY_ASU_LCPMC_018 | Urine | ERR10431635 |
| PRY | 2020 | DTU_2020_TWIW_01_PRY_ASU_LCPMC_019 | Urine | ERR10431885 |
| PRY | 2020 | DTU_2020_TWIW_01_PRY_ASU_LCPMC_020 | Urine | ERR10431766 |
| PRY | 2020 | DTU_2020_TWIW_01_PRY_ASU_LCPMC_021 | Urine | ERR10431784 |
| PRY | 2020 | DTU_2020_TWIW_01_PRY_ASU_LCPMC_022 | Urine | ERR10431886 |
| PRY | 2020 | DTU_2020_TWIW_01_PRY_ASU_LCPMC_023 | Urine | ERR10431790 |
| PRY | 2020 | DTU_2020_TWIW_01_PRY_ASU_LCPMC_024 | Urine | ERR10432294 |
| PRY | 2020 | DTU_2020_TWIW_01_PRY_ASU_LCPMC_025 | Wound Pus Biopsy | ERR10431959 |
| PRY | 2020 | DTU_2020_TWIW_01_PRY_ASU_LCPMC_026 | Wound Pus Biopsy | ERR10431638 |
| PRY | 2020 | DTU_2020_TWIW_01_PRY_ASU_LCPMC_027 | Wound Pus Biopsy | ERR10431773 |
| PRY | 2020 | DTU_2020_TWIW_01_PRY_ASU_LCPMC_027 | Wound Pus Biopsy | ERR14129395 |
| PRY | 2020 | DTU_2020_TWIW_01_PRY_ASU_LCPMC_028 | Wound Pus Biopsy | ERR10431977 |
| PRY | 2020 | DTU_2020_TWIW_01_PRY_ASU_LCPMC_029 | Urine | ERR10431783 |
| PRY | 2020 | DTU_2020_TWIW_01_PRY_ASU_LCPMC_030 | Urine | ERR10431778 |
| PRY | 2020 | DTU_2020_TWIW_01_PRY_ASU_LCPMC_031 | Urine | ERR10431775 |
| PRY | 2020 | DTU_2020_TWIW_01_PRY_ASU_LCPMC_032 | Urine | ERR10431777 |
| PRY | 2020 | DTU_2020_TWIW_01_PRY_ASU_LCPMC_033 | Urine | ERR10432284 |
| PRY | 2020 | DTU_2020_TWIW_01_PRY_ASU_LCPMC_034 | Urine | ERR10431826 |
| PRY | 2020 | DTU_2020_TWIW_01_PRY_ASU_LCPMC_035 | Urine | ERR10431781 |
| PRY | 2020 | DTU_2020_TWIW_01_PRY_ASU_LCPMC_036 | Urine | ERR10432276 |
| PRY | 2020 | DTU_2020_TWIW_01_PRY_ASU_LCPMC_037 | Urine | ERR10431874 |
| PRY | 2020 | DTU_2020_TWIW_01_PRY_ASU_LCPMC_038 | Urine | ERR10431842 |
| PRY | 2020 | DTU_2020_TWIW_01_PRY_ASU_LCPMC_039 | Urine | ERR10431802 |
| PRY | 2020 | DTU_2020_TWIW_01_PRY_ASU_LCPMC_040 | Urine | ERR10431941 |
| PRY | 2020 | DTU_2020_TWIW_01_PRY_ASU_LCPMC_041 | Urine | ERR10431805 |
| PRY | 2020 | DTU_2020_TWIW_01_PRY_ASU_LCPMC_042 | Urine | ERR10431807 |
| PRY | 2020 | DTU_2020_TWIW_01_PRY_ASU_LCPMC_043 | Other | ERR10431889 |
| PRY | 2020 | DTU_2020_TWIW_01_PRY_ASU_LCPMC_044 | Urine | ERR10431812 |
| PRY | 2020 | DTU_2020_TWIW_01_PRY_ASU_LCPMC_045 | Other | ERR10431984 |
| PRY | 2020 | DTU_2020_TWIW_01_PRY_ASU_LCPMC_046 | Urine | ERR10431791 |
| PRY | 2020 | DTU_2020_TWIW_01_PRY_ASU_LCPMC_047 | Urine | ERR10431794 |
| PRY | 2020 | DTU_2020_TWIW_01_PRY_ASU_LCPMC_048 | Urine | ERR10431811 |
| PRY | 2020 | DTU_2020_TWIW_01_PRY_ASU_LCPMC_049 | Urine | ERR10431900 |
| PRY | 2020 | DTU_2020_TWIW_01_PRY_ASU_LCPMC_050 | Wound Pus Biopsy | ERR10431979 |
| PRY | 2020 | DTU_2020_TWIW_01_PRY_ASU_LCPMC_051 | Urine | ERR10431818 |
| PRY | 2020 | DTU_2020_TWIW_01_PRY_ASU_LCPMC_052 | Urine | ERR10431901 |
| PRY | 2020 | DTU_2020_TWIW_01_PRY_ASU_LCPMC_053 | Urine | ERR10431825 |
| PRY | 2020 | DTU_2020_TWIW_01_PRY_ASU_LCPMC_054 | Urine | ERR10431675 |
| PRY | 2020 | DTU_2020_TWIW_01_PRY_ASU_LCPMC_055 | Blood | ERR10431827 |
| PRY | 2020 | DTU_2020_TWIW_01_PRY_ASU_LCPMC_056 | Urine | ERR10431829 |
| PRY | 2020 | DTU_2020_TWIW_01_PRY_ASU_LCPMC_057 | Urine | ERR10431845 |
| PRY | 2020 | DTU_2020_TWIW_01_PRY_ASU_LCPMC_058 | Urine | ERR10431836 |
| PRY | 2020 | DTU_2020_TWIW_01_PRY_ASU_LCPMC_059 | Urine | ERR10431843 |
| PRY | 2020 | DTU_2020_TWIW_01_PRY_ASU_LCPMC_060 | Wound Pus Biopsy | ERR10431992 |
| PRY | 2020 | DTU_2020_TWIW_01_PRY_CIU_RH_001 | Respiratory System | ERR10431668 |
| PRY | 2020 | DTU_2020_TWIW_01_PRY_CIU_RH_002 | Respiratory System | ERR10431857 |
| PRY | 2020 | DTU_2020_TWIW_01_PRY_CIU_RH_002 | Respiratory System | ERR14129398 |
| PRY | 2020 | DTU_2020_TWIW_01_PRY_CIU_RH_003 | Respiratory System | ERR10431861 |
| PRY | 2020 | DTU_2020_TWIW_01_PRY_CIU_RH_004 | Respiratory System | ERR10431858 |
| PRY | 2020 | DTU_2020_TWIW_01_PRY_CIU_RH_005 | Other | ERR10431964 |
| PRY | 2020 | DTU_2020_TWIW_01_PRY_CIU_RH_006 | Respiratory System | ERR10431866 |
| PRY | 2020 | DTU_2020_TWIW_01_PRY_CIU_RH_007 | Respiratory System | ERR10431661 |
| PRY | 2020 | DTU_2020_TWIW_01_PRY_CIU_RH_008 | Respiratory System | ERR10431677 |
| PRY | 2020 | DTU_2020_TWIW_01_PRY_CIU_RH_009 | Respiratory System | ERR10432309 |
| PRY | 2020 | DTU_2020_TWIW_01_PRY_CIU_RH_010 | Wound Pus Biopsy | ERR10431932 |
| PRY | 2020 | DTU_2020_TWIW_01_PRY_CIU_RH_011 | Other | ERR10431683 |
| PRY | 2020 | DTU_2020_TWIW_01_PRY_CIU_RH_012 | Respiratory System | ERR10431881 |
| PRY | 2020 | DTU_2020_TWIW_01_PRY_CIU_RH_013 | Respiratory System | ERR10431681 |
| PRY | 2020 | DTU_2020_TWIW_01_PRY_CIU_RH_014 | Wound Pus Biopsy | ERR10431961 |
| PRY | 2020 | DTU_2020_TWIW_01_PRY_CIU_RH_015 | Blood | ERR10431679 |
| PRY | 2020 | DTU_2020_TWIW_01_PRY_CIU_RH_016 | Urine | ERR10431780 |
| PRY | 2020 | DTU_2020_TWIW_01_PRY_CIU_RH_017 | Urine | ERR10431772 |
| PRY | 2020 | DTU_2020_TWIW_01_PRY_CIU_RH_018 | Other | ERR10431751 |
| PRY | 2020 | DTU_2020_TWIW_01_PRY_CIU_RH_019 | Urine | ERR10431868 |
| PRY | 2020 | DTU_2020_TWIW_01_PRY_CIU_RH_020 | Blood | ERR10431927 |
| PRY | 2020 | DTU_2020_TWIW_01_PRY_CIU_RH_021 | Urine | ERR10431923 |
| PRY | 2020 | DTU_2020_TWIW_01_PRY_CIU_RH_022 | Respiratory System | ERR10431685 |
| PRY | 2020 | DTU_2020_TWIW_01_PRY_CIU_RH_023 | Respiratory System | ERR10431879 |
| PRY | 2020 | DTU_2020_TWIW_01_PRY_CIU_RH_025 | Other | ERR10432311 |
| PRY | 2020 | DTU_2020_TWIW_01_PRY_CIU_RH_026 | Respiratory System | ERR10431684 |
| PRY | 2020 | DTU_2020_TWIW_01_PRY_CIU_RH_027 | Respiratory System | ERR10431875 |
| PRY | 2020 | DTU_2020_TWIW_01_PRY_CIU_RH_027 | Respiratory System | ERR14129399 |
| PRY | 2020 | DTU_2020_TWIW_01_PRY_CIU_RH_028 | Respiratory System | ERR10431789 |
| PRY | 2020 | DTU_2020_TWIW_01_PRY_CIU_RH_029 | Wound Pus Biopsy | NA |
| PRY | 2020 | DTU_2020_TWIW_01_PRY_CIU_RH_030 | Respiratory System | ERR10431888 |
| PRY | 2020 | DTU_2020_TWIW_01_PRY_CIU_RH_031 | Respiratory System | ERR10431690 |
| PRY | 2020 | DTU_2020_TWIW_01_PRY_CIU_RH_032 | Swab | ERR10431813 |
| PRY | 2020 | DTU_2020_TWIW_01_PRY_CIU_RH_033 | Blood | ERR10431890 |
| PRY | 2020 | DTU_2020_TWIW_01_PRY_CIU_RH_033 | Blood | ERR14129400 |
| PRY | 2020 | DTU_2020_TWIW_01_PRY_CIU_RH_034 | Respiratory System | ERR10431648 |
| PRY | 2020 | DTU_2020_TWIW_01_PRY_CIU_RH_035 | Respiratory System | ERR10432296 |
| PRY | 2020 | DTU_2020_TWIW_01_PRY_CIU_RH_036 | Urine | ERR10431925 |
| PRY | 2020 | DTU_2020_TWIW_01_PRY_CIU_RH_037 | Respiratory System | ERR10431691 |
| PRY | 2020 | DTU_2020_TWIW_01_PRY_CIU_RH_038 | Blood | ERR10431970 |
| PRY | 2020 | DTU_2020_TWIW_01_PRY_CIU_RH_039 | Wound Pus Biopsy | ERR10432275 |
| PRY | 2020 | DTU_2020_TWIW_01_PRY_CIU_RH_040 | Swab | ERR10431982 |
| PRY | 2020 | DTU_2020_TWIW_01_PRY_CIU_RH_041 | Respiratory System | ERR10431689 |
| PRY | 2020 | DTU_2020_TWIW_01_PRY_CIU_RH_042 | Blood | ERR10431692 |
| PRY | 2020 | DTU_2020_TWIW_01_PRY_CIU_RH_043 | Urine | ERR10431822 |
| PRY | 2020 | DTU_2020_TWIW_01_PRY_CIU_RH_044 | Wound Pus Biopsy | ERR10431824 |
| PRY | 2020 | DTU_2020_TWIW_01_PRY_CIU_RH_045 | Urine | ERR10432316 |
| PRY | 2020 | DTU_2020_TWIW_01_PRY_CIU_RH_046 | Respiratory System | ERR10431981 |
| PRY | 2020 | DTU_2020_TWIW_01_PRY_CIU_RH_047 | Urine | ERR10431630 |
| PRY | 2020 | DTU_2020_TWIW_01_PRY_CIU_RH_048 | Urine | ERR10431816 |
| PRY | 2020 | DTU_2020_TWIW_01_PRY_CIU_RH_049 | Urine | ERR10431936 |
| PRY | 2020 | DTU_2020_TWIW_01_PRY_CIU_RH_050 | Respiratory System | ERR10431658 |
| PRY | 2020 | DTU_2020_TWIW_01_PRY_CIU_RH_051 | Other | ERR10431931 |
| PRY | 2020 | DTU_2020_TWIW_01_PRY_CIU_RH_052 | Respiratory System | ERR10431701 |
| PRY | 2020 | DTU_2020_TWIW_01_PRY_CIU_RH_053 | Urine | ERR10431828 |
| PRY | 2020 | DTU_2020_TWIW_01_PRY_CIU_RH_054 | Urine | ERR10431830 |
| PRY | 2020 | DTU_2020_TWIW_01_PRY_CIU_RH_055 | Wound Pus Biopsy | ERR10431706 |
| PRY | 2020 | DTU_2020_TWIW_01_PRY_CIU_RH_056 | Respiratory System | ERR10432326 |
| PRY | 2020 | DTU_2020_TWIW_01_PRY_CIU_RH_057 | Respiratory System | ERR10431938 |
| PRY | 2020 | DTU_2020_TWIW_01_PRY_CIU_RH_058 | Respiratory System | ERR10431909 |
| PRY | 2020 | DTU_2020_TWIW_01_PRY_CIU_RH_059 | Swab | ERR10431670 |
| PRY | 2020 | DTU_2020_TWIW_01_PRY_CIU_RH_060 | Swab | ERR10432317 |
| PRY | 2020 | DTU_2020_TWIW_01_PRY_ASU_CLPH_001 | Respiratory System | ERR10431674 |
| PRY | 2020 | DTU_2020_TWIW_01_PRY_ASU_CLPH_002 | Respiratory System | ERR10431623 |
| PRY | 2020 | DTU_2020_TWIW_01_PRY_ASU_CLPH_002 | Respiratory System | ERR14129402 |
| PRY | 2020 | DTU_2020_TWIW_01_PRY_ASU_CLPH_003 | Respiratory System | ERR10431669 |
| PRY | 2020 | DTU_2020_TWIW_01_PRY_ASU_CLPH_004 | Respiratory System | ERR10431620 |
| PRY | 2020 | DTU_2020_TWIW_01_PRY_ASU_CLPH_005 | Blood | ERR10431673 |
| PRY | 2020 | DTU_2020_TWIW_01_PRY_ASU_CLPH_006 | Wound Pus Biopsy | ERR10431947 |
| PRY | 2020 | DTU_2020_TWIW_01_PRY_ASU_CLPH_007 | Wound Pus Biopsy | ERR10431945 |
| PRY | 2020 | DTU_2020_TWIW_01_PRY_ASU_CLPH_008 | Wound Pus Biopsy | ERR10431943 |
| PRY | 2020 | DTU_2020_TWIW_01_PRY_ASU_CLPH_009 | Urine | ERR10432280 |
| PRY | 2020 | DTU_2020_TWIW_01_PRY_ASU_CLPH_009 | Urine | ERR14150450 |
| PRY | 2020 | DTU_2020_TWIW_01_PRY_ASU_CLPH_010 | Blood | ERR10431664 |
| PRY | 2020 | DTU_2020_TWIW_01_PRY_ASU_CLPH_011 | Blood | ERR10432282 |
| PRY | 2020 | DTU_2020_TWIW_01_PRY_ASU_CLPH_012 | Blood | ERR10431952 |
| PRY | 2020 | DTU_2020_TWIW_01_PRY_ASU_CLPH_013 | Respiratory System | ERR10431626 |
| PRY | 2020 | DTU_2020_TWIW_01_PRY_ASU_CLPH_014 | Respiratory System | ERR10431864 |
| PRY | 2020 | DTU_2020_TWIW_01_PRY_ASU_CLPH_015 | Respiratory System | ERR10431671 |
| PRY | 2020 | DTU_2020_TWIW_01_PRY_ASU_CLPH_016 | Blood | ERR10432302 |
| PRY | 2020 | DTU_2020_TWIW_01_PRY_ASU_CLPH_017 | Urine | ERR10431883 |
| PRY | 2020 | DTU_2020_TWIW_01_PRY_ASU_CLPH_018 | Blood | ERR10431963 |
| PRY | 2020 | DTU_2020_TWIW_01_PRY_ASU_CLPH_019 | Urine | ERR10431768 |
| PRY | 2020 | DTU_2020_TWIW_01_PRY_ASU_CLPH_020 | Other | ERR10431632 |
| PRY | 2020 | DTU_2020_TWIW_01_PRY_ASU_CLPH_021 | Wound Pus Biopsy | ERR10431967 |
| PRY | 2020 | DTU_2020_TWIW_01_PRY_ASU_CLPH_022 | Wound Pus Biopsy | ERR10431771 |
| PRY | 2020 | DTU_2020_TWIW_01_PRY_ASU_CLPH_023 | Wound Pus Biopsy | ERR10431637 |
| PRY | 2020 | DTU_2020_TWIW_01_PRY_ASU_CLPH_024 | Blood | ERR10432308 |
| PRY | 2020 | DTU_2020_TWIW_01_PRY_ASU_CLPH_025 | Blood | ERR10432306 |
| PRY | 2020 | DTU_2020_TWIW_01_PRY_ASU_CLPH_026 | Swab | ERR10432292 |
| PRY | 2020 | DTU_2020_TWIW_01_PRY_ASU_CLPH_026 | Swab | ERR14150518 |
| PRY | 2020 | DTU_2020_TWIW_01_PRY_ASU_CLPH_027 | Swab | ERR10431856 |
| PRY | 2020 | DTU_2020_TWIW_01_PRY_ASU_CLPH_028 | Respiratory System | ERR10431686 |
| PRY | 2020 | DTU_2020_TWIW_01_PRY_ASU_CLPH_029 | Urine | ERR10431792 |
| PRY | 2020 | DTU_2020_TWIW_01_PRY_ASU_CLPH_030 | Urine | ERR10431801 |
| PRY | 2020 | DTU_2020_TWIW_01_PRY_ASU_CLPH_031 | Urine | ERR10431905 |
| PRY | 2020 | DTU_2020_TWIW_01_PRY_ASU_CLPH_032 | Urine | ERR10431815 |
| PRY | 2020 | DTU_2020_TWIW_01_PRY_ASU_CLPH_033 | Urine | ERR10431821 |
| PRY | 2020 | DTU_2020_TWIW_01_PRY_ASU_CLPH_034 | Other | ERR10431649 |
| PRY | 2020 | DTU_2020_TWIW_01_PRY_ASU_CLPH_035 | Other | ERR10431899 |
| PRY | 2020 | DTU_2020_TWIW_01_PRY_ASU_CLPH_036 | Blood | ERR10431891 |
| PRY | 2020 | DTU_2020_TWIW_01_PRY_ASU_CLPH_037 | Wound Pus Biopsy | ERR10431893 |
| PRY | 2020 | DTU_2020_TWIW_01_PRY_ASU_CLPH_038 | Wound Pus Biopsy | ERR10431906 |
| PRY | 2020 | DTU_2020_TWIW_01_PRY_ASU_CLPH_039 | Wound Pus Biopsy | ERR10431987 |
| PRY | 2020 | DTU_2020_TWIW_01_PRY_ASU_CLPH_040 | Urine | ERR10431837 |
| PRY | 2020 | DTU_2020_TWIW_01_PRY_ASU_CLPH_041 | Urine | ERR10431922 |
| PRY | 2020 | DTU_2020_TWIW_01_PRY_ASU_CLPH_042 | Urine | ERR10431834 |
| PRY | 2020 | DTU_2020_TWIW_01_PRY_ASU_CLPH_043 | Urine | ERR10431831 |
| PRY | 2020 | DTU_2020_TWIW_01_PRY_ASU_CLPH_044 | Wound Pus Biopsy | ERR10431895 |
| PRY | 2020 | DTU_2020_TWIW_01_PRY_ASU_CLPH_045 | Wound Pus Biopsy | ERR10431898 |
| PRY | 2020 | DTU_2020_TWIW_01_PRY_ASU_CLPH_046 | Urine | ERR10431914 |
| PRY | 2020 | DTU_2020_TWIW_01_PRY_ASU_CLPH_047 | Urine | ERR10431915 |
| PRY | 2020 | DTU_2020_TWIW_01_PRY_ASU_CLPH_048 | Respiratory System | ERR10431918 |
| PRY | 2020 | DTU_2020_TWIW_01_PRY_ASU_CLPH_049 | Respiratory System | ERR10431919 |
| PRY | 2020 | DTU_2020_TWIW_01_PRY_ASU_CLPH_050 | Respiratory System | ERR10431916 |
| PRY | 2020 | DTU_2020_TWIW_01_PRY_ASU_CLPH_051 | Respiratory System | ERR10431697 |
| PRY | 2020 | DTU_2020_TWIW_01_PRY_ASU_CLPH_052 | Respiratory System | ERR10431917 |
| PRY | 2020 | DTU_2020_TWIW_01_PRY_ASU_CLPH_053 | Urine | ERR10432379 |
| PRY | 2020 | DTU_2020_TWIW_01_PRY_ASU_CLPH_054 | Blood | ERR10431997 |
| PRY | 2020 | DTU_2020_TWIW_01_PRY_ASU_CLPH_055 | Respiratory System | ERR10431911 |
| PRY | 2020 | DTU_2020_TWIW_01_PRY_ASU_CLPH_056 | Urine | ERR10431929 |
| PRY | 2020 | DTU_2020_TWIW_01_PRY_CIU_FMNU_001 | Urine | ERR10431844 |
| PRY | 2020 | DTU_2020_TWIW_01_PRY_CIU_FMNU_001 | Urine | ERR14129403 |
| PRY | 2020 | DTU_2020_TWIW_01_PRY_CIU_FMNU_002 | Urine | ERR10431849 |
| PRY | 2020 | DTU_2020_TWIW_01_PRY_CIU_FMNU_003 | Urine | ERR10431723 |
| PRY | 2020 | DTU_2020_TWIW_01_PRY_CIU_FMNU_004 | Other | ERR10431955 |
| PRY | 2020 | DTU_2020_TWIW_01_PRY_CIU_FMNU_005 | Urine | ERR10431848 |
| PRY | 2020 | DTU_2020_TWIW_01_PRY_CIU_FMNU_006 | Urine | ERR10431851 |
| PRY | 2020 | DTU_2020_TWIW_01_PRY_CIU_FMNU_007 | Other | ERR10431615 |
| PRY | 2020 | DTU_2020_TWIW_01_PRY_CIU_FMNU_008 | Urine | ERR10431852 |
| PRY | 2020 | DTU_2020_TWIW_01_PRY_CIU_FMNU_009 | Urine | ERR10431850 |
| PRY | 2020 | DTU_2020_TWIW_01_PRY_CIU_FMNU_010 | Urine | ERR10431713 |
| PRY | 2020 | DTU_2020_TWIW_01_PRY_CIU_FMNU_011 | Wound Pus Biopsy | ERR10431953 |
| PRY | 2020 | DTU_2020_TWIW_01_PRY_CIU_FMNU_012 | Blood | ERR10431636 |
| PRY | 2020 | DTU_2020_TWIW_01_PRY_CIU_FMNU_013 | Urine | ERR10431619 |
| PRY | 2020 | DTU_2020_TWIW_01_PRY_CIU_FMNU_014 | Blood | ERR10431958 |
| PRY | 2020 | DTU_2020_TWIW_01_PRY_CIU_FMNU_015 | Other | ERR10431853 |
| PRY | 2020 | DTU_2020_TWIW_01_PRY_CIU_FMNU_016 | Respiratory System | ERR10432305 |
| PRY | 2020 | DTU_2020_TWIW_01_PRY_CIU_FMNU_017 | Respiratory System | ERR10431933 |
| PRY | 2020 | DTU_2020_TWIW_01_PRY_CIU_FMNU_018 | Blood | ERR10431676 |
| PRY | 2020 | DTU_2020_TWIW_01_PRY_CIU_FMNU_019 | Blood | ERR10432268 |
| PRY | 2020 | DTU_2020_TWIW_01_PRY_CIU_FMNU_020 | Urine | ERR10432343 |
| PRY | 2020 | DTU_2020_TWIW_01_PRY_CIU_FMNU_021 | Wound Pus Biopsy | ERR10431956 |
| PRY | 2020 | DTU_2020_TWIW_01_PRY_CIU_FMNU_022 | Urine | ERR10431622 |
| PRY | 2020 | DTU_2020_TWIW_01_PRY_CIU_FMNU_023 | Blood | ERR10431682 |
| PRY | 2020 | DTU_2020_TWIW_01_PRY_CIU_FMNU_024 | Urine | ERR10432327 |
| PRY | 2020 | DTU_2020_TWIW_01_PRY_CIU_FMNU_025 | Urine | ERR10431793 |
| PRY | 2020 | DTU_2020_TWIW_01_PRY_CIU_FMNU_026 | Respiratory System | ERR10431643 |
| PRY | 2020 | DTU_2020_TWIW_01_PRY_CIU_FMNU_027 | Urine | ERR10431647 |
| PRY | 2020 | DTU_2020_TWIW_01_PRY_CIU_FMNU_028 | Respiratory System | ERR10431651 |
| PRY | 2020 | DTU_2020_TWIW_01_PRY_CIU_FMNU_029 | Blood | ERR10431989 |
| PRY | 2020 | DTU_2020_TWIW_01_PRY_CIU_FMNU_030 | Respiratory System | ERR10431935 |
| PRY | 2020 | DTU_2020_TWIW_01_PRY_CIU_FMNU_031 | Urine | ERR10431833 |
| PRY | 2020 | DTU_2020_TWIW_01_PRY_CIU_FMNU_032 | Blood | ERR10432322 |
| PRY | 2020 | DTU_2020_TWIW_01_PRY_CIU_FMNU_033 | Blood | ERR10432337 |
| PRY | 2020 | DTU_2020_TWIW_01_PRY_CIU_FMNU_034 | Blood | ERR10432333 |
| PRY | 2020 | DTU_2020_TWIW_01_PRY_CIU_FMNU_035 | Urine | ERR10431839 |
| PRY | 2020 | DTU_2020_TWIW_01_PRY_CIU_FMNU_036 | Urine | ERR10431838 |
| PRY | 2020 | DTU_2020_TWIW_01_PRY_CIU_FMNU_037 | Blood | ERR10431924 |
| PRY | 2020 | DTU_2020_TWIW_01_PRY_CIU_FMNU_038 | Respiratory System | ERR10431921 |
| PRY | 2020 | DTU_2020_TWIW_01_PRY_CIU_FMNU_039 | Blood | ERR10432321 |
| PRY | 2020 | DTU_2020_TWIW_01_PRY_ENC_001 | Urine | ERR10431709 |
| PRY | 2020 | DTU_2020_TWIW_01_PRY_ENC_002 | Wound Pus Biopsy | ERR10431854 |
| PRY | 2020 | DTU_2020_TWIW_01_PRY_ENC_003 | Other | ERR10431715 |
| PRY | 2020 | DTU_2020_TWIW_01_PRY_ENC_004 | Other | ERR10431711 |
| PRY | 2020 | DTU_2020_TWIW_01_PRY_ENC_004 | Other | ERR14150539 |
| PRY | 2020 | DTU_2020_TWIW_01_PRY_ENC_005 | Wound Pus Biopsy | ERR10431894 |
| PRY | 2020 | DTU_2020_TWIW_01_PRY_ENC_006 | Urine | ERR10431716 |
| PRY | 2020 | DTU_2020_TWIW_01_PRY_ENC_007 | Wound Pus Biopsy | ERR10431944 |
| PRY | 2020 | DTU_2020_TWIW_01_PRY_ENC_008 | Urine | ERR10439814 |
| PRY | 2020 | DTU_2020_TWIW_01_PRY_ENC_010 | Wound Pus Biopsy | ERR10431860 |
| PRY | 2020 | DTU_2020_TWIW_01_PRY_ENC_011 | Urine | ERR10431720 |
| PRY | 2020 | DTU_2020_TWIW_01_PRY_ENC_012 | Urine | ERR10431721 |
| PRY | 2020 | DTU_2020_TWIW_01_PRY_ENC_012 | Urine | ERR14129406 |
| PRY | 2020 | DTU_2020_TWIW_01_PRY_ENC_013 | Wound Pus Biopsy | ERR10431870 |
| PRY | 2020 | DTU_2020_TWIW_01_PRY_ENC_014 | Wound Pus Biopsy | ERR10431869 |
| PRY | 2020 | DTU_2020_TWIW_01_PRY_ENC_014 | Wound Pus Biopsy | ERR14129407 |
| PRY | 2020 | DTU_2020_TWIW_01_PRY_ENC_015 | Wound Pus Biopsy | ERR10431718 |
| PRY | 2020 | DTU_2020_TWIW_01_PRY_ENC_016 | Wound Pus Biopsy | ERR10431722 |
| PRY | 2020 | DTU_2020_TWIW_01_PRY_ENC_017 | Blood | ERR10431957 |
| PRY | 2020 | DTU_2020_TWIW_01_PRY_ENC_018 | Blood | ERR10432270 |
| PRY | 2020 | DTU_2020_TWIW_01_PRY_ENC_019 | Blood | ERR10432244 |
| PRY | 2020 | DTU_2020_TWIW_01_PRY_ENC_020 | Urine | ERR10431859 |
| PRY | 2020 | DTU_2020_TWIW_01_PRY_ENC_021 | Urine | ERR10431717 |
| PRY | 2020 | DTU_2020_TWIW_01_PRY_ENC_022 | Other | ERR10431749 |
| PRY | 2020 | DTU_2020_TWIW_01_PRY_ENC_023 | Urine | ERR10431728 |
| PRY | 2020 | DTU_2020_TWIW_01_PRY_ENC_024 | Urine | ERR10431926 |
| PRY | 2020 | DTU_2020_TWIW_01_PRY_ENC_025 | Other | ERR10432319 |
| PRY | 2020 | DTU_2020_TWIW_01_PRY_ENC_026 | Urine | ERR10431863 |
| PRY | 2020 | DTU_2020_TWIW_01_PRY_ENC_027 | Urine | ERR10431741 |
| PRY | 2020 | DTU_2020_TWIW_01_PRY_ENC_028 | Urine | ERR10431759 |
| PRY | 2020 | DTU_2020_TWIW_01_PRY_ENC_029 | Blood | ERR10431847 |
| PRY | 2020 | DTU_2020_TWIW_01_PRY_ENC_030 | Wound Pus Biopsy | ERR10431735 |
| PRY | 2020 | DTU_2020_TWIW_01_PRY_ENC_031 | Other | ERR10431640 |
| PRY | 2020 | DTU_2020_TWIW_01_PRY_ENC_032 | Other | ERR10431846 |
| PRY | 2020 | DTU_2020_TWIW_01_PRY_ENC_033 | Blood | ERR10432274 |
| PRY | 2020 | DTU_2020_TWIW_01_PRY_ENC_034 | Urine | ERR10431876 |
| PRY | 2020 | DTU_2020_TWIW_01_PRY_ENC_035 | Urine | ERR10431887 |
| PRY | 2020 | DTU_2020_TWIW_01_PRY_ENC_036 | Wound Pus Biopsy | ERR10431655 |
| PRY | 2020 | DTU_2020_TWIW_01_PRY_ENC_037 | Swab | ERR10431991 |
| PRY | 2020 | DTU_2020_TWIW_01_PRY_ENC_038 | Blood | ERR10432266 |
| PRY | 2020 | DTU_2020_TWIW_01_PRY_ENC_039 | Blood | ERR10432263 |
| PRY | 2020 | DTU_2020_TWIW_01_PRY_ENC_040 | Urine | ERR10431971 |
| PRY | 2020 | DTU_2020_TWIW_01_PRY_ENC_041 | Blood | ERR10431978 |
| PRY | 2020 | DTU_2020_TWIW_01_PRY_ENC_042 | Urine | ERR10432264 |
| PRY | 2020 | DTU_2020_TWIW_01_PRY_ENC_043 | Blood | ERR10432265 |
| PRY | 2020 | DTU_2020_TWIW_01_PRY_ENC_044 | Other | ERR10432315 |
| PRY | 2020 | DTU_2020_TWIW_01_PRY_ENC_045 | Other | ERR10431776 |
| PRY | 2020 | DTU_2020_TWIW_01_PRY_ENC_046 | Urine | ERR10431796 |
| PRY | 2020 | DTU_2020_TWIW_01_PRY_ENC_047 | Swab | ERR10431980 |
| PRY | 2020 | DTU_2020_TWIW_01_PRY_ENC_048 | Urine | ERR10431808 |
| PRY | 2020 | DTU_2020_TWIW_01_PRY_ENC_049 | Urine | ERR10431896 |
| PRY | 2020 | DTU_2020_TWIW_01_PRY_ENC_050 | Wound Pus Biopsy | ERR10431806 |
| PRY | 2020 | DTU_2020_TWIW_01_PRY_ENC_051 | Urine | ERR10431809 |
| PRY | 2020 | DTU_2020_TWIW_01_PRY_ENC_052 | Blood | ERR10432269 |
| PRY | 2020 | DTU_2020_TWIW_01_PRY_ENC_053 | Wound Pus Biopsy | ERR10431996 |
| PRY | 2020 | DTU_2020_TWIW_01_PRY_ENC_054 | Urine | ERR10431940 |
| PRY | 2020 | DTU_2020_TWIW_01_PRY_ENC_055 | Urine | ERR10432285 |
| PRY | 2020 | DTU_2020_TWIW_01_PRY_ENC_056 | Urine | ERR10431840 |
| PRY | 2020 | DTU_2020_TWIW_01_PRY_ENC_057 | Urine | ERR10431913 |
| PRY | 2020 | DTU_2020_TWIW_01_PRY_ENC_058 | Other | ERR10432325 |
| PRY | 2020 | DTU_2020_TWIW_01_PRY_ENC_059 | Other | ERR10431995 |
| PRY | 2020 | DTU_2020_TWIW_01_PRY_ENC_060 | Other | ERR10431693 |
| PRY | 2020 | DTU_2020_TWIW_01_PRY_ENC_061 | Urine | ERR10431912 |
| PRY | 2020 | DTU_2020_TWIW_01_PRY_ENC_062 | Urine | ERR10431832 |
| PRY | 2020 | DTU_2020_TWIW_01_PRY_SAN_001 | Urine | ERR10431754 |
| PRY | 2020 | DTU_2020_TWIW_01_PRY_SAN_002 | Urine | ERR10431761 |
| PRY | 2020 | DTU_2020_TWIW_01_PRY_SAN_003 | Urine | ERR10431748 |
| PRY | 2020 | DTU_2020_TWIW_01_PRY_SAN_004 | Urine | ERR10431871 |
| PRY | 2020 | DTU_2020_TWIW_01_PRY_SAN_005 | Urine | ERR10431939 |
| PRY | 2020 | DTU_2020_TWIW_01_PRY_SAN_007 | Urine | ERR10431767 |
| PRY | 2020 | DTU_2020_TWIW_01_PRY_SAN_008 | Urine | ERR10431750 |
| PRY | 2020 | DTU_2020_TWIW_01_PRY_SAN_009 | Wound Pus Biopsy | ERR10431975 |
| PRY | 2020 | DTU_2020_TWIW_01_PRY_SAN_010 | Respiratory System | ERR10431946 |
| PRY | 2020 | DTU_2020_TWIW_01_PRY_SAN_011 | Urine | ERR10432281 |
| PRY | 2020 | DTU_2020_TWIW_01_PRY_SAN_012 | Urine | ERR10431726 |
| PRY | 2020 | DTU_2020_TWIW_01_PRY_SAN_013 | Urine | ERR10431727 |
| PRY | 2020 | DTU_2020_TWIW_01_PRY_SAN_014 | Urine | ERR10431733 |
| PRY | 2020 | DTU_2020_TWIW_01_PRY_SAN_015 | Urine | ERR10431729 |
| PRY | 2020 | DTU_2020_TWIW_01_PRY_SAN_016 | Urine | ERR10431731 |
| PRY | 2020 | DTU_2020_TWIW_01_PRY_SAN_017 | Urine | ERR10431862 |
| PRY | 2020 | DTU_2020_TWIW_01_PRY_SAN_018 | Urine | ERR10431736 |
| PRY | 2020 | DTU_2020_TWIW_01_PRY_SAN_019 | Urine | ERR10431734 |
| PRY | 2020 | DTU_2020_TWIW_01_PRY_SAN_020 | Urine | ERR10431738 |
| PRY | 2020 | DTU_2020_TWIW_01_PRY_SAN_021 | Urine | ERR10431742 |
| PRY | 2020 | DTU_2020_TWIW_01_PRY_SAN_022 | Urine | ERR10431737 |
| PRY | 2020 | DTU_2020_TWIW_01_PRY_SAN_023 | Urine | ERR10431707 |
| PRY | 2020 | DTU_2020_TWIW_01_PRY_SAN_024 | Urine | ERR10431625 |
| PRY | 2020 | DTU_2020_TWIW_01_PRY_SAN_025 | Urine | ERR10431744 |
| PRY | 2020 | DTU_2020_TWIW_01_PRY_SAN_026 | Urine | ERR10431739 |
| PRY | 2020 | DTU_2020_TWIW_01_PRY_SAN_027 | Urine | ERR10431758 |
| PRY | 2020 | DTU_2020_TWIW_01_PRY_SAN_028 | Urine | ERR10431753 |
| PRY | 2020 | DTU_2020_TWIW_01_PRY_SAN_029 | Wound Pus Biopsy | ERR10431937 |
| PRY | 2020 | DTU_2020_TWIW_01_PRY_SAN_031 | Urine | ERR10431757 |
| PRY | 2020 | DTU_2020_TWIW_01_PRY_SAN_032 | Urine | ERR10431755 |
| PRY | 2020 | DTU_2020_TWIW_01_PRY_SAN_033 | Urine | ERR10431756 |
| PRY | 2020 | DTU_2020_TWIW_01_PRY_SAN_034 | Wound Pus Biopsy | ERR10431951 |
| PRY | 2020 | DTU_2020_TWIW_01_PRY_SAN_037 | Respiratory System | ERR10432299 |
| PRY | 2020 | DTU_2020_TWIW_01_PRY_SAN_038 | Urine | ERR10431653 |
| PRY | 2020 | DTU_2020_TWIW_01_PRY_SAN_039 | Wound Pus Biopsy | ERR10431704 |
| PRY | 2020 | DTU_2020_TWIW_01_PRY_SAN_040 | Other | ERR10431642 |
| PRY | 2020 | DTU_2020_TWIW_01_PRY_SAN_041 | Wound Pus Biopsy | ERR10431882 |
| PRY | 2020 | DTU_2020_TWIW_01_PRY_SAN_042 | Wound Pus Biopsy | ERR10432320 |
| PRY | 2020 | DTU_2020_TWIW_01_PRY_SAN_043 | Respiratory System | ERR10431672 |
| PRY | 2020 | DTU_2020_TWIW_01_PRY_SAN_044 | Respiratory System | ERR10431624 |
| PRY | 2020 | DTU_2020_TWIW_01_PRY_SAN_045 | Respiratory System | ERR10431972 |
| PRY | 2020 | DTU_2020_TWIW_01_PRY_SAN_046 | Urine | ERR10431965 |
| PRY | 2020 | DTU_2020_TWIW_01_PRY_SAN_047 | Respiratory System | ERR10431897 |
| PRY | 2020 | DTU_2020_TWIW_01_PRY_SAN_048 | Urine | ERR10431990 |
| PRY | 2020 | DTU_2020_TWIW_01_PRY_SAN_049 | Urine | ERR10431786 |
| PRY | 2020 | DTU_2020_TWIW_01_PRY_SAN_050 | Urine | ERR10431782 |
| PRY | 2020 | DTU_2020_TWIW_01_PRY_SAN_051 | Urine | ERR10431795 |
| PRY | 2020 | DTU_2020_TWIW_01_PRY_SAN_052 | Urine | ERR10431797 |
| PRY | 2020 | DTU_2020_TWIW_01_PRY_SAN_053 | Urine | ERR10431803 |
| PRY | 2020 | DTU_2020_TWIW_01_PRY_SAN_054 | Urine | ERR10431650 |
| PRY | 2020 | DTU_2020_TWIW_01_PRY_SAN_055 | Urine | ERR10431798 |
| PRY | 2020 | DTU_2020_TWIW_01_PRY_SAN_056 | Wound Pus Biopsy | ERR10431974 |
| PRY | 2020 | DTU_2020_TWIW_01_PRY_SAN_057 | Urine | ERR10431817 |
| PRY | 2020 | DTU_2020_TWIW_01_PRY_SAN_058 | Other | ERR10431930 |
| PRY | 2020 | DTU_2020_TWIW_01_PRY_SAN_059 | Wound Pus Biopsy | ERR10432312 |
| PRY | 2020 | DTU_2020_TWIW_01_PRY_SAN_060 | Urine | ERR10431659 |
| PRY | 2020 | DTU_2020_TWIW_01_PRY_SAN_061 | Swab | ERR10432279 |
| PRY | 2020 | DTU_2020_TWIW_01_PRY_SAN_062 | Blood | ERR10432318 |
| PRY | 2020 | DTU_2020_TWIW_01_PRY_SAN_063 | Respiratory System | ERR10431710 |
| PRY | 2020 | DTU_2020_TWIW_01_PRY_NANC_001 | Blood | ERR10431667 |
| PRY | 2020 | DTU_2020_TWIW_01_PRY_NANC_002 | Blood | ERR10431665 |
| PRY | 2020 | DTU_2020_TWIW_01_PRY_NANC_003 | Urine | ERR10431724 |
| PRY | 2020 | DTU_2020_TWIW_01_PRY_NANC_004 | Urine | ERR10431725 |
| PRY | 2020 | DTU_2020_TWIW_01_PRY_NANC_005 | Respiratory System | ERR10431634 |
| PRY | 2020 | DTU_2020_TWIW_01_PRY_NANC_006 | Respiratory System | ERR10431627 |
| PRY | 2020 | DTU_2020_TWIW_01_PRY_NANC_007 | Urine | ERR10431740 |
| PRY | 2020 | DTU_2020_TWIW_01_PRY_NANC_008 | Respiratory System | ERR10431867 |
| PRY | 2020 | DTU_2020_TWIW_01_PRY_NANC_009 | Blood | ERR10431976 |
| PRY | 2020 | DTU_2020_TWIW_01_PRY_NANC_010 | Respiratory System | ERR10431752 |
| PRY | 2020 | DTU_2020_TWIW_01_PRY_NANC_011 | Respiratory System | ERR10431884 |
| PRY | 2020 | DTU_2020_TWIW_01_PRY_NANC_012 | Blood | ERR10431962 |
| PRY | 2020 | DTU_2020_TWIW_01_PRY_NANC_013 | Urine | ERR10431873 |
| PRY | 2020 | DTU_2020_TWIW_01_PRY_NANC_014 | Blood | ERR10431633 |
| PRY | 2020 | DTU_2020_TWIW_01_PRY_NANC_015 | Urine | ERR10431770 |
| PRY | 2020 | DTU_2020_TWIW_01_PRY_NANC_016 | Urine | ERR10431779 |
| PRY | 2020 | DTU_2020_TWIW_01_PRY_NANC_017 | Respiratory System | ERR10431687 |
| PRY | 2020 | DTU_2020_TWIW_01_PRY_NANC_018 | Respiratory System | ERR10431646 |
| PRY | 2020 | DTU_2020_TWIW_01_PRY_NANC_019 | Respiratory System | ERR10431694 |
| PRY | 2020 | DTU_2020_TWIW_01_PRY_NANC_020 | Blood | ERR10432300 |
| PRY | 2020 | DTU_2020_TWIW_01_PRY_NANC_021 | Respiratory System | ERR10431644 |
| PRY | 2020 | DTU_2020_TWIW_01_PRY_NANC_022 | Respiratory System | ERR10431988 |
| PRY | 2020 | DTU_2020_TWIW_01_PRY_NANC_023 | Respiratory System | ERR10431652 |
| PRY | 2020 | DTU_2020_TWIW_01_PRY_NANC_024 | Respiratory System | ERR10431878 |
| PRY | 2020 | DTU_2020_TWIW_01_PRY_NANC_025 | Respiratory System | ERR10431877 |
| PRY | 2020 | DTU_2020_TWIW_01_PRY_NANC_027 | Respiratory System | ERR10431702 |
| PRY | 2020 | DTU_2020_TWIW_01_PRY_NANC_028 | Respiratory System | ERR10431902 |
| PRY | 2020 | DTU_2020_TWIW_01_PRY_NANC_029 | Respiratory System | ERR10431688 |
| PRY | 2020 | DTU_2020_TWIW_01_PRY_NANC_030 | Respiratory System | ERR10431699 |
| PRY | 2020 | DTU_2020_TWIW_01_PRY_NANC_031 | Other | ERR10432330 |
| PRY | 2020 | DTU_2020_TWIW_01_PRY_NANC_032 | Respiratory System | ERR10431904 |
| PRY | 2020 | DTU_2020_TWIW_01_PRY_NANC_033 | Respiratory System | ERR10431810 |
| PRY | 2020 | DTU_2020_TWIW_01_PRY_NANC_034 | Urine | ERR10431819 |
| PRY | 2020 | DTU_2020_TWIW_01_PRY_NANC_035 | Respiratory System | ERR10431698 |
| PRY | 2020 | DTU_2020_TWIW_01_PRY_NANC_036 | Respiratory System | ERR10431695 |
| PRY | 2020 | DTU_2020_TWIW_01_PRY_NANC_037 | Respiratory System | ERR10431907 |
| PRY | 2020 | DTU_2020_TWIW_01_PRY_NANC_038 | Respiratory System | ERR10432304 |
| PRY | 2020 | DTU_2020_TWIW_01_PRY_NANC_039 | Urine | ERR10432271 |
| PRY | 2020 | DTU_2020_TWIW_01_PRY_NANC_040 | Respiratory System | ERR10431910 |
| PRY | 2020 | DTU_2020_TWIW_01_PRY_NANC_041 | Blood | ERR10431920 |
| PRY | 2020 | DTU_2020_TWIW_01_PRY_NANC_042 | Respiratory System | ERR10431696 |
| PRY | 2020 | DTU_2020_TWIW_01_PRY_NANC_043 | Urine | ERR10431666 |
| PRY | 2020 | DTU_2020_TWIW_01_PRY_NANC_044 | Urine | ERR10431663 |
| PRY | 2020 | DTU_2020_TWIW_01_PRY_NANC_045 | Respiratory System | ERR10432324 |
| PRY | 2020 | DTU_2020_TWIW_01_PRY_NANC_046 | Respiratory System | ERR10431660 |
| PRY | 2020 | DTU_2020_TWIW_01_PRY_NANC_047 | Respiratory System | ERR10431705 |
| PRY | 2020 | DTU_2020_TWIW_01_PRY_NANC_048 | Urine | ERR10431835 |
| PRY | 2020 | DTU_2020_TWIW_01_PRY_NANC_049 | Blood | ERR10432293 |
| PRY | 2020 | DTU_2020_TWIW_01_PRY_NANC_050 | Respiratory System | ERR10431700 |
| PRY | 2020 | DTU_2020_TWIW_01_PRY_NANC_051 | Urine | ERR10431934 |
| PRY | 2020 | DTU_2020_TWIW_01_PRY_NANC_052 | Respiratory System | ERR10432303 |
| PRY | 2020 | DTU_2020_TWIW_01_PRY_ASU_HSWI_001 | Blood | ERR10432310 |
| PRY | 2020 | DTU_2020_TWIW_01_PRY_ASU_HSWI_002 | Blood | ERR10431950 |
| PRY | 2020 | DTU_2020_TWIW_01_PRY_ASU_HSWI_003 | Blood | ERR10432277 |
| PRY | 2020 | DTU_2020_TWIW_01_PRY_ASU_HSWI_004 | Other | ERR10431841 |
| PRY | 2020 | DTU_2020_TWIW_01_PRY_ASU_HSWI_005 | Other | ERR10432332 |
| PRY | 2020 | DTU_2020_TWIW_01_PRY_ASU_HSWI_006 | Blood | ERR10432323 |
| PRY | 2020 | DTU_2020_TWIW_01_PRY_ASU_HSWI_007 | Urine | NA |
| PRY | 2020 | DTU_2020_TWIW_01_PRY_ASU_HSWI_008 | Other | ERR10431732 |
| PRY | 2020 | DTU_2020_TWIW_01_PRY_ASU_HSWI_009 | Other | ERR10431865 |
| PRY | 2020 | DTU_2020_TWIW_01_PRY_ASU_HSWI_010 | Urine | ERR10431745 |
| PRY | 2020 | DTU_2020_TWIW_01_PRY_ASU_HSWI_011 | Urine | ERR10431743 |
| PRY | 2020 | DTU_2020_TWIW_01_PRY_ASU_HSWI_012 | Urine | ERR10431762 |
| PRY | 2020 | DTU_2020_TWIW_01_PRY_ASU_HSWI_013 | Urine | ERR10431747 |
| PRY | 2020 | DTU_2020_TWIW_01_PRY_ASU_HSWI_014 | Wound Pus Biopsy | ERR10431960 |
| PRY | 2020 | DTU_2020_TWIW_01_PRY_ASU_HSWI_015 | Wound Pus Biopsy | ERR10431973 |
| PRY | 2020 | DTU_2020_TWIW_01_PRY_ASU_HSWI_017 | Other | ERR10431769 |
| PRY | 2020 | DTU_2020_TWIW_01_PRY_ASU_HSWI_018 | Urine | ERR10431760 |
| PRY | 2020 | DTU_2020_TWIW_01_PRY_ASU_HSWI_019 | Blood | ERR10431998 |
| PRY | 2020 | DTU_2020_TWIW_01_PRY_ASU_HSWI_020 | Blood | ERR10432262 |
| PRY | 2020 | DTU_2020_TWIW_01_PRY_ASU_HSWI_021 | Urine | ERR10431763 |
| PRY | 2020 | DTU_2020_TWIW_01_PRY_ASU_HSWI_022 | Respiratory System | ERR10431680 |
| PRY | 2020 | DTU_2020_TWIW_01_PRY_ASU_HSWI_023 | Blood | ERR10431678 |
| PRY | 2020 | DTU_2020_TWIW_01_PRY_ASU_HSWI_024 | Urine | ERR10431708 |
| PRY | 2020 | DTU_2020_TWIW_01_PRY_ASU_HSWI_025 | Urine | ERR10432289 |
| PRY | 2020 | DTU_2020_TWIW_01_PRY_ASU_HSWI_026 | Urine | ERR10431641 |
| PRY | 2020 | DTU_2020_TWIW_01_PRY_ASU_HSWI_027 | Other | ERR10432297 |
| PRY | 2020 | DTU_2020_TWIW_01_PRY_ASU_HSWI_028 | Other | ERR10431968 |
| PRY | 2020 | DTU_2020_TWIW_01_PRY_ASU_HSWI_029 | Urine | ERR10431774 |
| PRY | 2020 | DTU_2020_TWIW_01_PRY_ASU_HSWI_030 | Urine | ERR10431880 |
| PRY | 2020 | DTU_2020_TWIW_01_PRY_ASU_HSWI_031 | Blood | ERR10431966 |
| PRY | 2020 | DTU_2020_TWIW_01_PRY_ASU_HSWI_032 | Swab | ERR10431639 |
| PRY | 2020 | DTU_2020_TWIW_01_PRY_ASU_HSWI_033 | Wound Pus Biopsy | ERR10431942 |
| PRY | 2020 | DTU_2020_TWIW_01_PRY_ASU_HSWI_034 | Other | ERR10431986 |
| PRY | 2020 | DTU_2020_TWIW_01_PRY_ASU_HSWI_035 | Other | ERR10431785 |
| PRY | 2020 | DTU_2020_TWIW_01_PRY_ASU_HSWI_036 | Blood | ERR10431654 |
| PRY | 2020 | DTU_2020_TWIW_01_PRY_ASU_HSWI_037 | Blood | ERR10432295 |
| PRY | 2020 | DTU_2020_TWIW_01_PRY_ASU_HSWI_038 | Blood | ERR10432278 |
| PRY | 2020 | DTU_2020_TWIW_01_PRY_ASU_HSWI_039 | Blood | ERR10432267 |
| PRY | 2020 | DTU_2020_TWIW_01_PRY_ASU_HSWI_040 | Blood | ERR10431656 |
| PRY | 2020 | DTU_2020_TWIW_01_PRY_ASU_HSWI_041 | Other | ERR10431788 |
| PRY | 2020 | DTU_2020_TWIW_01_PRY_ASU_HSWI_042 | Wound Pus Biopsy | ERR10431787 |
| PRY | 2020 | DTU_2020_TWIW_01_PRY_ASU_HSWI_043 | Urine | ERR10431657 |
| PRY | 2020 | DTU_2020_TWIW_01_PRY_ASU_HSWI_044 | Urine | ERR10431799 |
| PRY | 2020 | DTU_2020_TWIW_01_PRY_ASU_HSWI_045 | Blood | ERR10432243 |
| PRY | 2020 | DTU_2020_TWIW_01_PRY_ASU_HSWI_046 | Urine | ERR10431645 |
| PRY | 2020 | DTU_2020_TWIW_01_PRY_ASU_HSWI_047 | Urine | ERR10431892 |
| PRY | 2020 | DTU_2020_TWIW_01_PRY_ASU_HSWI_048 | Urine | ERR10431804 |
| PRY | 2020 | DTU_2020_TWIW_01_PRY_ASU_HSWI_049 | Urine | ERR10431814 |
| PRY | 2020 | DTU_2020_TWIW_01_PRY_ASU_HSWI_050 | Urine | ERR10431903 |
| PRY | 2020 | DTU_2020_TWIW_01_PRY_ASU_HSWI_051 | Urine | ERR10432334 |
| PRY | 2020 | DTU_2020_TWIW_01_PRY_ASU_HSWI_052 | Other | ERR10431983 |
| PRY | 2020 | DTU_2020_TWIW_01_PRY_ASU_HSWI_053 | Wound Pus Biopsy | ERR10431703 |
| PRY | 2020 | DTU_2020_TWIW_01_PRY_ASU_HSWI_054 | Wound Pus Biopsy | ERR10431985 |
| PRY | 2020 | DTU_2020_TWIW_01_PRY_ASU_HSWI_055 | Urine | ERR10431820 |
| PRY | 2020 | DTU_2020_TWIW_01_PRY_ASU_HSWI_056 | Wound Pus Biopsy | ERR10432288 |
| PRY | 2020 | DTU_2020_TWIW_01_PRY_ASU_HSWI_057 | Respiratory System | ERR10431662 |
| PRY | 2020 | DTU_2020_TWIW_01_PRY_ASU_HSWI_058 | Respiratory System | ERR10432314 |
| PRY | 2020 | DTU_2020_TWIW_01_PRY_ASU_HSWI_059 | Urine | ERR10431908 |
| PRY | 2020 | DTU_2020_TWIW_01_PRY_ASU_HSWI_060 | Other | ERR10432286 |
| PRY | 2020 | DTU_2020_TWIW_01_PRY_ASU_HSWI_061 | Urine | ERR10431823 |
| PRY | 2020 | DTU_2020_TWIW_01_PRY_ASU_HSWI_062 | Wound Pus Biopsy | ERR10431993 |
| PRY | 2020 | DTU_2020_TWIW_01_PRY_ASU_HSWI_063 | Blood | ERR10432272 |
| PRY | 2020 | DTU_2020_TWIW_01_PRY_ASU_HSWI_064 | Blood | ERR10432283 |
| PRY | 2020 | DTU_2020_TWIW_01_PRY_ASU_HSWI_065 | Blood | ERR10432287 |
| PRY | 2020 | DTU_2020_TWIW_01_PRY_ASU_HSWI_065 | Blood | ERR14150548 |
| PRY | 2020 | DTU_2020_TWIW_01_PRY_ASU_HSWI_066 | Other | ERR10431994 |
| PRY | 2020 | DTU_2020_TWIW_01_PRY_ASU_HSWI_067 | Blood | ERR10432242 |
| PRY | 2020 | DTU_2020_TWIW_01_PRY_ASU_HSWI_068 | Wound Pus Biopsy | ERR10432298 |
| PRY | 2020 | DTU_2020_TWIW_01_PRY_ASU_HSWI_069 | Wound Pus Biopsy | ERR10432291 |
| PRY | 2020 | DTU_2020_TWIW_01_PRY_ASU_HSWI_069 | Wound Pus Biopsy | ERR14150995 |
| PRY | 2020 | DTU_2020_TWIW_01_PRY_ASU_HSWI_070 | Blood | ERR10432273 |
| PRY | 2020 | DTU_2020_TWIW_01_PRY_CIU_FMNU_008A | Urine | ERR10432301 |
| PRY | 2020 | DTU_2020_TWIW_01_PRY_CIU_FMNU_014A | Blood | ERR10432313 |
| PRY | 2020 | DTU_2020_TWIW_01_PRY_CIU_FMNU_036A | Urine | ERR10431928 |
| PRY | 2020 | DTU_2020_TWIW_01_PRY_ASU_CLPH_054A | Blood | ERR10432290 |
| THA | 2020 | DTU_2020_TWIW_01_THA_BAN_001 | Blood | ERR10432704 |
| THA | 2020 | DTU_2020_TWIW_01_THA_BAN_002 | Wound Pus Biopsy | ERR10432664 |
| THA | 2020 | DTU_2020_TWIW_01_THA_BAN_003 | Urine | ERR10432641 |
| THA | 2020 | DTU_2020_TWIW_01_THA_BAN_004 | Respiratory System | ERR10432584 |
| THA | 2020 | DTU_2020_TWIW_01_THA_BAN_005 | Respiratory System | ERR10432685 |
| THA | 2020 | DTU_2020_TWIW_01_THA_BAN_006 | Urine | ERR10432638 |
| THA | 2020 | DTU_2020_TWIW_01_THA_BAN_007 | Wound Pus Biopsy | ERR10432622 |
| THA | 2020 | DTU_2020_TWIW_01_THA_BAN_008 | Urine | ERR10432720 |
| THA | 2020 | DTU_2020_TWIW_01_THA_BAN_009 | Urine | ERR10432582 |
| THA | 2020 | DTU_2020_TWIW_01_THA_BAN_010 | Urine | ERR10432646 |
| THA | 2020 | DTU_2020_TWIW_01_THA_BAN_011 | Urine | ERR10432665 |
| THA | 2020 | DTU_2020_TWIW_01_THA_BAN_012 | Urine | ERR10432679 |
| THA | 2020 | DTU_2020_TWIW_01_THA_BAN_013 | Respiratory System | ERR10432588 |
| THA | 2020 | DTU_2020_TWIW_01_THA_BAN_014 | Respiratory System | ERR10432647 |
| THA | 2020 | DTU_2020_TWIW_01_THA_BAN_014 | Respiratory System | ERR14151015 |
| THA | 2020 | DTU_2020_TWIW_01_THA_BAN_015 | Respiratory System | ERR10432715 |
| THA | 2020 | DTU_2020_TWIW_01_THA_BAN_016 | Respiratory System | ERR10432583 |
| THA | 2020 | DTU_2020_TWIW_01_THA_BAN_017 | Respiratory System | ERR10432625 |
| THA | 2020 | DTU_2020_TWIW_01_THA_BAN_018 | Respiratory System | ERR10432586 |
| THA | 2020 | DTU_2020_TWIW_01_THA_BAN_019 | Respiratory System | ERR10432710 |
| THA | 2020 | DTU_2020_TWIW_01_THA_BAN_020 | Respiratory System | ERR10432587 |
| THA | 2020 | DTU_2020_TWIW_01_THA_BAN_021 | Urine | ERR10432688 |
| THA | 2020 | DTU_2020_TWIW_01_THA_BAN_022 | Urine | ERR10432590 |
| THA | 2020 | DTU_2020_TWIW_01_THA_BAN_023 | Respiratory System | ERR10432593 |
| THA | 2020 | DTU_2020_TWIW_01_THA_BAN_024 | Urine | ERR10432649 |
| THA | 2020 | DTU_2020_TWIW_01_THA_BAN_025 | Urine | ERR10432698 |
| THA | 2020 | DTU_2020_TWIW_01_THA_BAN_026 | Respiratory System | ERR10432595 |
| THA | 2020 | DTU_2020_TWIW_01_THA_BAN_027 | Respiratory System | ERR10432705 |
| THA | 2020 | DTU_2020_TWIW_01_THA_BAN_028 | Respiratory System | ERR10432596 |
| THA | 2020 | DTU_2020_TWIW_01_THA_BAN_029 | Respiratory System | ERR10432669 |
| THA | 2020 | DTU_2020_TWIW_01_THA_BAN_030 | Urine | ERR10432670 |
| THA | 2020 | DTU_2020_TWIW_01_THA_BAN_031 | Respiratory System | ERR10432672 |
| THA | 2020 | DTU_2020_TWIW_01_THA_BAN_032 | Respiratory System | ERR10432681 |
| THA | 2020 | DTU_2020_TWIW_01_THA_BAN_033 | Respiratory System | ERR10432709 |
| THA | 2020 | DTU_2020_TWIW_01_THA_BAN_034 | Respiratory System | ERR10432706 |
| THA | 2020 | DTU_2020_TWIW_01_THA_BAN_035 | Wound Pus Biopsy | ERR10432697 |
| THA | 2020 | DTU_2020_TWIW_01_THA_BAN_036 | Urine | ERR10432656 |
| THA | 2020 | DTU_2020_TWIW_01_THA_BAN_037 | Respiratory System | ERR10432598 |
| THA | 2020 | DTU_2020_TWIW_01_THA_BAN_038 | Urine | ERR10432597 |
| THA | 2020 | DTU_2020_TWIW_01_THA_BAN_039 | Urine | ERR10432677 |
| THA | 2020 | DTU_2020_TWIW_01_THA_BAN_040 | Urine | ERR10432620 |
| THA | 2020 | DTU_2020_TWIW_01_THA_BAN_041 | Respiratory System | ERR10432599 |
| THA | 2020 | DTU_2020_TWIW_01_THA_BAN_042 | Respiratory System | ERR10432671 |
| THA | 2020 | DTU_2020_TWIW_01_THA_BAN_043 | Urine | ERR10432723 |
| THA | 2020 | DTU_2020_TWIW_01_THA_BAN_044 | Urine | ERR10432678 |
| THA | 2020 | DTU_2020_TWIW_01_THA_BAN_045 | Urine | ERR10432655 |
| THA | 2020 | DTU_2020_TWIW_01_THA_BAN_046 | Respiratory System | ERR10432707 |
| THA | 2020 | DTU_2020_TWIW_01_THA_BAN_047 | Respiratory System | ERR10432601 |
| THA | 2020 | DTU_2020_TWIW_01_THA_BAN_048 | Wound Pus Biopsy | ERR10432591 |
| THA | 2020 | DTU_2020_TWIW_01_THA_BAN_049 | Wound Pus Biopsy | ERR10432689 |
| THA | 2020 | DTU_2020_TWIW_01_THA_BAN_049 | Wound Pus Biopsy | ERR14129409 |
| THA | 2020 | DTU_2020_TWIW_01_THA_BAN_050 | Respiratory System | ERR10432674 |
| THA | 2020 | DTU_2020_TWIW_01_THA_BAN_051 | Respiratory System | ERR10432604 |
| THA | 2020 | DTU_2020_TWIW_01_THA_BAN_052 | Respiratory System | ERR10432686 |
| THA | 2020 | DTU_2020_TWIW_01_THA_BAN_053 | Urine | ERR10432660 |
| THA | 2020 | DTU_2020_TWIW_01_THA_BAN_055 | Respiratory System | ERR10432603 |
| THA | 2020 | DTU_2020_TWIW_01_THA_BAN_056 | Urine | ERR10432702 |
| THA | 2020 | DTU_2020_TWIW_01_THA_BAN_057 | Urine | ERR10432652 |
| THA | 2020 | DTU_2020_TWIW_01_THA_BAN_058 | Respiratory System | ERR10432718 |
| THA | 2020 | DTU_2020_TWIW_01_THA_BAN_059 | Blood | ERR10432667 |
| THA | 2020 | DTU_2020_TWIW_01_THA_BAN_060 | Blood | ERR10432666 |
| BGD | 2020 | DTU_2020_TWIW_BGD_DHA_001 | Urine | ERR10441123 |
| BGD | 2020 | DTU_2020_TWIW_BGD_DHA_002 | Respiratory System | ERR10431363 |
| BGD | 2020 | DTU_2020_TWIW_BGD_DHA_003 | Respiratory System | ERR10435984 |
| BGD | 2020 | DTU_2020_TWIW_BGD_DHA_004 | Wound Pus Biopsy | ERR10436010 |
| BGD | 2020 | DTU_2020_TWIW_BGD_DHA_005 | Urine | NA |
| BGD | 2020 | DTU_2020_TWIW_BGD_DHA_006 | Urine | ERR10436087 |
| BGD | 2020 | DTU_2020_TWIW_BGD_DHA_008 | Urine | ERR10436029 |
| BGD | 2020 | DTU_2020_TWIW_BGD_DHA_009 | Respiratory System | ERR10435980 |
| BGD | 2020 | DTU_2020_TWIW_BGD_DHA_010 | Respiratory System | ERR10435920 |
| BGD | 2020 | DTU_2020_TWIW_BGD_DHA_012 | Blood | ERR10436071 |
| BGD | 2020 | DTU_2020_TWIW_BGD_DHA_013 | Urine | ERR10446200 |
| BGD | 2020 | DTU_2020_TWIW_BGD_DHA_014 | Respiratory System | ERR10435974 |
| BGD | 2020 | DTU_2020_TWIW_BGD_DHA_016 | Respiratory System | ERR10441066 |
| BGD | 2020 | DTU_2020_TWIW_BGD_DHA_018 | Wound Pus Biopsy | ERR10436169 |
| BGD | 2020 | DTU_2020_TWIW_BGD_DHA_019 | Respiratory System | ERR10431355 |
| BGD | 2020 | DTU_2020_TWIW_BGD_DHA_020 | Wound Pus Biopsy | ERR10435883 |
| BGD | 2020 | DTU_2020_TWIW_BGD_DHA_021 | Urine | ERR10431361 |
| BGD | 2020 | DTU_2020_TWIW_BGD_DHA_024 | Blood | ERR10436173 |
| BGD | 2020 | DTU_2020_TWIW_BGD_DHA_026 | Urine | ERR10435876 |
| BGD | 2020 | DTU_2020_TWIW_BGD_DHA_027 | Urine | ERR10441095 |
| BGD | 2020 | DTU_2020_TWIW_BGD_DHA_028 | Other | ERR10431352 |
| BGD | 2020 | DTU_2020_TWIW_BGD_DHA_029 | Urine | ERR10435985 |
| BGD | 2020 | DTU_2020_TWIW_BGD_DHA_030 | Urine | ERR10435942 |
| BGD | 2020 | DTU_2020_TWIW_BGD_DHA_031 | Other | ERR10435877 |
| BGD | 2020 | DTU_2020_TWIW_BGD_DHA_032 | Blood | ERR10436061 |
| BGD | 2020 | DTU_2020_TWIW_BGD_DHA_033 | Urine | ERR10435916 |
| BGD | 2020 | DTU_2020_TWIW_BGD_DHA_035 | Urine | ERR10435999 |
| BGD | 2020 | DTU_2020_TWIW_BGD_DHA_039 | Wound Pus Biopsy | ERR10431364 |
| BGD | 2020 | DTU_2020_TWIW_BGD_DHA_040 | Urine | ERR10441104 |
| BGD | 2020 | DTU_2020_TWIW_BGD_DHA_041 | Urine | ERR10441084 |
| BGD | 2020 | DTU_2020_TWIW_BGD_DHA_042 | Other | ERR10436171 |
| BGD | 2020 | DTU_2020_TWIW_BGD_DHA_043 | Wound Pus Biopsy | ERR10436065 |
| BGD | 2020 | DTU_2020_TWIW_BGD_DHA_044 | Respiratory System | ERR10431360 |
| BGD | 2020 | DTU_2020_TWIW_BGD_DHA_045 | Wound Pus Biopsy | ERR10436168 |
| BGD | 2020 | DTU_2020_TWIW_BGD_DHA_046 | Urine | ERR10441080 |
| BGD | 2020 | DTU_2020_TWIW_BGD_DHA_047 | Other | ERR10436174 |
| BGD | 2020 | DTU_2020_TWIW_BGD_DHA_048 | Respiratory System | ERR10441077 |
| BGD | 2020 | DTU_2020_TWIW_BGD_DHA_049 | Wound Pus Biopsy | ERR10431370 |
| BGD | 2020 | DTU_2020_TWIW_BGD_DHA_050 | Wound Pus Biopsy | ERR10431359 |
| BGD | 2020 | DTU_2020_TWIW_BGD_DHA_051 | Respiratory System | ERR10441060 |
| BGD | 2020 | DTU_2020_TWIW_BGD_DHA_052 | Blood | ERR10436170 |
| BGD | 2020 | DTU_2020_TWIW_BGD_DHA_053 | Blood | ERR10436088 |
| BGD | 2020 | DTU_2020_TWIW_BGD_DHA_054 | Urine | ERR10441078 |
| BGD | 2020 | DTU_2020_TWIW_BGD_DHA_059 | Urine | ERR10441087 |
| BGD | 2020 | DTU_2020_TWIW_BGD_DHA_060 | Urine | ERR10436081 |
| NGA | 2020 | DTU_2020_TWIW_01_NGA_IBARAP_016 | Blood | ERR10438978 |
| NGA | 2020 | DTU_2020_TWIW_01_NGA_IBARAP_017 | Wound Pus Biopsy | ERR10438864 |
| NGA | 2020 | DTU_2020_TWIW_01_NGA_IBARAP_018 | Other | ERR10439053 |
| NGA | 2020 | DTU_2020_TWIW_01_NGA_IBARAP_019 | Swab | ERR10438865 |
| NGA | 2020 | DTU_2020_TWIW_01_NGA_IBARAP_020 | Blood | ERR10438839 |
| NGA | 2020 | DTU_2020_TWIW_01_NGA_IBARAP_021 | Swab | ERR10438823 |
| NGA | 2020 | DTU_2020_TWIW_01_NGA_IBARAP_022 | Other | ERR10438877 |
| NGA | 2020 | DTU_2020_TWIW_01_NGA_IBARAP_023 | Other | ERR10438904 |
| NGA | 2020 | DTU_2020_TWIW_01_NGA_IBARAP_024 | Wound Pus Biopsy | ERR10439007 |
| NGA | 2020 | DTU_2020_TWIW_01_NGA_IBARAP_025 | Wound Pus Biopsy | ERR10438982 |
| NGA | 2020 | DTU_2020_TWIW_01_NGA_IBARAP_026 | Wound Pus Biopsy | ERR10439013 |
| NGA | 2020 | DTU_2020_TWIW_01_NGA_IBARAP_027 | Blood | ERR10438840 |
| NGA | 2020 | DTU_2020_TWIW_01_NGA_IBARAP_028 | Urine | ERR10438835 |
| NGA | 2020 | DTU_2020_TWIW_01_NGA_IBARAP_029 | Wound Pus Biopsy | ERR10438988 |
| NGA | 2020 | DTU_2020_TWIW_01_NGA_IBARAP_030 | Wound Pus Biopsy | ERR10438963 |
| NGA | 2020 | DTU_2020_TWIW_01_NGA_IBARAP_032 | Other | ERR10438955 |
| NGA | 2020 | DTU_2020_TWIW_01_NGA_IBARAP_033 | Wound Pus Biopsy | ERR10438836 |
| NGA | 2020 | DTU_2020_TWIW_01_NGA_IBARAP_034 | Wound Pus Biopsy | ERR10438956 |
| NGA | 2020 | DTU_2020_TWIW_01_NGA_IBARAP_035 | Urine | ERR10438903 |
| NGA | 2020 | DTU_2020_TWIW_01_NGA_IBARAP_036 | Wound Pus Biopsy | ERR10438958 |
| NGA | 2020 | DTU_2020_TWIW_01_NGA_IBARAP_037 | Wound Pus Biopsy | ERR10438834 |
| NGA | 2020 | DTU_2020_TWIW_01_NGA_IBARAP_038 | Wound Pus Biopsy | ERR10438902 |
| NGA | 2020 | DTU_2020_TWIW_01_NGA_IBARAP_039 | Urine | ERR10438906 |
| NGA | 2020 | DTU_2020_TWIW_01_NGA_IBARAP_040 | Urine | ERR10438907 |
| NGA | 2020 | DTU_2020_TWIW_01_NGA_IBARAP_041 | Wound Pus Biopsy | ERR10438911 |
| MOZ | 2020 | DTU_2020_TWIW_01_MOZ_MAN_001 | Urine | ERR10438710 |
| MOZ | 2020 | DTU_2020_TWIW_01_MOZ_MAN_002 | Urine | ERR10438732 |
| MOZ | 2020 | DTU_2020_TWIW_01_MOZ_MAN_003 | Other | ERR10438730 |
| MOZ | 2020 | DTU_2020_TWIW_01_MOZ_MAN_004 | Blood | ERR10438726 |
| MOZ | 2020 | DTU_2020_TWIW_01_MOZ_MAN_005 | Urine | ERR10438717 |
| MOZ | 2020 | DTU_2020_TWIW_01_MOZ_MAN_007 | Urine | ERR10438721 |
| MOZ | 2020 | DTU_2020_TWIW_01_MOZ_QUE_001 | Blood | ERR10438722 |
| MOZ | 2020 | DTU_2020_TWIW_01_MOZ_QUE_002 | Blood | ERR10438727 |
| MOZ | 2020 | DTU_2020_TWIW_01_MOZ_QUE_003 | Blood | ERR10438712 |
| MOZ | 2020 | DTU_2020_TWIW_01_MOZ_QUE_004 | Urine | ERR10438728 |
| MOZ | 2020 | DTU_2020_TWIW_01_MOZ_QUE_005 | Urine | ERR10438743 |
| MOZ | 2020 | DTU_2020_TWIW_01_MOZ_QUE_006 | Other | ERR10438715 |
| MOZ | 2020 | DTU_2020_TWIW_01_MOZ_QUE_007 | Other | ERR10438714 |
| MOZ | 2020 | DTU_2020_TWIW_01_MOZ_QUE_008 | Wound Pus Biopsy | ERR10438738 |
| MOZ | 2020 | DTU_2020_TWIW_01_MOZ_QUE_009 | Blood | ERR10438724 |
| MOZ | 2020 | DTU_2020_TWIW_01_MOZ_QUE_010 | Blood | ERR10438737 |
| MOZ | 2020 | DTU_2020_TWIW_01_MOZ_QUE_011 | Other | ERR10438729 |
| MOZ | 2020 | DTU_2020_TWIW_01_MOZ_QUE_012 | Blood | ERR10438744 |
| MOZ | 2020 | DTU_2020_TWIW_01_MOZ_QUE_013 | Blood | ERR10438707 |
| MOZ | 2020 | DTU_2020_TWIW_01_MOZ_QUE_014 | Blood | ERR10502526 |
| MOZ | 2020 | DTU_2020_TWIW_01_MOZ_QUE_015 | Blood | ERR10438734 |
| MOZ | 2020 | DTU_2020_TWIW_01_MOZ_QUE_016 | Urine | ERR10438716 |
| MOZ | 2020 | DTU_2020_TWIW_01_MOZ_QUE_017 | Urine | ERR10438713 |
| MOZ | 2020 | DTU_2020_TWIW_01_MOZ_QUE_018 | Other | ERR10438741 |
| MOZ | 2020 | DTU_2020_TWIW_01_MOZ_QUE_019 | Other | ERR10438731 |
| MOZ | 2020 | DTU_2020_TWIW_01_MOZ_QUE_020 | Other | ERR10438735 |
| MOZ | 2020 | DTU_2020_TWIW_01_MOZ_QUE_021 | Other | ERR10438742 |
| MOZ | 2020 | DTU_2020_TWIW_01_MOZ_QUE_022 | Blood | ERR10438733 |
| MOZ | 2020 | DTU_2020_TWIW_01_MOZ_QUE_023 | Urine | ERR10438745 |
| MOZ | 2020 | DTU_2020_TWIW_01_MOZ_QUE_024 | Urine | ERR10438720 |
| MOZ | 2020 | DTU_2020_TWIW_01_MOZ_QUE_026 | Wound Pus Biopsy | ERR10438718 |
| MOZ | 2020 | DTU_2020_TWIW_01_MOZ_QUE_027 | Wound Pus Biopsy | ERR10438719 |
| MOZ | 2020 | DTU_2020_TWIW_01_MOZ_QUE_028 | Blood | ERR10438739 |
| MOZ | 2020 | DTU_2020_TWIW_01_MOZ_QUE_030 | Wound Pus Biopsy | ERR10438709 |
| MOZ | 2020 | DTU_2020_TWIW_01_MOZ_QUE_031 | Wound Pus Biopsy | ERR10438736 |
| MOZ | 2020 | DTU_2020_TWIW_01_MOZ_QUE_033 | Wound Pus Biopsy | ERR10438747 |
| MOZ | 2020 | DTU_2020_TWIW_01_MOZ_QUE_034 | Wound Pus Biopsy | ERR10438723 |
| MOZ | 2020 | DTU_2020_TWIW_01_MOZ_QUE_035 | Wound Pus Biopsy | ERR10438740 |
| MOZ | 2020 | DTU_2020_TWIW_01_MOZ_QUE_036 | Wound Pus Biopsy | ERR10438746 |
| MOZ | 2020 | DTU_2020_TWIW_01_MOZ_QUE_037 | Wound Pus Biopsy | ERR10438711 |
| MOZ | 2020 | DTU_2020_TWIW_01_MOZ_MAN_001A | Urine | ERR10438725 |
| PRY | 2020 | DTU_2020_TWIW_01_PRY_ASU_HSWI_015A | Wound Pus Biopsy | ERR10432307 |
| PRY | 2020 | DTU_2020_TWIW_01_PRY_ASU_HSWI_038A | Blood | ERR10502527 |

# Table S5

Spearman’s rho, p-value for correlation and q-value (adjusted p-value) for ARGs from the correlation analysis (Fig. 4). See more details in methods section.

| **ARG** | **Spearman correlation** | **p_value** | **q_value** |
| --- | --- | --- | --- |
| sul2_2 | 0.75213675 | 0.000011092 | 0.000310586 |
| tet(B)_3 | 0.73318306 | 0.000103676 | 0.001451465 |
| qnrS10_1 | 0.71785714 | 0.003569791 | 0.033318052 |
| aph(6)-Id_5 | 0.51428590 | 0.007191333 | 0.040271466 |
| dfrA12_10 | 0.61236078 | 0.006904533 | 0.040271466 |
| ant(6)-Ia_1 | 0.60522753 | 0.028395442 | 0.132512062 |
| aph(3')-Ia_1 | 0.44180798 | 0.039531942 | 0.158127767 |
| dfrA1_1 | 0.44427761 | 0.049703689 | 0.173962911 |
| dfrA32_1 | 0.36090226 | 0.118521746 | 0.266622247 |
| blaDHA-1_1 | 0.39705067 | 0.127814547 | 0.266622247 |
| catB7_1 | 0.40714286 | 0.133311123 | 0.266622247 |
| dfrG_1 | 0.43727879 | 0.103112804 | 0.266622247 |
| lsa(A)_3 | -0.44812799 | 0.108055270 | 0.266622247 |
| erm(C)_15 | 0.54103593 | 0.106317546 | 0.266622247 |
| tet(M)_9 | -0.37058824 | 0.158240173 | 0.295381657 |
| mph(A)_2 | 0.28126204 | 0.173199528 | 0.303099173 |
| aac(3)-IIa_3 | 0.25720763 | 0.247860504 | 0.389660450 |
| msr(C)_2 | 0.40121766 | 0.250496004 | 0.389660450 |
| aadA4_1 | -0.17877340 | 0.403257500 | 0.586911454 |
| aph(3')-III_1 | -0.16390977 | 0.488241914 | 0.586911454 |
| mph(E)_1 | 0.23158037 | 0.468929537 | 0.586911454 |
| msr(E)_1 | 0.22066584 | 0.490702491 | 0.586911454 |
| catB3_1 | -0.22727273 | 0.503066961 | 0.586911454 |
| sul3_2 | 0.26139938 | 0.465684706 | 0.586911454 |
| tet(D)_1 | 0.18481859 | 0.527033473 | 0.590277490 |
| dfrA30_1 | 0.18213688 | 0.571015781 | 0.614940072 |
| fosA5_1 | -0.11413525 | 0.641749257 | 0.665517748 |
| blaOXA-47_1 | -0.03410776 | 0.877217451 | 0.877217451 |
